# Enantioselective Addition of Remote Alkyl Radicals to Double Bonds by Photocatalytic Proton-Coupled Electron Transfer (PCET) Deconstruction of Unstrained Cycloalkanols

**DOI:** 10.1021/acs.orglett.2c00662

**Published:** 2022-04-01

**Authors:** Noelia Salaverri, Benedetta Carli, Sergio Díaz-Tendero, Leyre Marzo, José Alemán

**Affiliations:** † Departamento de Química Orgánica (Módulo 1), Universidad Autónoma de Madrid, Cantoblanco, 28049, Madrid, Spain; ‡ Institute for Advanced Research in Chemical Sciences (IAdChem), Universidad Autónoma de Madrid, 28049, Madrid, Spain; § Condensed Matter Physics Center (IFIMAC), Facultad de Ciencias, Universidad Autónoma de Madrid, Cantoblanco, 28049, Madrid, Spain; ∥ Departamento de Química (Módulo 13), Facultad de Ciencias, Universidad Autónoma de Madrid, Cantoblanco, 28049, Madrid, Spain

## Abstract

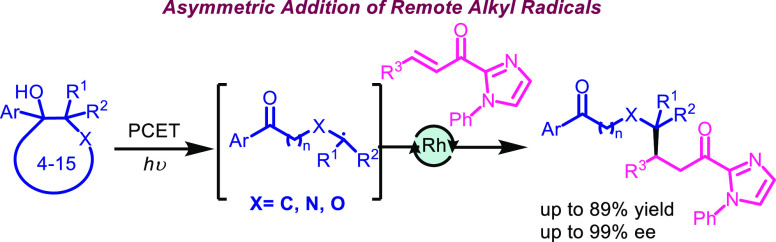

Herein, we report the enantioselective addition of remote alkyl radicals, generated from the ring opening of unstrained cycloalkanols by a proton-coupled electron transfer (PCET) process, to 2-acyl imidazoles previously coordinated to a rhodium-based chiral Lewis acid. High yields and enantioselectivites up to 99% are achieved in 1 h. Mechanistic investigations support the formation of the remote alkyl radical by a PCET process, and theoretical studies explain the observed stereochemistry in the addition step.

Proton-coupled electron transfer (PCET) processes play an essential role in biological redox reactions and consist of a concerted exchange of a proton and an electron in one step.^1^ This strategy allows the activation of substrates with high redox potentials because the favorable energetics related with the proton transfer step compensates the unfavorable energetics of the electron transfer event. Lately, this approach has been implemented in organic synthesis, giving access to the development of new transformations.^2^ The first example was reported by Knowles and involved a ketyl-olefin cyclization in which the coordination of the ketone to the phosphoric acid decreases the activation barrier of the reduction, enabling the single electron transfer (SET) step.^3a^ In addition, the oxidative PCET strategy has been applied in other racemic examples, such as the formation of new C–N bonds,^4^ or in the catalytic ring opening of unstrained cycloalkanols^5^ following a deconstructive strategy (Figure 1a).^6^

**Figure 1:**
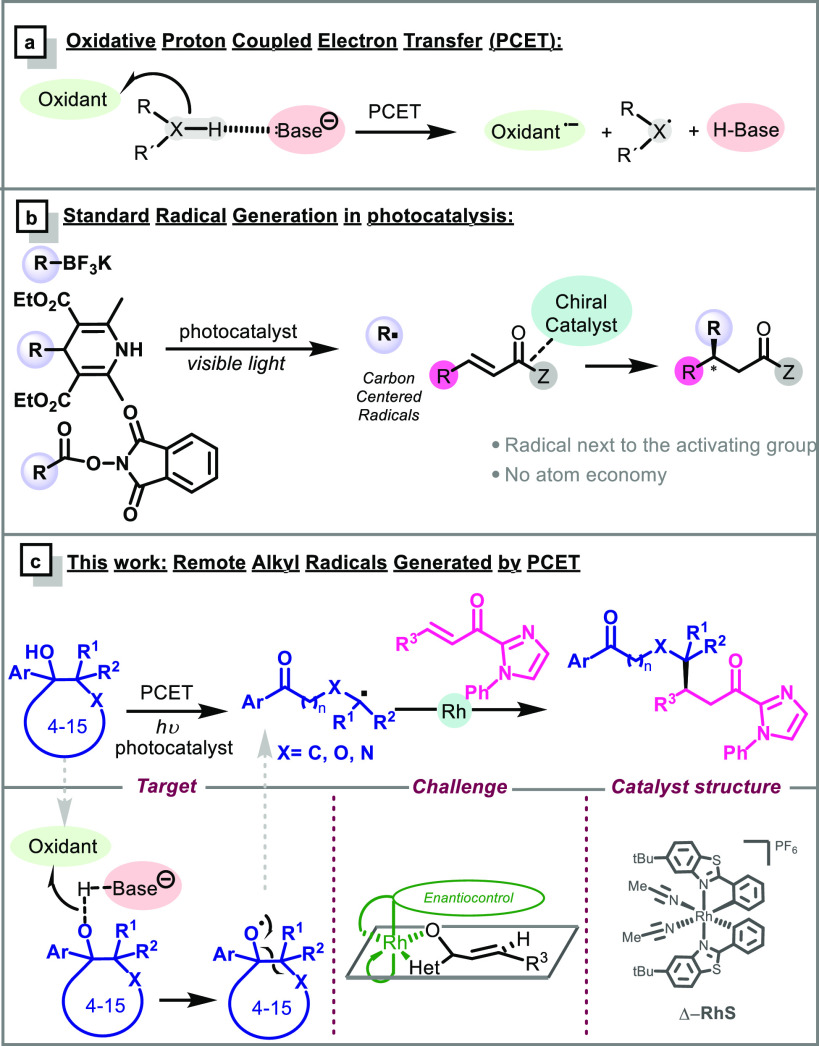
(a) Oxidative PCET process. (b) Lewis acid mediated enantioselective addition of alkyl radicals. (c) This work.

The addition of nucleophilic alkyl C-centered radicals to electron-deficient double bonds is a robust strategy, broadly employed for the construction of new C–C bonds (Figure 1b). However, the enantioselective version of this type of radical addition has been scarcely developed. To achieve this goal, two main catalytic asymmetric systems are employed. One system involves the use of chiral aminocatalysts,^7^ while the other approach involves the use of a chiral Lewis acid.^8^ In particular, among the methods that employ chiral Lewis acids, Megger’s group has developed a new family of chiral rhodium and iridium catalysts that have enabled the addition of simple alkyl radicals generated from potassium trifluoroborates,^9a^ Hantzsch esters,^9b^ or *N*-(acyloxy)phthalimides.^9c^ In this context, we hypothesized that, in the presence of a centrochiral complex,^8^ alkyl radicals of any length, generated in remote positions to a ketone via a ring-opening of unstrained cycloalkanols,^5^ could be introduced in an enantioselective manner (Figure 1c).

To probe the feasibility of our hypothesis, we carried out the reaction between the alcohol **1a** and the α,β-unsaturated-2-acyl imidazole **2a** in the presence of different photocatalysts as well as Lewis acids and bases. After intensive screening of the reaction conditions, compound **3a** was obtained in 79% isolated yield and 94% ee, using 2.5 mol % of the Mes-acridinium salt as a photocatalyst (*E*_PC^•–^/*PC_ = 2.18 V), 5 mol % of the rhodium complex and 2,6-lutidine as the base, in CH_2_Cl_2_, under blue LED irradiation in just 1 h (Table 1, entry 1). It is worth mentioning that the C–C bond scission took place exclusively at the α-position of the oxygen, with excellent regioselectivity for the formation of the most stabilized radical, which consecutively adds to **2a**. Control experiments corroborated the photocatalytic nature of the reaction when no conversion was obtained in the absence of light or a photocatalyst (entries 2–3). In addition, the PCET process was confirmed because the reaction did not work in the absence of base (entry 4). Moreover, the radical addition took place without the chiral Lewis acid and **3a** was formed in a 20% yield as a racemic mixture (Table 1, entry 5). This result is proof that the rhodium catalyst enhances the process and avoids the racemic background reaction. Other bases typically employed in PCET processes such as phosphates or a decrease in the catalyst loading of the rhodium complex afforded lower yields and enantioselectivities (Table 1, entries 6 and 7). A change in the concentration did not improve this result (entries 8 and 9), neither did different ratios of the alcohol **1a** and double bond **2a** (1:2 or 2:1) (Table 1, entries 10 and 11). The 2-acyl pyrazole as a heterocycle in the Michael acceptor was also tried, but a very low yield was obtained.

**Table 1:**
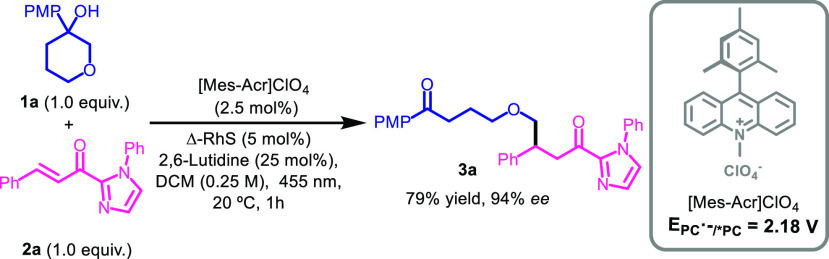
Optimization of the Reaction Conditions^a^

Entry	Deviation from standard conditions	Yield (%)	ee (%)
1	Standard^a^	79	94
2	No [Mes-Acr]ClO_4_	n.r.	–
3	No light	n.r.	–
4	No base	n.r.	–
5	No [Rh] catalyst	20	0
6	Phosphate^b^ instead of 2,6-lutidine	25	n.d.
7	[Rh] (2.5 mol %)	74	83
8	Concentration 0.1 M	91	80
9	Concentration 0.5 M	82	78
10	2.0 equiv of **2a**	81	84
11	2.0 equiv of **1a**	80	94

a Standard conditions: **1a** (0.05 mmol), **2a** (0.05 mmol), Lutidine (25 mol %), Mes-Acr (2.5 mol %), [Rh] (5 mol %), 0.250 mL of CH_2_Cl_2_, 455 nm LED, 20 °C, 1 h.

b Phosphate = (BuO)_2_P(O)ONBu_3_Me.

Encouraged by these results, we proceeded to study the scope of the reaction (Figure 2). First, we focused our attention towards the use of different tertiary alcohols **1** in their reactions with **2a**. In addition to oxygenated cycloalkanols (**3a**), other heteroatoms that were able to stabilize alkyl radicals such as amino groups also afforded the desired products (**3b**, **3c**). The deconstructive-enantioselective alkylation was not limited to six-membered rings, but also 4- and 5-membered rings bearing and heteroatom in its structure (**3d**, **3e**), as well as 6-, 8-, or 15-membered bicyclic spiro compounds were viable (**3f**–**3h**). These results proved that regardless the ring-strain energy, it is possible to carry out the alkylation with carbon chains of almost any length. In addition, the use of nonstabilized alkyl radicals also afforded the product with lower enantioselectivity (**3i**). Moreover, the robustness of the method was proven when **3e** could be obtained in 0.25 and 1.0 mmol scale (see Figure 2). To further prove the utility of the method, we next studied whether the *p*-methoxyphenyl (PMP) group could be placed in farther positions or if it could be substituted by other oxidable (hetero)-arenes. Placing the PMP group for the first time distally separated by a triple bond or in the 2-position of cyclohexanol also afforded **3j** and **3k**, proving that, even at distant positions, the PCET process can take place. Then, the reaction was carried out with cyclohexanols bearing a furan, benzofuran, or phenanthrene instead of the PMP group, obtaining moderate to excellent results in terms of yields and enantioselectivities (**3l**–**n**).^10^

**Figure 2:**
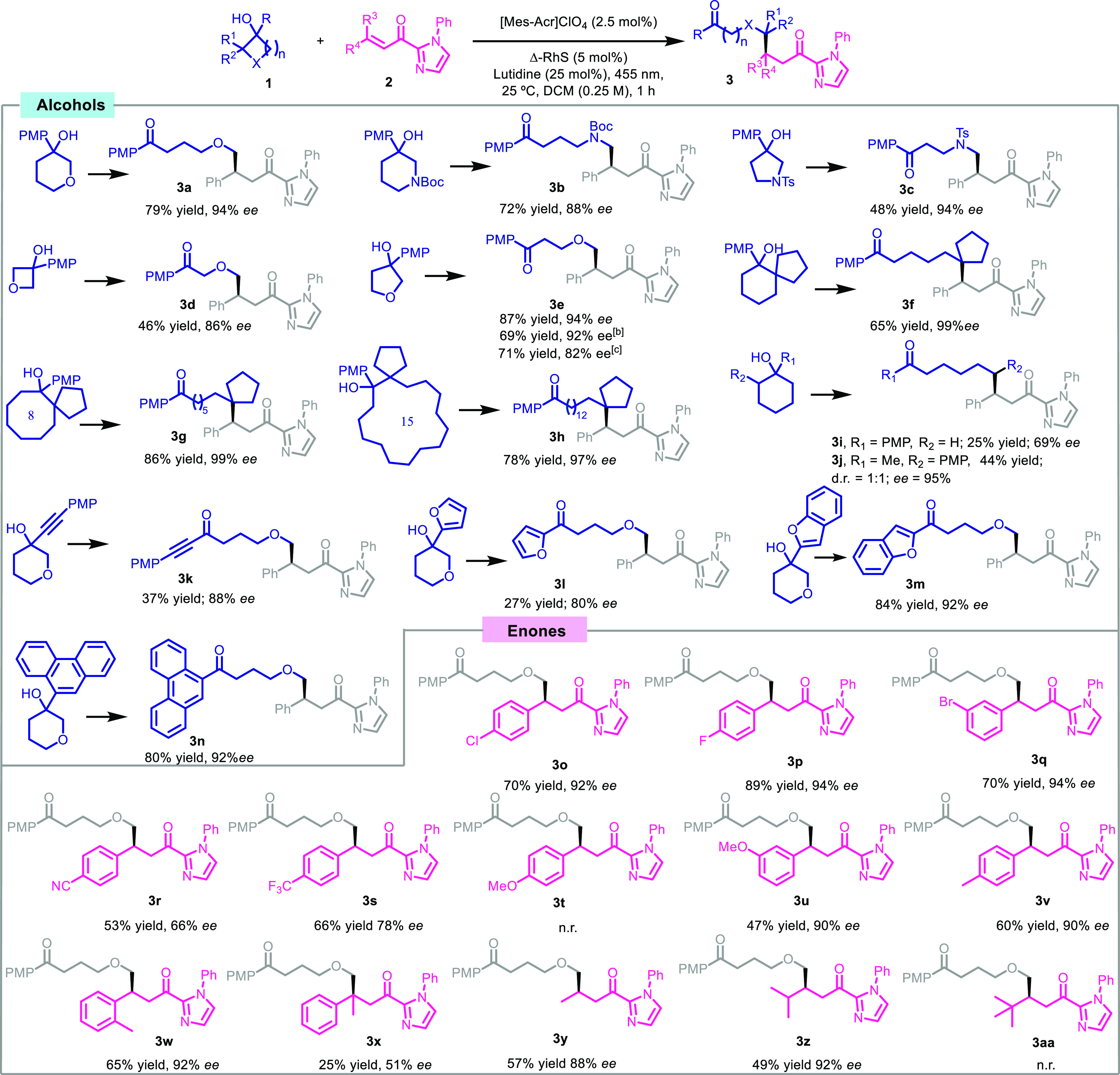
Scope of the enantioselective addition of remote alkyl radicals to enones.^*a*^^*a*^ Reactions performed using 0.05 mmol of **1**, 0.05 mmol of **2**, 2.5 mol % of Mes-acridinium, 5 mol % of rhodium complex, 25 mol % of 2,6-lutidine, 0.25 mL of DCM, under 1 h of blue LED irradiation. ^*b*^ The reaction was carried out in 5 times larger scale starting from 0.25 mmol of **1e** for 3h. ^*c*^ The reaction was carried out starting from 1.0 mmol of **1e**, using 2.5 mol % of Δ-RhS for 17 h.

Next, we turned our attention to the scope of the α,β-unsaturated 2-acyl imidazoles **2** in their reactions with the alcohol **1a**. The presence of halogen substituents in different positions of the aromatic ring did not affect the yield or enantioselectivity (**3o**–**3q**), but electron-withdrawing substituents in the *para* position provoked a decrease in the enantioselectivity (**3r**, **3s**).^11^ On the contrary, strong electron-donating substituents in the *para* position completely suppressed the reaction (**3t**), probably because the substrate could be oxidized by the photocatalyst as was corroborated by fluorescence quenching studies that evidenced the efficient interaction between the excited photocatalyst and the enone (see Supporting Information (SI)). Therefore, we next performed the reaction with the *meta*-methoxy-substituted enone, obtaining **3u** with good enantioselectivity, but moderate yield.^12^ Other electron-donating substituents in *para* or even *ortho* positions did not affect the reactivity (**3v**, **3w**). The formation of quaternary centers was also accessible albeit in lower yields and enantioselectivities (**3x**). Good results were obtained with enones bearing primary or secondary alkyl substituents (**3y**, **3z**), but with the most hindered *tert*-butyl group the reaction was completely suppressed (**3aa**).

Then, the conversion of the imidazole moiety to a versatile ester group was performed in good yield without degradation of the enantiomeric purity of the final product **4** (Scheme 1).

**Scheme 1:**
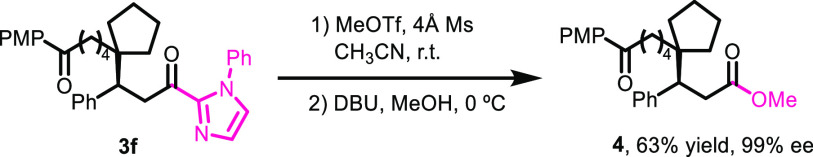
Transformation of the Imidazole in a Versatile Building Block

The mechanistic proposal is depicted in Figure 3a. Upon 455 nm LED irradiation, the acridinium catalyst is excited and oxidizes the *p*-methoxyphenyl ring, forming the radical cation intermediate **I**. This oxidizing step was further analyzed by steady state and time-resolved fluorescence quenching studies of the photocatalyst with the alcohol **1a**, a mixture of the alcohol **1a** and the base, and the enone **2a** (Figure 3c). The excited photocatalyst is only efficiently quenched by the alcohol **1a** and the mixture of **1a** and the base. Then, intermediate **I** undergoes a concerted intramolecular electron transfer and deprotonation of the alcohol by the base to form the alkoxyl radical **II**. The PCET process is confirmed by the fact that the reaction only takes place in the presence of the base (Table 1, entry 3). Intermediate **II** evolves through the scission of the β-C–C bond to form the stabilized α-oxy radical **III**. On the other hand, **2a** coordinates with the initial rhodium complex to form the *N,O*-rhodium-coordinated 2-acyl imidazole **IV** that suffers the addition of the alkyl radical **III**, through the less hindered face, generating the intermediate **V**. This radical **V** is further reduced by the radical anion of the photocatalyst to give **VI**, and after protonation (**VII**), the photocatalytic cycle is closed to afford **3a**. The quantum yield of the reaction is 0.05, discarding a possible radical chain, in which intermediate **V** can further oxidize the electron-rich arene.

**Figure 3:**
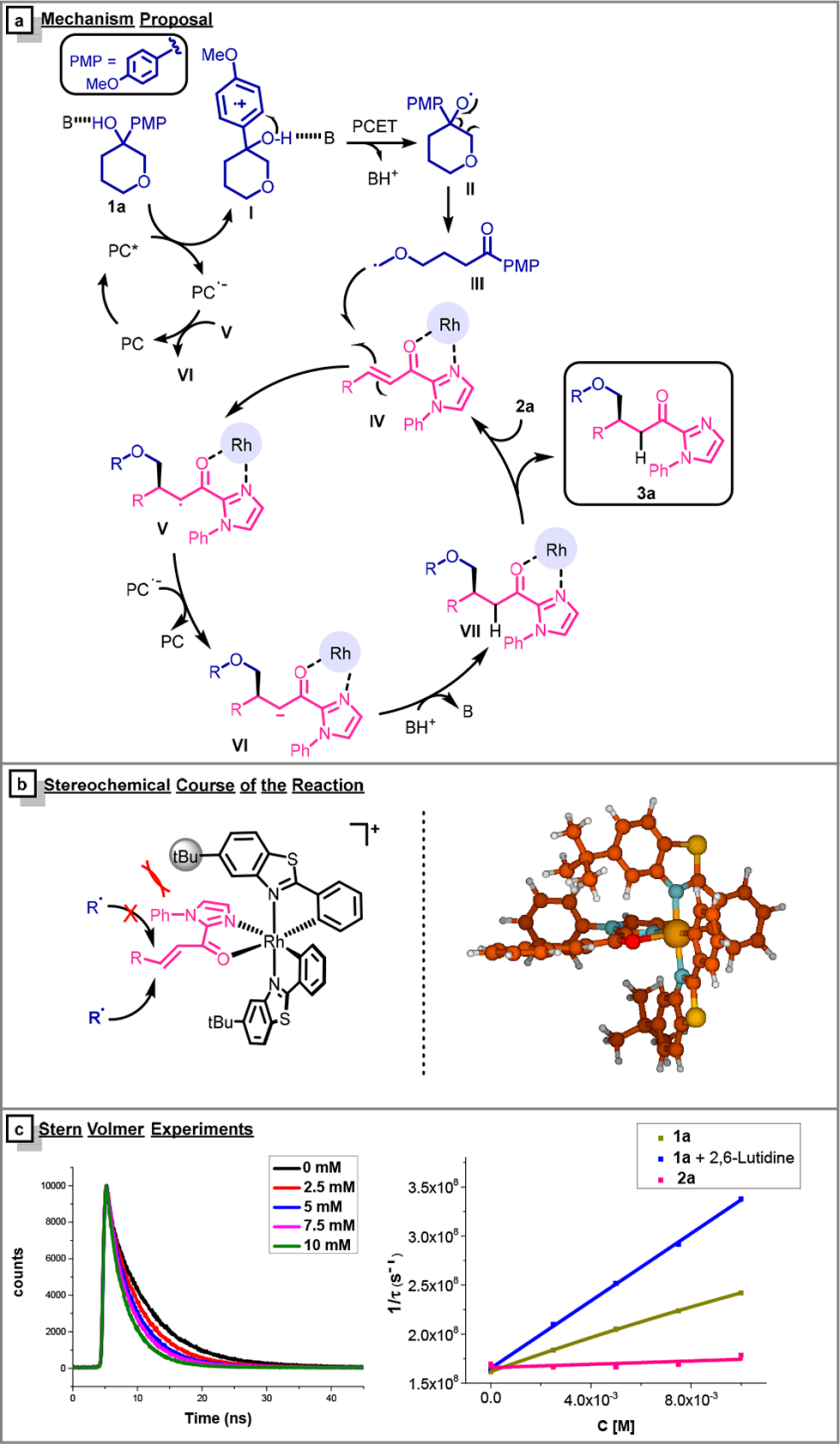
(a) Mechanistic proposal. (b) DFT Optimized structure of complex **IV**. (c) Time resolved fluorescence quenching studies of Mes-Acr with a mixture of **1a** and 2,6-lutidine (left) and Stern–Volmer plot of the time-resolved fluorescence quenching of Mes-Acr with the different components of the reaction (right).

The absolute configuration of the final product was assigned by correlation with a known compound in the literature (see SI). In addition, the geometry of intermediate **IV** was optimized (Figure 3b) using the density functional theory (DFT), with the B3LYP functional^13^ and including dispersion with the D3 method^14^ in combination with the Def2SVP basis set,^15^ as implemented in Gaussian16.^16^ As can be seen in Figure 3b, one of the faces is sterically blocked by the *tert*-butyl group, which explains the observed stereochemistry of the final products. When assuming the same stereochemical outcome, it was possible to assign the stereochemistry of the rest of compounds. These evidences are in agreement with the previously described stereochemical course for this catalytic system.^9^

In conclusion, here we reported the first enantioselective addition of remote alkyl radicals, generated by the PCET process from unstrained cycloalkanols, under visible light irradiation, to 2-acyl imidazoles coordinated to a chiral rhodium Lewis acid. This method will be of significant relevance for synthesis, since it allows the preparation of diketones with alkyl chains of any length bearing a chiral center, and it is compatible with a large variety of functional groups. Finally, mechanistic investigations support the mechanistic proposal and the stereochemical outcome of the reaction.

## Supporting Information Available

Experimental procedures and spectral data (PDF)
