# Supplementary material for: Enantioselective Addition of Remote Alkyl Radicals to Double Bonds by Photocatalytic Proton-Coupled Electron Transfer (PCET) Deconstruction of Unstrained Cycloalkanols
Source: Org Lett. 2022 Apr 1;24(17):3123–7. doi: 10.1021/acs.orglett.2c00662 (PMC9087350; doi:10.1021/acs.orglett.2c00662)
Supplement: Supplementary file 1 — ol2c00662_si_001.pdf [file ol2c00662_si_001.pdf]

Supplementary Information

Enantioselective Addition of Remote Alkyl Radicals to Double Bonds by Photocatalytic PCET Deconstruction of Unstrained Cycloalkanols

Noelia Salaverri,[a] Benedetta Carli,[a] Sergio Díaz-Tendero,[b, c, d] Leyre Marzo,*[a] José Alemán*[a,b]

[a] Organic Chemistry Department, Módulo 1, Universidad Autónoma de Madrid, Madrid 28049, Spain

[b] Institute for Advanced Research in Chemical Sciences (IAdChem), Universidad Autónoma de Madrid, Madrid 28049, Spain

[c] Condensed Matter Physics Center (IFIMAC), Facultad de Ciencias, Universidad Autónoma de Madrid, 28049 Madrid, Spain.

[d] Departamento de Química (Módulo 13), Facultad de Ciencias, Universidad Autónoma de Madrid, 28049 Madrid, Spain

E-mail: leyre.marzo@uam.es, jose.aleman@uam.es.

Table of contents

1. General methods and materials....................S3

2. Synthesis and characterization of alcohols **1** S5

3. Synthesis and characterization of α,β-unsaturated alkenes **2**....................S11

4. Synthesis and characterization of products **3**....................S17

5. Synthetic transformation....................S29

6. Mechanistic studies on the photocatalytic reaction....................S30

   a) Fluorescence quenching studies S30

   b) Cyclic voltammetry....................S31

   c) Quantum yield determination S32

7. Stereochemical assigments and computational details ….. ....S36

   a) Synthesis of **4ab** S36

   b) Computational details. DFT calculations....................S39

8. SFC Traces S43

9. NMR Spectra S56

10. References S113

1. General methods and materials

NMR spectra were acquired on a BRUKER AVANCE 300 or 500 MHz spectrometer running at 300 or 500 MHz for ^{1}H, 75 or 125 MHz for ^{13}C, 282 or 471 MHz for ^{19}F, and are internally referenced to residual solvent signals ($CDCl_3$ referenced at δ 7.26 ppm for ^{1}H NMR and δ 77.2 ppm for ^{13}C NMR). Data for ^{1}H NMR are reported as follows: chemical shift (δ ppm), multiplicity (s = singlet, d = doublet, t = triplet, quint = quintuplet, m = multiplet), coupling constant (Hz) and integration. Data for ^{13}C and ^{19}F are reported in terms of chemical shift.

High-Resolution Mass Spectra (HRMS) were obtained on an Agilent Technologies 6120 Quadrupole LC/MS coupled with an SFC Agilent Technologies 1260 Infinity Series instrument for the MS (ESI) (Electrospray Ionization). MassWorks software version 4.0.0.0 (Cerno Bioscience) was used for the formula identification. MassWorks is an MS calibration software which calibrates isotope profiles to achieve high mass accuracy and enables elemental composition determination on conventional mass spectrometers of unit mass resolution allowing highly accurate comparisons between calibrated and theoretical spectra.

Enantiomeric ratios were determined by Supercritical Fluid Chromatography (SFC) with chiral columns on an Agilent Technologies 1260 Infinity Series instrument, employing Daicel Chiralpak IA, IB-3, IC, ID-3 and IG-3 columns and a UV-Vis detector. The exact conditions for the analyses are specified in each case.

Optical rotations were measured on a Perkin-Elmer 241 MC Polarimeter and are reported as follows: $[\alpha]_D^{r.t.}$ (c in g/100 mL, solvent).

Commercial grade reagents and solvents were purchased from Acros Organics, Alfa Aesar, Fluorochem, Sigma-Aldrich and TCI Chemicals, and used as received without further purification. THF and toluene were purified by passing through a Pure Solv^{TM} column drying system from Innovative Technology, Inc.

Analytical TLC was performed using pre-coated aluminum-backed plates (Merck TLC Silicagel 60 F_{254}) and visualized by ultraviolet irradiation. Chromatographic purification of products was accomplished by flash chromatography using silica gel (Merck Geduran® Si 60) or aluminum oxide (activated, basic, Brockmann I).

A custom-made photoreactor setup was used for the photocatalytic reactions. The vial is placed inside the fitted well in which irradiation takes place at the desired wavelengths (365, 385, 420, 450 or 540 nm) using 380 mW single LEDs. Reaction temperature is kept at 20-25 ºC using a recirculating chiller.

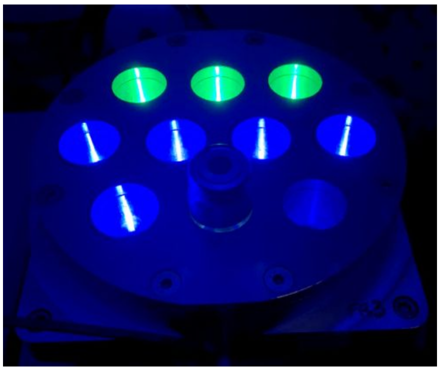

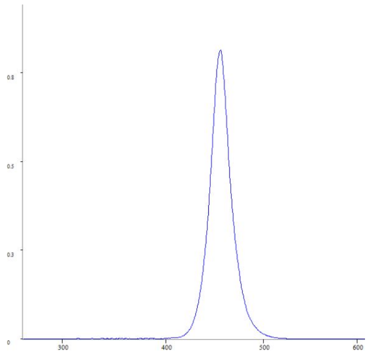

Fig. S1. Experimental setup employed during photocatalytic reactions and emission spectrum of the LED used.

UV-Vis measurements were carried out on an Agilent 8453 UV-Visible Spectroscopy System controlled by UV-Visible ChemStation Software. Emission intensities were recorded using a JASCO Spectrofluorometer FP-8600 equipped with a TC-815 Peltier thermostated single cell holder (water-cooled) controlled by Spectra Manager Version 2.10.01. Time resolved emission spectra were recorded using an Edimburg Instruments FS5 Spectrofluorometer, and a 450 nm EPL laser.

2. Synthesis and characterization of alcohols 1

General procedure for the synthesis of the starting ketones[1]

All the ketones were commercially available less for alcohols **1f**-**1h** for which the corresponding ketones were synthesized according to the following procedure:

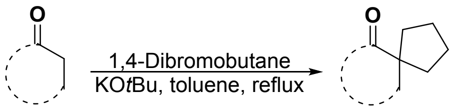

In a flame-dried two-necked round bottom flask equipped with a reflux condenser under agon atmosphere was added the corresponding ketone (1.0 equiv.), KO*t*Bu (2.2 equiv.) and anhydrous toluene (1.0 M). The reaction mixture was stirred for one hour at room temperature. Then, 1,4-dibromobutane was added dropwise and the reaction was stirred under reflux for 5 hours using an oil bath. The mixture was quenched with an aqueous solution of HCl (15% v/v) and extracted three times with EtOAc. The combined organic layers were washed with brine, dried over anhydrous Na_2SO_4, filtered, concentrated under reduced pressure and purified by column chromatography to obtain the pure spiro ketones.

Spiro[4.5]decan-6-one (SI1)[1]

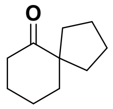

Following the procedure described above with cyclohexanone on 5.0 mmol scale. Purification by column chromatography (silica, gradient from 0% to 10% of EtOAc in cyclohexane) afforded 219 mg (29%) of the title compound **SI1** as a colourless oil. **R_f** = 0.57 (5:1 cyclohexane:EtOAc).

^{1}H NMR (300 MHz, $CDCl_3$): δ 2.40 (t, *J* = 6.6 H, 2H), 2.10 – 2.02 (m, 2H), 1.85 – 1.79 (m, 2H), 1.71 (t, *J* = 2.9 Hz, 4H), 1.63 – 1.54 (m, 4H), 1.43 – 1.34 (m, 2H).

Spiro[4.7]dodecan-6-one (SI2)

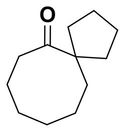

Following the procedure described above with cyclooctanone on 10.0 mmol scale. Purification by column chromatography (silica, gradient from 0% to 10% of EtOAc in cyclohexane) afforded 230 mg (13%) of the title compound **SI2** as a colourless oil. **R_f** = 0.71 (5:1 cyclohexane:EtOAc).

^{1}H NMR (300 MHz, $CDCl_3$): δ 2.50 (t, *J* = 6.3 Hz, 2H), 2.03 – 1.94 (m, 4H), 1.83 – 1.75 (m, 2H), 1.62 – 1.26 (m, 12H).

^{13}C NMR (75 MHz, $CDCl_3$): δ 219.1, 58.8, 37.4, 35.1, 33.9 (2C), 30.2, 25.8, 25.8, 25.3 (2C), 24.4.

HRMS (ESI) m/z: $[M+H]^+$ Calcd for $C_{12}H_{21}O$ 181.1587; Found: 181.1592.

Spiro[4.14]nonadecan-6-one (SI3)

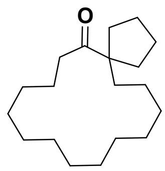

Following the procedure described above with cyclopentadecanone on 10.0 mmol scale. Purification by column chromatography (silica, gradient from 0% to 10% of EtOAc in cyclohexane) afforded 335 mg (12%) of the title compound **SI3** as a colourless oil. **R_f** = 0.65 (5:1 cyclohexane:EtOAc).

^{1}H NMR (300 MHz, $CDCl_3$): δ 2.46 (t, *J* = 7.2 Hz, 2H), 2.14 – 2.00 (m, 2H), 1.64 – 1.59 (m, 5H), 1.50 – 1.24 (m, 23H), 1.14 – 1.06 (m, 2H).

^{13}C NMR (75 MHz, CDCl$_3$): δ 215.1, 60.7, 39.1, 36.6, 34.3 (2C), 27.6, 27.6, 27.2, 27.1, 26.9, 26.2, 26.1, 25.9, 25.8, 24.6 (2C), 24.3, 22.8.
HRMS (ESI) m/z: $[M+H]^+$ Calcd for $C_{19}H_{35}O$ 279.2682; Found: 279.2680.

General procedure for the synthesis of alcohols[2]

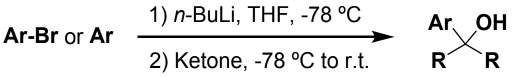

In a flame-dried 100 mL round bottom flask containing a stir bar under argon atmosphere the corresponding aryl bromide or arene (1.0 equiv.) was added and dissolved in anhydrous THF (0.5 M). The solution was cooled to -78 ºC and then *n*-BuLi was added (2.5 M in hexanes, 1.1 equiv.). The solution was stirred at this temperature for 30 min. Then, the corresponding ketone (1.0 equiv.) was added dropwise. The reaction mixture was stirred at -78 ºC for 30 min. After this time, if the ketone was not fully consumed, the reaction is allowed to warm to room temperature. Upon completion, monitored by TLC, the reaction was quenched with a saturated aqueous solution of NH_4Cl and extracted three times with EtOAc. The combined organic layers were washed with brine, dried over anhydrous Na_2SO_4, filtered, concentrated under reduced pressure and purified by silica or basic alumina flash column chromatography (specified in each case) to obtain the pure alcohols.

3-(4-Methoxyphenyl)tetrahydro-2H-pyran-3-ol (1a)[2]

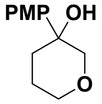

Following the general procedure with dihydro-2*H*-pyran-3(4*H*)-one and 4-bromoanisole on 2.5 mmol scale. Purification by flash column chromatography (silica, gradient from 20% to 60% of EtOAc in cyclohexane) afforded 220 mg (42%) of the title compound **1a** as a white solid. **R$_f$** = 0.14 (5:1 cyclohexane:EtOAc).
^{1}H NMR (300 MHz, C$_6$D$_6$): δ 7.44 (d, *J* = 8.9 Hz, 2H), 6.84 (d, *J* = 8.9 Hz, 2H), 3.69 – 3.57 (m, 2H), 3.39 (d, *J* = 11.4 Hz, 1H), 3.32 (s, 3H), 3.13 – 3.06 (m, 1H), 2.69 (brs, 1H), 1.77 – 1.66 (m, 3H), 1.16 – 1.06 (m, 1H).

tert-Butyl 3-hydroxy-3-(4-methoxyphenyl)piperidine-1-carboxylate (1b)[2]

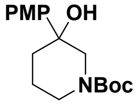

Following the general procedure with tert-butyl 3-oxopiperidine-1-carboxylate and 4-bromoanisole on 5.0 mmol scale. Purification by flash column chromatography (silica, gradient from 10% to 50% of EtOAc in cyclohexane) afforded 300 mg (20%) of the title compound **1b** as a colourless oil. **R$_f$** = 0.17 (5:1 cyclohexane:EtOAc).
^{1}H NMR (300 MHz, C$_6$D$_6$): δ 7.35 (d, *J* = 9.0 Hz, 2H), 6.81 (d, *J* = 9.0 Hz, 2H), 4.29 – 3.99 (m, 2H), 3.34 (s, 3H), 2.99 (d, *J* = 13.7 Hz, 1H), 2.57 (t, *J* = 12.6 Hz, 1H), 1.70 – 1.65 (m, 1H), 1.60 – 1.57 (m, 1H), 1.46 (s, 9H), 1.19 – 1.11 (m, 2H).

3-(4-Methoxyphenyl)-1-tosylpyrrolidin-3-ol (1c)

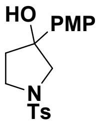

Following the general procedure with 1-tosylpyrrolidin-3-one and 4-bromoanisole on 1.1 mmol scale. Purification by flash column chromatography (silica, gradient from 10% to 50% of EtOAc in cyclohexane) afforded 190 mg (51%) of the title compound **1c** as a brown solid. $\mathbf{R}_{\boldsymbol{f}}$ = 0.20 (2:1 cyclohexane:EtOAc).

^{1}H NMR (300 MHz, CD_2Cl_2): δ 7.73 (d, *J* = 6.6 Hz, 2H), 7.35 (d, *J* = 8.2 Hz, 2H), 7.27 (d, *J* = 7.1 Hz, 2H), 6.85 (d, *J* = 7.3 Hz, 2H), 3.76 (s, 3H), 3.59 – 3.42 (m, 4H), 2.43 (s, 3H), 2.27 – 2.03 (m, 2H).

^{13}C NMR (75 MHz, CD_2Cl_2): δ 159.2, 143.7, 134.3, 134.1, 129.7 (2C), 127.5 (2C), 126.4 (2C), 113.8 (2C), 80.1, 60.7, 55.3, 47.0, 39.4, 21.3.

HRMS (ESI) m/z: $[M+H]^+$ Calcd for $C_{18}H_{22}NO_4S$ 348.1264; Found: 348.1276.

3-(4-Methoxyphenyl)oxetan-3-ol (1d)[3]

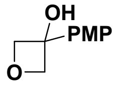

Following the general procedure with oxetan-3-one and 4-bromoanisole on 5.0 mmol scale. Purification by flash column chromatography (silica, gradient from 20% to 60% of EtOAc in cyclohexane) afforded 713 mg (79%) of the title compound **1d** as a white solid. $\mathbf{R}_{\boldsymbol{f}}$ = 0.25 (2:1 cyclohexane:EtOAc).

^{1}H NMR (300 MHz, $CDCl_3$): δ 7.49 (d, *J* = 8.9 Hz, 2H), 6.95 (d, *J* = 8.9 Hz, 2H), 4.93 – 4.90 (m, 4H), 3.83 (s, 3H).

3-(4-Methoxyphenyl)tetrahydrofuran-3-ol (1e)[4]

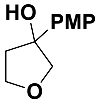

Following the general procedure with dihydrofuran-3(2*H*)-one and 4-bromoanisole on 5.0 mmol scale. Purification by flash column chromatography (silica, gradient from 20% to 60% of EtOAc in cyclohexane) afforded 570 mg (59%) of the title compound **1e** as a white solid. $\mathbf{R}_{\boldsymbol{f}}$ = 0.25 (2:1 cyclohexane:EtOAc).

^{1}H NMR (300 MHz, $CDCl_3$): δ 7.41 (d, *J* = 9.0 Hz, 2H), 6.91 (d, *J* = 8.9 Hz, 2H), 4.25 – 4.13 (m, 2H), 3.98 (dd, *J* = 9.3, 1.4 Hz, 1H), 3.87 (d, *J* = 9.3 Hz, 1H), 3.82 (s, 3H), 2.46 – 2.35 (m, 1H), 2.29 – 2.21 (m, 1H).

6-(4-Methoxyphenyl)spiro[4.5]decan-6-ol (1f)

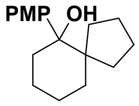

Following the general procedure with compound **SI1** and 4-bromoanisole on 1.0 mmol scale. Purification by flash column chromatography (silica, gradient from 5% to 10% of EtOAc in cyclohexane) afforded 133 mg (51%) of the title compound **1f** as a white solid. $\mathbf{R}_{\boldsymbol{f}}$ = 0.33 (10:1 cyclohexane:EtOAc).

^{1}H NMR (300 MHz, CD_2Cl_2): δ 7.42 (d, *J* = 9.1 Hz, 2H), 6.82 (d, *J* = 9.0 Hz, 2H), 3.77 (s, 3H), 2.32 – 2.21 (m, 1H), 1.90 – 1.68 (m, 3H), 1.61 – 1.55 (m, 4H), 1.44 – 1.28 (m, 5H), 1.17 – 1.08 (m, 1H), 0.99 – 0.92 (m, 2H).

^{13}C NMR (75 MHz, CD_2Cl_2): δ 158.2, 138.6, 128.6 (2C), 112.5 (2C), 77.2, 55.2, 50.1, 36.6, 35.1, 35.1, 32.4, 25.4, 24.6, 22.6, 21.4.

HRMS (ESI) m/z: $[M+H]^+$ Calcd for $C_{17}H_{25}O_2$ 261.1849; Found: 261.1860.

6-(4-Methoxyphenyl)spiro[4.7]dodecan-6-ol (1g)

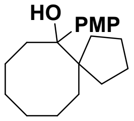

Following the general procedure with compound **SI2** and 4-bromoanisole on 1.3 mmol scale. Purification by flash column chromatography (basic alumina, gradient from 5% to 20% of EtOAc in cyclohexane) afforded 72.3 mg (20%) of the title compound **1g** as a white solid. **R_f** = 0.40 (5:1 cyclohexane:EtOAc).

^{1}H NMR (300 MHz, CD_2Cl_2): δ 7.37 (d, J = 9.0 Hz, 2H), 6.78 (d, J = 9.0 Hz, 2H), 3.76 (s, 3H), 2.48 – 2.33 (m, 1H), 2.16 – 1.89 (m, 3H), 1.75 – 1.56 (m, 8H), 1.49 – 1.21 (m, 7H), 0.92 – 0.79 (m, 1H).

^{13}C NMR (75 MHz, CD_2Cl_2): δ 157.9, 141.8, 128.5 (2C), 112.2 (2C), 79.2, 55.2, 53.3, 39.2, 36.1, 33.5, 33.0, 28.3, 27.3, 25.2, 23.8, 23.7, 21.2.

HRMS (ESI) m/z: $[M+H]^+$ Calcd for $C_{19}H_{29}O_2$ 289.2162; Found: 289.2169.

6-(4-Methoxyphenyl)spiro[4.14]nonadecan-6-ol (1h)

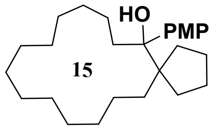

Following the general procedure with compound **SI3** and 4-bromoanisole on 1.2 mmol scale. Purification by flash column chromatography (basic alumina, gradient from 5% to 20% of EtOAc in cyclohexane) afforded 146 mg (32%) of the title compound **1h** as a colourless oil. **R_f** = 0.33 (10:1 cyclohexane:EtOAc).

^{1}H NMR (300 MHz, CD_2Cl_2): δ 7.30 (d, J = 8.9 Hz, 2H), 6.82 (d, J = 9.0 Hz, 2H), 3.78 (s, 3H), 2.20 – 2.10 (m, 1H), 1.89 – 1.79 (m, 1H), 1.67 – 1.57 (m, 3H), 1.48 – 0.94 (m, 29H).

^{13}C NMR (75 MHz, CD_2Cl_2): δ 157.9, 136.2, 129.2 (2C), 112.2 (2C), 81.3, 55.0, 36.2, 36.0, 34.7, 30.7, 28.4, 27.9, 26.9, 26.9, 26.9, 26.8, 26.8, 26.3, 26.2, 25.9, 24.5, 24.4, 24.1, 21.8.

HRMS (ESI) m/z: $[M+H]^+$ Calcd for $C_{26}H_{43}O_2$ 387.3258; Found: 387.3265.

1-(4-methoxyphenyl)cyclohexan-1-ol (1i)[2]

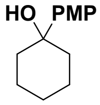

Following the general procedure with cyclohexanone and 4-bromoanisole on 5.0 mmol scale. Purification by flash column chromatography (silica, gradient from 5% to 20% of EtOAc in cyclohexane) afforded 769 mg (75%) of the title compound **1i** as a colourless oil. **R_f** = 0.18 (4:1 cyclohexane:EtOAc).

^{1}H NMR (300 MHz, C_6D_6): δ 7.35 (d, J = 8.9 Hz, 2H), 6.85 (d, J = 9.0 Hz, 2H), 3.36 (s, 3H), 1.82 – 1.57 (m, 7H), 1.51 – 1.44 (m, 2H), 1.19 – 1.05 (m, 1H).

2-(4-Methoxyphenyl)-1-methylcyclohexan-1-ol (1j)[2]

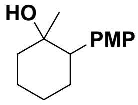

Following a reported procedure[2] on 1.1 mmol scale. Purification by flash column chromatography (silica, gradient from 9% to 20% of EtOAc in cyclohexane) afforded 128 mg (51%) of the title compound **1j** as a colourless oil. **R_f** = 0.48 (4:1 cyclohexane:EtOAc).

^{1}H NMR (300 MHz, C_6D_6): δ 7.17 (d, J = 8.9 Hz, 2H), 6.84 (d, J = 8.8 Hz, 2H), 3.40 (s, 3H), 2.30 – 2.25 (m, 1H), 2.19 – 2.05 (m, 1H), 1.97 – 1.86 (m, 1H), 1.83 – 1.75 (m, 2H), 1.63 – 1.50 (m, 2H), 1.34 – 1.22 (m, 2H), 0.97 (brs, 1H), 0.90 (s, 3H).

3-((4-Methoxyphenyl)ethynyl)tetrahydro-2H-pyran-3-ol (1k)

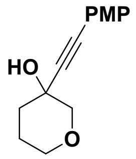

Following the general procedure with dihydro-2*H*-pyran-3(4*H*)-one and 1-ethynyl-4-methoxybenzene on 3.0 mmol scale. Purification by flash column chromatography (silica, gradient from 10% to 40% of EtOAc in cyclohexane) afforded 212 mg (30%) of the title compound **1k** as a yellow oil. $\mathbf{R_f}$ = 0.20 (4:1 cyclohexane:EtOAc).

^{1}H NMR (300 MHz, $CDCl_3$): δ 7.37 (d, *J* = 9.0 Hz, 2H), 6.83 (d, *J* = 9.0 Hz, 2H), 3.81 (s, 3H), 3.77 – 3.74 (m, 1H), 3.71 – 3.69 (m, 2H), 3.64 – 3.56 (m, 1H), 2.05 – 2.01 (m, 2H), 1.97 – 1.84 (m, 1H), 1.72 – 1.63 (m, 1H), 1.51 (brs, 1H).

^{13}C NMR (75 MHz, $CDCl_3$): δ 159.6, 133.2 (2C), 114.4, 113.8 (2C), 88.8, 84.6, 75.6, 67.8, 65.4, 55.1, 36.4, 22.2.

HRMS (ESI) m/z: $[M+H]^+$ Calcd for $C_{14}H_{17}O_3$ 233.1172; Found: 233.1180.

3-(Furan-2-yl)tetrahydro-2H-pyran-3-ol (1l)

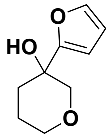

Following the general procedure with dihydro-2*H*-pyran-3(4*H*)-one and furan on 2.0 mmol scale. Purification by flash column chromatography (silica, gradient from 10% to 40% of EtOAc in cyclohexane) afforded 99.0 mg (29%) of the title compound **1l** as a yellowish oil. $\mathbf{R_f}$ = 0.20 (2:1 cyclohexane:EtOAc).

^{1}H NMR (300 MHz, CD_2Cl_2): δ 7.39 – 7.38 (m, 1H), 6.36 – 6.35 (m, 1H), 6.31 – 6.29 (m, 1H), 3.84 – 3.77 (m, 1H), 3.71 – 3.70 (m, 2H), 3.54 – 3.46 (m, 1H), 2.14 – 2.06 (m, 1H), 1.95 – 1.89 (m, 2H), 1.59 – 1.52 (m, 1H).

^{13}C NMR (75 MHz, CD_2Cl_2): δ 157.0, 141.9, 110.1, 105.7, 74.4, 67.9, 67.8, 33.2, 22.0.

HRMS (ESI) m/z: $[M+H]^+$ Calcd for $C_9H_{13}O_3$ 169.0859; Found: 169.0862.

3-(Benzofuran-2-yl)tetrahydro-2H-pyran-3-ol (1m)

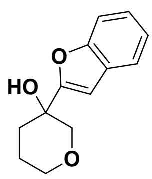

Following the general procedure with dihydro-2*H*-pyran-3(4*H*)-one and benzofuran on 2.0 mmol scale. Purification by flash column chromatography (silica, gradient from 20% to 60% of EtOAc in cyclohexane) afforded 252 mg (58%) of the title compound **1m** as a yellowish oil. $\mathbf{R_f}$ = 0.23 (2:1 cyclohexane:EtOAc).

^{1}H NMR (300 MHz, $CDCl_3$): δ 7.55 (d, *J* = 7.3 Hz, 1H), 7.46 (d, *J* = 7.3 Hz, 1H), 7.30 – 7.21 (m, 2H), 6.73 (d, *J* = 1.1 Hz, 1H), 3.96 – 3.86 (m, 3H), 3.63 – 3.55 (m, 1H), 2.31 – 2.21 (m, 1H), 2.10 – 1.97 (m, 2H), 1.69 – 1.63 (m, 1H).

^{13}C NMR (75 MHz, $CDCl_3$): δ 159.2, 154.6, 128.0, 124.1, 122.8, 121.0, 111.2, 102.7, 74.5, 68.5, 68.0, 33.2, 21.8.

HRMS (ESI) m/z: $[M+H]^+$ Calcd for $C_{13}H_{15}O_3$ 219.1016; Found: 219.1027.

3-(Phenanthren-9-yl)tetrahydro-2H-pyran-3-ol (1n)

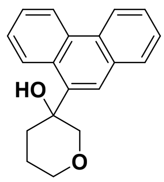

Following the general procedure with dihydro-2*H*-pyran-3(4*H*)-one and 9-bromophenanthrene on 5.0 mmol scale. Purification by flash column

chromatography (silica, gradient from 5% to 15% of EtOAc in cyclohexane) afforded 416 mg (30%) of the title compound **1n** as a white solid. **R_f** = 0.25 (2:1 cyclohexane:EtOAc).

^{1}H NMR (300 MHz, $CDCl_3$): δ 8.98 (d, *J* = 7.8 Hz, 1H), 8.76 (d, *J* = 8.4 Hz, 1H), 8.66 (d, *J* = 8.2 Hz, 1H), 7.87 (d, *J* = 8.1 Hz, 1H), 7.80 (s, 1H), 7.68 – 7.57 (m, 4H), 4.20 (d, *J* = 11.4 Hz, 1H), 4.07 (d, *J* = 10.7 Hz, 1H), 4.01 – 3.97 (m, 1H), 3.66 (td, *J* = 11.1, 3.2 Hz, 1H), 3.02 (s, 1H), 2.47 – 2.31 (m, 2H), 2.15 – 2.04 (m, 1H), 1.69 – 1.58 (m, 1H).

^{13}C NMR (75 MHz, $CDCl_3$): δ 137.0, 131.6, 130.9, 130.3, 130.1, 129.0, 127.9, 127.0, 126.7, 126.1, 126.0, 125.0, 123.2, 122.3, 76.3, 72.0, 68.5, 35.4, 22.7.

HRMS (ESI) m/z: $[M+H]^+$ Calcd for $C_{19}H_{19}O_2$ 279.1380; Found: 279.1392.

3. Synthesis and characterization of α,β-unsaturated alkenes 2

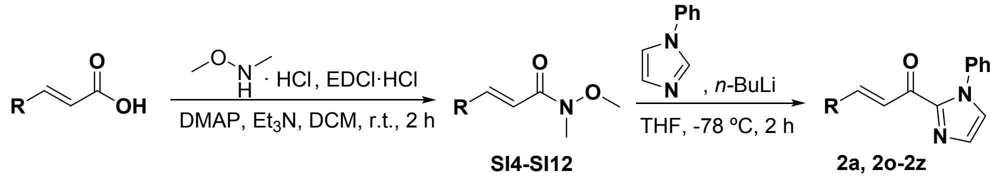

α,β-Unsaturated 2-acyl imidazoles were obtained in a two-step synthesis from the commercially available carboxylic acids.

General procedure for the synthesis of Weinreb amides: In a 100 mL round bottom flask with a stir bar, the corresponding acid (1.0 equiv.), *N*,*O*-dimethylhydroxylamine hydrochloride (1.5 equiv.), DMAP (0.2 equiv.) and EDCI hydrochloride (1.5 equiv.) were dissolved in DCM (0.5 M) under argon atmosphere. Then, Et_3N (1.5 equiv.) was added at 0 ºC. After stirring 10 minutes at this temperature, the reaction was allowed to stir at room temperature for 2 hours. Upon completion (monitored by TLC), the reaction was quenched with 10 mL of HCl 1M an extracted three times with 40 mL of EtOAc. The combined organic layers were washed with an aqueous saturated solution of $NaHCO_3$, brine and dried over anhydrous $MgSO_4$. The solution was filtered and concentrated under reduced pressure to obtain the pure Weinreb amides.

(*E*)-*N*-methoxy-*N*-methylcinnamamide (SI4)[5]

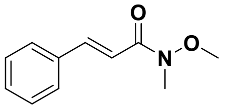

Following the general procedure with *E*-cinnamic acid on 20 mmol scale afforded 3.70 g (97%) of the title compound **SI4** as a yellowish solid. $\mathbf{R_f}$ = 0.37 (2:1 cyclohexane:EtOAc).

^{1}H NMR (300 MHz, $CDCl_3$): δ 7.74 (d, *J* = 15.8 Hz, 1H), 7.63 – 7.52 (m, 2H), 7.42 – 7.37 (m, 3H), 7.04 (d, *J* = 15.8 Hz, 1H), 3.77 (s, 3H), 3.31 (s, 3H).

(*E*)-3-(4-chlorophenyl)-*N*-methoxy-*N*-methylacrylamide (SI5)[6]

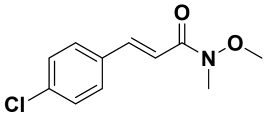

Following the general procedure with (*E*)-3-(4-chlorophenyl)acrylic acid on 3.0 mmol scale afforded 580 mg (86%) of the title compound **SI5** as a white solid. $\mathbf{R_f}$ = 0.33 (2:1 cyclohexane:EtOAc).

^{1}H NMR (300 MHz, $CDCl_3$): δ 7.68 (d, *J* = 15.7 Hz, 1H), 7.50 (d, *J* = 8.9 Hz, 2H), 7.35 (d, *J* = 8.6 Hz, 2H), 7.00 (d, *J* = 15.7 Hz, 1H), 3.77 (s, 3H), 3.32 (s, 3H).

(*E*)-3-(4-fluorophenyl)-*N*-methoxy-*N*-methylacrylamide (SI6)

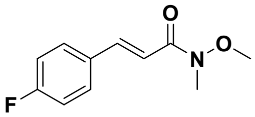

Following the general procedure with (*E*)-3-(4-fluorophenyl)acrylic acid on 3.0 mmol scale afforded 560 mg (89%) of the title compound **SI6** as a white solid. $\mathbf{R_f}$ = 0.38 (2:1 cyclohexane:EtOAc).

^{1}H NMR (300 MHz, $CDCl_3$): δ 7.70 (d, *J* = 15.7 Hz, 1H), 7.61 – 7.50 (m, 2H), 7.10 – 7.04 (m, 2H), 6.96 (d, *J* = 15.9 Hz, 1H), 3.77 (s, 3H), 3.31 (s, 3H).

^{13}C NMR (75 MHz, $CDCl_3$): δ 166.8, 163.6 (d, *J* = 248.8 Hz) 142.1, 131.4 (d, *J* = 3.4 Hz), 129.9 (d, *J* = 8.4 Hz, 2C), 115.9 (d, *J* = 22.1 Hz, 2C), 115.5 (d, *J* = 2.4 Hz) 61.8, 32.5.

^{19}F NMR (471 MHz, $CDCl_3$): δ -110.5.

HRMS (ESI) m/z: $[M+H]^+$ Calcd for $C_{11}H_{13}FNO_2$ 210.0925; Found: 210.0936.

(*E*)-3-(3-bromophenyl)-*N*-methoxy-*N*-methylacrylamide (SI7)

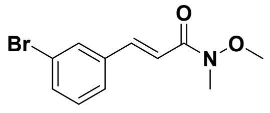

Following the general procedure with (*E*)-3-(3-bromophenyl)acrylic acid on 5.0 mmol scale afforded 1.32 g (98%) of the title compound **SI7** as a brown oil. **R*f*** = 0.30 (2:1 cyclohexane:EtOAc).

^{1}H NMR (300 MHz, CDCl$_3$): δ 7.72 (s, 1H), 7.64 (d, *J* = 16.2 Hz, 1H), 7.48 (t, *J* = 7.0 Hz, 2H), 7.28 – 7.23 (m, 1H), 7.02 (d, *J* = 15.8 Hz, 1H), 3.78 (s, 3H), 3.32 (s, 3H).

^{13}C NMR (75 MHz, CDCl$_3$): δ 166.1, 141.5, 137.1, 132.4, 130.2, 130.1, 126.7, 122.7, 117.1, 61.8, 32.3.

HRMS (ESI) m/z: $[M+H]^+$ Calcd for $C_{11}H_{13}BrNO_2$ 270.0124; Found: 270.0129.

(*E*)-3-(4-cyanophenyl)-*N*-methoxy-*N*-methylacrylamide (SI8)

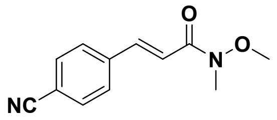

Following the general procedure with (*E*)-3-(4-cyanophenyl)acrylic acid on 1.3 mmol scale afforded 170 mg (62%) of the title compound **SI8** as a white solid. **R*f*** = 0.13 (2:1 cyclohexane:EtOAc).

^{1}H NMR (300 MHz, CDCl$_3$): δ 7.77 – 7.60 (m, 5H), 7.12 (d, *J* = 15.8 Hz, 1H), 3.78 (s, 3H), 3.33 (s, 3H).

^{13}C NMR (75 MHz, CDCl$_3$): δ 165.9, 141.0, 139.5, 132.5 (2C), 128.4 (2C), 119.3, 118.5, 112.9, 62.0, 32.5.

HRMS (ESI) m/z: $[M+H]^+$ Calcd for $C_{12}H_{13}N_2O_2$ 217.0972; Found: 217.0978.

(*E*)-*N*-methoxy-*N*-methyl-3-(4-(trifluoromethyl)phenyl)acrylamide (SI9)

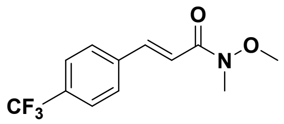

Following the general procedure with (*E*)-3-(4-(trifluoromethyl)phenyl)acrylic acid on 3.0 mmol scale afforded 720 mg (93%) of the title compound **SI9** as a white solid. **R*f*** = 0.25 (2:1 cyclohexane:EtOAc).

^{1}H NMR (300 MHz, CDCl$_3$): δ 7.74 (d, *J* = 15.9 Hz, 1H), 7.69 – 7.62 (m, 4H), 7.10 (d, *J* = 15.9 Hz, 1H), 3.78 (s, 3H), 3.33 (s, 3H).

^{13}C NMR (75 MHz, CDCl$_3$): δ 166.2, 141.6, 138.6, 131.3 (q, *J* = 32.5 Hz), 128.1 (2C), 125.7 (q, *J* = 3.5 Hz, 2C), 124.0 (q, *J* = 273.2 Hz), 118.3, 62.0, 32.5.

^{19}F NMR (471 MHz, CDCl$_3$): δ -62.8.

HRMS (ESI) m/z: $[M+H]^+$ Calcd for $C_{12}H_{13}F_3NO_2$ 260.0893; Found: 260.0898.

(*E*)-*N*-methoxy-3-(3-methoxyphenyl)-*N*-methylacrylamide (SI10)[6]

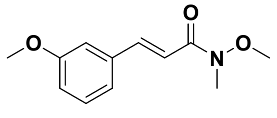

Following the general procedure with (*E*)-3-(3-methoxyphenyl)acrylic acid on 3.0 mmol scale afforded 660 mg (99%) of the title compound **SI10** as a colourless oil. **R*f*** = 0.30 (2:1 cyclohexane:EtOAc).

^{1}H NMR (300 MHz, CDCl$_3$): δ 7.63 (d, *J* = 15.7 Hz, 1H), 7.26 – 7.19 (m, 1H), 7.10 (d, *J* = 7.6 Hz, 1H), 7.03 – 7.01 (m, 1H), 6.94 (d, *J* = 15.9 Hz, 1H), 6.87 – 6.93 (m, 1H), 3.77 (s, 3H), 3.70 (s, 3H), 3.24 (s, 3H).

(*E*)-*N*-methoxy-*N*-methyl-3-(*p*-tolyl)acrylamide (SI11)[6]

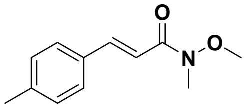

Following the general procedure with (*E*)-3-(*p*-tolyl)acrylic acid on 3.0 mmol scale afforded 540 mg (86%) of the title compound **SI11** as a white solid. **R*f*** = 0.30 (2:1 cyclohexane:EtOAc).

^{1}H NMR (300 MHz, $CDCl_3$): δ 7.71 (d, *J* = 15.8 Hz, 1H), 7.47 (d, *J* = 8.4 Hz, 2H), 7.19 (d, *J* = 7.5 Hz, 2H), 6.99 (d, *J* = 15.8 Hz, 1H), 3.77 (s, 3H), 3.31 (s, 3H), 2.38 (s, 3H).

(*E*)-*N*-methoxy-*N*-methyl-3-(*o*-tolyl)acrylamide (SI12)[6]

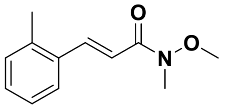

Following the general procedure with (*E*)-3-(*o*-tolyl)acrylic acid on 3.0 mmol scale afforded 612 mg (99%) of the title compound **SI12** as a colourless oil. **R*f*** = 0.33 (2:1 cyclohexane:EtOAc).

^{1}H NMR (300 MHz, $CDCl_3$): δ 8.02 (d, *J* = 15.7 Hz, 1H), 7.60 (d, *J* = 8.3 Hz, 1H), 7.32 – 7.16 (m, 3H), 6.95 (d, *J* = 15.6 Hz, 1H), 3.76 (s, 3H), 3.32 (s, 3H), 2.45 (s, 3H).

(*E*)-*N*-methoxy-*N*-methylbut-2-enamide (SI13)[7]

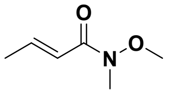

Following the general procedure with (*E*)-but-2-enoic acid on 10 mmol scale afforded 1.22 g (94%) of the title compound **SI13** as a colourless oil. **R*f*** = 0.24 (2:1 cyclohexane:EtOAc).

^{1}H NMR (300 MHz, $CDCl_3$): δ 7.07 – 6.89 (m, 1H), 6.41 (dd, *J* = 15.3, 1.7 Hz, 1H), 3.70 (s, 3H), 3.23 (s, 3H), 1.90 (dd, *J* = 6.9, 1.7 Hz, 3H).

General procedure for the synthesis of α,β-unsaturated 2-acyl imidazoles: In a 100 mL round bottom flask with an stir bar, *N*-phenylimidazole (1.2 equiv.) was dissolved in dry THF (0.5 M) under argon atmosphere and cooled to -78 ºC. Then, *n*-BuLi (2.5 M in hexanes, 1.2 equiv.) was added at this temperature and the solution was stirred for 2 hours. After this time, the Weinreb amide (1.0 equiv.) was added and the reaction was stirred at -78 ºC for 2 hours. Upon completion (monitored by TLC), the reaction was quenched with water (20 mL) and extracted three times with EtOAc (60 mL). The combined organic layers were washed with brine, dried over anhydrous $MgSO_4$, evaporated under reduced pressure and purified by flash column chromatography to obtain the pure products.

(*E*)-3-phenyl-1-(1-phenyl-1H-imidazol-2-yl)prop-2-en-1-one (2a)[8]

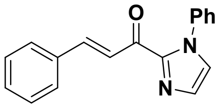

Following the general procedure with compound **SI4** on 5.0 mmol scale. Purification by flash column chromatography (silica, 2:1 cyclohexane:EtOAc) afforded 904 mg (66%) of the title compound **2a** as a yellowish solid. **R*f*** = 0.25 (2:1 cyclohexane:EtOAc).

^{1}H NMR (300 MHz, $CDCl_3$): δ 8.10 (d, *J* = 15.9 Hz, 1H), 7.76 (d, *J* = 16.9 Hz, 1H), 7.69 – 7.66 (m, 2H), 7.51 – 7.48 (m, 3H), 7.41 – 7.33 (m, 6H), 7.25 – 7.24 (m, 1H).

(*E*)-3-(4-chlorophenyl)-1-(1-phenyl-1H-imidazol-2-yl)prop-2-en-1-one (2o)[9]

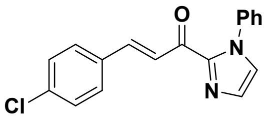

Following the general procedure with compound **SI5** on 2.0 mmol scale. Purification by flash column chromatography (silica, 2:1 cyclohexane:EtOAc) afforded 150 mg (49%) of the title compound **2o** as a white solid. **R_f** = 0.50 (2:1 cyclohexane:EtOAc).

^{1}H NMR (300 MHz, $CDCl_3$): δ 8.07 (d, *J* = 16.0 Hz, 1H), 7.69 (d, *J* = 15.9 Hz, 1H), 7.60 (d, *J* = 8.6 Hz, 2H), 7.51 – 7.48 (m, 3H), 7.38 – 7.32 (m, 5H), 7.26 – 7.25 (m, 1H).

(*E*)-3-(4-fluorophenyl)-1-(1-phenyl-1H-imidazol-2-yl)prop-2-en-1-one (2p)[10]

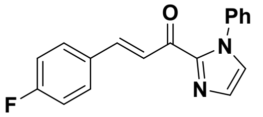

Following the general procedure with compound **SI6** on 2.0 mmol scale. Purification by flash column chromatography (silica, gradient from 10% to 60% of EtOAc in cyclohexane) afforded 150 mg (49%) of the title compound **2p** as a white solid. **R_f** = 0.55 (2:1 cyclohexane:EtOAc).

^{1}H NMR (300 MHz, $CDCl_3$): δ 8.02 (d, *J* = 15.9 Hz, 1H), 7.76 – 7.60 (m, 3H), 7.52 – 7.47 (m, 3H), 7.37 – 7.32 (m, 3H), 7.25 – 7.24 (m, 1H), 7.08 (t, *J* = 8.6 Hz, 2H).

(*E*)-3-(3-bromophenyl)-1-(1-phenyl-1H-imidazol-2-yl)prop-2-en-1-one (2q)[8]

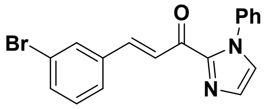

Following the general procedure with compound **SI7** on 0.6 mmol scale. Purification by flash column chromatography (silica, gradient from 5% to 40% of EtOAc in cyclohexane) afforded 58.0 mg (28%) of the title compound **2q** as a yellowish solid. **R_f** = 0.56 (2:1 cyclohexane:EtOAc).

^{1}H NMR (300 MHz, $CDCl_3$): δ 8.07 (d, *J* = 15.9 Hz, 1H), 7.82 (s, 1H), 7.64 (d, *J* = 15.6 Hz, 1H), 7.57 – 7.49 (m, 5H), 7.38 – 7.29 (m, 5H).

(*E*)-4-(3-oxo-3-(1-phenyl-1H-imidazol-2-yl)prop-1-en-1-yl)benzonitrile (2r)

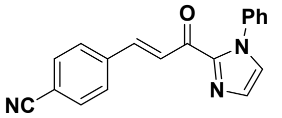

Following the general procedure with compound **SI8** on 0.7 mmol scale. Purification by flash column chromatography (silica, 1:1 cyclohexane:EtOAc) afforded 94.0 mg (45%) of the title compound **2r** as a white solid. **R_f** = 0.30 (2:1 cyclohexane:EtOAc).

^{1}H NMR (300 MHz, $CDCl_3$): δ 8.11 (d, *J* = 16.1 Hz, 1H), 7.69 – 7.59 (m, 5H), 7.45 – 7.43 (m, 3H), 7.32 – 7.25 (m, 3H), 7.20 – 7.19 (m, 1H).

^{13}C NMR (75 MHz, $CDCl_3$): δ 178.3, 143.6, 140.9, 139.1, 138.2, 132.5 (2C), 130.1, 129.0 (2C), 128.9 (2C), 128.9, 127.7, 125.9, 125.8 (2C), 118.4, 113.3.

HRMS (ESI) m/z: $[M+H]^+$ Calcd for $C_{19}H_{14}N_3O$ 300.1131; Found: 300.1140.

(*E*)-1-(1-phenyl-1H-imidazol-2-yl)-3-(4-(trifluoromethyl)phenyl)prop-2-en-1-one (2s)[8]

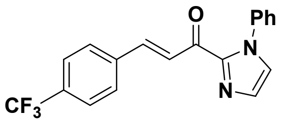

Following the general procedure with compound **SI9** on 1.0 mmol scale. Purification by flash column chromatography (silica, 2:1

cyclohexane:EtOAc) afforded 130 mg (39%) of the title compound **2s** as a white solid. $\mathbf{R_f}$ = 0.50 (2:1 cyclohexane:EtOAc).

^{1}H NMR (300 MHz, $CDCl_3$): δ 8.15 (d, *J* = 16.0 Hz, 1H), 7.80 – 7.72 (m, 2H), 7.72 – 7.60 (m, 2H), 7.52 – 7.48 (m, 3H), 7.41 – 7.30 (m, 3H), 7.30 – 7.17 (m, 2H).

(*E*)-3-(3-methoxyphenyl)-1-(1-phenyl-1H-imidazol-2-yl)prop-2-en-1-one (2u)[9]

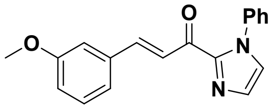

Following the general procedure with compound **SI10** on 2.0 mmol scale. Purification by flash column chromatography (silica, gradient from 5% to 40% of EtOAc in cyclohexane) afforded 270 mg (37%) of the title compound **2u** as a yellow solid. $\mathbf{R_f}$ = 0.44 (2:1 cyclohexane:EtOAc).

^{1}H NMR (300 MHz, $CDCl_3$): δ 8.09 (d, *J* = 15.9 Hz, 1H), 7.73 (d, *J* = 15.9 Hz, 1H), 7.55 – 7.44 (m, 3H), 7.41 – 7.18 (m, 7H), 7.00 – 6.91 (m, 1H), 3.86 (s, 3H).

(*E*)-1-(1-phenyl-1H-imidazol-2-yl)-3-(*p*-tolyl)prop-2-en-1-one (2v)

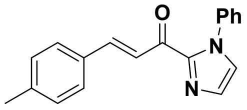

Following the general procedure with compound **SI11** on 2.0 mmol scale. Purification by flash column chromatography (silica, 10% to 50% of EtOAc in cyclohexane) afforded 330 mg (57%) of the title compound **2v** as a yellow solid. $\mathbf{R_f}$ = 0.55 (2:1 cyclohexane:EtOAc).

^{1}H NMR (300 MHz, $CDCl_3$): δ 8.06 (d, *J* = 15.8 Hz, 1H), 7.74 (d, *J* = 15.8 Hz, 1H), 7.57 (d, *J* = 8.1 Hz, 2H), 7.51 – 7.48 (m, 3H), 7.37 – 7.32 (m, 3H), 7.24 – 7.19 (m, 3H), 2.38 (s, 3H).

^{13}C NMR (75 MHz, $CDCl_3$): δ 179.1, 144.0, 141.0, 138.5, 132.1, 129.7, 129.6, 129.6 (2C), 128.9 (2C), 128.8 (2C), 128.7, 127.1, 125.9 (2C), 121.5, 21.5.

HRMS (ESI) m/z: $[M+H]^+$ Calcd for $C_{19}H_{17}N_2O$ 289.1335; Found: 289.1339.

(*E*)-1-(1-phenyl-1H-imidazol-2-yl)-3-(*o*-tolyl)prop-2-en-1-one (2w)

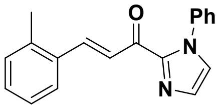

Following the general procedure with compound **SI12** on 2.0 mmol scale. Purification by flash column chromatography (silica, 2:1 cyclohexane:EtOAc) afforded 370 mg (63%) of the title compound **2w** as an orange solid. $\mathbf{R_f}$ = 0.60 (2:1 cyclohexane:EtOAc).

^{1}H NMR (300 MHz, $CDCl_3$): δ 7.99 (d, *J* = 4.6 Hz, 2H), 7.76 (d, *J* = 7.4 Hz, 1H), 7.44 – 7.42 (m, 3H), 7.30 – 7.27 (m, 3H), 7.21 – 7.11 (m, 4H), 2.36 (s, 3H).

^{13}C NMR (75 MHz, $CDCl_3$): δ 179.1, 143.9, 141.3, 138.6, 138.5, 133.6, 130.8, 130.3, 129.7, 128.9 (2C), 128.8, 127.3, 126.8, 126.3, 126.0 (2C), 123.3, 19.8.

HRMS (ESI) m/z: $[M+H]^+$ Calcd for $C_{19}H_{17}N_2O$ 289.1335; Found: 289.1341.

Preparation of (*E*)-3-phenyl-1-(1-phenyl-1H-imidazol-2-yl)but-2-en-1-one (**2x**):

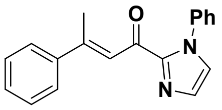

In a 100 mL round bottom flask with a stir bar, TMEDA (1.2 equiv.) was dissolved in dry THF (2 mL) under argon atmosphere and cooled to -78 ºC. Then, *n*-BuLi (2.5 M in hexanes, 1.2 equiv.) was added at this temperature. A solution of *N*-phenylimidazole (1.0 equiv.) in THF (2 mL) was added and the reaction was stirred at -78 ºC for 1 hour. After this time, a solution of methyl (*E*)-3-phenylbut-2-enoate (2.5 mmol, 1.0 equiv.) in THF (2 mL) was added and the mixture was stirred for 1 hour. The reaction was warmed to room temperature and stirred overnight. Upon completion (monitored by TLC), the reaction was quenched with an aqueous saturated solution of $NaHCO_3$ (20 mL) and extracted three times with EtOAc (60 mL). The combined organic layers were washed with brine, dried over anhydrous $MgSO_4$, evaporated under reduced pressure and purified by flash column chromatography (silica, gradient from 5% to 40% of EtOAc in cyclohexane) to afford 202 mg (29%) of the title compound **2x** as a white solid. $\mathbf{R_f}$ = 0.31 (4:1 cyclohexane:EtOAc).
^{1}H NMR (300 MHz, $CDCl_3$): δ 7.81 (q, *J* = 1.2 Hz, 1H), 7.65 – 7.61 (m, 2H), 7.52 – 7.47 (m, 3H), 7.41 – 7.33 (m, 5H), 7.17 (d, *J* = 1.1 Hz, 1H), 7.17 (d, *J* = 0.9 Hz, 1H), 2.57 (d, *J* = 1.3 Hz, 3H).
^{13}C NMR (75 MHz, $CDCl_3$): δ 180.4, 156.4, 145.0, 142.6, 138.8, 129.2, 129.2, 128.9 (2C), 128.5, 128.4 (2C), 126.8, 126.6 (2C), 125.8 (2C), 121.7, 18.6.
HRMS (ESI) m/z: $[M+H]^+$ Calcd for $C_{19}H_{17}N_2O$ 289.1335; Found: 289.1345.

(*E*)-1-(1-phenyl-1H-imidazol-2-yl)but-2-en-1-one (2y)[10]

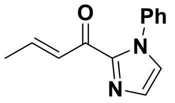

Following the general procedure with compound **SI13** on 3.0 mmol scale. Purification by flash column chromatography (silica, gradient from 20% to 60% of EtOAc in cyclohexane) afforded 535 mg (84%) of the title compound **2y** as a yellow solid. $\mathbf{R_f}$ = 0.29 (2:1 cyclohexane:EtOAc).
^{1}H NMR (300 MHz, $CDCl_3$): δ 7.48 – 7.39 (m, 4H), 7.32 – 7.27 (m, 3H), 7.22 – 7.20 (m, 1H), 7.13 – 7.03 (m, 1H), 1.98 (dd, *J* = 6.9, 1.6 Hz, 3H).

Preparation of (*E*)-4-methyl-1-(1-phenyl-1H-imidazol-2-yl)pent-2-en-1-one (**2z**)[11]

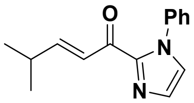

Following a reported procedure[11] on 1.5 mmol scale. Purification by flash column chromatography (silica, gradient from 10% to 25% of EtOAc in cyclohexane) afforded 84.0 mg (24%) of the title compound **2z** as a white solid. $\mathbf{R_f}$ = 0.35 (2:1 cyclohexane:EtOAc).
^{1}H NMR (300 MHz, $CDCl_3$): δ 7.48- 7.45 (m, 3H), 7.37 – 7.26 (m, 5H), 7.03 (dd, *J* = 15.7, 6.7 Hz, 1H), 2.59 – 2.50 (m, 1H), 1.10 (d, *J* = 6.8 Hz, 6H).

4. Synthesis and characterization of products 3

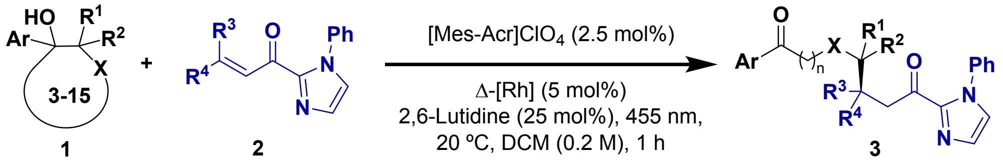

General procedure for the conjugate addition of remote alkyl radicals to alkenes. A vial equipped with a magnetic stir bar was charged with alcohol **1** (0.05 mmol, 1.0 equiv.), alkene **2** (0.05 mmol, 1.0 equiv.), Δ-Rh (1.8 mg, 2.5 μmol, 0.05 equiv.) and 250 μL of a stock solution of [Mes-Acr]ClO_4 in DCM (4.85 μM) followed by 2,6-lutidine (1.4 mg, 0.0125 mmol, 0.25 equiv.). Degasification of the reaction mixture was performed via freeze-pump-thaw cycling (3 x 10 min under vacuum). Then, the reaction mixture was irradiated and stirred in the photoreactor setup at 455 nm for 1 h. The reaction mixture was concentrated under reduced pressure and purified by flash column chromatography to provide the products.

(*S*)-4-(4-(4-Methoxyphenyl)-4-oxobutoxy)-3-phenyl-1-(1-phenyl-1H-imidazol-2-yl)butan-1-one (3a)

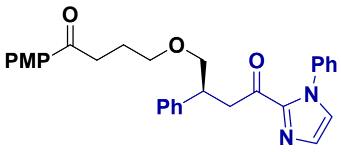

Following the general procedure with compound **1a** and **2a**. Purification by flash column chromatography (silica, gradient from 10% to 40% of EtOAc in cyclohexane) afforded 19.0 mg (79%) of the title compound **3a** as a colourless oil. **R*f*** = 0.13 (2:1 cyclohexane:EtOAc). The enantiomeric excess was determined by SFC on a Daicel Chiralpak IB-3 column: CO_2/MeOH 95:5, flow rate 2.0 mL/min, τ_{major} = 11.7 min, τ_{minor} = 12.9 min, *ee* = 94%. $[\alpha_D^{20}]$ = +9.19 (*c* = 0.77, $CHCl_3$)

^{1}H NMR (300 MHz, $CDCl_3$): δ 7.81 (d, *J* = 8.9 Hz, 2H), 7.32 – 7.28 (m, 3H), 7.20 – 7.16 (m, 5H), 7.13 – 7.08 (m, 1H), 7.04 – 7.01 (m, 3H), 6.83 (d, *J* = 8.9 Hz, 2H), 3.79 (s, 3H), 3.64 – 3.40 (m, 5H), 3.36 (d, *J* = 5.1 Hz, 2H), 2.79 (td, *J* = 7.0, 1.6 Hz, 2H), 1.84 – 1.75 (m, 2H).

^{13}C NMR (75 MHz, $CDCl_3$): δ 198.7, 189.7, 163.3, 143.0, 141.9, 138.2, 130.3 (2C), 130.1, 129.1, 128.9 (2C), 128.6, 128.3 (2C), 128.0 (2C), 126.8, 126.5, 125.7 (2C), 113.6 (2C), 75.0, 70.0, 55.4, 42.3, 41.6, 34.7, 24.3.

HRMS (ESI) m/z: $[M+H]^+$ Calcd for $C_{30}H_{31}N_2O_4$ 483.2278; Found: 483.2267.

Tert-butyl (*S*)-(4-(4-methoxyphenyl)-4-oxobutyl)(4-oxo-2-phenyl-4-(1-phenyl-1H-imidazol-2-yl)butyl)carbamate (3b)

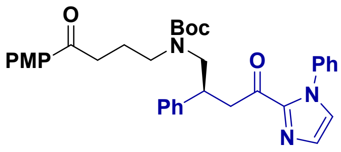

Following the general procedure with compound **1b** and **2a**. Purification by flash column chromatography (silica, gradient from 15% to 60% of EtOAc in cyclohexane) afforded 20.8 mg (72%) of the title compound **3b** as a yellowish oil. **R*f*** = 0.14 (2:1 cyclohexane:EtOAc). The enantiomeric excess was determined by SFC on a Daicel Chiralpak IB-3 column: CO_2/MeOH 90:10, flow rate 2.0 mL/min, τ_{major} = 3.39 min, τ_{minor} = 4.06 min, *ee* = 88%. $[\alpha_D^{20}]$ = -15.9 (*c* = 0.56, $CHCl_3$)

^{1}H NMR (300 MHz, $CDCl_3$): δ 7.82 (d, J = 8.9 Hz, 2H), 7.31 – 7.29 (m, 3H), 7.17 – 7.02 (m, 7H), 6.99 – 6.98 (m, 2H), 6.84 (d, J = 8.9 Hz, 2H), 3.79 (s, 3H), 3.69 – 3.35 (m, 5H), 3.14 – 3.02 (m, 2H), 2.75 (brs, 2H), 1.78 (brs, 2H), 1.27 (s, 9H).

^{13}C NMR (75 MHz, $CDCl_3$): δ 198.1, 189.4, 163.3, 155.6, 143.1, 141.9, 138.2, 130.2 (2C), 130.0, 129.5 (2C), 128.8 (2C), 128.5, 128.4, 128.1 (2C), 126.9, 126.6, 125.7 (2C), 113.6 (2C), 79.5, 77.2, 55.4, 52.7, 46.4, 42.4, 40.4, 35.3, 28.2 (3C).

HRMS (ESI) m/z: $[M+H]^+$ Calcd for $C_{35}H_{40}N_3O_5$ 582.2962; Found: 582.2976.

(*S*)-*N*-(3-(4-Methoxyphenyl)-3-oxopropyl)-4-methyl-N-(4-oxo-2-phenyl-4-(1-phenyl-1H-imidazol-2-yl)butyl)benzenesulfonamide (3c)

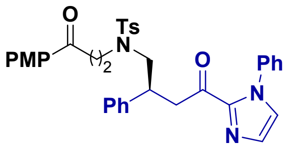

Following the general procedure with compound **1c** and **2a**. Purification by flash column chromatography (silica, gradient from 10% to 40% of EtOAc in cyclohexane) afforded 14.9 mg (48%) of the title compound **3c** as a yellowish oil. **R*f*** = 0.13 (2:1 cyclohexane:EtOAc). The enantiomeric excess was determined by SFC on a Daicel Chiralpak IB-3 column: CO_2/MeOH 90:10, flow rate 2.0 mL/min, τ_{major} = 8.87 min, τ_{minor} = 10.6 min, *ee* = 94%.

$[\alpha_D^{20}]$ = -21.0 (c = 0.61, $CHCl_3$)

^{1}H NMR (300 MHz, $CDCl_3$): δ 7.84 (d, J = 8.9 Hz, 2H), 7.60 (d, J = 8.3 Hz, 2H), 7.42 – 7.33 (m, 4H), 7.24 – 7.16 (m, 7H), 7.11 – 7.04 (m, 3H), 6.90 (d, J = 8.9 Hz, 2H), 3.88 (s, 3H), 3.75 – 3.51 (m, 5H), 3.25 – 3.11 (m, 4H), 2.39 (s, 3H).

^{13}C NMR (75 MHz, $CDCl_3$): δ 197.0, 189.5, 163.6, 143.3, 142.9, 141.2, 138.2, 135.6, 130.3 (2C), 129.7 (2C), 129.6, 129.5, 128.9 (2C), 128.7 (2C), 128.5, 128.2 (2C), 127.4 (2C), 127.0, 126.8, 125.7 (2C), 113.6 (2C), 55.8, 55.5, 45.5, 42.9, 40.81, 38.4, 21.5.

HRMS (ESI) m/z: $[M+H]^+$ Calcd for $C_{36}H_{36}N_3O_5S$ 622.2370; Found: 622.2369.

(*S*)-4-(2-(4-Methoxyphenyl)-2-oxoethoxy)-3-phenyl-1-(1-phenyl-1H-imidazol-2-yl)butan-1-one (3d)

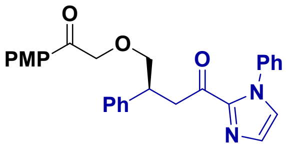

Following the general procedure with compound **1d** and **2a**. Purification by flash column chromatography (silica, gradient from 5% to 50% of EtOAc in cyclohexane) afforded 10.5 mg (46%) of the title compound **3d** as a yellowish oil. **R*f*** = 0.13 (2:1 cyclohexane:EtOAc). The enantiomeric excess was determined by SFC on a Daicel Chiralpak IA column: CO_2/MeOH 80:20, flow rate 3.0 mL/min, τ_{major} = 7.41 min, τ_{minor} = 8.51 min, *ee* = 86%.

$[\alpha_D^{20}]$ = -2.93 (c = 0.34, $CHCl_3$)

^{1}H NMR (300 MHz, $CDCl_3$): δ 7.77 (d, J = 8.9 Hz, 2H), 7.31 – 7.28 (m, 3H), 7.20 – 7.14 (m, 6H), 7.05 – 7.02 (m, 3H), 6.79 (d, J = 9.0 Hz, 2H), 4.49 (s, 2H), 3.78 (s, 3H), 3.70 – 3.63 (m, 4H), 3.48 – 3.40 (m, 1H).

^{13}C NMR (75 MHz, $CDCl_3$): δ 194.9, 189.5, 163.6, 143.0, 141.5, 138.2, 130.4 (2C), 129.2, 128.8 (2C), 128.6, 128.4 (2C), 128.0 (2C), 128.0, 126.8, 126.7, 125.7 (2C), 113.7 (2C), 75.8, 74.1, 55.4, 42.2, 41.4.

HRMS (ESI) m/z: $[M+H]^+$ Calcd for $C_{28}H_{27}N_2O_4$ 455.1965; Found: 455.1911.

(*S*)-4-(3-(4-Methoxyphenyl)-3-oxopropoxy)-3-phenyl-1-(1-phenyl-1H-imidazol-2-yl)butan-1-one (3e)

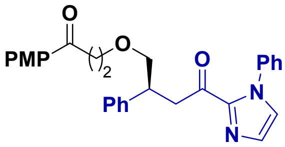

Following the general procedure with compound **1e** and **2a**. Purification by flash column chromatography (silica, gradient from 10% to 50% of EtOAc in cyclohexane) afforded 20.4 mg (87%) of the title compound **3e** as a yellow oil. $\mathbf{R_f}$ = 0.12 (2:1 cyclohexane:EtOAc). The enantiomeric excess was determined by SFC on a Daicel Chiralpak IA column: CO_2/MeOH 80:20, flow rate 2.0 mL/min, τ_{major} = 13.8 min, τ_{minor} = 13.1 min, *ee* = 94%.

$[\alpha_D^{20}]$ = +7.76 (*c* = 0.63, $CHCl_3$)

^{1}H NMR (300 MHz, $CDCl_3$): δ 7.81 (d, *J* = 8.8 Hz, 2H), 7.35 – 7.28 (m, 3H), 7.19 – 7.16 (m, 5H), 7.14 – 7.08 (m, 1H), 7.04 – 7.00 (m, 3H), 6.83 (d, *J* = 9.0 Hz, 2H), 3.78 (s, 3H), 3.75 – 3.69 (m, 2H), 3.58 – 3.50 (m, 4H), 3.44 – 3.38 (m, 1H), 3.00 (t, *J* = 6.8 Hz, 2H).

^{13}C NMR (75 MHz, $CDCl_3$): δ 197.0, 189.8, 163.4, 143.10, 141.8, 138.2, 130.4 (2C), 130.1, 129.2, 128.9 (2C), 128.6, 128.3 (2C), 128.0 (2C), 126.7, 126.6, 125.7 (2C), 113.6 (2C), 75.4, 66.6, 55.4, 42.2, 41.5, 38.4.

HRMS (ESI) m/z: $[M+H]^+$ Calcd for $C_{29}H_{29}N_2O_4$ 469.2122; Found: 469.2127.

The reaction was also performed on a large scale (1.0 mmol or 0.25 mmol of **1e**) employing the setup described in Fig. S2 with 2.5 mol% or 5 mol% of Δ-[Rh] and stirring the reaction for 17 h or 3h respectively. Under these conditions, **3e** was obtained in a 71% or 69% yield (335.3 mg or 81.1 mg) and 82% or 92% *ee* respectively.

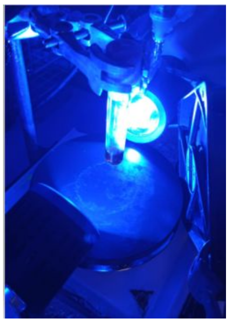

Fig. S2. Experimental setup employed during large scale photocatalytic reactions.

(*R*)-1-(4-Methoxyphenyl)-5-(1-(3-oxo-1-phenyl-3-(1-phenyl-1H-imidazol-2 yl)propyl)cyclopentyl)pentan-1-one (3f)

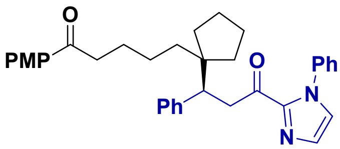

Following the general procedure with compound **1f** and **2a**. Purification by flash column chromatography (silica, gradient from 10% to 40% of EtOAc in cyclohexane) afforded 17.3 mg (65%) of the title compound **3f** as a colourless oil. $\mathbf{R_f}$ = 0.10 (5:1 cyclohexane:EtOAc). The enantiomeric excess was determined by SFC on a Daicel Chiralpak IB-3 column: CO_2/MeOH 90:10, flow rate 2.0 mL/min, τ_{major} = 7.11 min, τ_{minor} = 6.51 min, *ee* = 98%.

$[\alpha_D^{20}]$ = -52.8 (*c* = 0.16, $CHCl_3$)

^{1}H NMR (300 MHz, $CDCl_3$): δ 7.85 (d, *J* = 8.9 Hz, 2H), 7.25 – 7.07 (m, 9H), 6.99 (s, 1H), 6.85 (d, *J* = 8.9 Hz, 2H), 6.72 (d, *J* = 6.5 Hz, 2H), 3.89 – 3.83 (m, 1H), 3.79 (s, 3H), 3.40 (dd, *J* = 11.2, 3.7 Hz, 1H), 3.15 (dd, *J* = 15.9, 3.8 Hz, 1H), 2.79 (t, *J* = 7.4 Hz, 2H), 1.60 – 1.31 (m, 14H).

^{13}C NMR (75 MHz, $CDCl_3$): δ 199.0, 190.9, 163.2, 143.3, 142.3, 138.0, 130.3 (2C), 130.2, 129.9 (2C), 129.1, 128.8 (2C), 128.4, 127.6 (2C), 126.5, 126.1, 125.4 (2C), 113.6 (2C), 55.4, 48.6, 47.3, 40.8, 38.3, 37.6, 34.6 (2C), 25.4, 25.1 (2C), 24.0.

HRMS (ESI) m/z: $[M+H]^+$ Calcd for $C_{35}H_{39}N_2O_3$ 535.2955; Found: 535.2999.

(*R*)-1-(4-Methoxyphenyl)-7-(1-(3-oxo-1-phenyl-3-(1-phenyl-1H-imidazol-2-yl)propyl)cyclopentyl)heptan-1-one (3g)

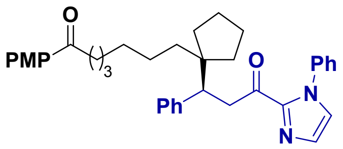

Following the general procedure with compound **1g** and **2a**. Purification by flash column chromatography (silica, gradient from 10% to 40% of EtOAc in cyclohexane) afforded 24.2 mg (86%) of the title compound **3g** as a colourless oil. **R*f*** = 0.38 (2:1 cyclohexane:EtOAc). The enantiomeric excess was determined by SFC on a Daicel Chiralpak ID-3 column: CO_2/MeOH 80:20, flow rate 2.0 mL/min, τ_{major} = 11.0 min, τ_{minor} = 13.6 min, *ee* = 99%.

$[\alpha_D^{20}]$ = -1.19 (*c* = 0.84, $CHCl_3$)

^{1}H NMR (300 MHz, $CDCl_3$): δ 7.87 (d, *J* = 8.9 Hz, 2H), 7.28 – 7.21 (m, 3H), 7.16 – 7.08 (m, 6H), 6.99 (brs, 1H), 6.86 (d, *J* = 8.9 Hz, 2H), 6.70 (d, *J* = 8.0 Hz, 2H), 3.90 – 3.85 (m, 1H), 3.79 (s, 3H), 3.39 (dd, *J* = 11.4, 3.6 Hz, 1H), 3.14 (dd, *J* = 15.9, 3.7 Hz, 1H), 2.81 (t, *J* = 7.5 Hz, 2H), 1.62 – 1.18 (m, 18H).

^{13}C NMR (75 MHz, $CDCl_3$): δ 199.2, 191.0, 163.3, 142.3, 138.0, 130.3 (2C), 130.2, 129.9 (2C), 129.0, 129.0, 128.8 (2C), 128.4, 127.6 (2C), 126.5, 126.0, 125.4 (2C), 113.6 (2C), 55.4, 48.6, 47.4, 40.8, 38.3, 37.7, 34.6, 34.5, 30.4, 29.4, 25.1 (2C), 24.6, 24.0.

HRMS (ESI) m/z: $[M+H]^+$ Calcd for $C_{37}H_{43}N_2O_3$ 563.3268; Found: 563.3287.

(*R*)-1-(4-Methoxyphenyl)-14-(1-(3-oxo-1-phenyl-3-(1-phenyl-1H-imidazol-2-yl)propyl)cyclopentyl)tetradecan-1-one (3h)

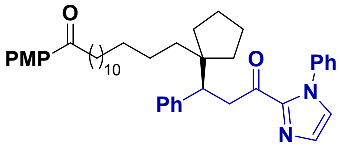

Following the general procedure with compound **1h** and **2a**. Purification by flash column chromatography (silica, gradient from 10% to 40% of EtOAc in cyclohexane) afforded 26.1 mg (78%) of the title compound **3h** as a colourless oil. **R*f*** = 0.60 (2:1 cyclohexane:EtOAc). The enantiomeric excess was determined by SFC on a Daicel Chiralpak IB-3 column: CO_2/MeOH 90:10, flow rate 2.0 mL/min, τ_{major} = 10.8 min, τ_{minor} = 9.84 min, *ee* = 97%.

$[\alpha_D^{20}]$ = -6.41 (*c* = 0.47, $CHCl_3$)

^{1}H NMR (300 MHz, $CDCl_3$): δ 7.87 (d, *J* = 8.9 Hz, 2H), 7.32 – 7.01 (m, 9H), 6.98 (d, *J* = 1.1 Hz, 1H), 6.85 (d, *J* = 8.9 Hz, 2H), 6.70 (d, *J* = 6.7 Hz, 2H), 3.86 (dd, J = 11.4, 4.5 Hz, 1H), 3.79 (s, 3H), 3.40 (dd, *J* = 11.3, 3.7 Hz, 1H), 3.13 (dd, *J* = 15.9, 3.7 Hz, 1H), 2.83 (t, *J* = 7.4 Hz, 2H), 1.45 – 1.15 (m, 32H).

^{13}C NMR (75 MHz, CDCl$_3$): δ 199.2, 191.1, 163.3, 143.4, 142.4, 138.1, 130.3 (2C), 130.2, 129.9 (2C), 129.1, 128.8 (2C), 128.4, 127.6 (2C), 126.4, 126.0, 125.4 (2C), 113.6 (2C), 55.4, 48.6, 47.3, 40.7, 38.3, 37.8, 34.6, 34.5, 30.9, 30.6, 29.7, 29.6, 29.6 (2C), 29.5, 29.5, 29.4, 25.1 (2C), 24.6, 24.1.

HRMS (ESI) m/z: [M+H]$^+$ Calcd for $C_{44}H_{57}N_2O_3$ 661.4364; Found: 661.4355.

(*S*)-9-(4-methoxyphenyl)-3-phenyl-1-(1-phenyl-1H-imidazol-2-yl)nonane-1,9-dione (3i)

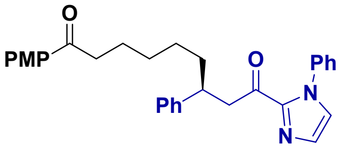

Following the general procedure with compound **1i** and **2a**. Purification by flash column chromatography (silica, gradient from 5% to 30% of EtOAc in cyclohexane) afforded 5.9 mg (25%) of the title compound **3i** as a colourless oil. **R*$_f$*** = 0.20 (2:1 cyclohexane:EtOAc). The enantiomeric excess was determined by SFC on a Daicel Chiralpak IB-3 column: CO_2/MeOH 90:10, flow rate 2.0 mL/min, τ_{major} = 7.45 min, τ_{minor} = 6.14 min, *ee* = 69%.

[α_D^{20}] = -36.7 (*c* = 0.26, CHCl$_3$)

^{1}H NMR (300 MHz, CDCl$_3$): δ 7.89 (d, *J* = 9.0 Hz, 2H), 7.39 – 7.36 (m, 3H), 7.25 – 7.10 (m, 7H), 7.05 – 6.98 (m, 2H), 6.91 (d, *J* = 9.0 Hz, 2H), 3.86 (s, 3H), 3.45 (d, *J* = 7.7 Hz, 2H), 3.33 – 3.26 (m, 1H), 2.81 (t, *J* = 7.4 Hz, 2H), 1.71 – 1.54 (m, 4H), 1.38 – 1.21 (m, 4H).

^{13}C NMR (75 MHz, CDCl$_3$): δ 199.1, 190.2, 163.3, 144.5, 138.0, 131.0, 130.3 (2C), 130.2, 128.9 (2C), 128.6, 128.3 (2C), 127.8 (2C), 126.8, 126.2, 125.6 (2C), 125.5, 113.6 (2C), 55.4, 46.0, 41.3, 38.1, 36.6, 29.3, 27.2, 24.4.

HRMS (ESI) m/z: [M+H]$^+$ Calcd for $C_{31}H_{33}N_2O_3$ 481.2486; Found: 481.2456.

(*R*)-4-(4-Methoxyphenyl)-3-phenyl-1-(1-phenyl-1H-imidazol-2-yl)decane-1,9-dione (3j)

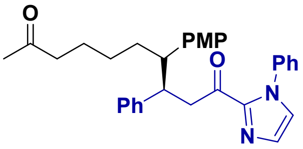

Following the general procedure with compound **1j** and **2a**. Purification by flash column chromatography (silica, gradient from 10% to 40% of EtOAc in cyclohexane) afforded 11.0 mg (44%) of the title compound **3j** as a yellowish oil as a 1:1 mixture of diastereomers. **R*$_f$*** = 0.15 (2:1 cyclohexane:EtOAc). The enantiomeric excess was determined by SFC on a Daicel Chiralpak IA column: CO_2/MeOH 80:20, flow rate 2.0 mL/min, τ_{major} = 3.68 min, τ_{minor} = 3.36 min, *ee* = 95%, τ_{major} = 5.25 min, τ_{minor} = 4.51, *ee* = 95%.

[α_D^{20}] = -3.14 (*c* = 0.48, CHCl$_3$)

^{1}H NMR (300 MHz, CDCl$_3$): δ 7.35 – 7.28 (m, 6H), 7.24 – 7.20 (m, 7H), 7.14 – 7.06 (m, 7H), 6.92 – 6.67 (m, 10H), 6.68 (d, *J* = 8.8 Hz, 2H), 3.78 (s, 3H), 3.73 (s, 3H), 3.60 – 3.43 (m, 5H), 2.97 – 2.90 (m, 1H), 2.85 – 2.66 (m, 2H), 2.27 (t, *J* = 7.1 Hz, 2H), 2.14 (t, J = 6.8 Hz, 2H), 2.04 (s, 3H), 1.98 (s, 3H), 1.68 – 1.34 (m, 8H), 1.11 – 1.01 (m, 2H), 0.92 – 0.86 (m, 2H).

^{13}C NMR (75 MHz, CDCl$_3$): δ 209.1, 209.0, 190.4, 190.2, 158.0, 157.8, 143.6, 143.3, 143.2, 142.0, 138.1, 135.1, 133.8, 129.8 (2C), 129.3 (2C), 129.1, 129.1 (2C), 128.8 (2C), 128.7 (2C), 128.5 (2C), 128.4, 128.3, 128.2 (2C), 127.5 (2C), 126.6, 126.5, 126.2, 125.9, 125.5 (2C), 125.5 (2C),

113.8 (2C), 113.1 (2C), 55.1, 55.0, 51.0, 50.4, 47.7, 46.9, 44.3, 43.6, 43.4, 42.5, 33.7, 32.4, 29.7, 29.7, 27.2, 26.9, 23.7, 23.4.

HRMS (ESI) m/z: $[M+H]^+$ Calcd for $C_{32}H_{35}N_2O_3$ 495.2642; Found: 495.2667.

(*S*)-1-(4-Methoxyphenyl)-6-(4-oxo-2-phenyl-4-(1-phenyl-1H-imidazol-2-yl)butoxy)hex-1-yn-3-one (3k)

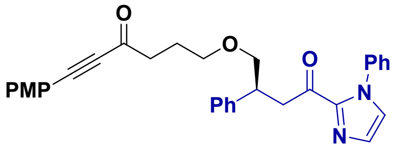

Following the general procedure with compound **1k** and **2a**. Purification by flash column chromatography (silica, gradient from 5% to 40% of EtOAc in cyclohexane) afforded 9.3 mg (37%) of the title compound **3k** as a yellowish oil. $\mathbf{R_f}$ = 0.20 (2:1 cyclohexane:EtOAc). The enantiomeric excess was determined by SFC on a Daicel Chiralpak IB-3 column: CO_2/MeOH 90:10, flow rate 2.0 mL/min, τ_{major} = 4.65 min, τ_{minor} = 5.77 min, *ee* = 88%.

$[\alpha_D^{20}]$ = -17.8 (*c* = 0.35, $CHCl_3$)

^{1}H NMR (300 MHz, $CDCl_3$): δ 7.44 (d, *J* = 8.9 Hz, 2H), 7.32 – 7.30 (m, 3H), 7.20 – 7.18 (m, 5H), 7.14 – 7.09 (m, 1H), 7.05 – 7.02 (m, 3H), 6.81 (d, *J* = 8.9 Hz, 2H), 3.77 (s, 3H), 3.64 – 3.32 (m, 7H), 2.53 (t, *J* = 7.4 Hz, 2H), 1.83 – 1.74 (m, 2H).

^{13}C NMR (75 MHz, $CDCl_3$): δ 189.8, 187.5, 161.6, 143.2, 141.9, 138.3, 135.1 (2C), 129.3, 128.9 (2C), 128.6, 128.3 (2C), 128.0 (2C), 126.8, 126.6, 125.7 (2C), 114.3 (2C), 111.8, 91.7, 87.7, 75.2, 69.7, 55.4, 42.2, 42.0, 41.5, 24.1.

HRMS (ESI) m/z: $[M+H]^+$ Calcd for $C_{32}H_{31}N_2O_4$ 507.2278; Found: 507.2314.

(*S*)-4-(4-(furan-2-yl)-4-oxobutoxy)-3-phenyl-1-(1-phenyl-1H-imidazol-2-yl)butan-1-one (3l)

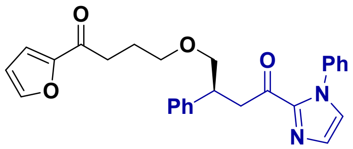

Following the general procedure with compound **1l** and **2a**. Purification by flash column chromatography (silica, gradient from 10% to 50% of EtOAc in cyclohexane) afforded 6.0 mg (27%) of the title compound **3l** as a yellowish oil. $\mathbf{R_f}$ = 0.13 (2:1 cyclohexane:EtOAc). The enantiomeric excess was determined by SFC on a Daicel Chiralpak IC column: CO_2/MeOH 80:20, flow rate 3.0 mL/min, τ_{major} = 11.8 min, τ_{minor} = 10.5 min, *ee* = 80%.

$[\alpha_D^{20}]$ = -50.9 (*c* = 0.17, $CHCl_3$)

^{1}H NMR (300 MHz, $CDCl_3$): δ 7.58 – 7.56 (m, 1H), 7.40 – 7.38 (m, 3H), 7.31 – 7.24 (m, 6H), 7.20 – 7.17 (m, 1H), 7.12 – 7.10 (m, 3H), 6.51 – 6.48 (m, 1H), 3.71 – 3.39 (m, 7H), 2.77 (t, *J* = 7.7 Hz, 1H), 1.94 – 1.79 (m, 2H).

^{13}C NMR (75 MHz, $CDCl_3$): δ 189.8, 189.2, 152.7, 146.2, 143.2, 142.0, 138.3, 129.3, 128.9 (2C), 128.6, 128.3 (2C), 128.0 (2C), 126.8, 126.5, 125.7 (2C), 117.0, 112.0, 75.1, 69.8, 42.2, 41.5, 35.0, 24.1.

HRMS (ESI) m/z: $[M+H]^+$ Calcd for $C_{27}H_{26}N_2O_4$ 443.1965; Found: 443.2038.

(*S*)-4-(4-(benzofuran-2-yl)-4-oxobutoxy)-3-phenyl-1-(1-phenyl-1H-imidazol-2-yl)butan-1-one (3m)

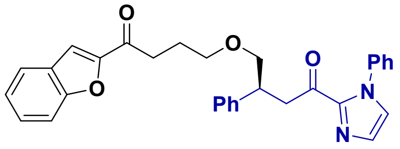

Following the general procedure with compound **1m** and **2a**. Purification by flash column chromatography (silica, gradient from 10% to 40% of EtOAc in cyclohexane) afforded 20.7 mg (84%) of the title compound **3m** as a yellowish oil. $\mathbf{R_f}$ = 0.25 (2:1 cyclohexane:EtOAc). The enantiomeric excess was determined by SFC on a Daicel Chiralpak IB-3 column: CO_2/MeOH 90:10, flow rate 2.0 mL/min, τ_{major} = 5.35 min, τ_{minor} = 5.96 min, *ee* = 92%.

$[\alpha_D^{20}]$ = +7.05 (*c* = 0.71, $CHCl_3$)

^{1}H NMR (300 MHz, $CDCl_3$): δ 7.62 (d, *J* = 7.1 Hz, 1H), 7.50 (d, *J* = 8.4 Hz, 1H), 7.42 – 7.36 (m, 2H), 7.31 – 7.28 (m, 3H), 7.24 – 7.16 (m, 6H), 7.12 – 7.10 (m, 1H), 7.05 – 7.01 (m, 3H), 3.67 – 3.37 (m, 7H), 2.84 (t, *J* = 7.6 Hz, 2H), 1.92 – 1.77 (m, 2H).

^{13}C NMR (75 MHz, $CDCl_3$): δ 191.1, 189.7, 155.6, 152.5, 143.1, 141.9, 138.2, 129.3, 128.9 (2C), 128.6, 128.3 (2C), 128.1, 128.0 (2C), 127.1, 126.8, 126.6, 125.7 (2C), 123.8, 123.2, 112.9, 112.4, 75.1, 69.7, 42.2, 41.5, 35.4, 24.1.

HRMS (ESI) m/z: $[M+H]^+$ Calcd for $C_{31}H_{29}N_2O_4$ 493.2122; Found: 493.2132.

(*S*)-4-(4-oxo-4-(phenanthren-9-yl)butoxy)-3-phenyl-1-(1-phenyl-1H-imidazol-2-yl)butan-1-one (3n)

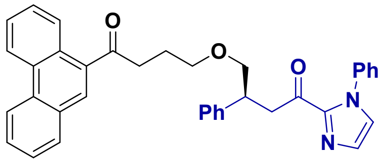

Following the general procedure with compound **1n** and **2a**. Purification by flash column chromatography (silica, gradient from 10% to 40% of EtOAc in cyclohexane) afforded 22.1 mg (80%) of the title compound **3n** as a colourless oil. $\mathbf{R_f}$ = 0.25 (2:1 cyclohexane:EtOAc). The enantiomeric excess was determined by SFC on a Daicel Chiralpak IA column: CO_2/MeOH 60:40, flow rate 3.0 mL/min, τ_{major} = 7.45 min, τ_{minor} = 8.29 min, *ee* = 92%.

$[\alpha_D^{20}]$ = -8.97 (*c* = 0.69, $CHCl_3$)

^{1}H NMR (300 MHz, $CDCl_3$): δ 8.62 (t, *J* = 7.6 Hz, 2H), 8.43 (d, *J* = 8.0 Hz, 1H), 7.97 (s, 1H), 7.86 (d, *J* = 8.0 Hz, 1H), 7.69 – 7.53 (m, 4H), 7.26 – 6.93 (m, 12H), 3.72 – 3.27 (m, 7H), 2.99 (t, *J* = 7.4 Hz, 2H), 1.90 (quint, *J* = 7.4 Hz, 2H).

^{13}C NMR (75 MHz, $CDCl_3$): δ 204.5, 189.6, 142.9, 141.9, 138.1, 135.3, 131.7, 130.7, 130.1, 129.8, 129.3, 129.0, 128.8 (2C), 128.6, 128.6, 128.3 (2C), 128.0 (2C), 127.4, 127.0, 127.0, 126.8, 126.6, 126.6, 126.6, 125.6 (2C), 122.8, 122.6, 75.2, 69.9, 42.4, 41.6, 38.7, 24.5.

HRMS (ESI) m/z: $[M+H]^+$ Calcd for $C_{37}H_{33}N_2O_3$ 553.2486; Found: 553.2490.

(*S*)-3-(4-chlorophenyl)-4-(4-(4-methoxyphenyl)-4-oxobutoxy)-1-(1-phenyl-1H-imidazol-2-yl)butan-1-one (3o)

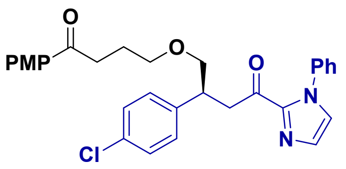

Following the general procedure with compound **1a** and **2o**. Purification by flash column chromatography (silica, gradient from 10% to 40% of EtOAc in cyclohexane) afforded 18.0 mg (70%) of the title compound **3o** as a yellow oil. **R*f*** = 0.13 (4:1 cyclohexane:EtOAc). The enantiomeric excess was determined by SFC on a Daicel Chiralpak IA column: CO_2/MeOH 80:20, flow rate 3.0 mL/min, τ_{major} = 10.3 min, τ_{minor} = 9.69 min, *ee* = 92%. $[\alpha_D^{20}]$ = +0.79 (*c* = 0.50, $CHCl_3$)

^{1}H NMR (300 MHz, $CDCl_3$): δ 7.87 (d, *J* = 9.0 Hz, 2H), 7.40 (dd, *J* = 5.1, 2.0 Hz, 3H), 7.26 – 7.20 (m, 5H), 7.13 – 7.10 (m, 3H), 6.91 (d, *J* = 9.0 Hz, 2H), 3.87 (s, 3H), 3.62 – 3.48 (m, 5H), 3.43 (t, *J* = 7.5 Hz, 2H), 2.85 (t, *J* = 7.5 Hz, 2H), 1.92 – 1.84 (m, 2H).

^{13}C NMR (75 MHz, $CDCl_3$): δ 198.6, 189.4, 163.3, 143.1, 140.6, 138.2, 132.2, 130.3 (2C), 130.1, 129.5, 129.4 (2C), 128.9 (2C), 128.7, 128.4 (2C), 127.0, 125.7 (2C), 113.6 (2C), 74.6, 70.0, 55.4, 42.0, 40.8, 34.6, 24.2.

HRMS (ESI) m/z: $[M+H]^+$ Calcd for $C_{30}H_{30}ClN_2O_4$ 517.1889; Found: 517.1854.

(*S*)-3-(4-fluorophenyl)-4-(4-(4-methoxyphenyl)-4-oxobutoxy)-1-(1-phenyl-1H-imidazol-2-yl)butan-1-one (3p)

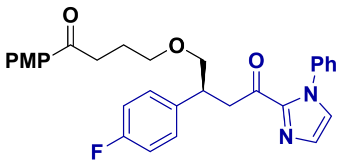

Following the general procedure with compound **1a** and **2p**. Purification by flash column chromatography (silica, gradient from 10% to 40% of EtOAc in cyclohexane) afforded 22.3 mg (89%) of the title compound **3p** as a yellowish oil. **R*f*** = 0.08 (2:1 cyclohexane:EtOAc). The enantiomeric excess was determined by SFC on a Daicel Chiralpak IA column: CO_2/MeOH 90:10, flow rate 3.0 mL/min, τ_{major} = 18.0 min, τ_{minor} = 17.0 min, *ee* = 94%. $[\alpha_D^{20}]$ = +1.55 (*c* = 0.90, $CHCl_3$)

^{1}H NMR (500 MHz, $CDCl_3$): δ 7.80 (d, *J* = 8.8 Hz, 2H), 7.34 – 7.30 (m, 3H), 7.19 – 7.14 (m, 3H), 7.06 – 7.04 (m, 3H), 6.87 – 6.83 (m, 4H), 3.79 (s, 3H), 3.59 – 3.39 (m, 5H), 3.36 (t, *J* = 7.8 Hz, 2H), 2.78 (td, *J* = 7.2, 2.8 Hz, 2H), 1.80 (quint, *J* = 6.5 Hz, 2H).

^{13}C NMR (126 MHz, $CDCl_3$): δ 198.6, 189.4, 163.3, 161.5 (d, *J* = 239.4 Hz), 142.9, 138.1, 137.7, 137.7, 130.3 (2C), 130.1, 129.5, 129.4, 129.2, 128.9 (2C), 128.7, 126.9, 125.7 (2C), 115.1 (d, *J* = 21.1 Hz), 113.6 (2C), 74.8, 70.0, 55.4, 42.3, 40.8, 34.6, 24.3.

^{19}F NMR (471 MHz, $CDCl_3$): δ -116.5.

HRMS (ESI) m/z: $[M+H]^+$ Calcd for $C_{30}H_{30}FN_2O_4$ 501.2184; Found: 501.2178.

(*S*)-3-(3-bromophenyl)-4-(4-(4-methoxyphenyl)-4-oxobutoxy)-1-(1-phenyl-1H-imidazol-2-yl)butan-1-one (3q)

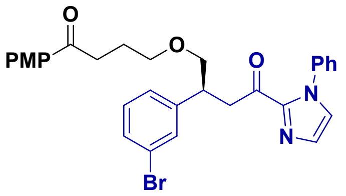

Following the general procedure with compound **1a** and **2q**. Purification by flash column chromatography (silica, gradient from 10% to 40% of EtOAc in cyclohexane) afforded 19.5 mg (70%) of the title compound **3q** as a yellow oil. **R*f*** = 0.10 (4:1 cyclohexane:EtOAc). The enantiomeric excess was determined

by SFC on a Daicel Chiralpak IA column: CO_2/MeOH 90:10, flow rate 3.0 mL/min, τ_{major} = 27.2 min, τ_{minor} = 29.1 min, *ee* = 92%.

$[\alpha_D^{20}]$ = -3.90 (*c* = 0.56, $CHCl_3$)

^{1}H NMR (300 MHz, $CDCl_3$): δ 7.81 (d, *J* = 9.0 Hz, 2H), 7.36 – 7.33 (m, 4H), 7.25 – 7.24 (m, 1H), 7.18 – 7.17 (m, 1H), 7.15 – 7.12 (m, 1H), 7.08 – 7.01 (m, 4H), 6.84 (d, *J* = 8.9 Hz, 2H), 3.79 (s, 3H), 3.61 – 3.40 (m, 5H), 3.36 (t, *J* = 7.6 Hz, 2H), 2.79 (t, *J* = 6.6 Hz, 2H), 1.85 – 1.76 (m, 2H).

^{13}C NMR (75 MHz, $CDCl_3$): δ 198.6, 189.3, 163.3, 144.6, 143.0, 138.2, 131.2, 130.3 (2C), 130.1, 129.9, 129.7, 129.5, 128.9 (2C), 128.7, 127.0, 126.69, 125.7 (2C), 122.3, 113.6 (2C), 74.5, 70.1, 55.4, 41.9, 41.1, 34.7, 24.3.

HRMS (ESI) m/z: $[M+H]^+$ Calcd for $C_{30}H_{30}BrN_2O_4$ 561.1383; Found: 561.1402.

(*S*)-4-(1-(4-(4-methoxyphenyl)-4-oxobutoxy)-4-oxo-4-(1-phenyl-1H-imidazol-2-yl)butan-2-yl)benzonitrile (3r)

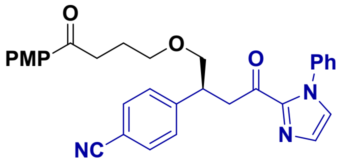

Following the general procedure with compound **1a** and **2r**. Purification by flash column chromatography (silica, gradient from 10% to 40% of EtOAc in cyclohexane) afforded 13.4 mg (53%) of the title compound **3r** as a yellow oil. **R_f** = 0.10 (4:1 cyclohexane:EtOAc). The enantiomeric excess was determined by SFC on a Daicel Chiralpak IA column: CO_2/MeOH 80:20, flow rate 3.0 mL/min, τ_{major} = 13.0 min, τ_{minor} = 10.4 min, *ee* = 66%.

$[\alpha_D^{20}]$ = -9.89 (*c* = 0.40, $CHCl_3$)

^{1}H NMR (300 MHz, $CDCl_3$): δ 7.86 (d, *J* = 8.9 Hz, 2H), 7.51 (d, *J* = 8.4 Hz, 2H), 7.43 – 7.36 (m, 5H), 7.24 (s, 1H), 7.16 – 7.14 (m, 3H), 6.93 (d, *J* = 8.9 Hz, 2H), 3.88 (s, 3H), 3.64 – 3.55 (m, 5H), 3.44 (t, *J* = 6.0 Hz, 2H), 2.84 (t, *J* = 7.4 Hz, 2H), 1.89 (quint, *J* = 6.8 Hz, 2H).

^{13}C NMR (75 MHz, $CDCl_3$): δ 198.4, 188.8, 163.4, 148.0, 142.8, 138.1, 132.1 (2C), 130.2 (2C), 130.1, 129.6, 128.9 (2C), 128.9 (2C), 128.8, 127.2, 125.7 (2C), 118.9, 113.7 (2C), 110.4, 74.0, 70.1, 55.5, 41.5, 41.3, 34.5, 24.1.

HRMS (ESI) m/z: $[M+H]^+$ Calcd for $C_{31}H_{30}N_3O_4$ 508.2231; Found: 508.2197.

(*S*)-4-(4-(4-methoxyphenyl)-4-oxobutoxy)-1-(1-phenyl-1H-imidazol-2-yl)-3-(4-(trifluoromethyl)phenyl)butan-1-one (3s)

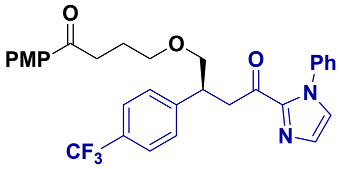

Following the general procedure with compound **1a** and **2s**. Purification by flash column chromatography (silica, gradient from 10% to 40% of EtOAc in cyclohexane) afforded 18.6 mg (66%) of the title compound **3s** as a yellow oil. **R_f** = 0.13 (4:1 cyclohexane:EtOAc). The enantiomeric excess was determined by SFC on a Daicel Chiralpak IA column: CO_2/MeOH 80:20, flow rate 3.0 mL/min, τ_{major} = 5.58 min, τ_{minor} = 5.04 min, *ee* = 78%.

$[\alpha_D^{20}]$ = -24.4 (*c* = 0.21, $CHCl_3$)

^{1}H NMR (500 MHz, $CDCl_3$): δ 7.80 (d, *J* = 8.8 Hz, 2H), 7.42 (d, *J* = 8.4 Hz, 2H), 7.34 – 7.31 (m, 5H), 7.18 (s, 1H), 7.06 – 7.03 (m, 3H), 6.83 (d, *J* = 9.0 Hz, 2H), 3.79 (s, 3H), 3.63 – 3.58 (m, 2H),

3.53 – 3.42 (m, 3H), 3.37 (t, *J* = 6.1 Hz, 2H), 2.79 (td, *J* = 7.0, 2.6 Hz, 2H), 1.81 (quint, *J* = 7.1 Hz, 2H).

^{13}C NMR (126 MHz, $CDCl_3$): δ 198.5, 189.0, 163.4, 146.3, 142.8, 138.1, 130.2 (2C), 130.1, 129.2, 128.9 (2C), 128.8, 128.7 (q, *J* = 33.1 Hz), 128.4 (2C), 127.1, 125.7 (2C), 125.2 (q, *J* = 4.1 Hz, 2C), 124.3 (q, *J* = 270.6 Hz), 113.6 (2C), 74.4, 70.1, 55.4, 41.9, 41.3, 34.6, 24.2.

^{19}F NMR (471 MHz, $CDCl_3$): δ -62.3.

HRMS (ESI) m/z: $[M+H]^+$ Calcd for $C_{31}H_{30}F_3N_2O_4$ 551.2152; Found: 551.2204.

(*S*)-3-(3-methoxyphenyl)-4-(4-(4-methoxyphenyl)-4-oxobutoxy)-1-(1-phenyl-1H-imidazol-2-yl)butan-1-one (3u)

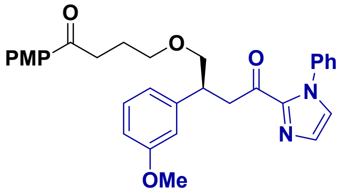

Following the general procedure with compound **1a** and **2u**. Purification by flash column chromatography (silica, gradient from 10% to 40% of EtOAc in cyclohexane) afforded 12.0 mg (47%) of the title compound **3u** as a yellowish oil. **R*f*** = 0.14 (4:1 cyclohexane:EtOAc). The enantiomeric excess was determined by SFC on a Daicel Chiralpak IC column: CO_2/MeOH 80:20, flow rate 3.0 mL/min, τ_{major} = 18.1 min, τ_{minor} = 15.8 min, *ee* = 90%.

$[\alpha_D^{20}]$ = -22.8 (*c* = 0.35, $CHCl_3$)

^{1}H NMR (500 MHz, $CDCl_3$): δ 7.82 (d, *J* = 9.0 Hz, 2H), 7.33 – 7.28 (m, 3H), 7.17 (s, 1H), 7.09 (t, *J* = 7.9 Hz, 1H), 7.06 – 7.02 (m, 3H), 6.83 (d, *J* = 8.8 Hz, 2H), 6.79 (d, *J* = 8.7 Hz, 1H), 6.77 – 6.76 (m, 1H), 6.65 (dd, *J* = 8.7, 3.0 Hz, 1H), 3.79 (s, 3H), 3.68 (s, 3H), 3.62 (dd, *J* = 15.9, 6.6 Hz, 1H), 3.58 – 3.47 (m, 3H), 3.41 – 3.34 (m, 3H), 2.80 (td, *J* = 7.2, 3.6 Hz, 2H), 1.82 – 1.76 (m, 2H).

^{13}C NMR (126 MHz, $CDCl_3$): δ 198.7, 189.6, 163.3, 159.6, 143.6, 143.0, 138.2, 130.3 (2C), 130.1, 129.3, 129.1, 128.9 (2C), 128.6, 126.8, 125.7 (2C), 120.3, 113.7, 113.6 (2C), 112.2, 75.0, 70.0, 55.4, 55.1, 42.3, 41.7, 34.8, 24.4.

HRMS (ESI) m/z: $[M+H]^+$ Calcd for $C_{31}H_{33}N_2O_5$ 513.2384; Found: 513.2400.

(*S*)-4-(4-(4-methoxyphenyl)-4-oxobutoxy)-1-(1-phenyl-1H-imidazol-2-yl)-3-(*p*-tolyl)butan-1-one (3v)

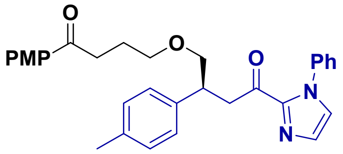

Following the general procedure with compound **1a** and **2v**. Purification by flash column chromatography (silica, gradient from 10% to 40% of EtOAc in cyclohexane) afforded 15.0 mg (60%) of the title compound **3v** as a yellow oil. **R*f*** = 0.11 (4:1 cyclohexane:EtOAc). The enantiomeric excess was determined by SFC on a Daicel Chiralpak IC column: CO_2/MeOH 70:30, flow rate 3.0 mL/min, τ_{major} = 10.9 min, τ_{minor} = 9.61 min, *ee* = 90%.

$[\alpha_D^{20}]$ = -1.93 (*c* = 0.36, $CHCl_3$)

^{1}H NMR (300 MHz, $CDCl_3$): δ 7.82 (d, *J* = 9.0 Hz, 2H), 7.31 – 7.29 (m, 3H), 7.18 (d, *J* = 6.8 Hz, 1H), 7.10 – 6.97 (m, 7H), 6.83 (d, *J* = 9.0 Hz, 2H), 3.79 (s, 3H), 3.64 – 3.30 (m, 7H), 2.79 (t, *J* = 6.9 Hz, 2H), 2.21 (s, 3H), 1.83 – 1.74 (m, 2H).

^{13}C NMR (75 MHz, $CDCl_3$): δ 198.7, 189.9, 163.3, 143.3, 138.9, 138.3, 136.0, 130.3 (2C), 130.2, 129.4, 129.0 (2C), 128.8 (2C), 128.5, 127.9 (2C), 126.8, 125.7 (2C), 113.6 (2C), 75.2, 70.0, 55.4, 42.3, 41.1, 34.8, 24.3, 21.0.

HRMS (ESI) m/z: $[M+H]^+$ Calcd for $C_{31}H_{33}N_2O_4$ 497.2435; Found: 497.2476.

(*S*)-4-(4-(4-methoxyphenyl)-4-oxobutoxy)-1-(1-phenyl-1H-imidazol-2-yl)-3-(*o*-tolyl)butan-1-one (3w)

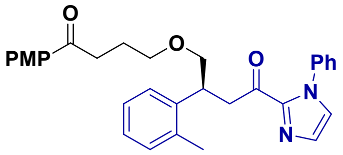

Following the general procedure with compound **1a** and **2w**. Purification by flash column chromatography (silica, gradient from 10% to 40% of EtOAc in cyclohexane) afforded 16.0 mg (65%) of the title compound **3w** as a yellowish oil. **R_f** = 0.10 (4:1 cyclohexane:EtOAc). The enantiomeric excess was determined by SFC on a Daicel Chiralpak IB-3 column: CO_2/MeOH 90:10, flow rate 2.0 mL/min, τ_{major} = 4.05 min, τ_{minor} = 4.84 min, *ee* = 92%. **$[\alpha_D^{20}]$** = -19.7 (*c* = 0.51, $CHCl_3$)

^{1}H NMR (300 MHz, $CDCl_3$): δ 7.82 (dd, *J* = 9.0, 2.5 Hz, 2H), 7.33 – 7.15 (m, 5H), 7.10 – 6.95 (m, 6H), 6.84 (dd, *J* = 9.0, 2.5 Hz, 2H), 3.93- 3.84 (m, 1H), 3.79 (s, 3H), 3.75 – 3.64 (m, 1H), 3.51 – 3.42 (m, 2H), 3.40 – 3.24 (m, 3H), 2.79 (t, *J* = 7.8 Hz, 2H), 2.26 (s, 3H), 1.84 – 1.71 (m, 2H).

^{13}C NMR (75 MHz, $CDCl_3$): δ 198.7, 189.8, 163.3, 142.9, 140.0, 138.1, 136.4, 130.3 (2C), 130.3, 130.1, 128.9 (2C), 128.9, 128.6, 126.7, 126.7, 126.3, 126.1, 125.6 (2C), 113.6 (2C), 74.8, 70.1, 55.4, 42.4, 37.0, 34.7, 24.4, 19.7.

HRMS (ESI) m/z: $[M+H]^+$ Calcd for $C_{31}H_{33}N_2O_4$ 497.2435; Found: 497.3430.

(*S*)-4-(4-(4-Methoxyphenyl)-4-oxobutoxy)-3-methyl-3-phenyl-1-(1-phenyl-1H-imidazol-2-yl)butan-1-one (3x)

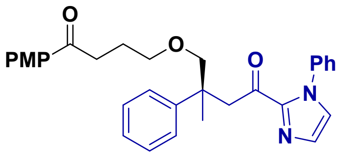

Following the general procedure with compound **1a** and **2x**. Purification by flash column chromatography (silica, gradient from 5% to 40% of EtOAc in cyclohexane) afforded 6.3 mg (25%) of the title compound **3x** as a yellow oil. **R_f** = 0.11 (4:1 cyclohexane:EtOAc). The enantiomeric excess was determined by SFC on a Daicel Chiralpak IG-3 column: CO_2/MeOH 70:30, flow rate 2.0 mL/min, τ_{major} = 12.7 min, τ_{minor} = 10.7, *ee* = 51%. **$[\alpha_D^{20}]$** = -4.23 (*c* = 0.24, $CHCl_3$)

^{1}H NMR (300 MHz, $CDCl_3$): δ 7.81 (d, *J* = 9.0 Hz, 2H), 7.34 – 7.26 (m, 4H), 7.20 – 7.15 (m, 4H), 7.09 – 7.04 (m, 1H), 7.02 – 7.00 (m, 1H), 6.95 – 6.92 (m, 2H), 6.83 (d, *J* = 9.0 Hz, 2H), 3.79 (s, 3H), 3.66 – 3.60 (m, 2H), 3.50 (dd, *J* = 12.2, 3.4 Hz, 2H), 3.35 (t, *J* = 6.1 Hz, 2H), 2.84 – 2.79 (m, 2H), 1.85 – 1.76 (m, 2H), 1.45 (s, 3H).

^{13}C NMR (75 MHz, $CDCl_3$): δ 198.8, 189.8, 163.3, 145.4, 143.7, 138.2, 130.3 (2C), 130.1, 128.9 (2C), 128.7, 128.5, 128.0 (2C), 126.7, 126.4, 126.0 (2C), 125.5 (2C), 113.6 (2C), 78.8, 70.2, 55.4, 46.2, 42.6, 34.8, 24.4, 23.7.

HRMS (ESI) m/z: $[M+H]^+$ Calcd for $C_{31}H_{33}N_2O_4$ 497.2435; Found: 497.2436.

(*R*)-4-(4-(4-methoxyphenyl)-4-oxobutoxy)-3-methyl-1-(1-phenyl-1H-imidazol-2-yl)butan-1-one (3y)

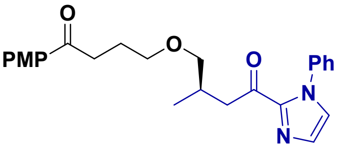

Following the general procedure with compound **1a** and **2y**. Purification by flash column chromatography (silica, gradient from 10% to 40% of EtOAc in cyclohexane) afforded 12.0 mg (57%) of the title compound **3y** as a yellowish oil. **R_f** = 0.11 (4:1 cyclohexane:EtOAc). The enantiomeric excess was determined by SFC on a Daicel Chiralpak IC column: CO_2/MeOH 90:10, flow rate 3.0 mL/min, τ_{major} = 24.6 min, τ_{minor} = 22.7 min, *ee* = 88%.

$[\alpha_D^{20}]$ = -49.6 (*c* = 0.21, $CHCl_3$)

^{1}H NMR (500 MHz, $CDCl_3$): δ 7.86 (d, *J* = 8.8 Hz, 2H), 7.40 – 7.34 (m, 3H), 7.21 – 7.19 (m, 3H), 7.08 (s, 1H), 6.84 (d, *J* = 9.0 Hz, 2H), 3.79 (s, 3H), 3.42 – 3.30 (m, 2H), 3.29 – 3.18 (m, 3H), 2.93 – 2.86 (m, 3H), 2.37 (h, *J* = 6.9 Hz, 1H), 1.87 – 1.78 (m, 2H), 0.89 (d, *J* = 6.8 Hz, 3H).

^{13}C NMR (126 MHz, $CDCl_3$): δ 198.7, 190.5, 163.3, 142.9, 138.3, 130.3 (2C), 130.2, 129.0 (2C), 128.8, 128.8, 126.8, 125.9 (2C), 113.6 (2C), 75.7, 70.0, 55.4, 43.6, 34.8, 30.3, 24.5, 17.2.

HRMS (ESI) m/z: $[M+H]^+$ Calcd for $C_{25}H_{29}N_2O_4$ 421.2122; Found: 421.2165.

(*S*)-3-((4-(4-methoxyphenyl)-4-oxobutoxy)methyl)-4-methyl-1-(1-phenyl-1H-imidazol-2-yl)pentan-1-one (3z)

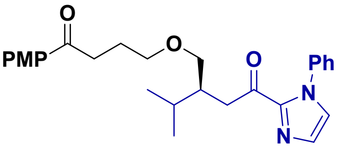

Following the general procedure with compound **1a** and **2z**. Purification by flash column chromatography (silica, gradient from 10% to 40% of EtOAc in cyclohexane) afforded 22.0 mg (49%) of the title compound **3z** as a brown oil. **R_f** = 0.20 (4:1 cyclohexane:EtOAc). The enantiomeric excess was determined by SFC on a Daicel Chiralpak IA column: CO_2/MeOH 90:10, flow rate 3.0 mL/min, τ_{major} = 7.87 min, τ_{minor} = 7.48 min, *ee* = 92%.

$[\alpha_D^{20}]$ = +4.51 (*c* = 0.18, $CHCl_3$)

^{1}H NMR (500 MHz, $CDCl_3$): δ 7.91 (d, *J* = 8.9 Hz, 2H), 7.43 – 7.40 (m, 3H), 7.26 – 7.22 (m, 3H), 7.12 – 7.11 (m, 1H), 6.90 (d, *J* = 8.9 Hz, 2H), 3.86 (s, 3H), 3.44 – 3.25 (m, 5H), 2.99 – 2.86 (m, 3H), 2.31 – 2.24 (m, 1H), 1.91 – 1.78 (m, 3H), 0.91 (d, *J* = 6.9 Hz, 6H).

^{13}C NMR (126 MHz, $CDCl_3$): δ 198.7, 191.2, 163.3, 143.4, 138.6, 130.3 (2C), 130.1, 129.3, 128.8 (2C), 128.5, 126.8, 125.8 (2C), 113.6 (2C), 72.3, 70.0, 55.4, 40.5, 38.6, 34.9, 28.8, 24.4, 19.9, 19.4.

HRMS (ESI) m/z: $[M+H]^+$ Calcd for $C_{27}H_{33}N_2O_4$ 449.2435; Found: 449.2434.

5. Synthetic transformation

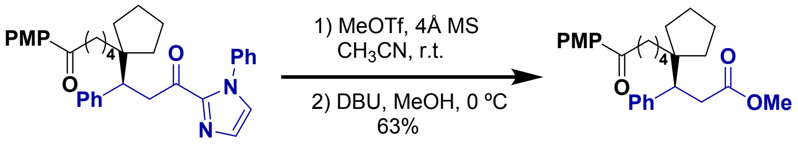

3f, 99% *ee* **4**, 99% *ee*

A solution of **3f** (17.3 mg, 0.032 mmol) in CH_3CN (1.0 mL) with 4 Å MS under nitrogen atmosphere was stirred for 30 min. Then, methyl trifluoromethanesulfonate (7.5 μL, 0.075 mmol) was added at room temperature. After stirring at this temperature for 2 h, MeOH (0.5 mL) and DBU (0.1 mL) were subsequently added to the reaction mixture at 0 °C. After stirring at 0 °C for 60 min, the solvent was evaporated under reduced pressure and the residue was purified by flash chromatography on silica gel (gradient from 10% to 40% of EtOAc in cyclohexane) to give **4** (8.5 mg, 0.02 mmol, 63% yield) as a colourless oil.

Methyl (*R*)-3-(1-(5-(4-methoxyphenyl)-5-oxopentyl)cyclopentyl)-3-phenylpropanoate (4)

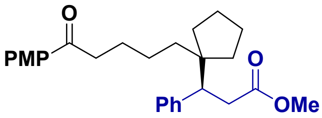

The enantiomeric excess was determined by SFC on a Daicel Chiralpak IG-3 column: CO_2/MeOH 90:10, flow rate 2.0 mL/min, τ_{major} = 25.7 min, τ_{minor} = 23.1, *ee* = 99%.

R_f = 0.63 (2:1 cyclohexane:EtOAc).

$[\alpha_D^{20}]$ = +4.63 (*c* = 0.26, $CHCl_3$)

^{1}H NMR (300 MHz, $CDCl_3$): δ 7.84 (d, *J* = 8.9 Hz, 2H), 7.19 – 7.09 (m, 5H), 6.85 (d, *J* = 8.9 Hz, 2H), 3.79 (s, 3H), 3.38 (s, 3H), 3.16 (dd, *J* = 9.6, 5.5 Hz, 1H), 2.79 (t, *J* = 7.4 Hz, 2H), 2.67 – 2.64 (m, 2H), 1.56 – 1.22 (m, 14H).

^{13}C NMR (75 MHz, $CDCl_3$): δ 198.9, 173.4, 163.3, 142.0, 130.3 (2C), 130.1, 129.2 (2C), 127.8 (2C), 126.3, 113.6 (2C), 55.4, 51.4, 48.4, 48.1, 38.1, 37.6, 36.5, 34.7, 34.5, 25.3, 25.1, 25.1, 23.9.

HRMS (ESI) m/z: $[M+H]^+$ Calcd for $C_{27}H_{34}O_4$ 423.2530; Found: 423.2535.

6. Mechanistic studies

a) Fluorescence quenching studies

For the steady-state and time resolved fluorescence quenching studies with the, increasing concentrations of quencher were added to a solution of Mes-Acrid photocatalyst in CH_2Cl_2 with an absorption of 0.12 at 455 nm under N_2 atmosphere (λ_{exc} = 455 nm).

a)

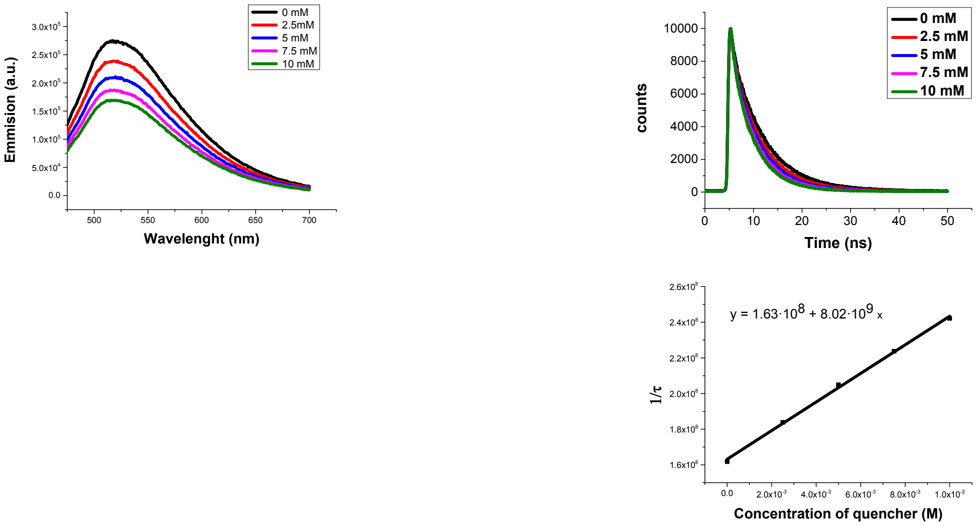

k_q = 8.02 x 10^9 $M^{-1}s^{-1}$

b)

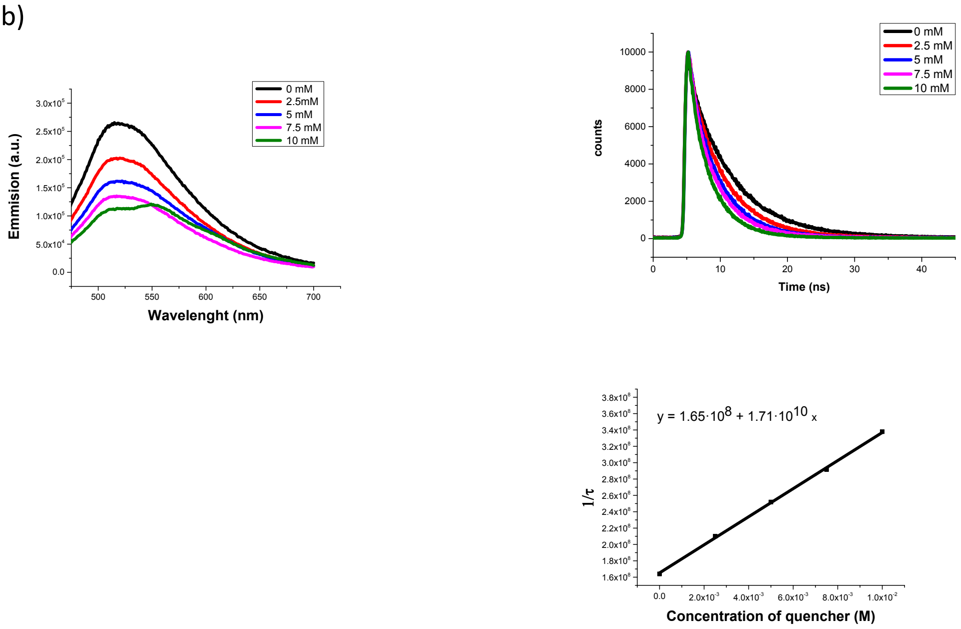

k_q = 1.71 x 10^{10} $M^{-1}s^{-1}$

c)

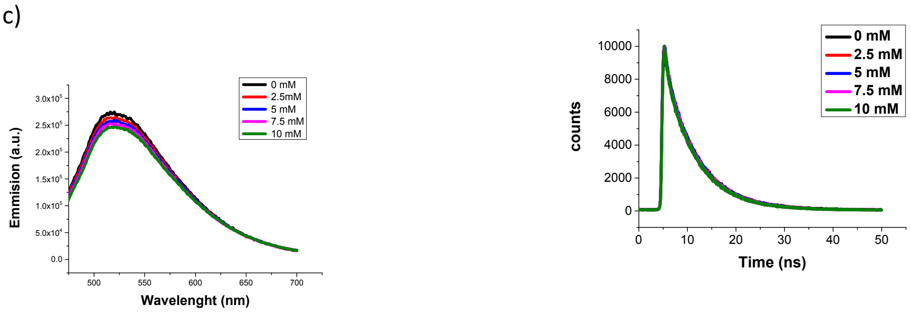

d)

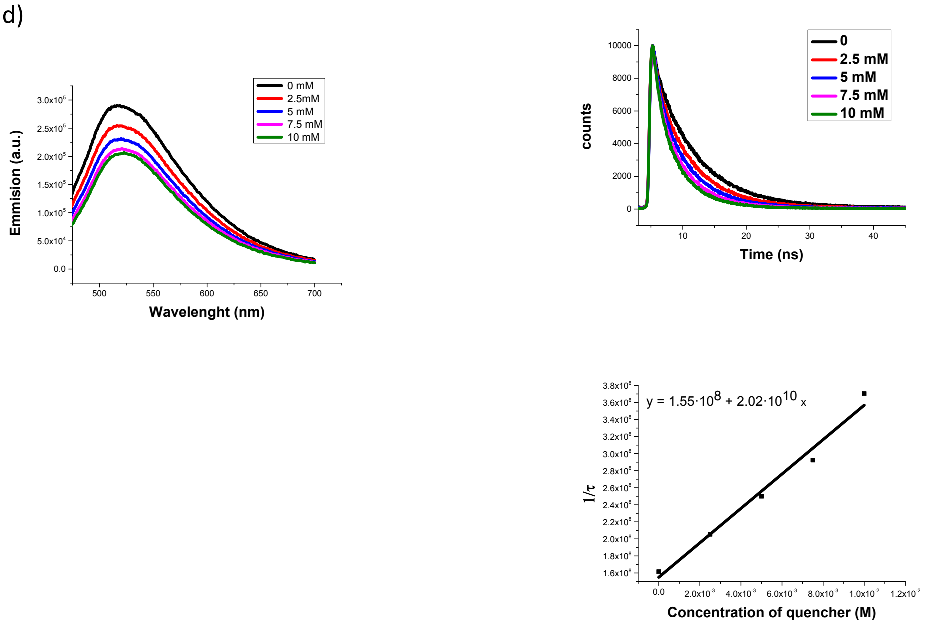

k_q = 2.02 x 10^{10} $M^{-1}s^{-1}$

Figure S3. Steady-state and time resolved fluorescence quenching of the Mes-acrid. photocatalyst with increasing concentrations of a) **1a**, Stern-Volmer plot to obtain k_q; b) **1a** + lutidine, Stern-Volmer plot to obtain k_q; c) **2a**; d) **2t**, Stern-Volmer plot to obtain k_q.

b) Cyclic voltammetry

CV measurements were carried out under argon atmosphere. The measurement were performed in MeCN containing 0.1 M tetra *n*-butylammonium tetrafluoroborate. A platinum electrode (working electrode), platinum wire counter electrode, and Ag/AgCl reference electrode was employed for the CV measurement. The scan rate was 100 mV/s, a step potential of 50 mV was applied.

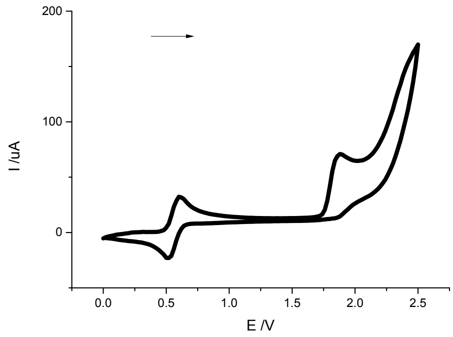

Figure S4. Cyclic voltammetry of **1a**, E p/2 = 1.81 eV vs SCE.

c) Quantum yield determination

A solution of ferrioxalate was chosen as actinometer following the procedure described by the IUPAC (subcommittee on photochemistry).[12] The procedure is based on the decomposition under irradiation of ferric ions to ferrous ions which are complexed by 1,10-phenanthroline. This photochemical transformation has a known quantum yield and the complexation of Fe^{2+} with 1,10-phenanthroline can be monitored by UV-Visible absorption since its extinction coefficient at 510 nm is known (ε =11100 M^{-1} cm^{-1}). Therefore, the moles transformed can be related with the moles of photons absorbed by the equation [1].

$$\Phi = \frac{mol\ transformed}{photons\ absorbed} \qquad [1]$$

The complete procedure should be done under a red safe-light environment. At 465 nm ferroxilate has a Φ = 0.85.[13] 0.006, 0.012, or 0.15 M solutions of $K_3[Fe(C_2O_4)_3]$ $3H_2O$ can be used for actinometry. In this case, we chose a concentration of 0.15 M. The solutions were prepared and stored in a dark laboratory:

1. Potassium ferrioxalate solution (0.15 M): 368.4 mg of $K_3[Fe(C_2O_4)_3]$ $3H_2O$ (commercially available) and 26.6 μL of H_2SO_4 were added into a 5 mL volumetric flask and filled to the mark with Milli-Q water.

2. Phenanthroline solution (0.15 M): 1.35 g of 1,10-phenantroline monohydrate were added to 50 mL volumetric flask and filled to the mark with MilliQ water.

3. Buffer solution: 4.94 g of NaOAc and 1 mL of H_2SO_4 were added to 100 mL volumetric flask and filled to the mark with MilliQ water.

4. Model reaction solution: A vial equipped with a magnetic stir bar was charged with alcohol **1e** (0.2 mmol, 1.0 equiv.), alkene **2** (0.2 mmol, 1.0 equiv.), Δ-Rh (0.05 equiv.) and 1 mL of a stock solution of [Mes-Acr]ClO_4 in DCM (4.85 μM) followed by 2,6-lutidine (0.25 equiv.). Degasification of the reaction mixture was performed via freeze-pump-thaw cycling (3 x 10 min under vacuum). The vial was then backfilled with N_2 and stirred under 465 nm LED irradiation (22.0216 W/m^2 intensity; approximate distance was 2 cm from the vial) at 20°C.

Actinometry procedure: Due to the reactor setup (Figure S20), the simultaneous irradiation of both the actinometer solution and model reaction is not feasible. However, the stability of the irradiation light was checked through radiometer measurements (from spectro-radiometer equipment Stellarnet model Blue-Wave UV-NB50). Therefore, we assumed that consecutive measurements of both actinometer and model reaction are comparable. In addition, using the same spectrometer, the LED source spectrum was measured, detecting a maximum wavelength of emission of 465 nm (Figure S21).

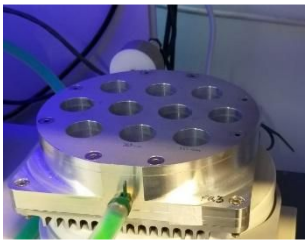

Figure S5. LED setup of the reaction.

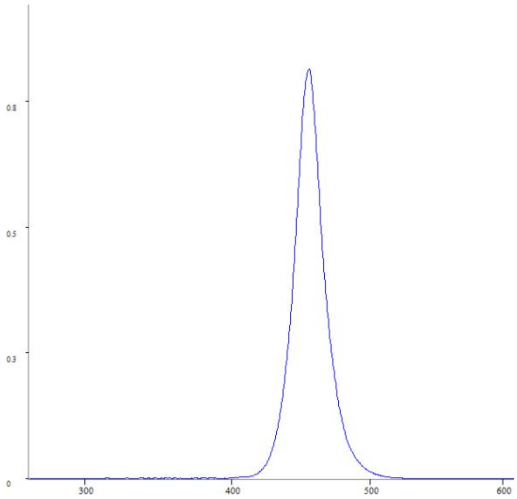

Figure S6. Emission spectrum of the blue LED of the photochemical reactor (λ_{max}= 465 nm).

2 mL of potassium ferrioxalate solution (0.15 M) were introduced into the photoreactor under dark conditions while being stirred. Then, the LED was switched on. Every 5 s the light was switched off and a 0.1 mL aliquot was taken. To each aliquot, 2 mL of buffer solution and 0.5 mL of 1,10-phenanthroline 0.15 M were added and the final volume was raised to 10 mL with MilliQ water. Then 83 µL of this solution were diluted to 5 mL with MilliQ water. As a blank sample, a solution was prepared with 0.1 mL of potassium ferrioxalate solution (0.15 M) before irradiation, 2 mL of buffer solution and 0.5 mL of 1,10-phenanthroline 0.15 M in a 10 mL of volumetric flask filled with water until the mark, and 83 µL of this solution were diluted to 5 mL with MilliQ water. The absorbance spectrum of each sample was monitored at 510 nm. The absorbance to each time was related with the photochemically produced Fe^{2+} ions across the Lambert-Beer Law (Equation [2]), where V_1 is the irradiated volume (noting that the initial volume is 2 mL but it changes as the aliquots are taken); V_2 is the aliquot volume (0.1 mL), V_3 is the final volume after addition of 1,10-

phenanthroline and buffer (10 mL). *b* is referred to the optical pathway (1 cm), *ΔA* (510 nm) is the difference in absorbance between the irradiated solution and the blank sample, *ε* (510 nm) is the extinction coefficient of the complex formed by Fe(II) and 1,10-phenanthroline (ca. 11100 M^{-1} cm^{-1}).

$$moles\ of\ Fe^{2+} = \frac{V_1 \cdot V_3 \cdot \Delta A_{(510\ nm)}}{10^3 \cdot V_2 \cdot b \cdot \varepsilon_{(510\ nm)}} \qquad [2]$$

The moles of Fe^{2+} formed (x) are plotted as a function of time (t) (Figure S).

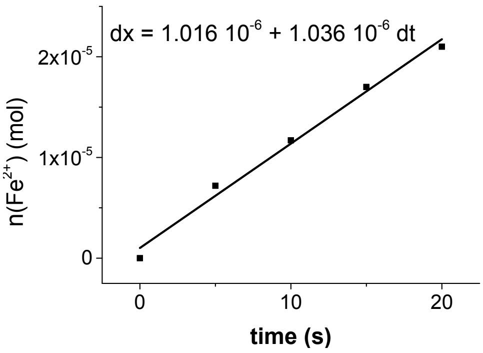

Figure S7. Actinometer.

The slope of this line (*dx*/*dt*) was correlated to the moles of incident photons by unit of time ($q^0_{n,p}$) using the following equation [3]:

$$q^0_{n,p} = \frac{dx/dt}{\Phi_{(\lambda)} \cdot [1 - 10^{-A(\lambda)}]} \qquad [3]$$

Where $\Phi_{(\lambda)}$ is the quantum yield of the actinometer reaction at the irradiated wavelength, in this case being 0.85 at 465 nm for 0.15 M dilution[32] and $A_{(\lambda)}$ is the absorbance of the actinometer solution (ferrioxalate) at the irradiated wavelength (465 nm). The absorbance at 465 nm was measured with an Agilent 8453 UV-visible Spectroscopy System using a quartz cuvette with 1 cm of optical pathway.

Therefore, the moles of incident photons by unit of time ($q^0_{n,p}$) was determined as 1.036 10^{-5} einstein s^{-1}.

The kinetics of the reaction under study were done as follows: the photoreactor (blue LEDs) was switched on and the reaction mixture was stirred. At 10, 15, 20, 30 and 60 minutes an aliquot of 0.05 mL was taken from the reaction mixture under a positive flow of nitrogen and the solvent was evaporated. Then 0.02 mL of a solution of trimethoxybencene in $CDCl_3$ (0.5 M) were added, and the resulting solution diluted with 0.4 mL of $CDCl_3$. Thus, the conversion of the reaction at the different indicated time was determined by ^1H NMR. Knowing the initial molar concentration, the determination of the moles of photo-converted product is possible.

Plotting the moles of product versus the irradiation time, the slope *dx*/*dt* can be related with the quantum yield across the equation [2] being equal to time ($q^0_{n,p}$) $\Phi_{(\lambda)}$□[1-10-$A_{(\lambda)}$]. Therefore, the quantum yield at the wavelength of irradiation Φ (465 nm) can be calculated once A (465 nm) is

determined. To measure A (465 nm), a model reaction solution was added to a 1 mm optical pathway cuvette and the UV-Visible spectrum was recorded obtaining an absorbance of 2.02.

Therefore, the quantum yield for the reaction is: Φ = 0.05 = 5%.

7. Stereochemical assignments and computational details

a) Synthesis of 4ab

The absolute configuration of the new chiral centre was determined by SFC correlation with the derivatization of a compound reported in the literature (**SI14**)[14].

Synthesis of ester **4ab** from the aldehyde **SI14**, obtained through the organo-photocatalytic method described by Melchiorre:[14]

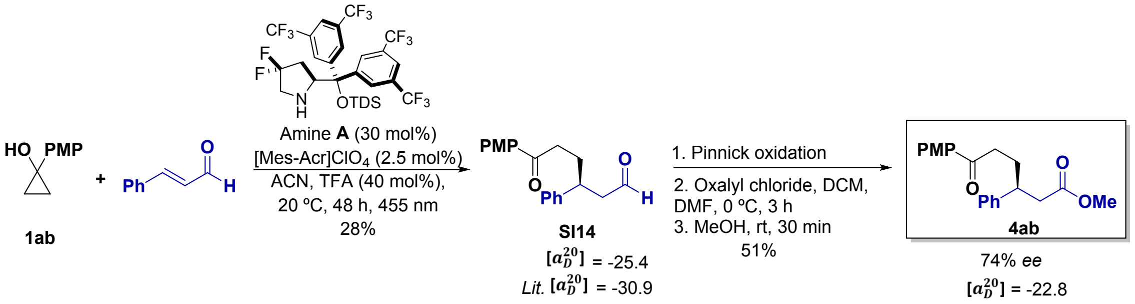

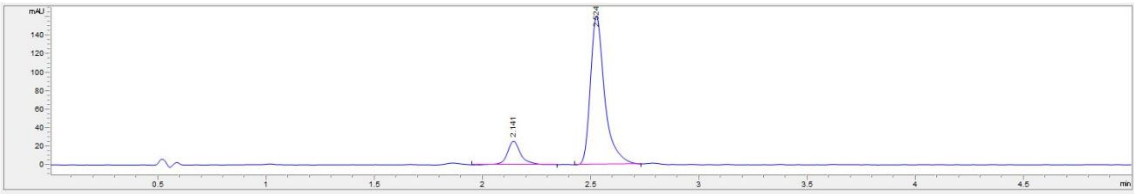

#	Time	Type	Area	Height	Width	Area%	Symmetry
1	2.141	BB	109.6	25.6	0.064	12.924	0.817
2	2.524	BB	738.4	161.9	0.0692	87.076	0.674

Fig. S8. SFC chromatogram for enantioenriched **4ab**.

Synthesis of the ester **4ab** from **3ab**, obtained following our Lewis acid photocatalytic procedure:

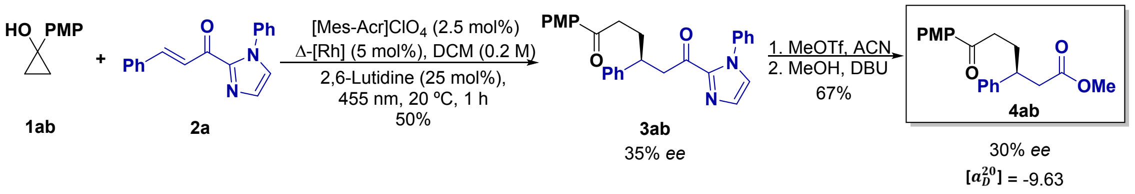

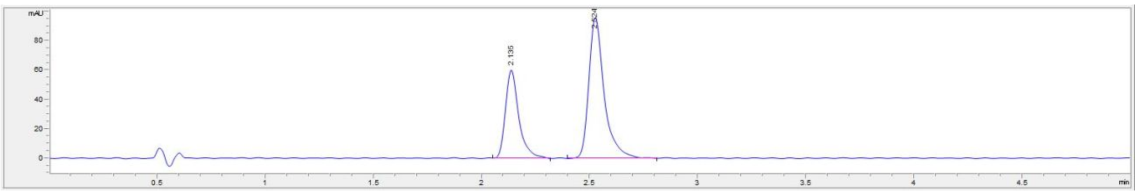

#	Time	Type	Area	Height	Width	Area%	Symmetry
1	2.135	BB	258.6	60.4	0.0659	35.177	0.692
2	2.524	BB	476.4	96.5	0.0736	64.823	0.671

Fig. S9. SFC chromatogram for enantioenriched **4ab**.

1-(4-methoxyphenyl)cyclopropan-1-ol (1ab)

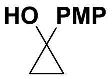

Following the reported procedure[15] on a 5.0 mmol scale. Purification by flash column chromatography (silica, gradient from 5% to 30% of EtOAc in cyclohexane) afforded 490 mg (60%) of the title compound **1ab** as a white solid. **R_f** = 0.25 (5:1 cyclohexane:EtOAc).

^{1}H NMR (300 MHz, DMSO): δ 7.15 (d, *J* = 6.7 Hz, 2H), 6.83 (d, *J* = 6.9 Hz, 2H), 5.78 (s, 1H), 3.71 (s, 3H), 0.99 (m, 2H), 0.95 – 0.70 (m, 2H).

(*S*)-6-(4-methoxyphenyl)-6-oxo-3-phenylhexanal (SI14)[14]

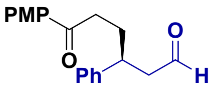

Following the reported procedure[14] on a 0.8 mmol scale with compound **1ab**, (*E*)-cinnamaldehyde, [Mes-Acr]ClO_4, the amine catalyst A in a 0.2 M acetonitrile solution of TFA (40 mol%) at 20 ºC for 48 h under 455 nm light irradiation. Purification by flash column chromatography (silica, gradient from 5% to 30% of EtOAc in cyclohexane) afforded 65.7 mg (28%) of the title compound **SI14** as a yellow oil. **R_f** = 0.23 (5:1 cyclohexane:EtOAc).

$[\alpha_D^{20}]$ = -25.4 (*c* = 0.10, $CHCl_3$)

Lit. $[\alpha_D^{20}]$ = -30.9 (*c* = 0.10, $CHCl_3$)

^{1}H NMR (300 MHz, $CDCl_3$): δ 9.69 (t, *J* = 1.9 Hz, 1H), 7.81 (d, *J* = 10.1 Hz, 2H), 7.38 – 7.16 (m, 5H), 6.88 (d, *J* = 10.1 Hz, 2H), 3.85 (s, 3H), 3.34 – 3.24 (m, 1H), 2.88 – 2.65 (m, 4H), 2.23 – 2.11 (m, 1H), 2.06 – 1.96 (m, 1H).

Synthesis of methyl (*S*)-6-(4-methoxyphenyl)-6-oxo-3-phenylhexanoate (**4ab**)

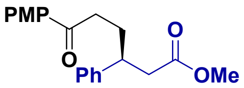

This compound was synthesized in a two-step process from (*S*)-6-(4-methoxyphenyl)-6-oxo-3-phenylhexanal (**SI14**):

Step 1. Following a reported procedure,[16] to a glass vial charged with **SI14** (1.0 equiv., 0.22 mmol, 65.7 mg), $NaClO_2$ (1.1 equiv., 24.6 mg), NaH_2PO_4 (1.1 equiv., 36.9 mg) and a mixture of *t*BuOH/H_2O (1:1) (2.0 mL) was added H_2O_2 35%w/w (0.2 mL) and the reaction was stirred at rt for 1 h. The reaction was treated with ethyl acetate and washed with water twice. The organic layer was dried under $MgSO_4$ and concentrated in vacuo to afford 62.4 mg of the corresponding carboxylic acid as a yellowish solid (91% yield) which was used in the next step without further purification.

Step 2. Following a reported procedure,[17] the carboxylic acid (62.4 mg, 1.0 equiv.) obtained from step A was dissolved in dry DCM (1.0 mL) under argon atmosphere. Then, oxalyl chloride (1.2 equiv., 0.24 mmol, 16 µL) was added dropwise followed by 2 drops of DMF. The reaction was stirred at rt for 3 h. After this time, MeOH (0.5 mL) was added and the reaction was stirred at rt for 30 min. After removing the solvent under reduced pressure, purification by flash column chromatography (silica, gradient from 5% to 30% of EtOAc in cyclohexane) afforded 36.3 mg (51%) of the title compound **4ab** as a yellowish solid. **R_f** = 0.45 (2:1 cyclohexane:EtOAc). The

enantiomeric excess was determined by SFC on a Daicel Chiralpak IB-3 column: CO_2/MeOH 95:5, flow rate 2.0 mL/min, τ_{major} = 2.51 min, τ_{minor} = 2.13 min, *ee* = 74%.

$[\alpha_D^{20}]$ = -22.8 (*c* = 1.5, $CHCl_3$)

^{1}H NMR (300 MHz, $CDCl_3$): δ 7.80 (d, *J* = 9.0 Hz, 2H), 7.40 – 7.09 (m, 5H), 6.87 (d, *J* = 9.0 Hz, 2H), 3.84 (s, 3H), 3.58 (s, 3H), 3.21 – 3.16 (m, 1H), 2.94 – 2.65 (m, 4H), 2.21 – 2.10 (m, 1H), 2.06 – 1.93 (m, 1H).

^{13}C NMR (75 MHz, $CDCl_3$): δ 198.3, 172.5, 163.4, 143.1, 130.2 (2C), 129.9, 128.6 (2C), 127.5 (2C), 126.8, 113.6 (2C), 55.4, 51.5, 41.7, 41.7, 36.0, 30.5.

HRMS (ESI) m/z: $[M+H]^+$ Calcd for $C_{20}H_{22}O_4$ 327.1591; Found: 327.1604.

(*S*)-6-(4-methoxyphenyl)-3-phenyl-1-(1-phenyl-1H-imidazol-2-yl)hexane-1,6-dione (3ab)

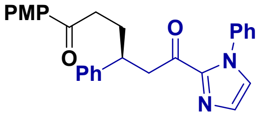

Following the general procedure for the photocatalytic reaction on a 0.1 mmol scale with compound **1ab** and **2a**. Purification by flash column chromatography (silica, gradient from 10% to 40% of EtOAc in cyclohexane) afforded 21.7 mg (50%) of the title compound **3ab** as a yellow oil. **R*f*** = 0.25 (2:1 cyclohexane:EtOAc). The enantiomeric excess was determined by SFC on a Daicel Chiralpak ID-3 column: CO_2/MeOH 80:20, flow rate 2.0 mL/min, τ_{major} = 4.54 min, τ_{minor} = 5.05 min, *ee* = 35%.

$[\alpha_D^{20}]$ = +1.34 (*c* = 0.74, $CHCl_3$)

^{1}H NMR (300 MHz, $CDCl_3$): δ 7.76 (d, *J* = 8.0 Hz, 2H), 7.40 – 7.37 (m, 3H), 7.30 – 7.19 (m, 6H), 7.13 – 7.12 (m, 1H), 7.08 – 7.05 (m, 2H), 6.85 (d, *J* = 9.0 Hz, 2H), 3.83 (s, 3H), 3.53 (d, *J* = 8.0 Hz, 2H), 3.43 – 3.35 (m, 1H), 2.87 – 2.64 (m, 2H), 2.21 – 1.97 (m, 2H).

^{13}C NMR (75 MHz, $CDCl_3$): δ 198.5, 189.8, 163.3, 143.7, 143.2, 138.3, 130.2 (2C), 130.0, 129.5, 128.9 (2C), 128.6, 128.5 (2C), 127.9 (2C), 126.9, 126.5, 125.7 (2C), 113.6 (2C), 55.4, 46.0, 40.9, 36.2, 31.0.

HRMS (ESI) m/z: $[M+H]^+$ Calcd for $C_{28}H_{26}N_2O_3$ 438.1943; Found: 438.1942.

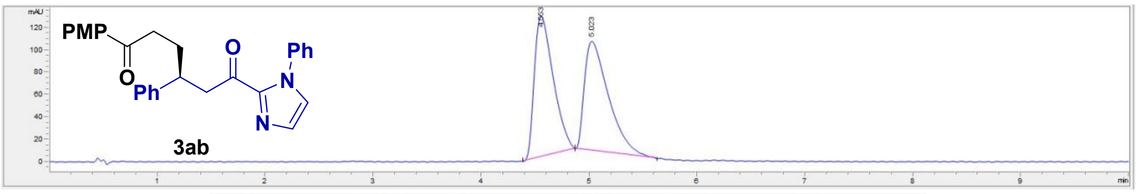

#	Time	Type	Area	Height	Width	Area%	Symmetry
1	4.553	BB	1547.1	126.8	0.1864	50.233	0.526
2	5.023	BB	1532.8	98.2	0.2361	49.767	0.409

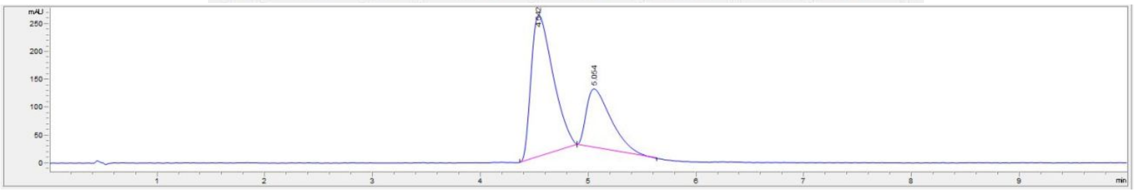

#	Time	Type	Area	Height	Width	Area%	Symmetry
1	4.542	BB	3579.6	253.2	0.2209	67.975	0.506
2	5.054	BB	1686.4	104.9	0.2416	32.025	0.412

Fig. S10. SFC chromatograms for racemic and enantioenriched **3ab**.

Synthesis of methyl (*S*)-6-(4-methoxyphenyl)-6-oxo-3-phenylhexanoate (**4ab**)

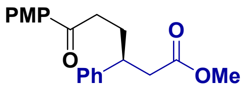

A solution of **3ab** (21.7 mg, 0.05 mmol) in CH_3CN (1.0 mL) with 4 Å MS under nitrogen atmosphere was stirred for 30 min. Then, methyl trifluoromethanesulfonate (7.5 μL, 0.075 mmol) was added at room temperature. After stirring at this temperature for 2 h, MeOH (0.5 mL) and DBU (0.1 mL) were subsequently added to the reaction mixture at 0 °C. After stirring at 0 °C for 60 min, the solvent was evaporated under reduced pressure and the residue was purified by flash chromatography on silica gel (gradient from 20% to 60% of EtOAc in cyclohexane) to give **4ab** (10.9 mg, 0.034 mmol, 67% yield) as a yellowish solid.

The enantiomeric excess was determined by SFC on a Daicel Chiralpak IB-3 column: CO_2/MeOH 95:5, flow rate 2.0 mL/min, τ_{major} = 2.52 min, τ_{minor} = 2.14 min, *ee* = 30%.

$[\alpha_D^{20}]$ = -9.63 (*c* = 0.22, $CHCl_3$)

b) Computational details. DFT calculations.

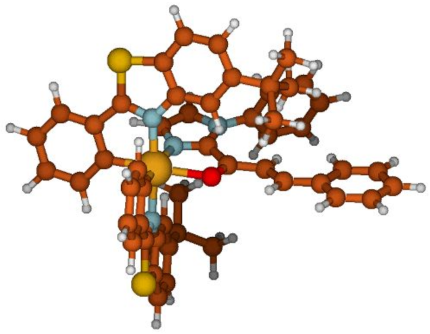

E = -3208.72428121 a.u. (no imaginary frequencies, it is a minimum in the potential energy surface)

Rh	-1.01315400	1.03916700	0.28407300
C	-2.46953200	2.39606300	0.53102800
C	-2.37942000	3.78983900	0.41063800
C	-3.74600500	1.84264000	0.82398600
C	-3.51479100	4.59647700	0.56879900
H	-1.41995300	4.26234100	0.19071500
C	-4.88613500	2.65006100	0.98276900
C	-4.76711900	4.03280400	0.85476900
H	-3.42122900	5.68159700	0.46954900
H	-5.85809200	2.19997700	1.20364600

H	-5.64417400	4.67216900	0.97697500
C	-0.46015700	1.32501400	2.16857700
C	0.63314300	2.22051900	2.33156100
C	-0.97251100	0.69991600	3.31329000
C	1.19582100	2.47292300	3.59539400
C	-0.41116600	0.95412000	4.57170800
H	-1.81223900	0.00721200	3.23373700
C	0.66983700	1.83686100	4.71782400
H	2.03921200	3.16160900	3.69643600
H	-0.82435200	0.45619600	5.45330200
H	1.09692300	2.02527100	5.70514100
C	-3.73368700	0.39703100	0.92428600
C	-4.02857800	-2.03973800	1.11916700
C	-2.70632500	-1.61673200	0.86163600
C	-4.33058300	-3.40019900	1.23982800
C	-1.67702500	-2.56211400	0.73610700
C	-3.29646900	-4.32018700	1.09226900
H	-5.35045700	-3.73437900	1.43897000
C	-1.95792000	-3.92549300	0.83827900
H	-0.67280000	-2.19750500	0.55789800
H	-3.53860700	-5.38102900	1.17731500
C	1.08224300	2.81042600	1.08717900
C	2.05052400	3.95072600	-0.86896400
C	0.95703100	3.10072700	-1.15490400
C	2.68385900	4.65850300	-1.89152000
C	0.48800100	2.97582700	-2.46934000
C	2.21151400	4.50625400	-3.19575000
H	3.52766200	5.31784700	-1.68037600
C	1.11264000	3.67252000	-3.51181000
H	-0.37261900	2.33793200	-2.64982900
H	2.71850500	5.06131500	-3.98391300
S	-5.09286400	-0.65022700	1.23542500

S	2.39120000	3.93925200	0.85382900
N	0.45040100	2.47305100	-0.02557300
N	-2.58391500	-0.23872700	0.75564300
C	0.62991300	3.47499200	-4.96275800
C	1.14698900	2.10521200	-5.46096300
H	0.81588200	1.91938700	-6.49565800
H	2.24782300	2.06951600	-5.44084900
H	0.76894100	1.28398700	-4.83618800
C	1.15918700	4.57297300	-5.90388400
H	0.74337300	4.42633200	-6.91265300
H	0.86660400	5.57738400	-5.55900800
H	2.25537200	4.54602200	-5.99822500
C	-0.91273000	3.49235400	-5.02923800
H	-1.36626800	2.68260400	-4.44061200
H	-1.31451500	4.44812200	-4.65727500
H	-1.24657500	3.35917700	-6.07020200
C	-0.86618200	-4.99612300	0.66546400
C	0.51740800	-4.37191000	0.41654600
H	0.52681300	-3.75911400	-0.49740300
H	0.84137200	-3.73743800	1.25546600
H	1.26881900	-5.16607300	0.28951500
C	-1.22039800	-5.88494300	-0.54881600
H	-2.18772900	-6.39333900	-0.41839200
H	-1.27356700	-5.28367800	-1.47070100
H	-0.45044300	-6.66074000	-0.68977100
C	-0.78703700	-5.86448300	1.94093600
H	-0.53699400	-5.24839000	2.81934400
H	-1.73675800	-6.38071000	2.14719300
H	-0.00662300	-6.63438700	1.82888100
C	-0.60910100	-0.34101400	-2.39017700
C	-0.75161500	-0.80265100	-3.75615500
H	-0.05166300	-1.55085000	-4.11596700

O	-1.42272200	0.45199900	-1.86184400
C	-1.68929000	-0.27049100	-4.58385300
C	0.53606900	-0.74127700	-1.55386300
N	1.69262200	-1.42084200	-1.85487700
C	1.72153900	-0.82354000	0.27350800
C	2.44425400	-1.47688900	-0.71094600
H	1.97818500	-0.65532600	1.31669000
H	3.41673300	-1.96134000	-0.69396600
N	0.55716700	-0.38120300	-0.26584600
H	-2.37344000	0.46876000	-4.15531000
C	-1.84878400	-0.54824300	-6.00115700
C	-0.98087300	-1.40780400	-6.71540200
C	-2.89224200	0.09539900	-6.70245500
C	-1.16150100	-1.61645000	-8.07925200
H	-0.15272700	-1.90417200	-6.20596700
C	-3.07133400	-0.11773000	-8.06924300
H	-3.56218500	0.76892000	-6.16201100
C	-2.20710400	-0.97434400	-8.76006700
H	-0.48253600	-2.27919500	-8.62062800
H	-3.88331100	0.38635300	-8.59810300
H	-2.34290700	-1.14047100	-9.83148700
C	2.11322300	-1.90687500	-3.14271500
C	1.94172600	-3.25688400	-3.46033900
C	2.64359000	-0.99993700	-4.06543400
C	2.29149800	-3.69996400	-4.73944900
H	1.52043100	-3.94104500	-2.72202100
C	2.98360800	-1.45262700	-5.34233600
H	2.76330600	0.04857100	-3.78846100
C	2.80359800	-2.79912100	-5.68043200
H	2.15397300	-4.75077500	-5.00254900
H	3.38404500	-0.74912300	-6.07523000
H	3.06382100	-3.14818100	-6.68214200

8. SFC traces

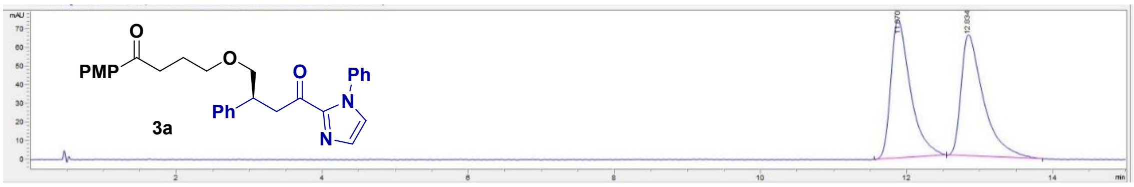

#	Time	Type	Area	Height	Width	Area%	Symmetry
1	11.87	BB	1398	74.7	0.2743	50.047	0.541
2	12.834	BB	1395.4	65.3	0.3114	49.953	0.45

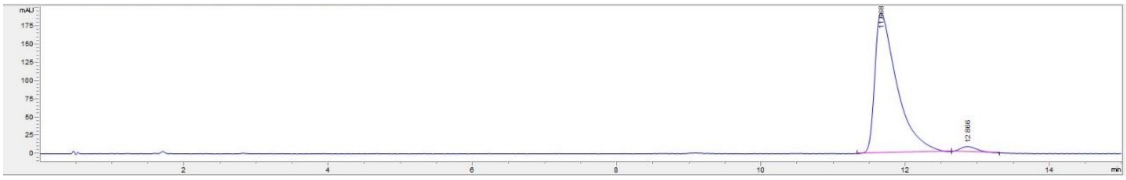

#	Time	Type	Area	Height	Width	Area%	Symmetry
1	11.668	BB	4094	191.3	0.3037	97.055	0.363
2	12.866	BB	124.2	7.5	0.2373	2.945	0.68

Fig. S11. SFC chromatograms for racemic and enantioenriched **3a**.

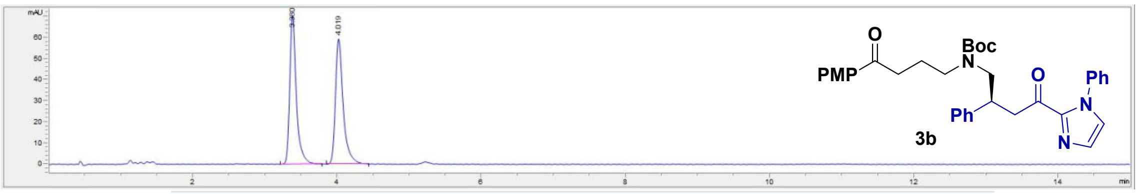

#	Time	Type	Area	Height	Width	Area%	Symmetry
1	3.38	BB	447.4	70.3	0.0962	50.124	0.676
2	4.019	BB	445.2	59.3	0.1136	49.876	0.64

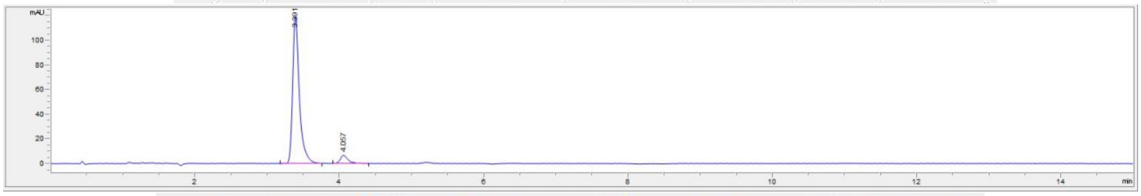

#	Time	Type	Area	Height	Width	Area%	Symmetry
1	3.391	BB	769.9	119.6	0.0971	93.814	0.65
2	4.057	BB	50.8	6.6	0.1116	6.186	0.638

Fig. S12. SFC chromatograms for racemic and enantioenriched **3b**.

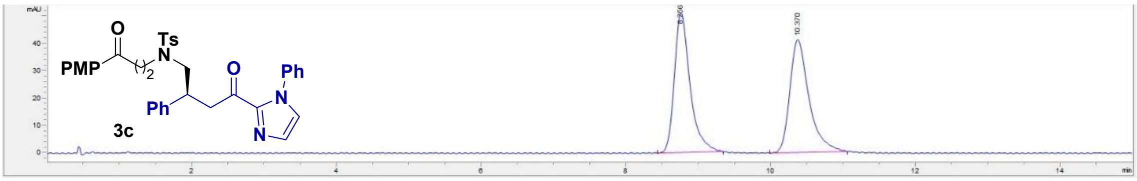

#	Time	Type	Area	Height	Width	Area%	Symmetry
1	8.756	BB	777.2	51.3	0.2269	50.308	0.669
2	10.37	BB	767.6	41.4	0.2762	49.692	0.661

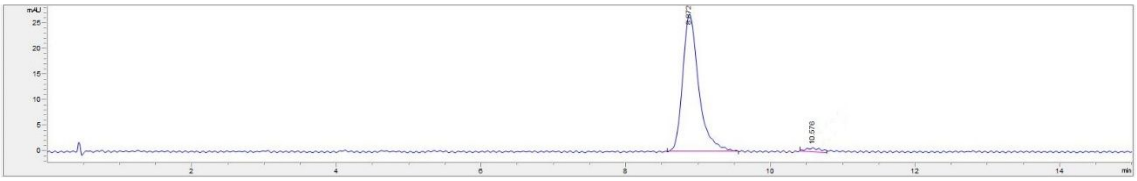

#	Time	Type	Area	Height	Width	Area%	Symmetry
1	8.872	BB	419.6	27.1	0.2369	97.357	0.687
2	10.576	MM	11.4	8.1E-1	0.2339	2.643	0.707

Fig. S13. SFC chromatograms for racemic and enantioenriched **3c**.

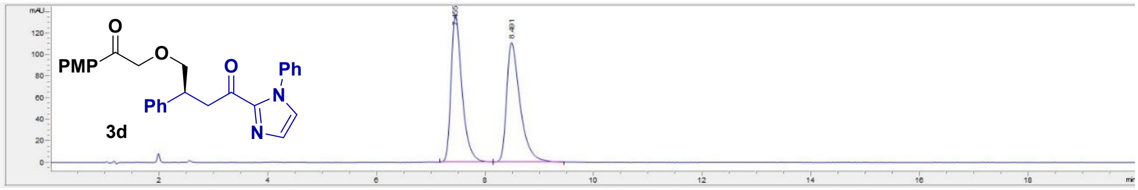

#	Time	Type	Area	Height	Width	Area%	Symmetry
1	7.455	BB	1930.7	137.5	0.2118	50.062	0.618
2	8.491	BB	1925.9	111.4	0.2576	49.938	0.538

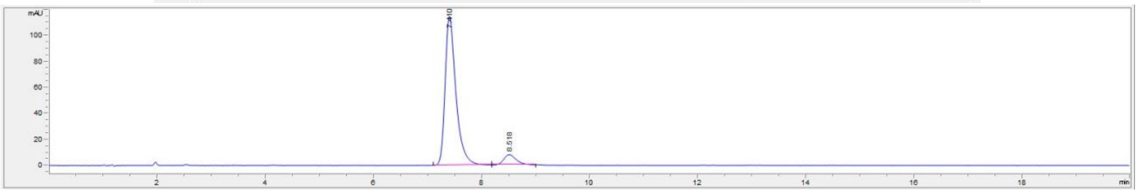

#	Time	Type	Area	Height	Width	Area%	Symmetry
1	7.41	BB	1586.7	114.2	0.2101	92.628	0.623
2	8.518	BB	126.3	7.8	0.2364	7.372	0.757

Fig. S14. SFC chromatograms for racemic and enantioenriched **3d**.

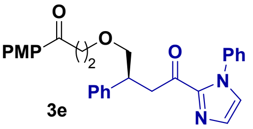

3e

#	Time	Type	Area	Height	Width	Area%	Symmetry
1	13.024	BB	811.8	42.6	0.2905	50.081	0.747
2	13.924	BB	809.2	37.8	0.3176	49.919	0.646

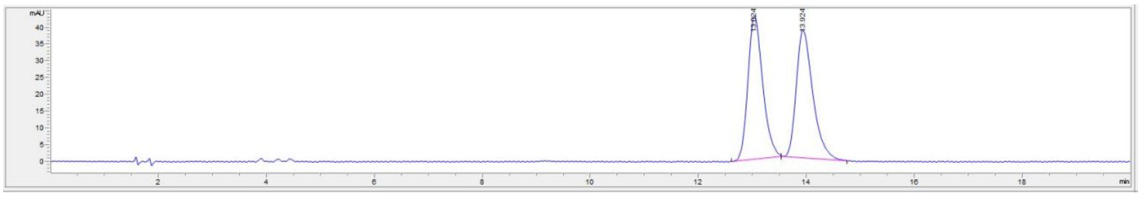

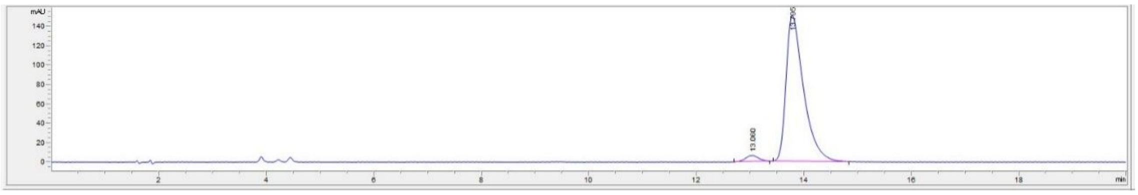

#	Time	Type	Area	Height	Width	Area%	Symmetry
1	13.06	BB	109.9	6.3	0.2175	3.112	1.239
2	13.795	BB	3420.8	150.9	0.3365	96.888	0.52

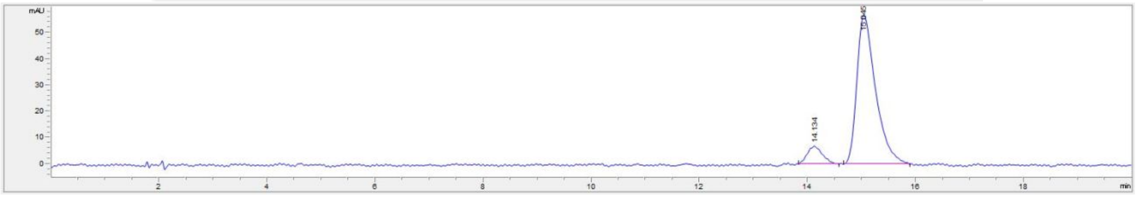

#	Time	Type	Area	Height	Width	Area%	Symmetry
1	14.134	BB	135.3	6.9	0.2453	8.936	0.866
2	15.045	BB	1378.9	57.1	0.3499	91.064	0.554

Fig. S15. SFC chromatograms for racemic and enantioenriched **3e** and for the reaction on 1.0 mmol.

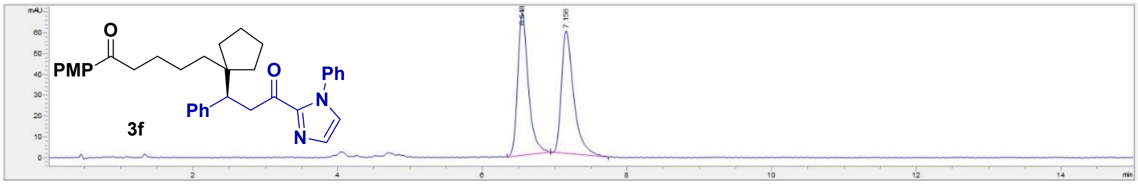

#	Time	Type	Area	Height	Width	Area%	Symmetry
1	6.548	BB	701.7	68.1	0.1562	50.243	0.69
2	7.156	BB	694.9	58.8	0.1779	49.757	0.575

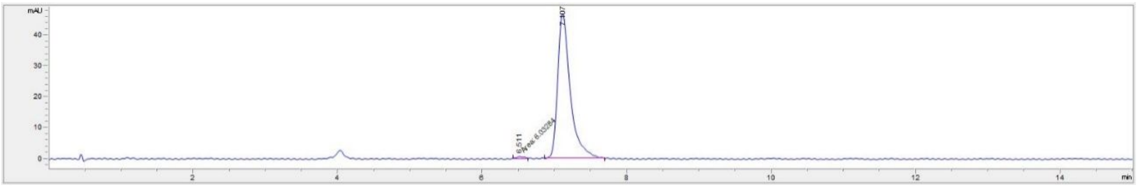

#	Time	Type	Area	Height	Width	Area%	Symmetry
1	6.511	MM	6	8E-1	0.126	1.051	0.771
2	7.107	BB	567.9	46.8	0.1816	98.949	0.585

Fig. S16. SFC chromatograms for racemic and enantioenriched **3f**.

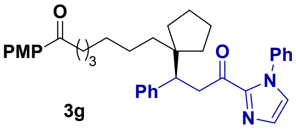

3g

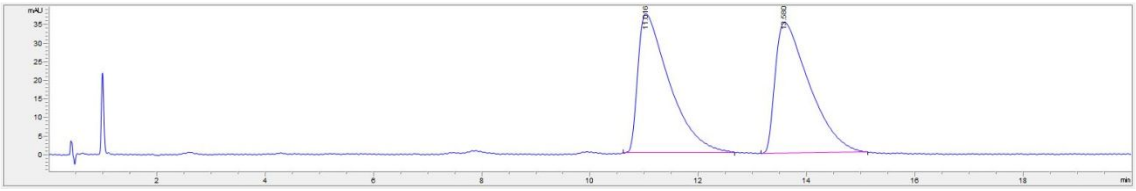

#	Time	Type	Area	Height	Width	Area%	Symmetry
1	11.016	BB	1516.7	37.4	0.543	49.546	0.309
2	13.58	BB	1544.5	35.1	0.5853	50.454	0.349

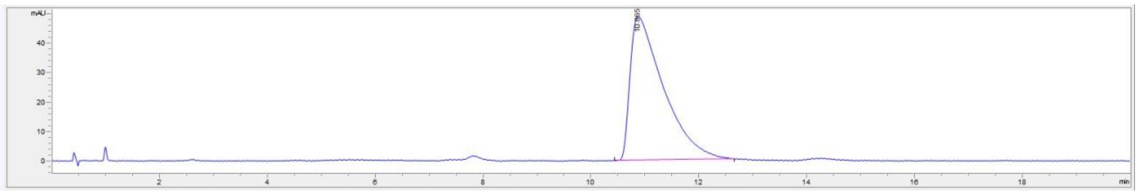

#	Time	Type	Area	Height	Width	Area%	Symmetry
1	10.865	BB	2105.6	48.8	0.5815	100.000	0.299

Fig. S17. SFC chromatograms for racemic and enantioenriched **3g**.

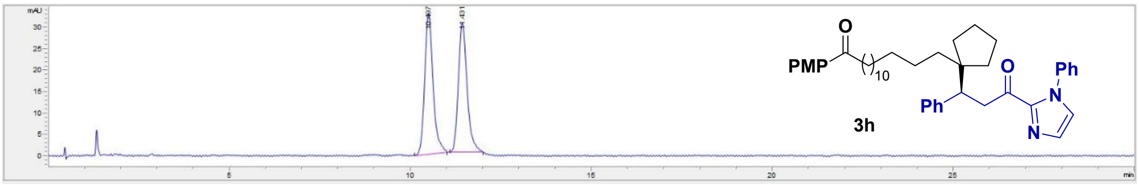

#	Time	Type	Area	Height	Width	Area%	Symmetry
1	10.497	BB	530.4	32.7	0.2491	50.917	0.798
2	11.431	BB	511.3	30.1	0.2503	49.083	0.777

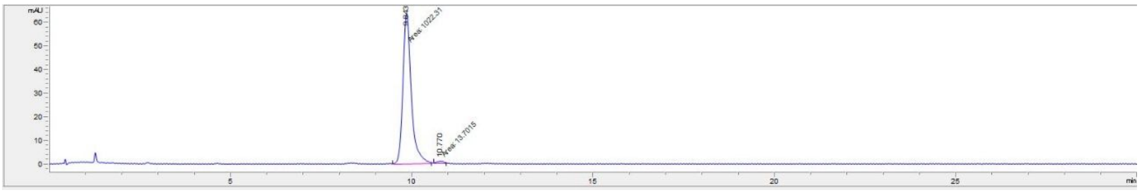

#	Time	Type	Area	Height	Width	Area%	Symmetry
1	9.843	MM	1022.3	63.5	0.2683	98.677	0.753
2	10.77	MM	13.7	1.1	0.2153	1.323	0.909

Fig. S18. SFC chromatograms for racemic and enantioenriched **3h**.

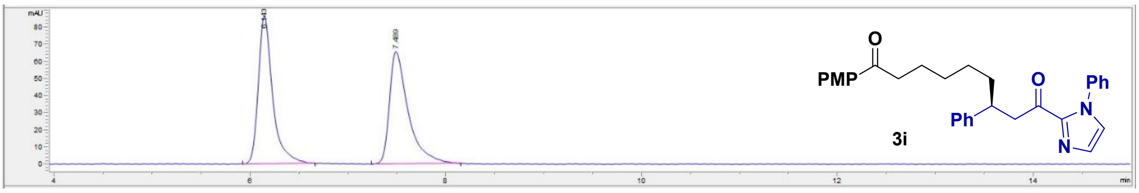

#	Time	Type	Area	Height	Width	Area%	Symmetry
1	6.143	BB	881.2	86.1	0.1514	50.030	0.663
2	7.489	BB	880.1	65.4	0.197	49.970	0.489

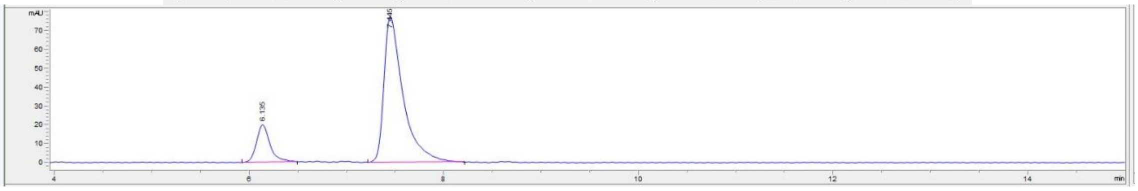

#	Time	Type	Area	Height	Width	Area%	Symmetry
1	6.135	BB	193.7	19.9	0.1477	15.518	0.771
2	7.445	BB	1054.4	77.2	0.1994	84.482	0.461

Fig. S19. SFC chromatograms for racemic and enantioenriched **3i**.

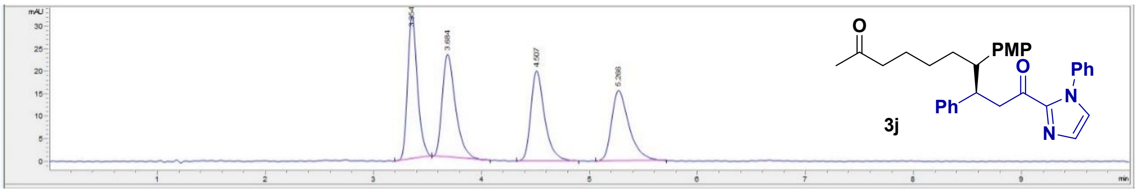

#	Time	Type	Area	Height	Width	Area%	Symmetry
1	3.354	BB	203.1	32	0.098	26.619	0.76
2	3.684	BB	198.5	23.1	0.1304	26.027	0.64
3	4.507	BB	180.9	20.2	0.1366	23.715	0.672
4	5.266	BB	180.3	15.8	0.1689	23.638	0.627

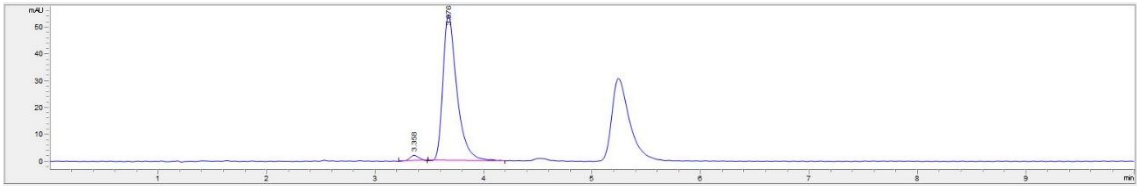

#	Time	Type	Area	Height	Width	Area%	Symmetry
1	3.358	BB	12.9	2.1	0.091	2.563	0.842
2	3.676	BB	490.1	54.8	0.1343	97.437	0.605

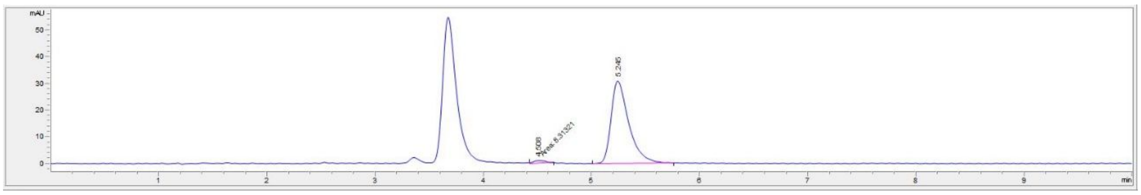

#	Time	Type	Area	Height	Width	Area%	Symmetry
1	4.508	MM	8.3	1	0.1355	2.298	0.62
2	5.245	BB	353.4	31.1	0.1708	97.702	0.562

Fig. S20. SFC chromatograms for racemic and enantioenriched **3j**.

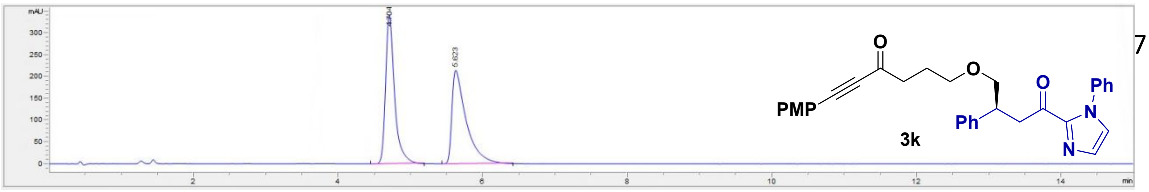

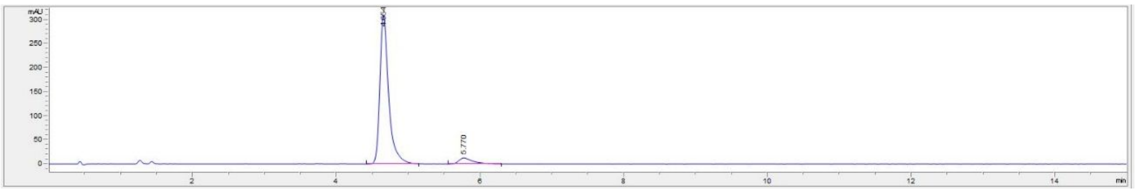

#	Time	Type	Area	Height	Width	Area%	Symmetry
1	4.654	BB	2626.3	309.7	0.1269	94.019	0.639
2	5.77	BB	167.1	12.4	0.1877	5.981	0.505

Fig. S21. SFC chromatograms for racemic and enantioenriched **3k**.

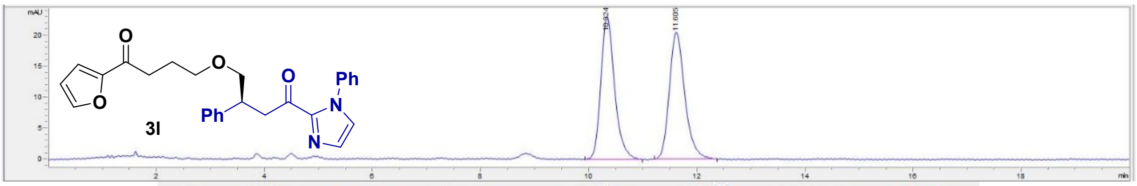

#	Time	Type	Area	Height	Width	Area%	Symmetry
1	10.324	BB	413.7	23.3	0.2696	50.145	0.759
2	11.605	BB	411.4	20.6	0.297	49.855	0.758

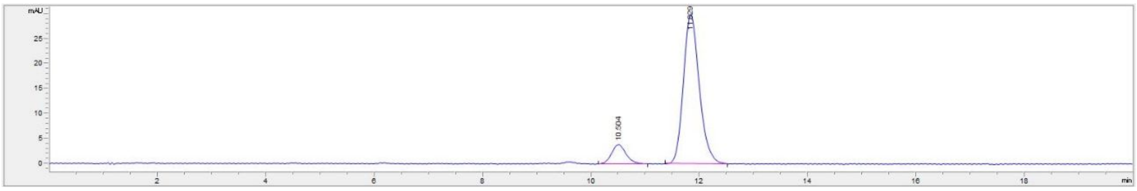

#	Time	Type	Area	Height	Width	Area%	Symmetry
1	10.504	BB	72	3.9	0.2582	10.547	0.932
2	11.829	BB	610.8	30	0.3079	89.453	0.808

Fig. S22. SFC chromatograms for racemic and enantioenriched **3l**.

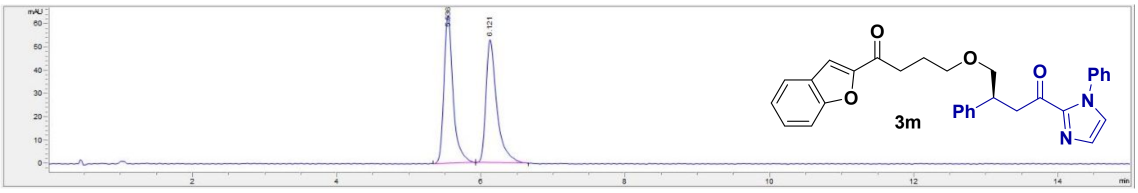

#	Time	Type	Area	Height	Width	Area%	Symmetry
1	5.536	BB	561	63.7	0.1328	50.064	0.716
2	6.121	BB	559.6	52.9	0.1574	49.936	0.565

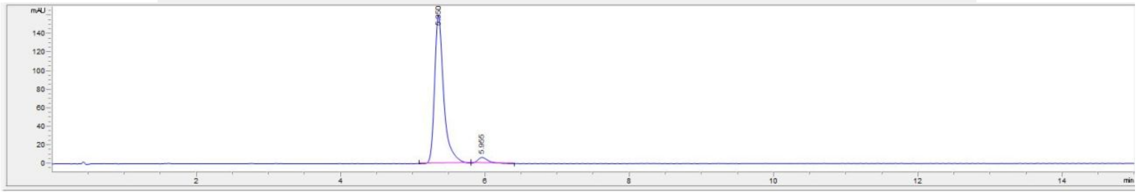

#	Time	Type	Area	Height	Width	Area%	Symmetry
1	5.35	BB	1415.6	161.2	0.1304	95.714	0.656
2	5.955	BB	63.4	6.3	0.1445	4.286	0.631

Fig. S23. SFC chromatograms for racemic and enantioenriched **3m**.

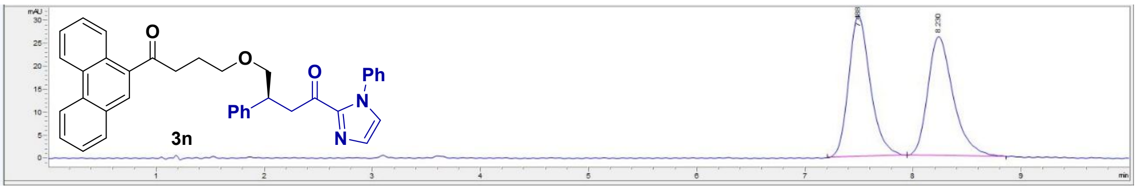

#	Time	Type	Area	Height	Width	Area%	Symmetry
1	7.488	BB	430.3	30.8	0.2088	50.242	0.784
2	8.23	BB	426.1	26	0.249	49.758	0.693

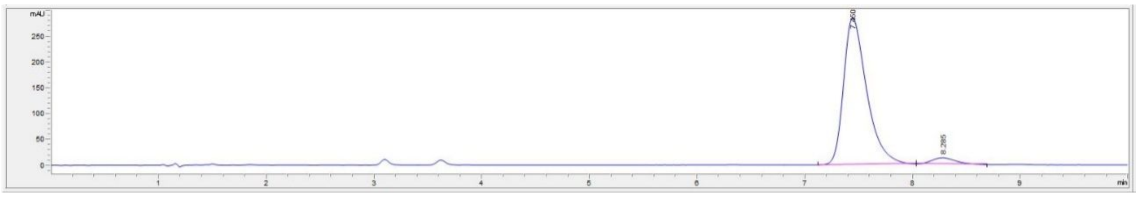

#	Time	Type	Area	Height	Width	Area%	Symmetry
1	7.45	BB	4248.5	284.8	0.226	95.712	0.616
2	8.285	BB	190.3	12.2	0.234	4.288	0.753

Fig. S24. SFC chromatograms for racemic and enantioenriched **3n**.

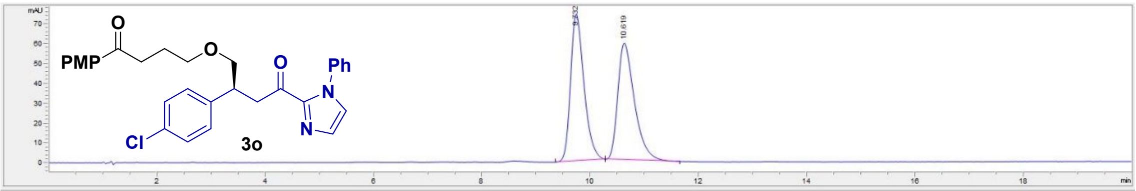

#	Time	Type	Area	Height	Width	Area%	Symmetry
1	9.732	BB	1272.4	73.8	0.2612	50.300	0.683
2	10.619	BB	1257.2	58.7	0.3258	49.700	0.595

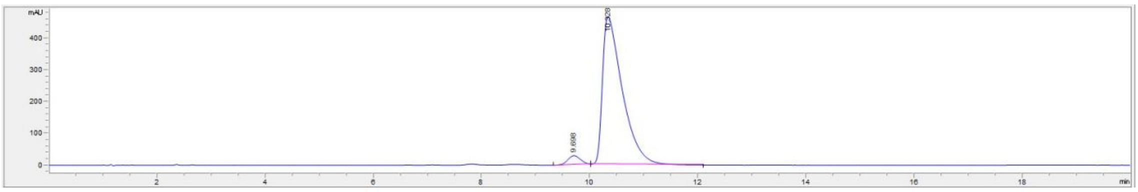

#	Time	Type	Area	Height	Width	Area%	Symmetry
1	9.698	BB	455.3	28.1	0.2531	3.710	0.907
2	10.328	BB	11816.1	465.9	0.3819	96.290	0.354

Fig. S25. SFC chromatograms for racemic and enantioenriched **3o**.

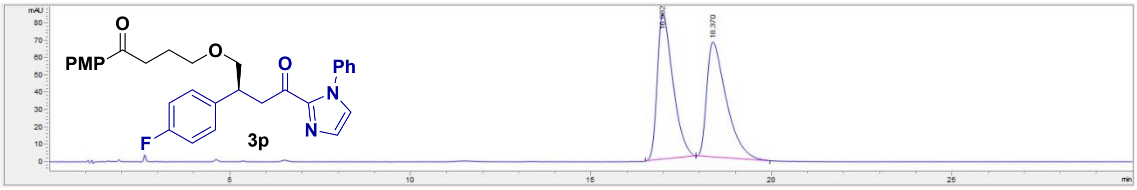

#	Time	Type	Area	Height	Width	Area%	Symmetry
1	16.982	BB	2531.8	83.9	0.4541	50.332	0.533
2	18.37	BB	2498.3	66.3	0.5499	49.668	0.442

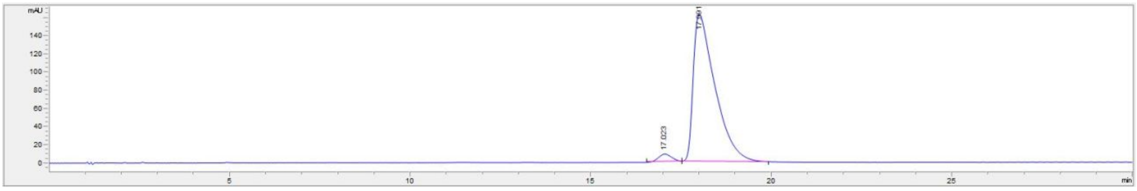

#	Time	Type	Area	Height	Width	Area%	Symmetry
1	17.023	BB	210.8	8.5	0.313	3.025	0.826
2	17.991	BB	6756.8	161.7	0.5916	96.975	0.359

Fig. S26. SFC chromatograms for racemic and enantioenriched **3p**.

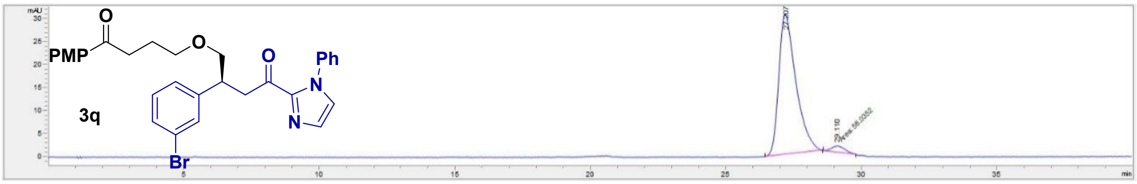

#	Time	Type	Area	Height	Width	Area%	Symmetry
1	27.352	BB	631.7	15.6	0.4953	50.077	0.692
2	29.012	BB	629.8	14	0.5515	49.923	0.576

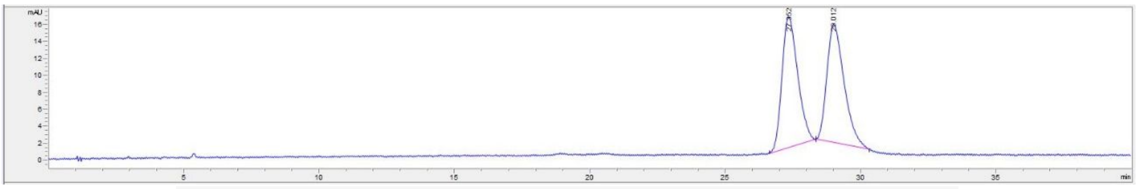

#	Time	Type	Area	Height	Width	Area%	Symmetry
1	27.207	BB	1347.4	30.5	0.5869	95.871	0.568
2	29.11	MM	58	1.6	0.6219	4.129	0.673

Fig. S27. SFC chromatograms for racemic and enantioenriched **3q**.

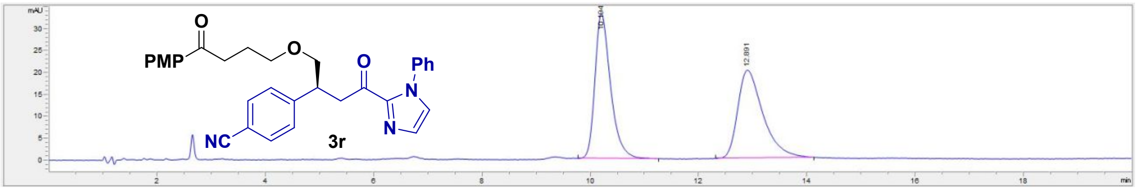

#	Time	Type	Area	Height	Width	Area%	Symmetry
1	10.194	BB	663.4	33	0.3006	50.475	0.66
2	12.891	BB	650.9	20	0.4511	49.525	0.595

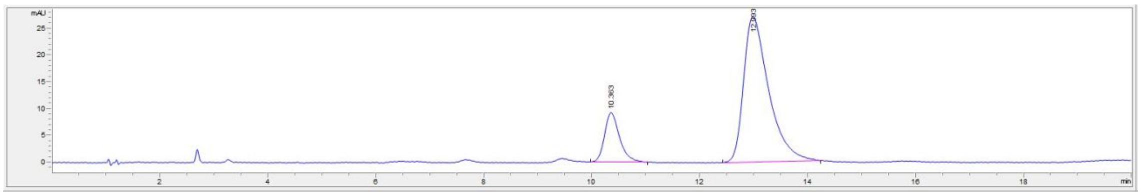

#	Time	Type	Area	Height	Width	Area%	Symmetry
1	10.363	BB	178.2	9.2	0.2876	16.920	0.711
2	12.993	BB	875.1	27.1	0.4567	83.080	0.555

Fig. S28. SFC chromatograms for racemic and enantioenriched **3r**.

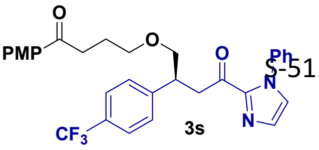

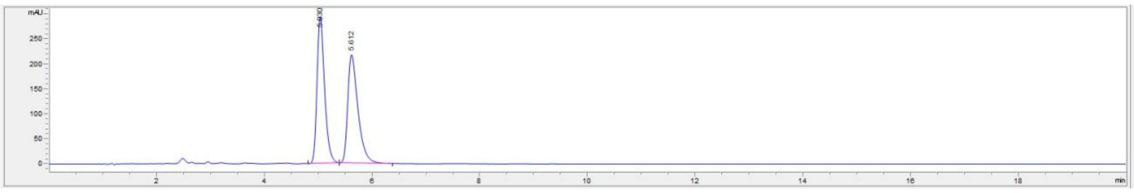

#	Time	Type	Area	Height	Width	Area%	Symmetry
1	5.03	BB	2881.6	295.3	0.1479	50.165	0.682
2	5.612	BB	2862.6	217.9	0.1972	49.835	0.553

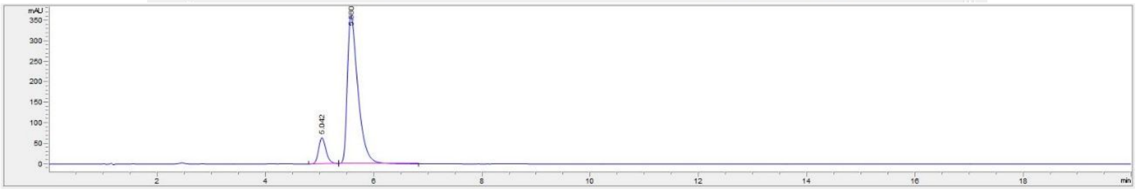

#	Time	Type	Area	Height	Width	Area%	Symmetry
1	5.042	BB	614.3	64.2	0.1455	11.024	0.789
2	5.58	BB	4957.7	364.1	0.2028	88.976	0.484

Fig. S29. SFC chromatograms for racemic and enantioenriched **3s**.

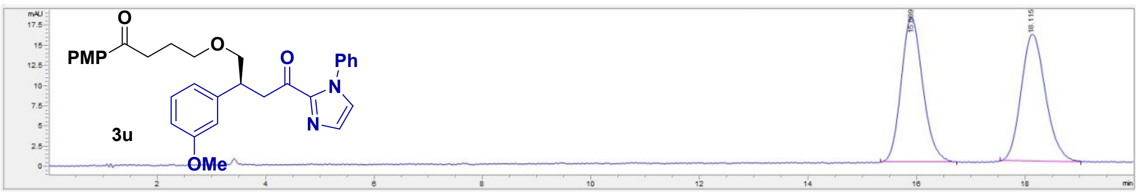

#	Time	Type	Area	Height	Width	Area%	Symmetry
1	15.889	BB	496.1	17.9	0.4329	50.335	0.838
2	18.115	BB	489.5	15.6	0.4311	49.665	0.793

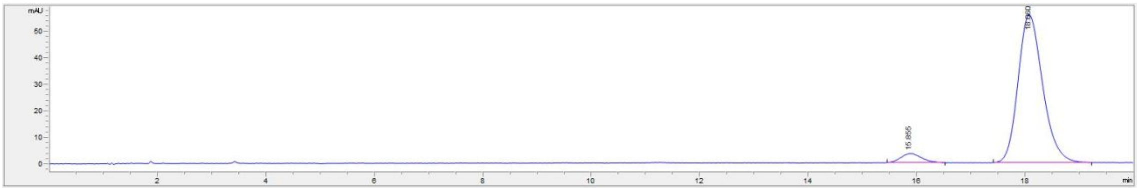

#	Time	Type	Area	Height	Width	Area%	Symmetry
1	15.855	BB	91.2	3.4	0.3263	4.923	0.678
2	18.06	BB	1762	56	0.4814	95.077	0.782

Fig. S30. SFC chromatograms for racemic and enantioenriched **3u**.

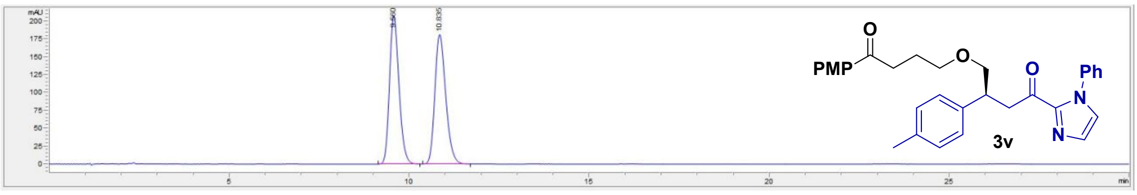

#	Time	Type	Area	Height	Width	Area%	Symmetry
1	9.56	BB	3798.4	207.6	0.2815	49.925	0.773
2	10.835	BB	3809.8	181	0.3256	50.075	0.753

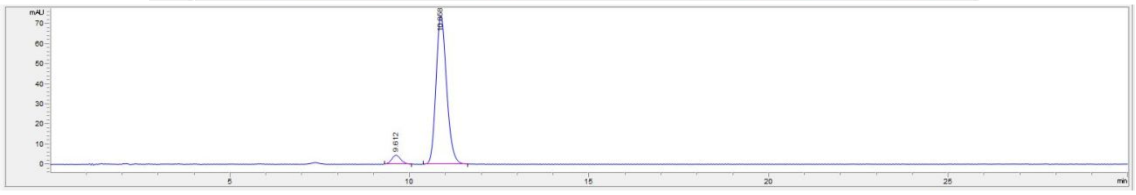

#	Time	Type	Area	Height	Width	Area%	Symmetry
1	9.612	BB	76.5	4.4	0.2465	4.827	0.863
2	10.858	BB	1509.4	73.8	0.3147	95.173	0.803

Fig. S31. SFC chromatograms for racemic and enantioenriched **3v**.

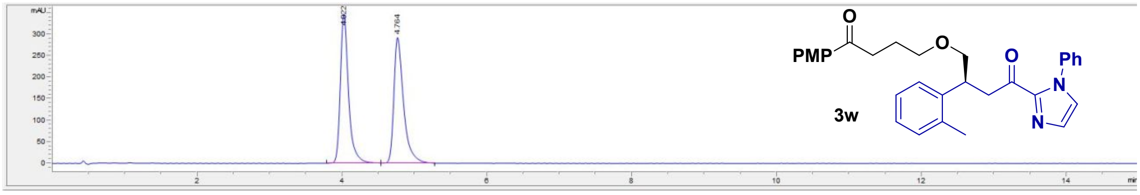

#	Time	Type	Area	Height	Width	Area%	Symmetry
1	4.022	BB	2720.4	346.8	0.1175	50.136	0.69
2	4.764	BB	2705.7	291.8	0.1382	49.864	0.57

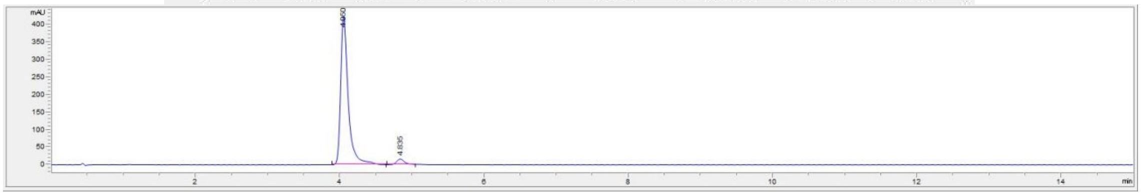

#	Time	Type	Area	Height	Width	Area%	Symmetry
1	4.05	BB	3067	422.7	0.1067	96.447	0.578
2	4.835	BB	113	15.5	0.1111	3.553	0.85

Fig. S32. SFC chromatograms for racemic and enantioenriched **3w**.

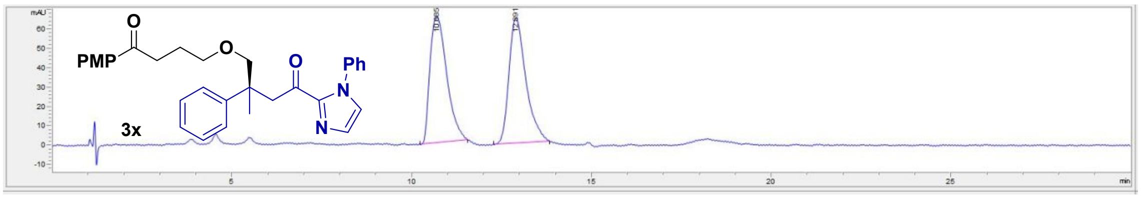

#	Time	Type	Area	Height	Width	Area%	Symmetry
1	10.685	BB	2135.1	65.6	0.4498	49.947	0.62
2	12.891	BB	2139.7	64.9	0.4628	50.053	0.672

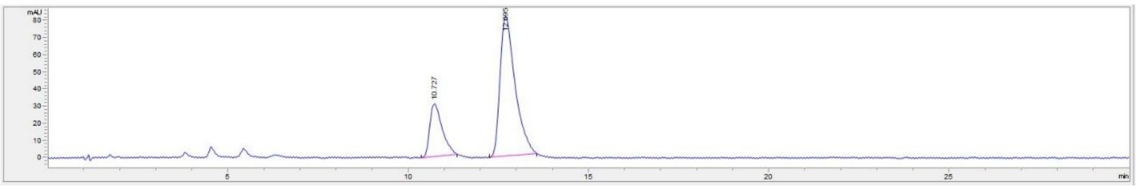

#	Time	Type	Area	Height	Width	Area%	Symmetry
1	10.727	BB	749.3	31.1	0.2975	23.750	0.63
2	12.695	BB	2405.6	82.2	0.4134	76.250	0.555

Fig. S33. SFC chromatograms for racemic and enantioenriched **3x**.

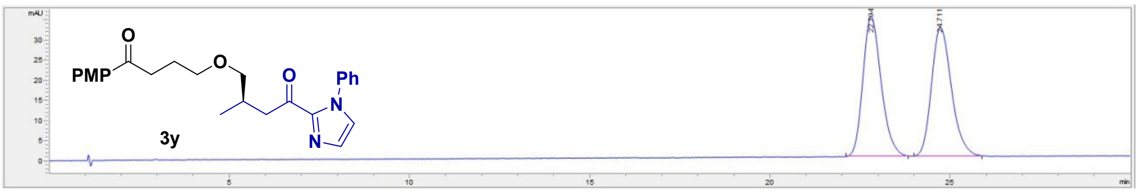

#	Time	Type	Area	Height	Width	Area%	Symmetry
1	22.794	BB	1223.6	34.6	0.5268	49.881	0.782
2	24.711	BB	1229.4	32	0.5626	50.119	0.757

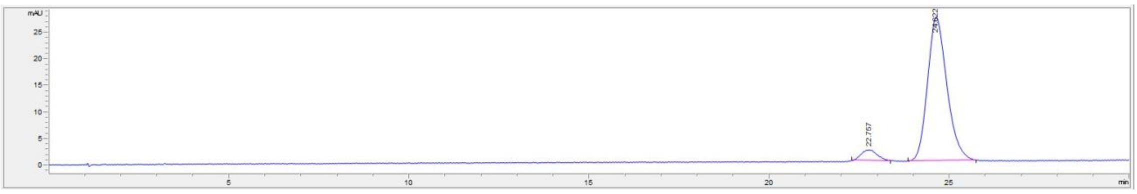

#	Time	Type	Area	Height	Width	Area%	Symmetry
1	22.757	BB	62.4	2	0.3734	5.796	0.935
2	24.622	BB	1014.1	27.1	0.5247	94.204	0.787

Fig. S34. SFC chromatograms for racemic and enantioenriched **3y**.

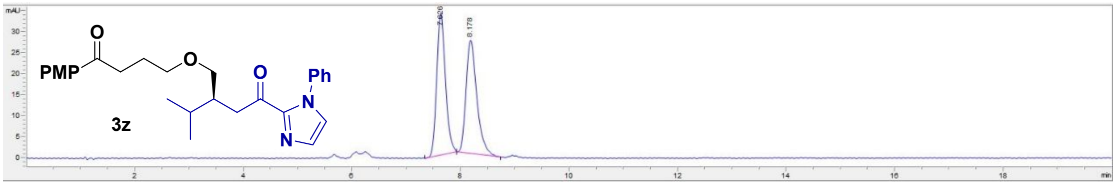

#	Time	Type	Area	Height	Width	Area%	Symmetry
1	7.626	BB	377.6	33.6	0.1731	50.238	0.814
2	8.178	BB	374	26.9	0.2085	49.762	0.695

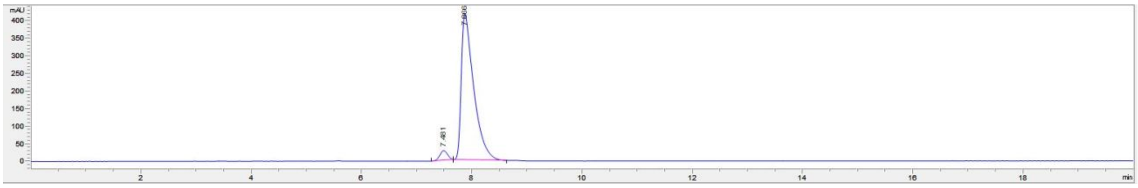

#	Time	Type	Area	Height	Width	Area%	Symmetry
1	7.481	BB	268.6	27.5	0.1539	3.970	0.961
2	7.866	BB	6497.9	415.6	0.2264	96.030	0.383

Fig. S35. SFC chromatograms for racemic and enantioenriched **3z**.

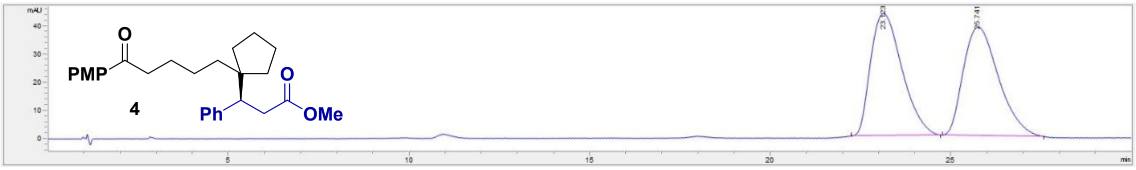

#	Time	Type	Area	Height	Width	Area%	Symmetry
1	23.123	BB	2637.3	43.5	0.7218	50.032	0.646
2	25.741	BB	2634	38.7	0.8085	49.968	0.668

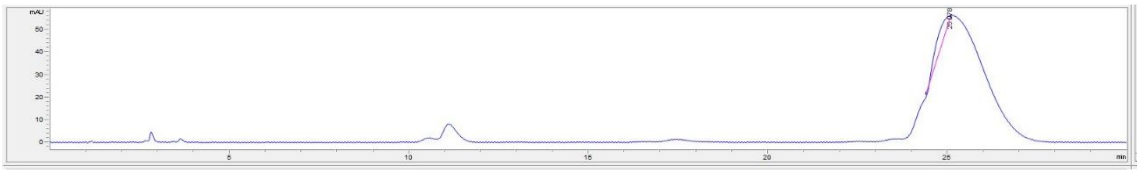

#	Time	Type	Area	Height	Width	Area%	Symmetry
1	25.078	BB	265.1	2.1	1.5214	100.000	115.923

Fig. S36. SFC chromatograms for racemic and enantioenriched **4**.

9. NMR Spectra

^{1}H NMR of **SI1** (300 MHz, $CDCl_3$)

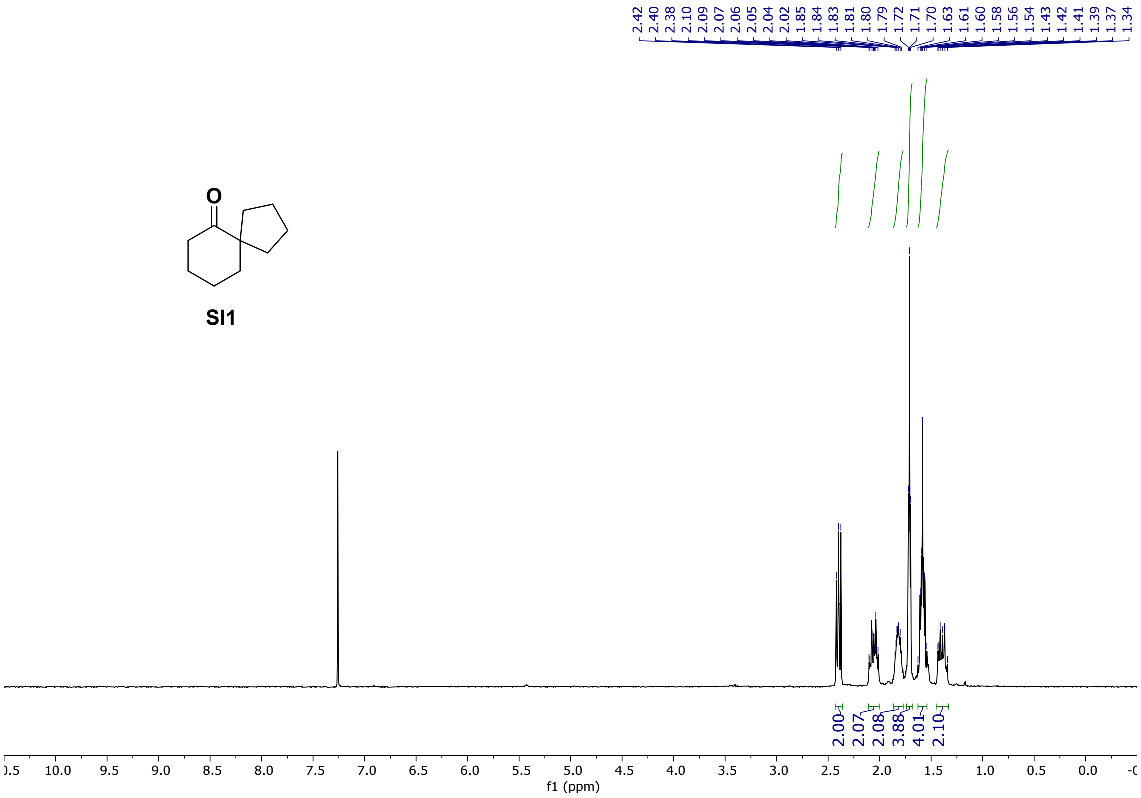

^{1}H NMR of **SI2** (300 MHz, $CDCl_3$)

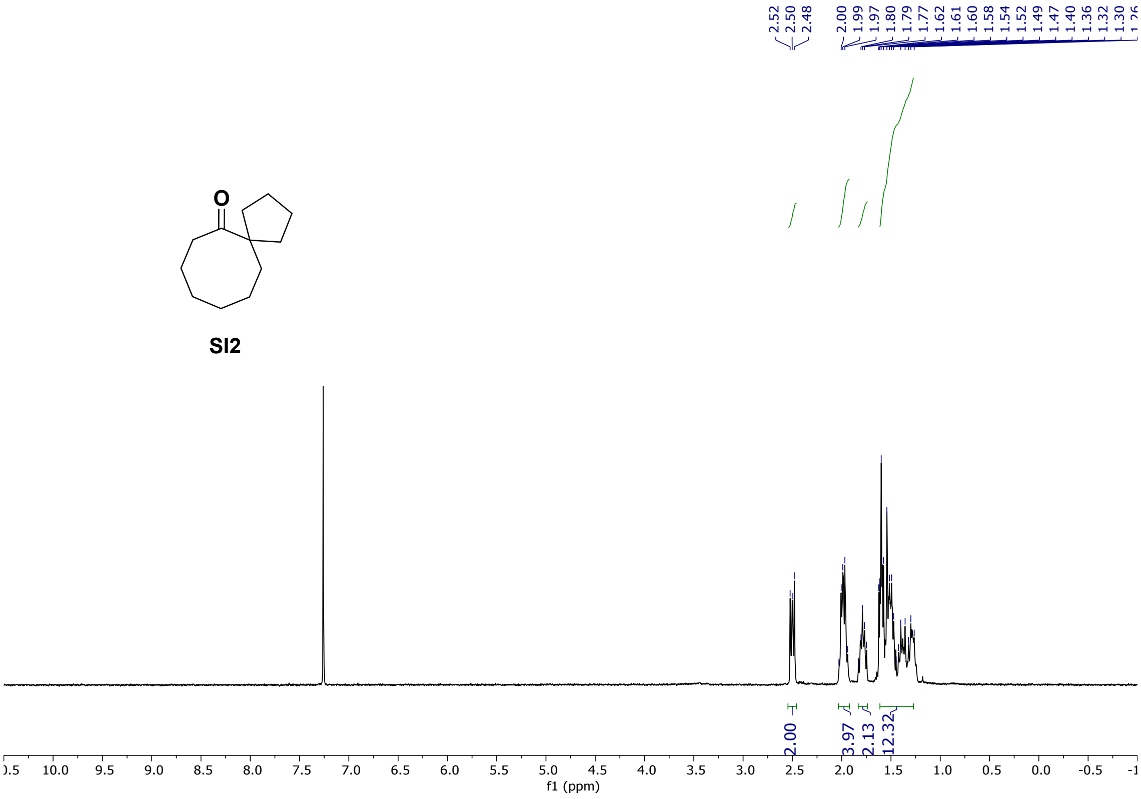

^{13}C NMR of **SI2** (75 MHz, $CDCl_3$)

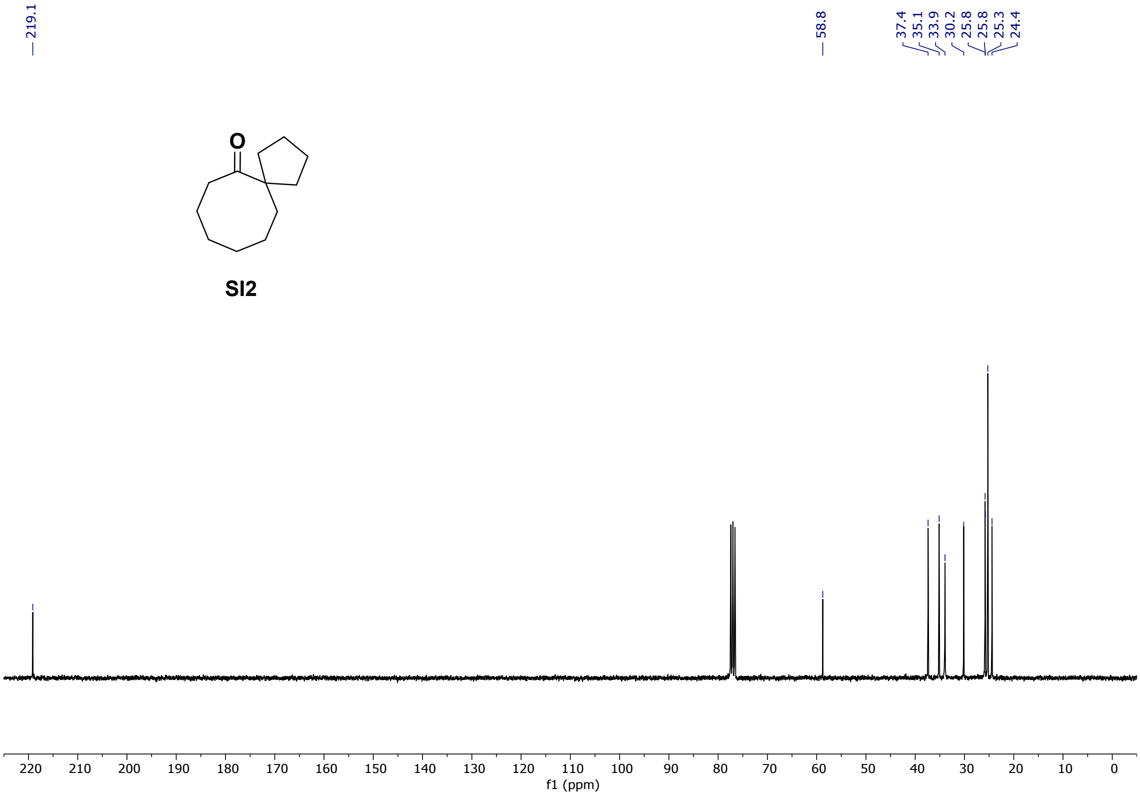

^{1}H NMR of **SI3** (300 MHz, $CDCl_3$)

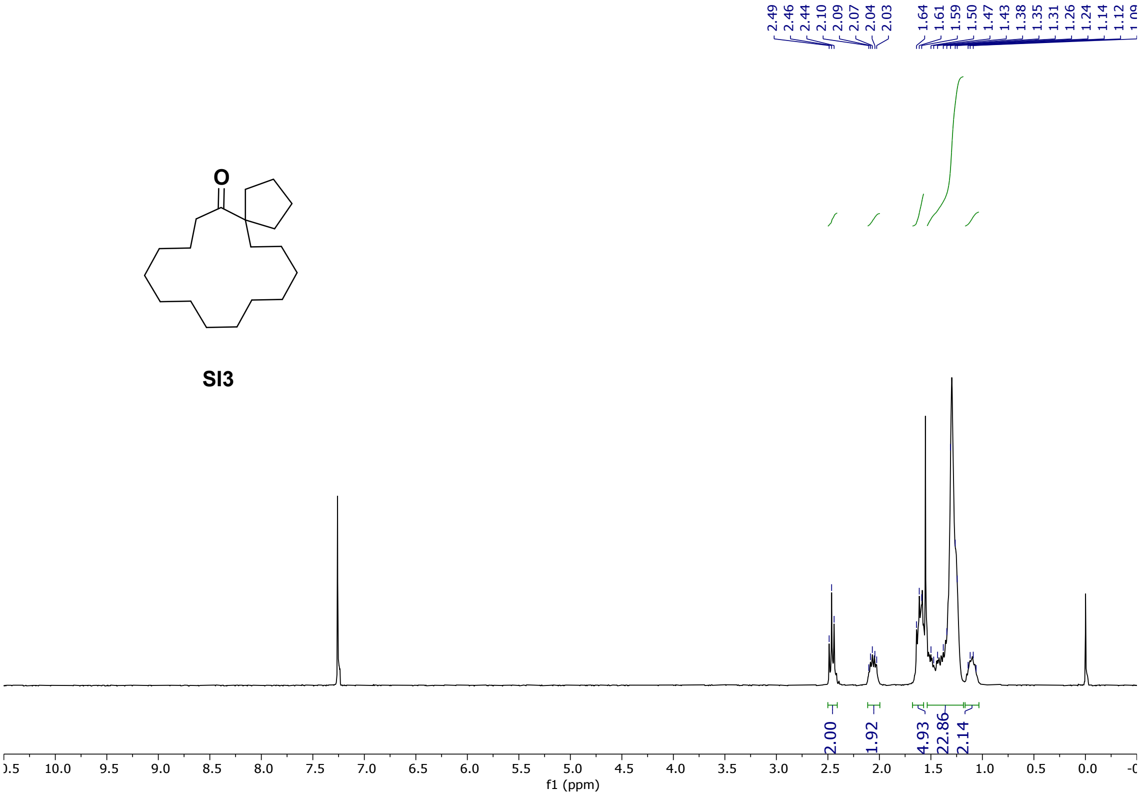

^{13}C NMR of **SI3** (75 MHz, $CDCl_3$)

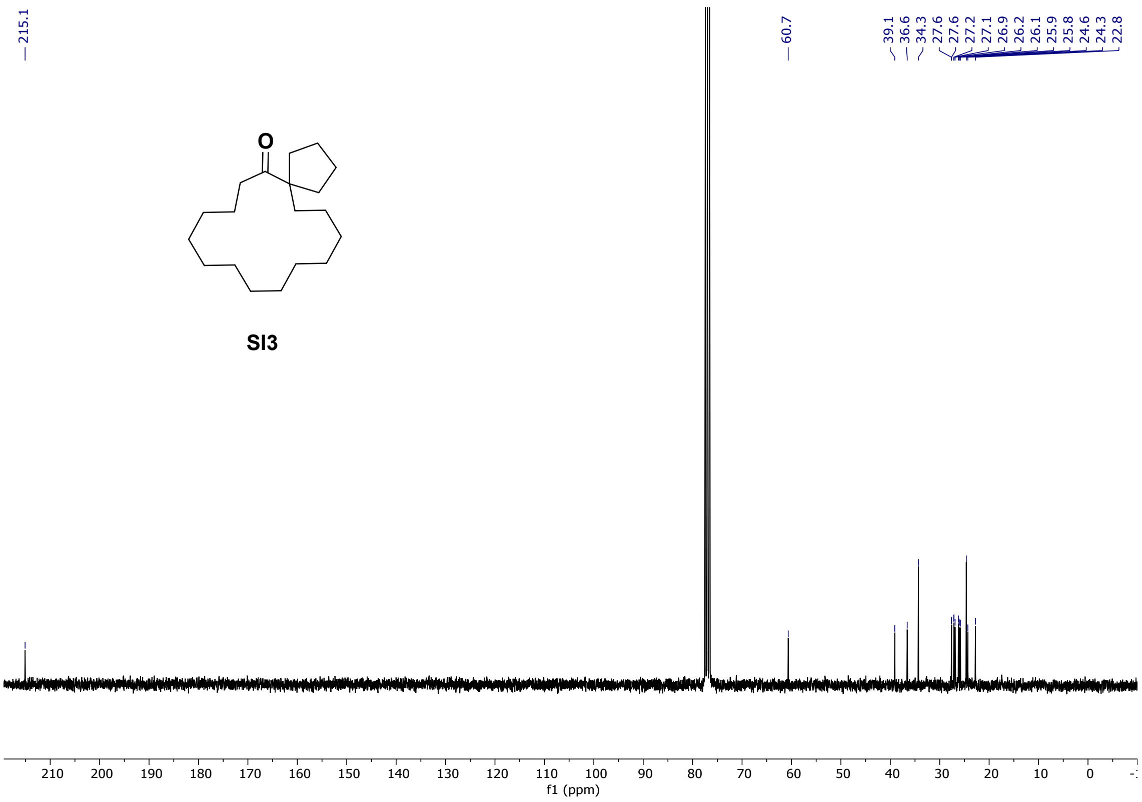

^1H NMR of **1a** (300 MHz, $CDCl_3$)

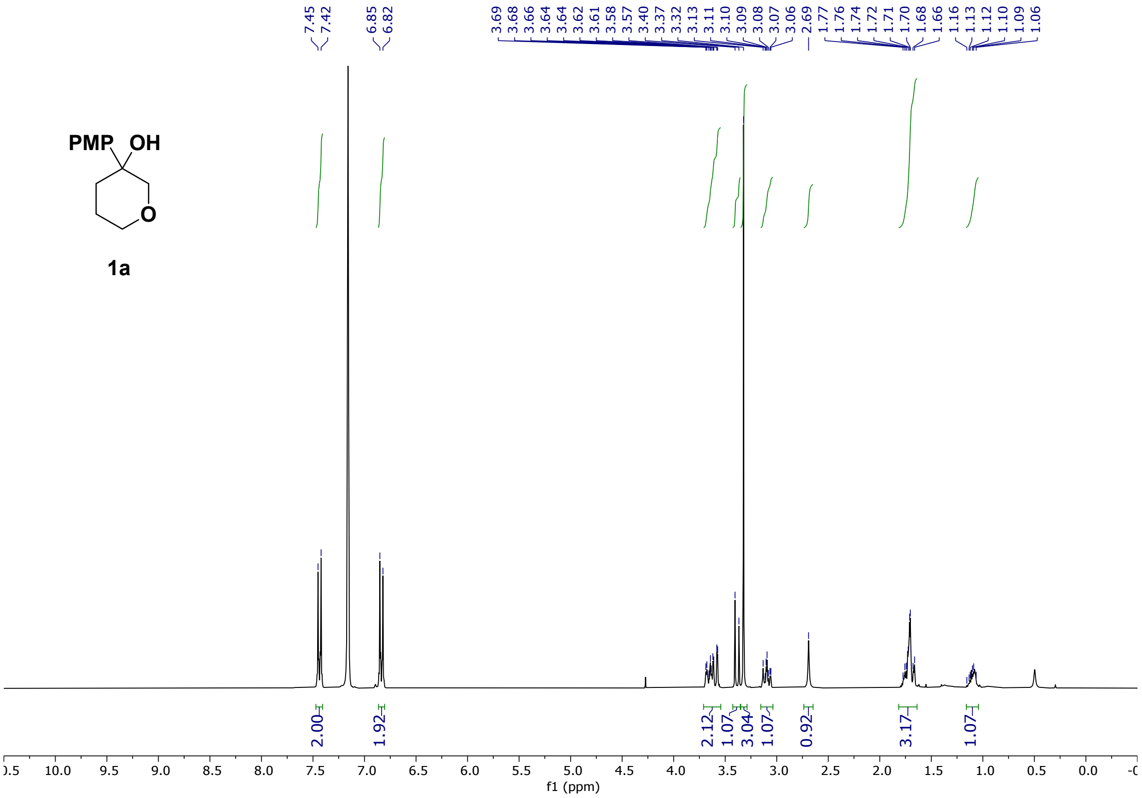

^{1}H NMR of **1b** (300 MHz, $CDCl_3$)

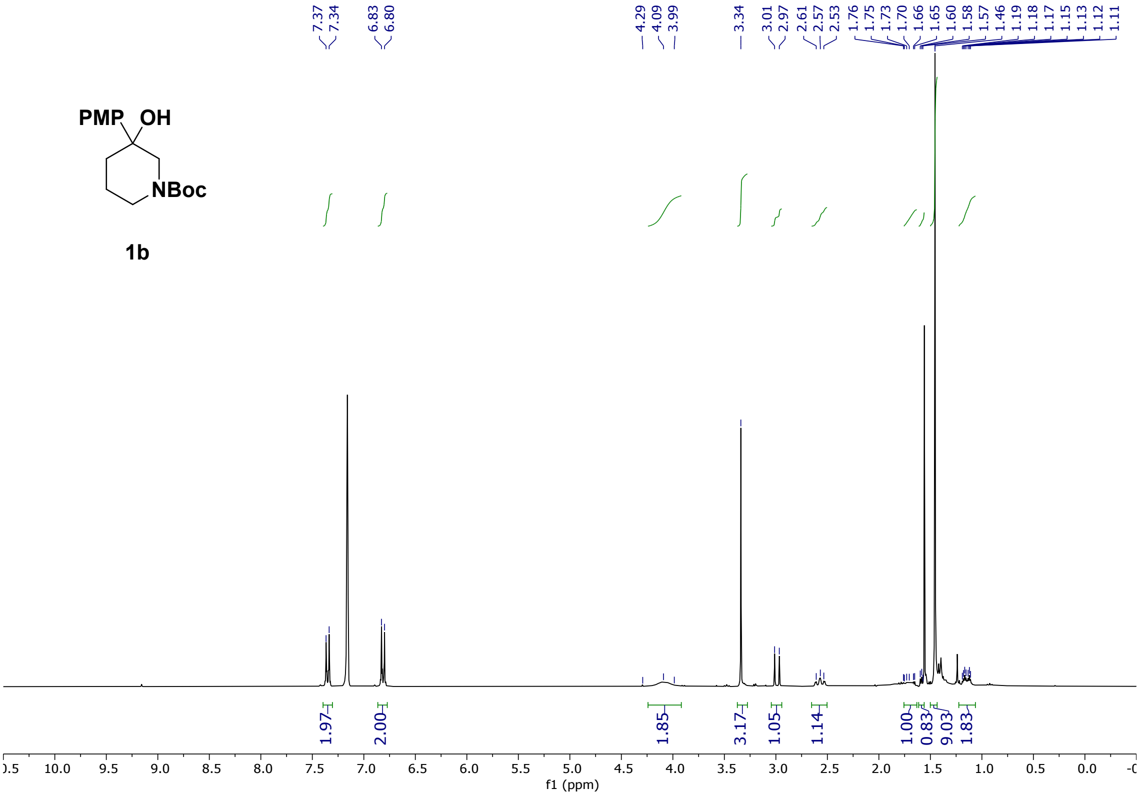

^{1}H NMR of **1c** (300 MHz, $CDCl_3$)

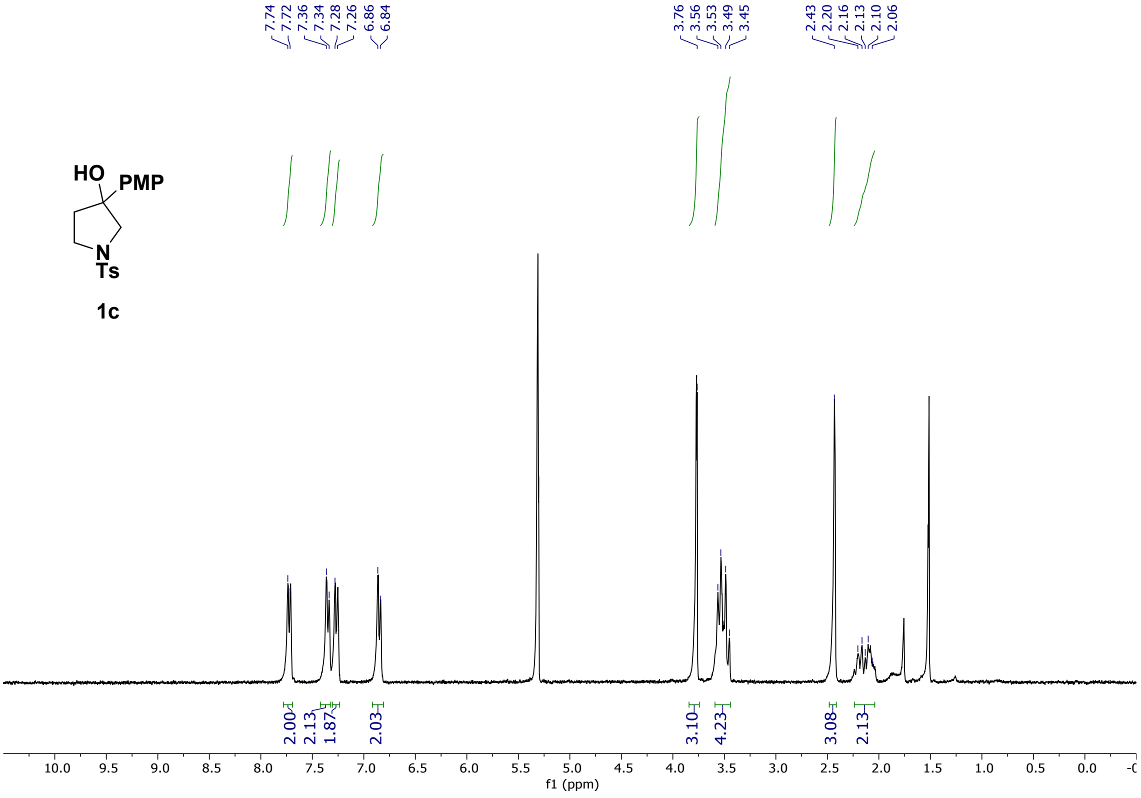

^{13}C NMR of **1c** (75 MHz, $CDCl_3$)

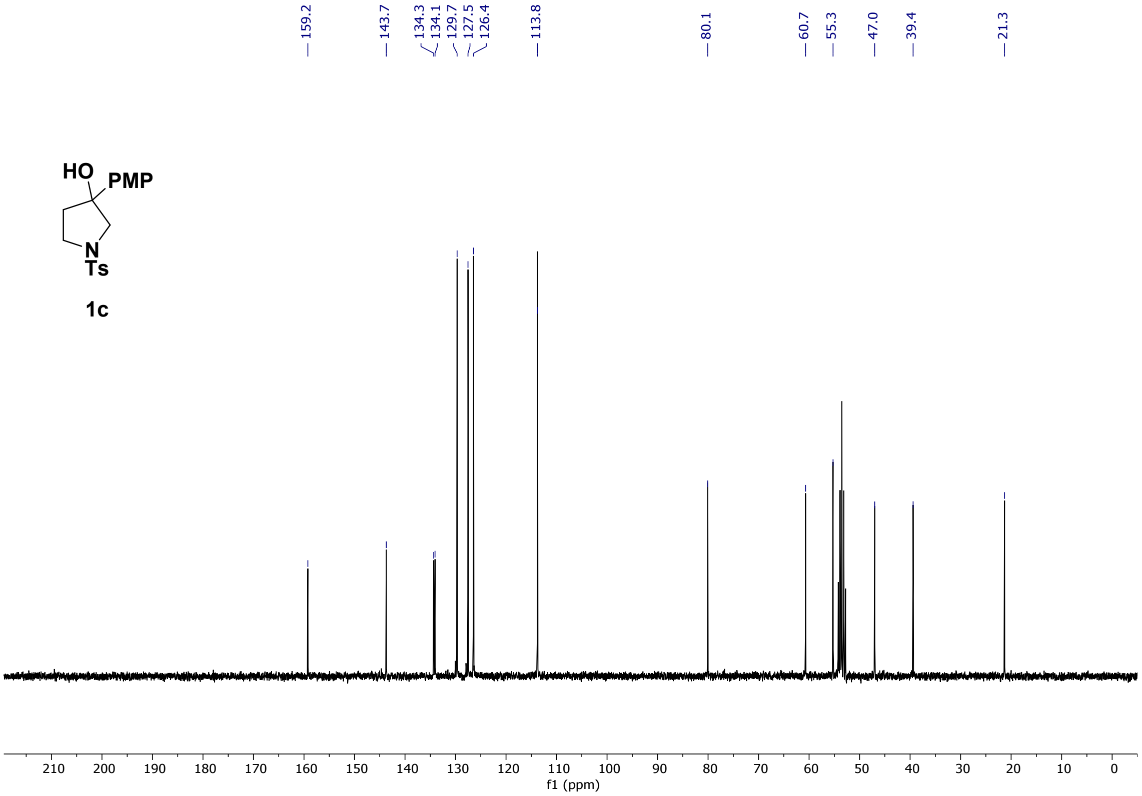

1c

^{1}H NMR of **1d** (300 MHz, $CDCl_3$)

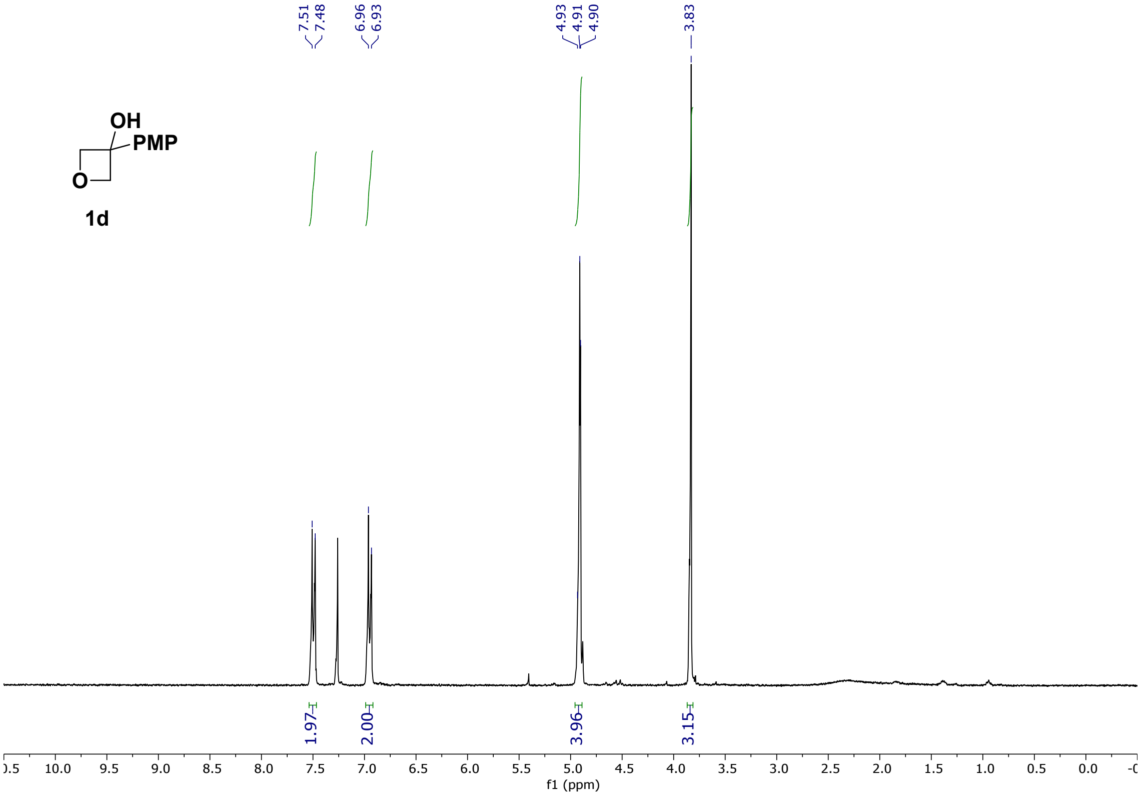

1d

^{1}H NMR of **1e** (300 MHz, $CDCl_3$)

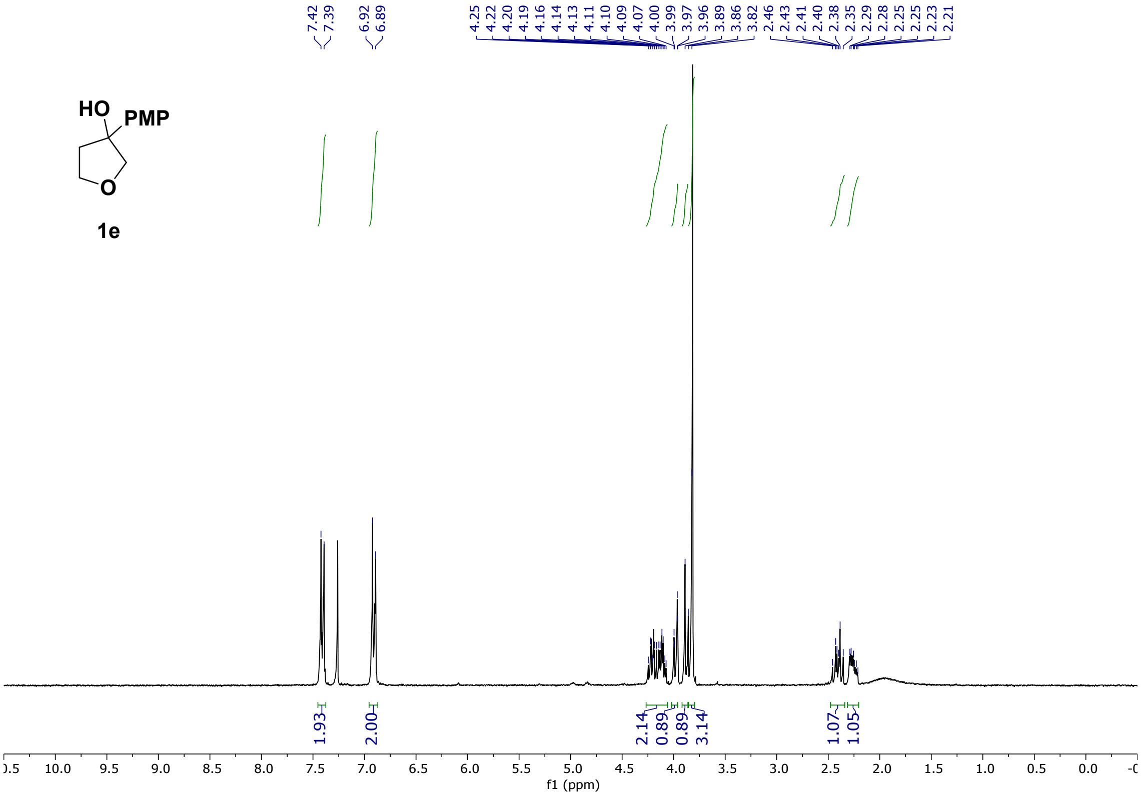

^{1}H NMR of **1f** (300 MHz, CD_2Cl_2)

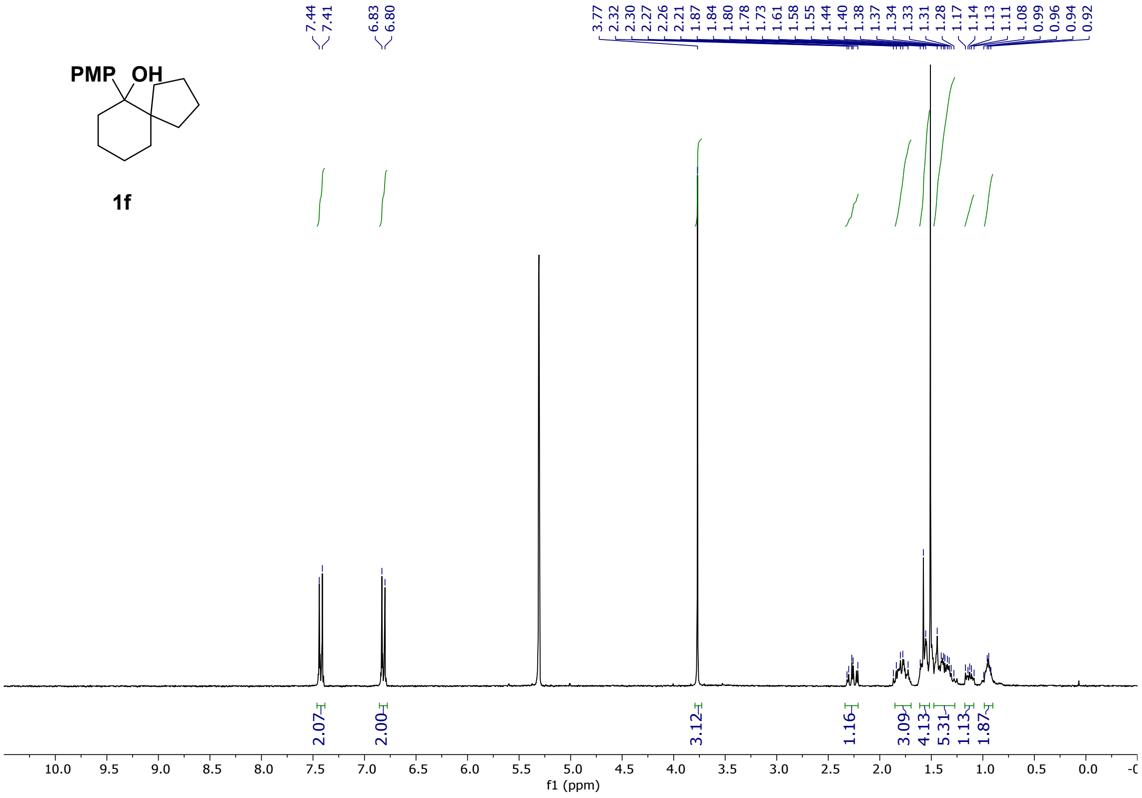

^{13}C NMR of **1f** (75 MHz, CD_2Cl_2)

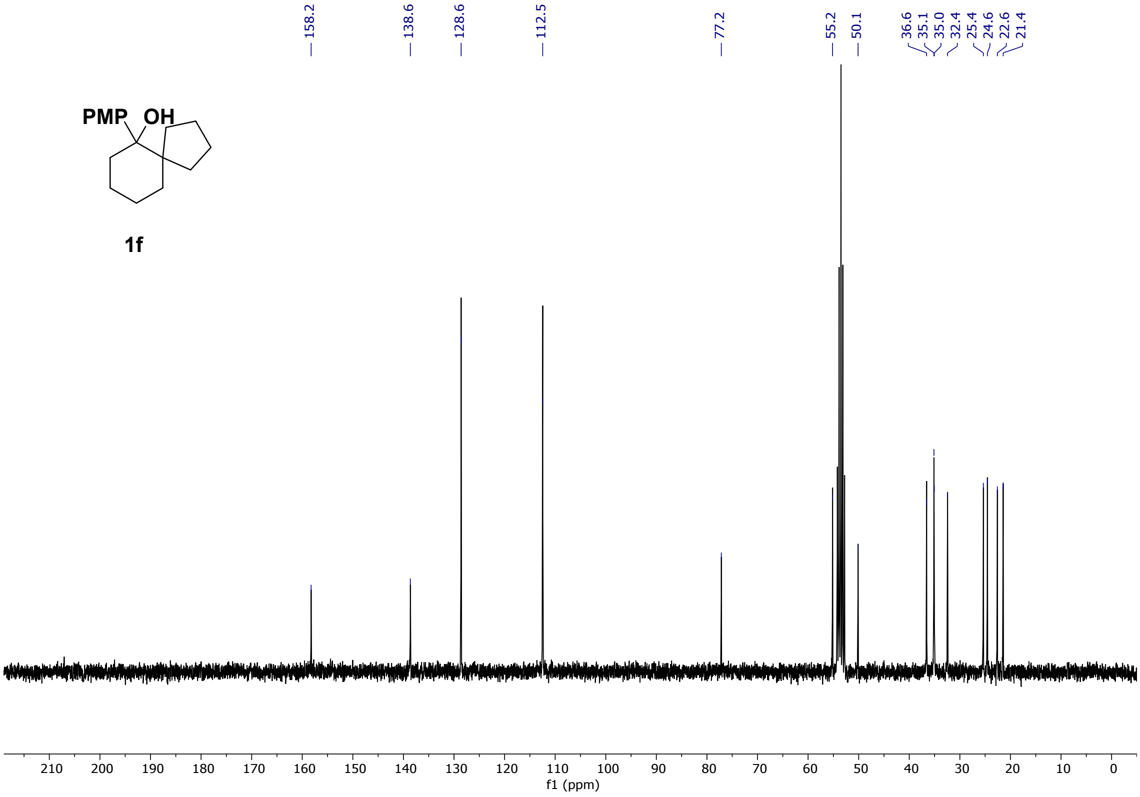

^{1}H NMR of **1g** (300 MHz, CD_2Cl_2)

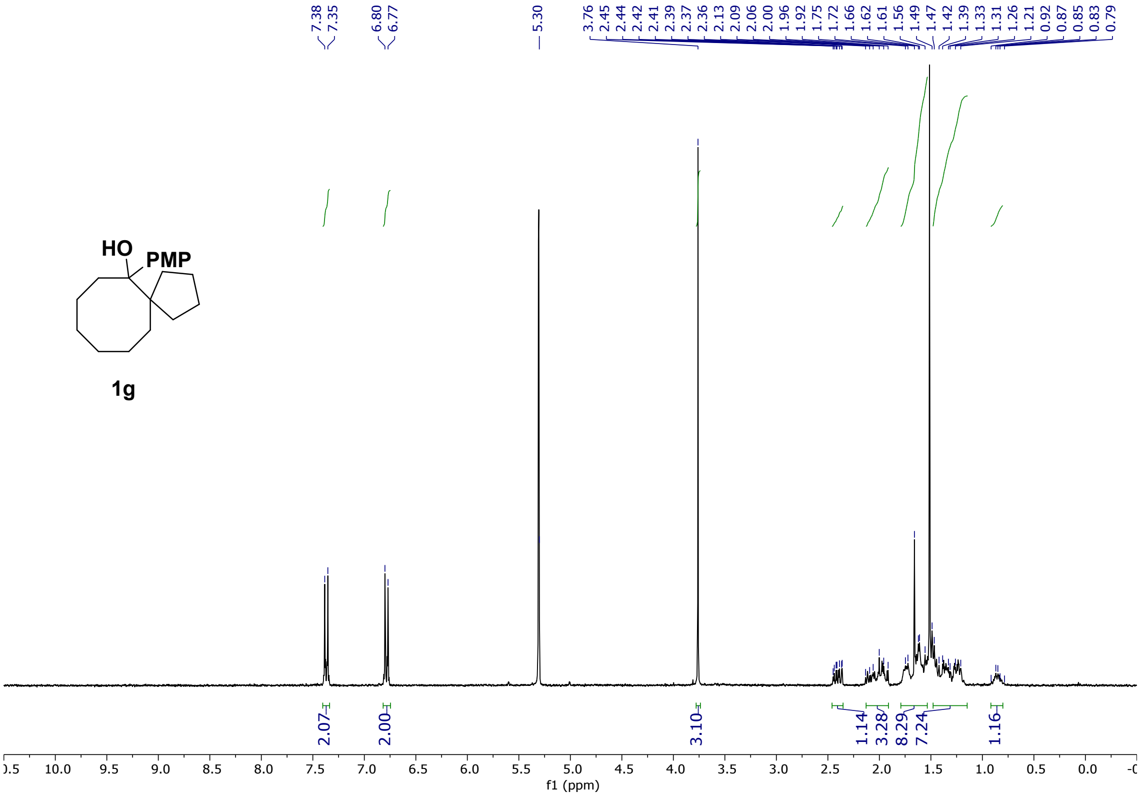

^{13}C NMR of **1g** (75 MHz, CD_2Cl_2)

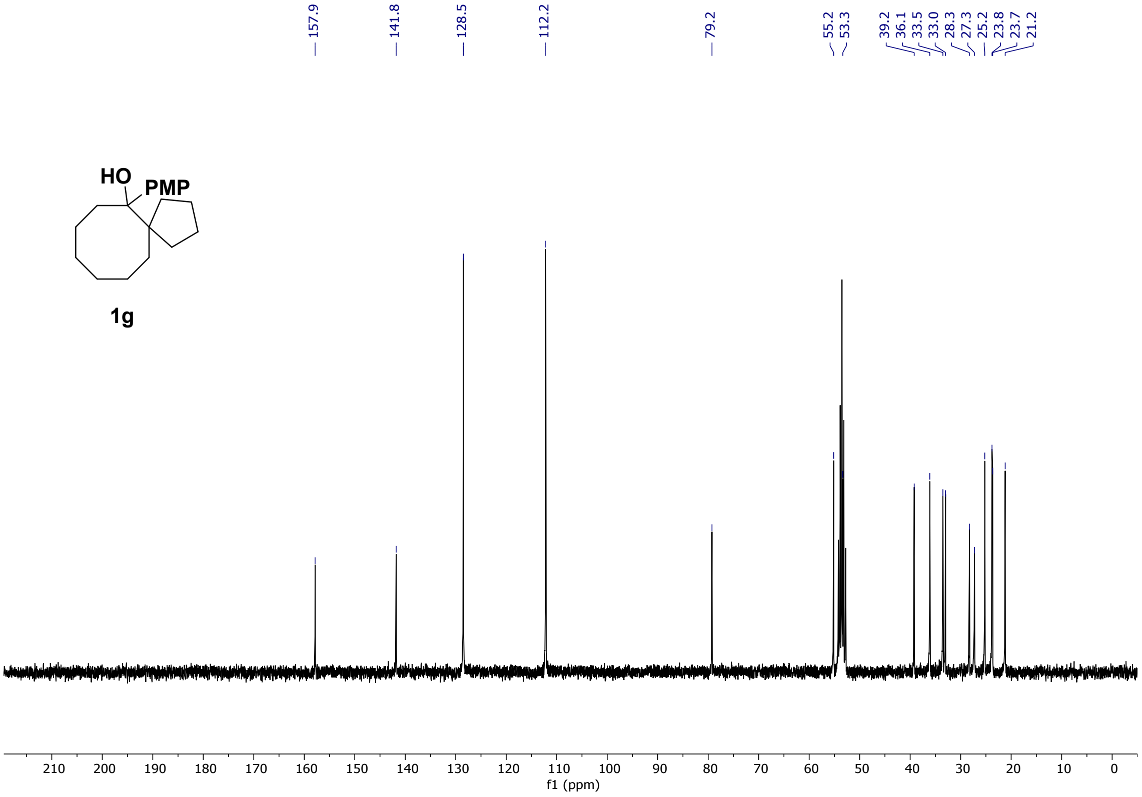

1g

^{1}H NMR of **1h** (300 MHz, CD_2Cl_2)

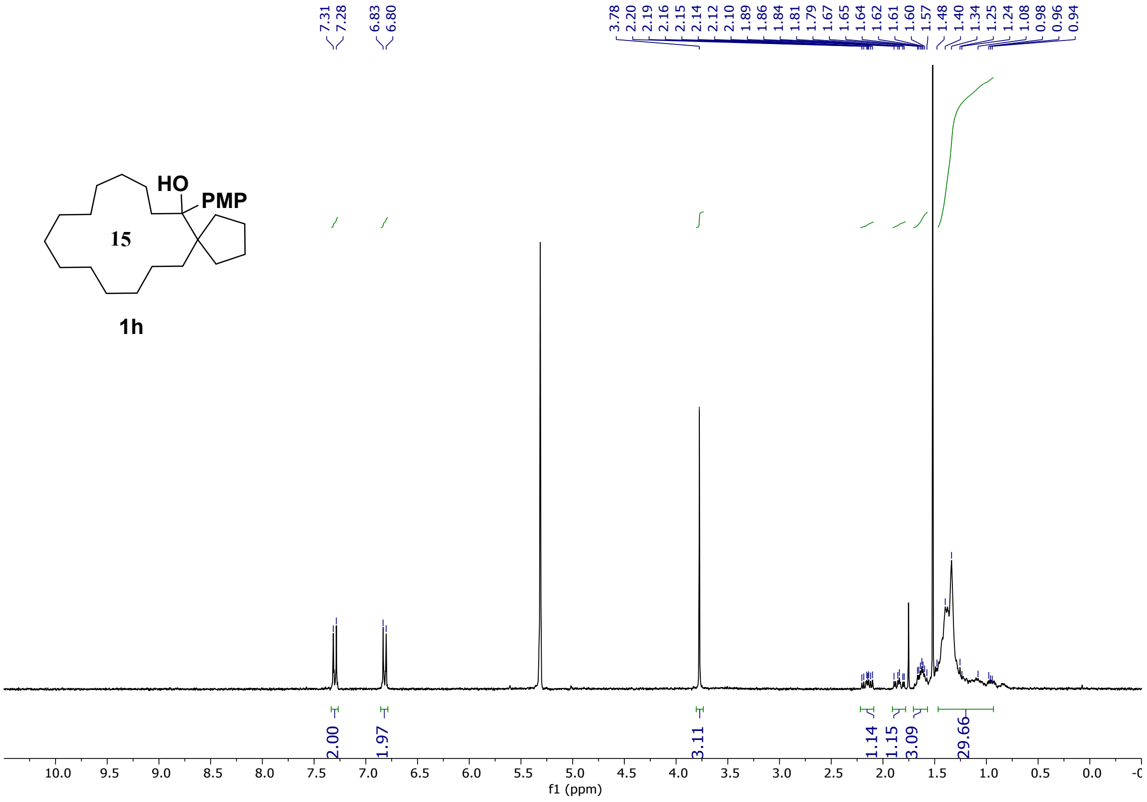

1h

^{13}C NMR of **1h** (75 MHz, CD_2Cl_2)

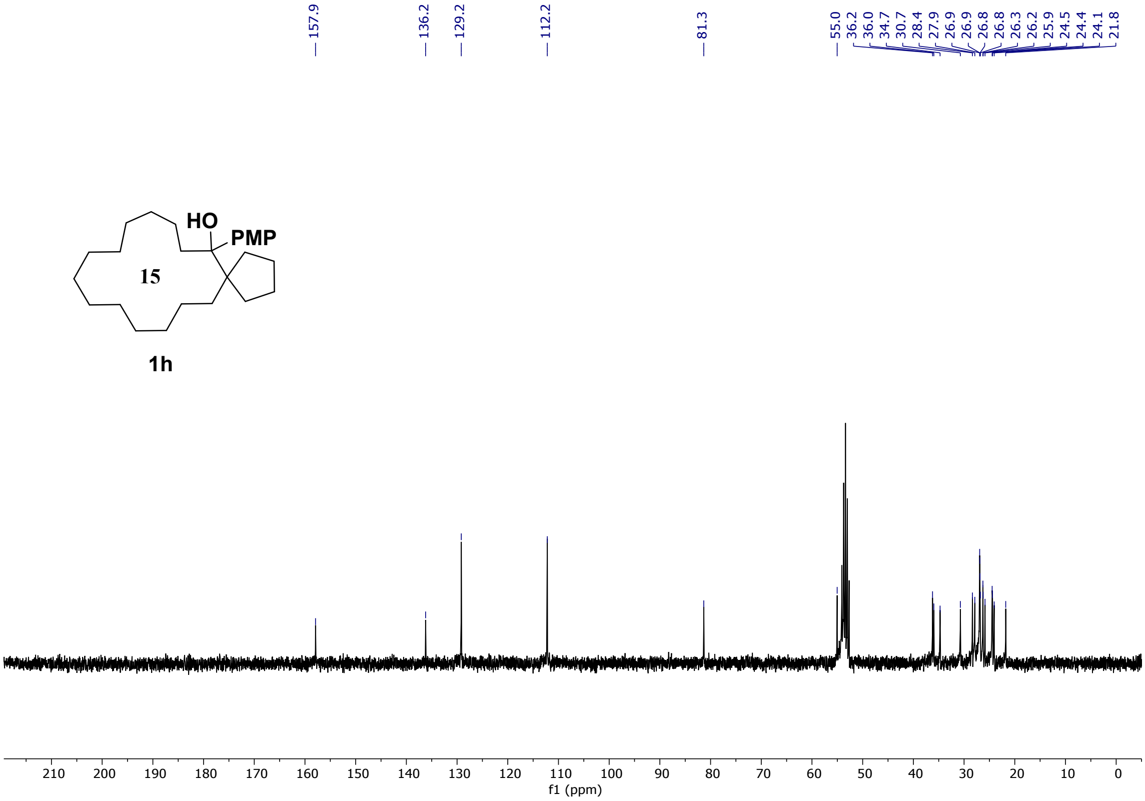

^{1}H NMR of **1i** (300 MHz, $CDCl_3$)

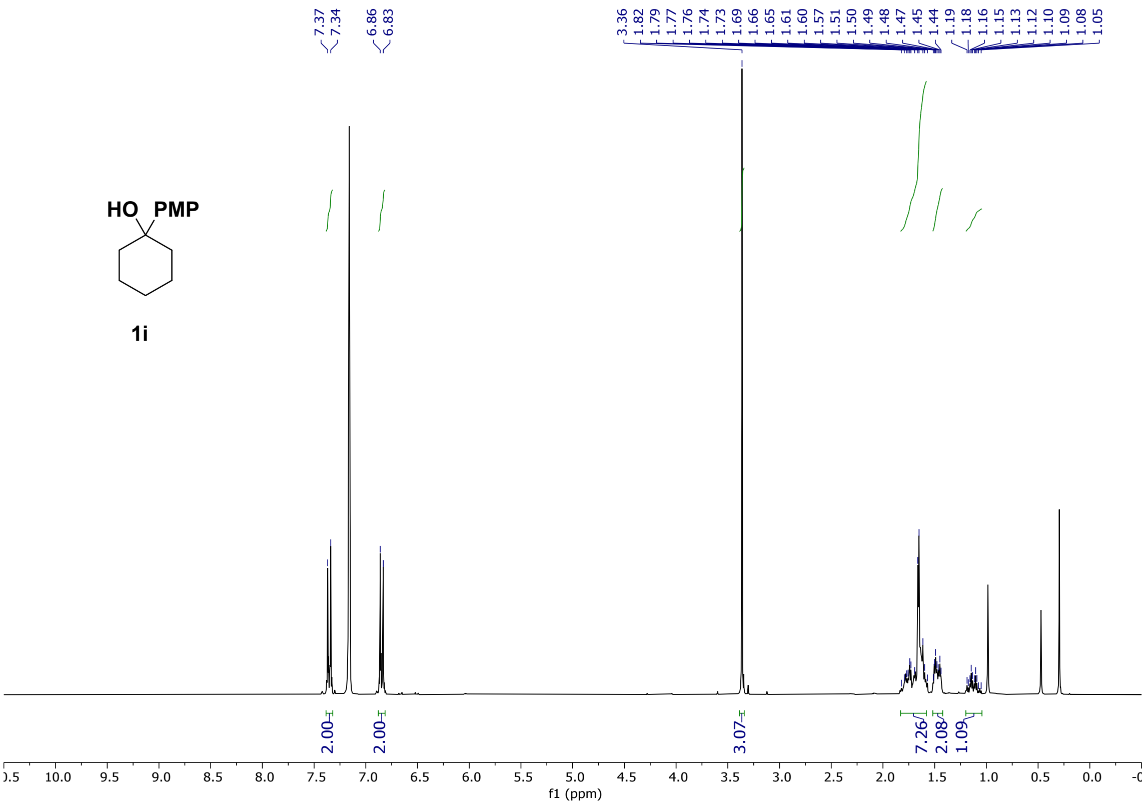

^{1}H NMR of **1j** (300 MHz, C_6D_6)

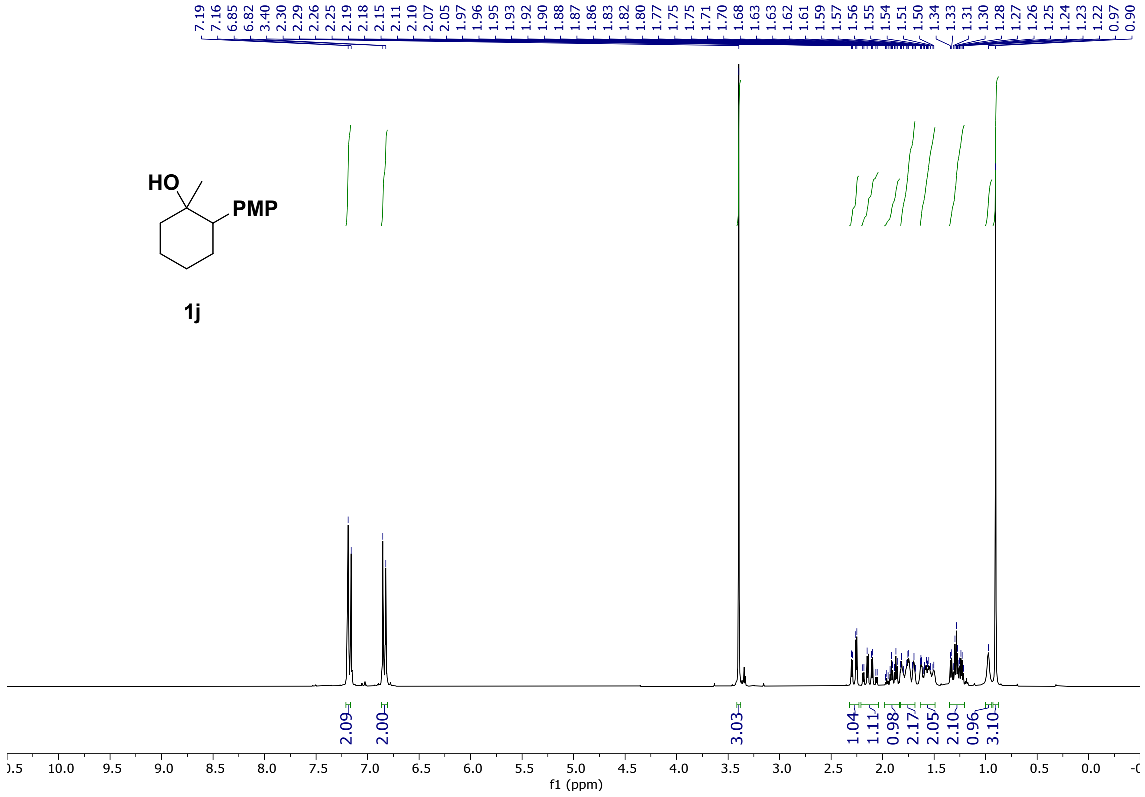

^{1}H NMR of **1k** (300 MHz, $CDCl_3$)

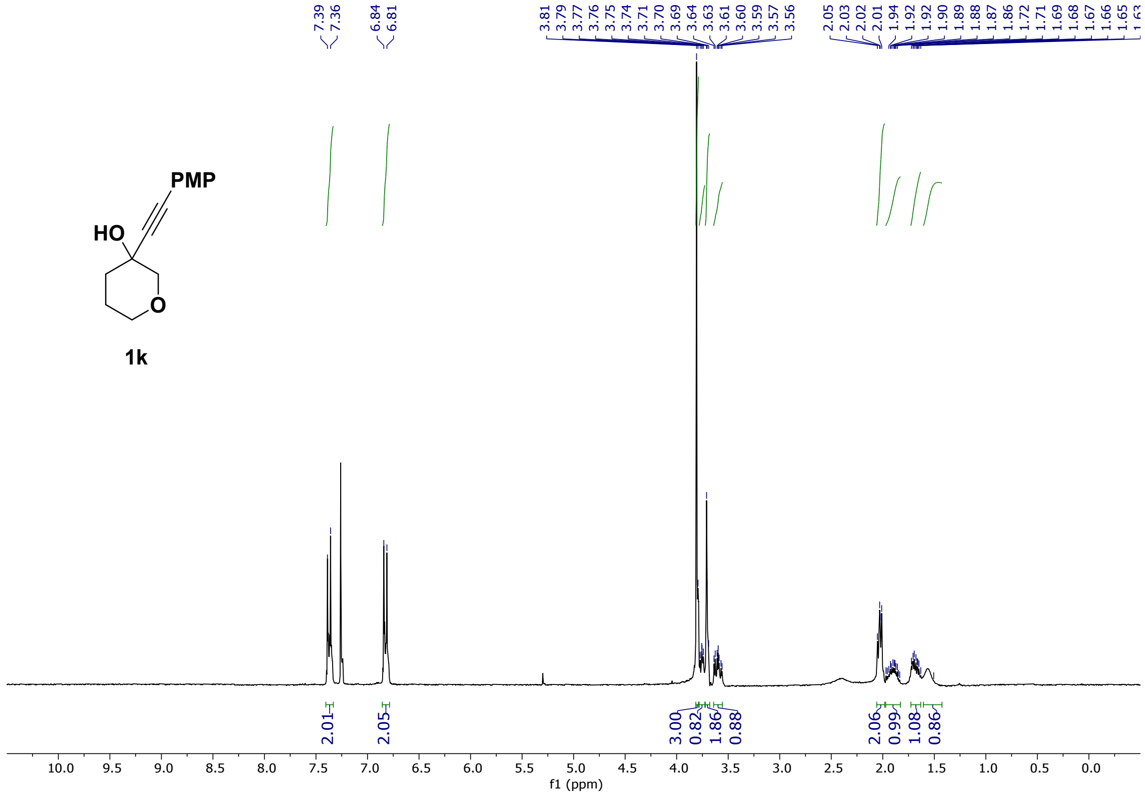

^{13}C NMR of **1k** (75 MHz, $CDCl_3$)

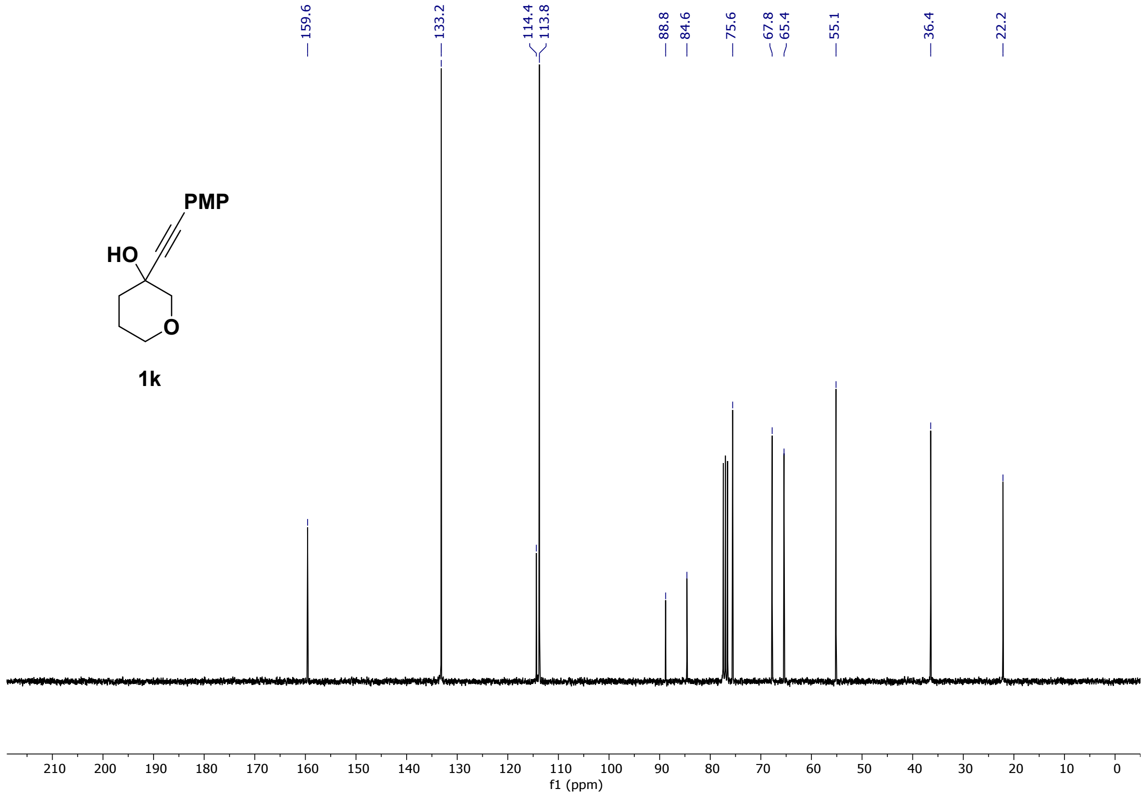

^{1}H NMR of **1l** (300 MHz, CD_2Cl_2)

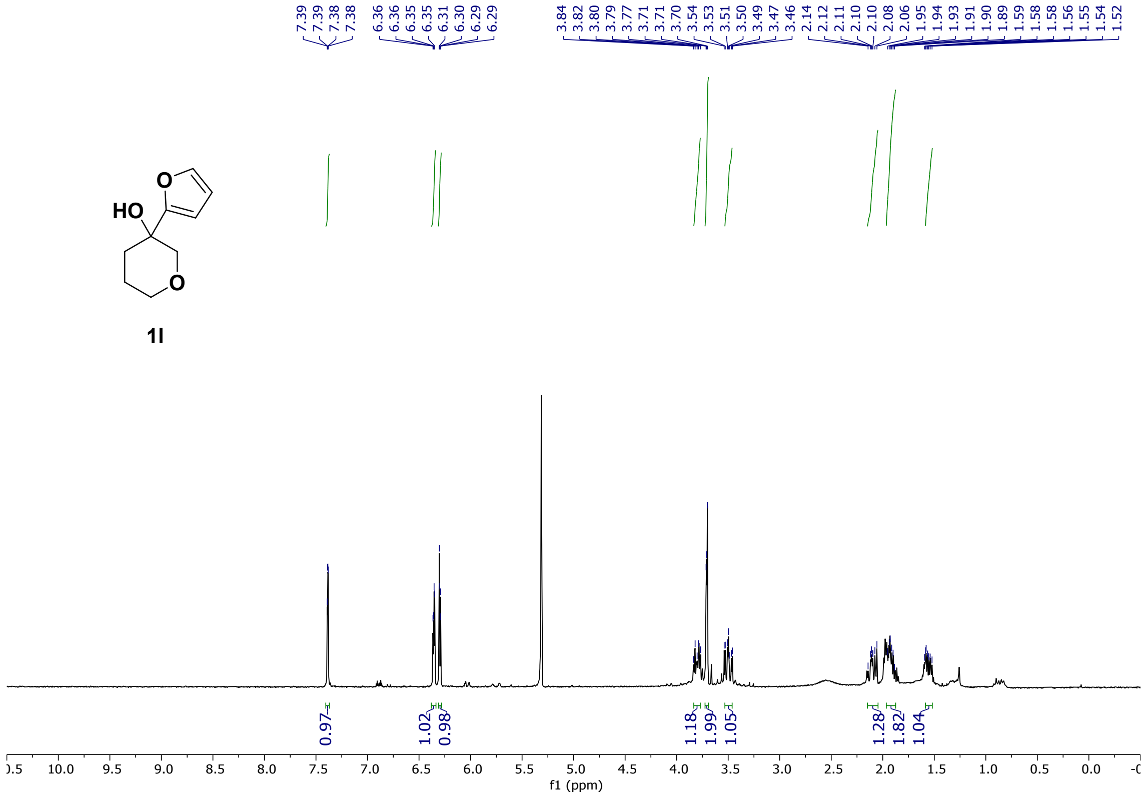

^{13}C NMR of **1l** (75 MHz, CD_2Cl_2)

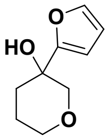

1l

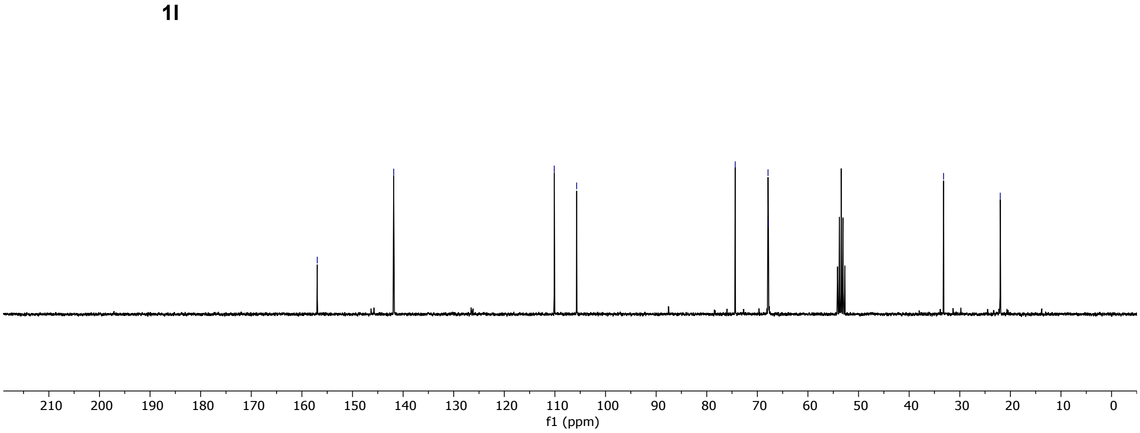

^{1}H NMR of **1m** (300 MHz, $CDCl_3$)

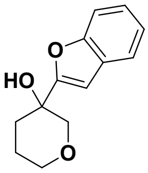

1m

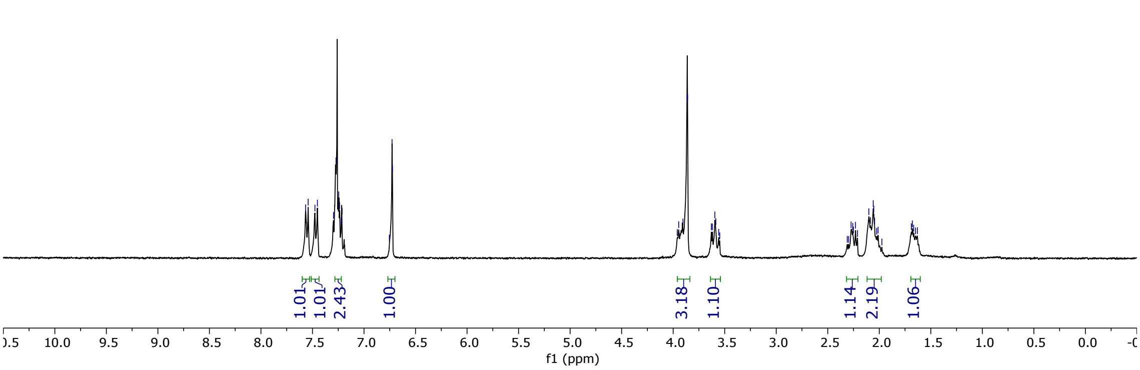

^{13}C NMR of **1m** (75 MHz, CDCl$_3$)

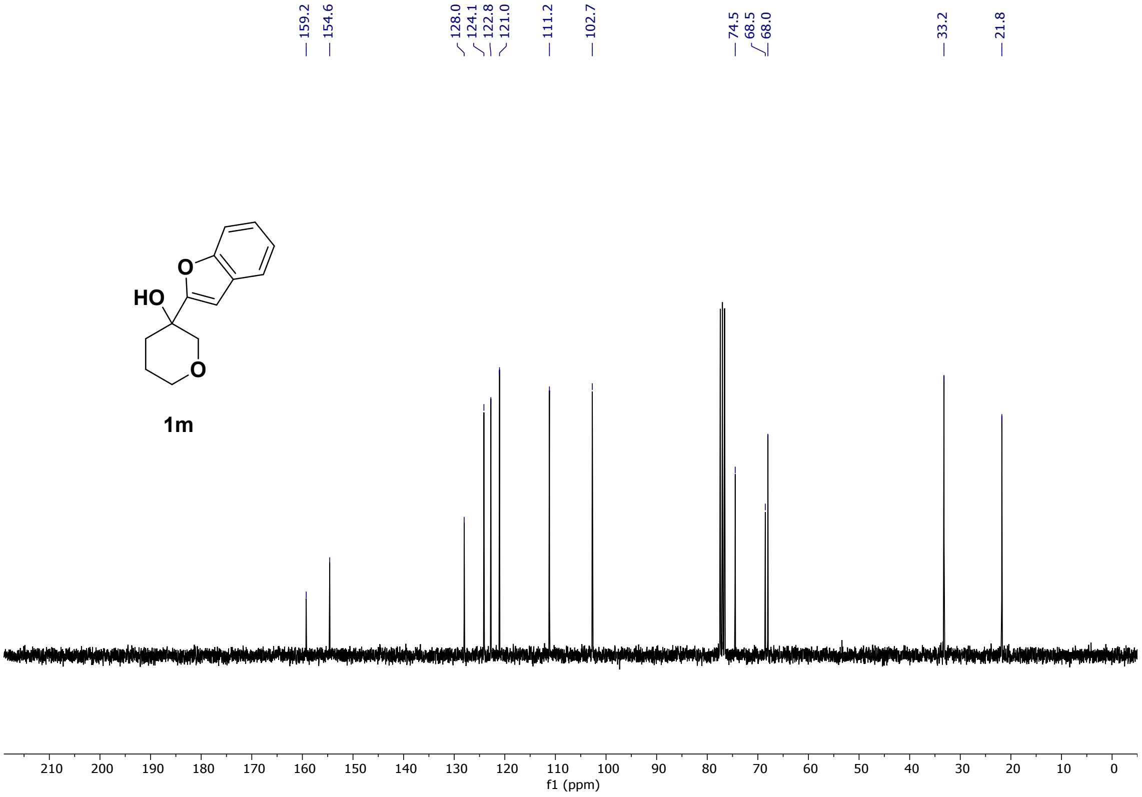

^{1}H NMR of **1n** (300 MHz, CDCl$_3$)

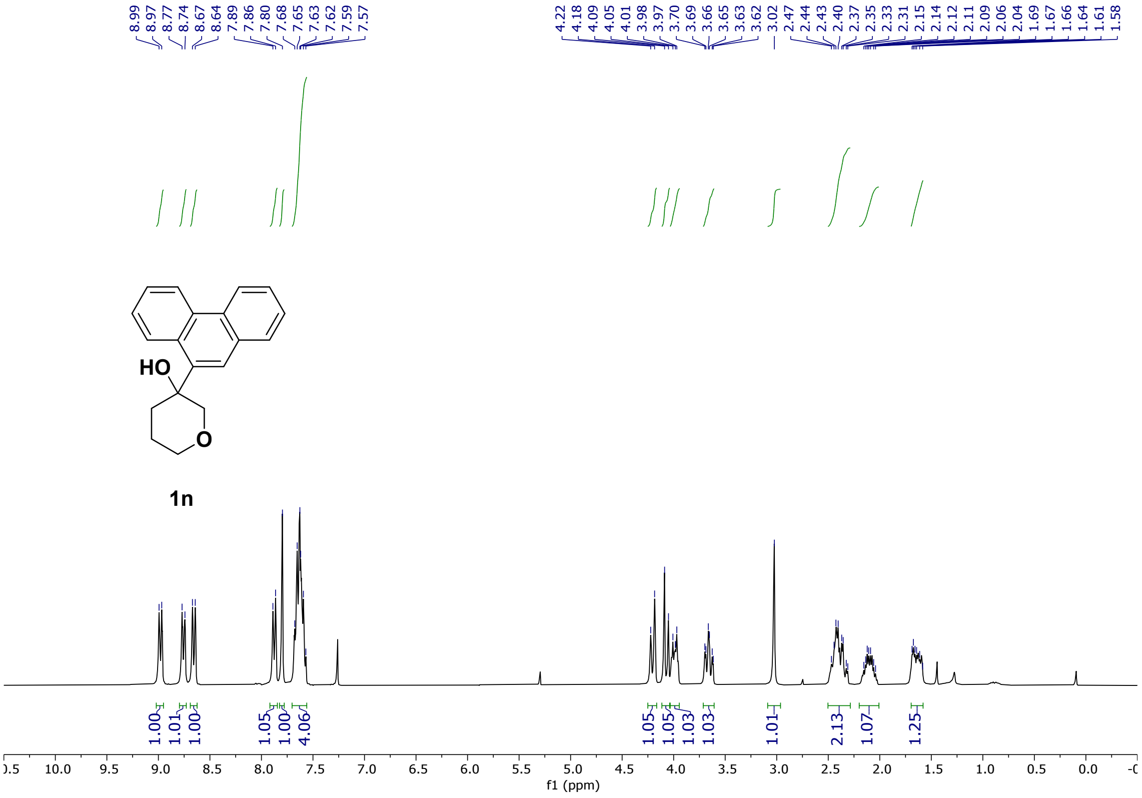

^{13}C NMR of **1n** (75 MHz, $CDCl_3$)

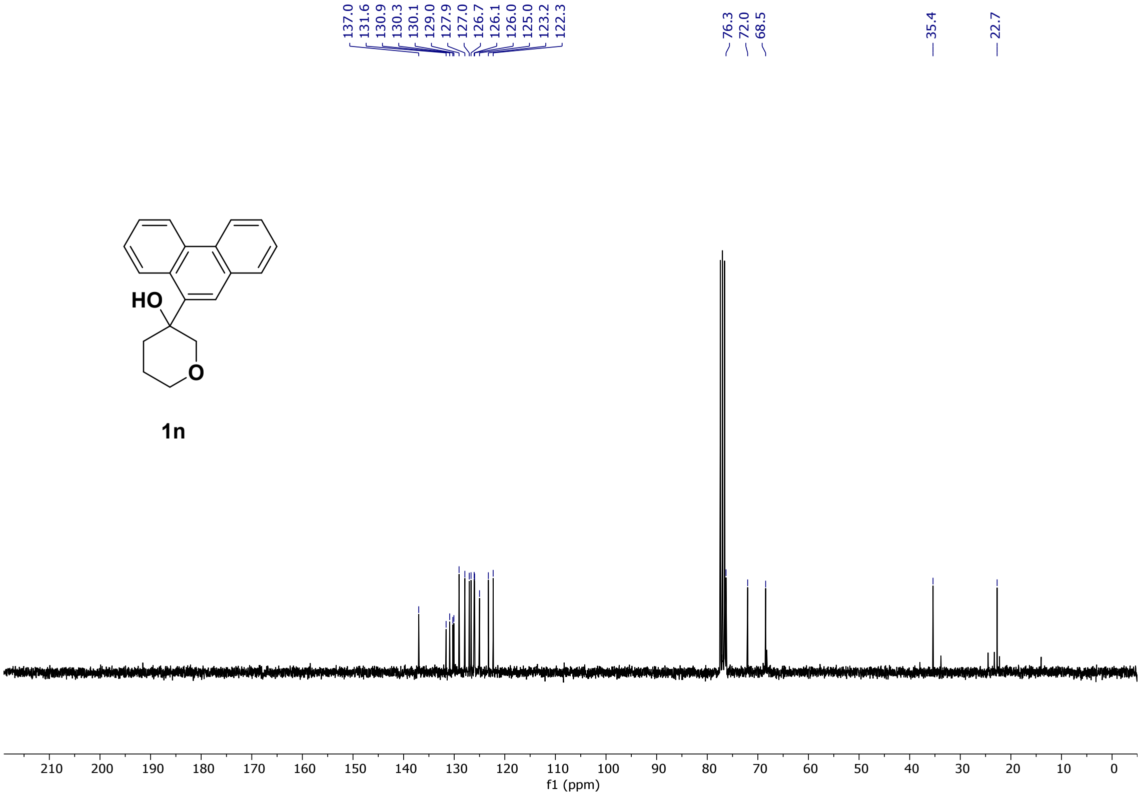

^{1}H NMR of **SI6** (300 MHz, $CDCl_3$)

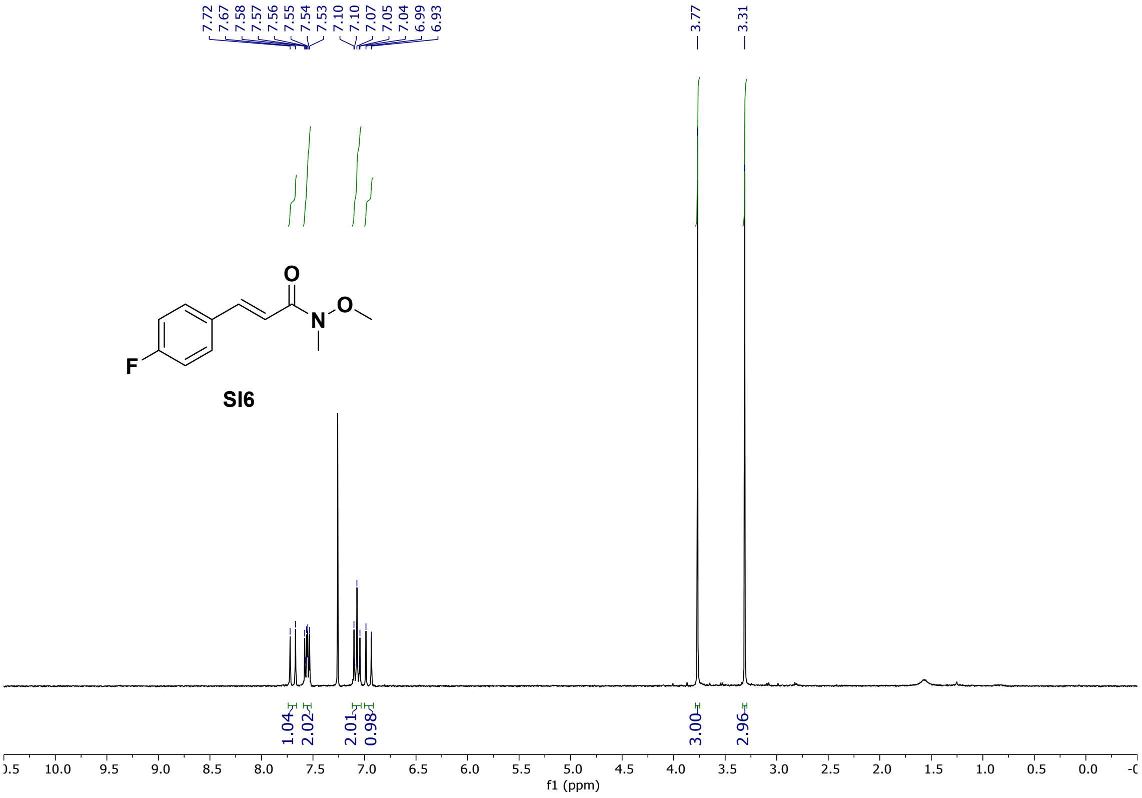

^{13}C NMR of **SI6** (75 MHz, $CDCl_3$)

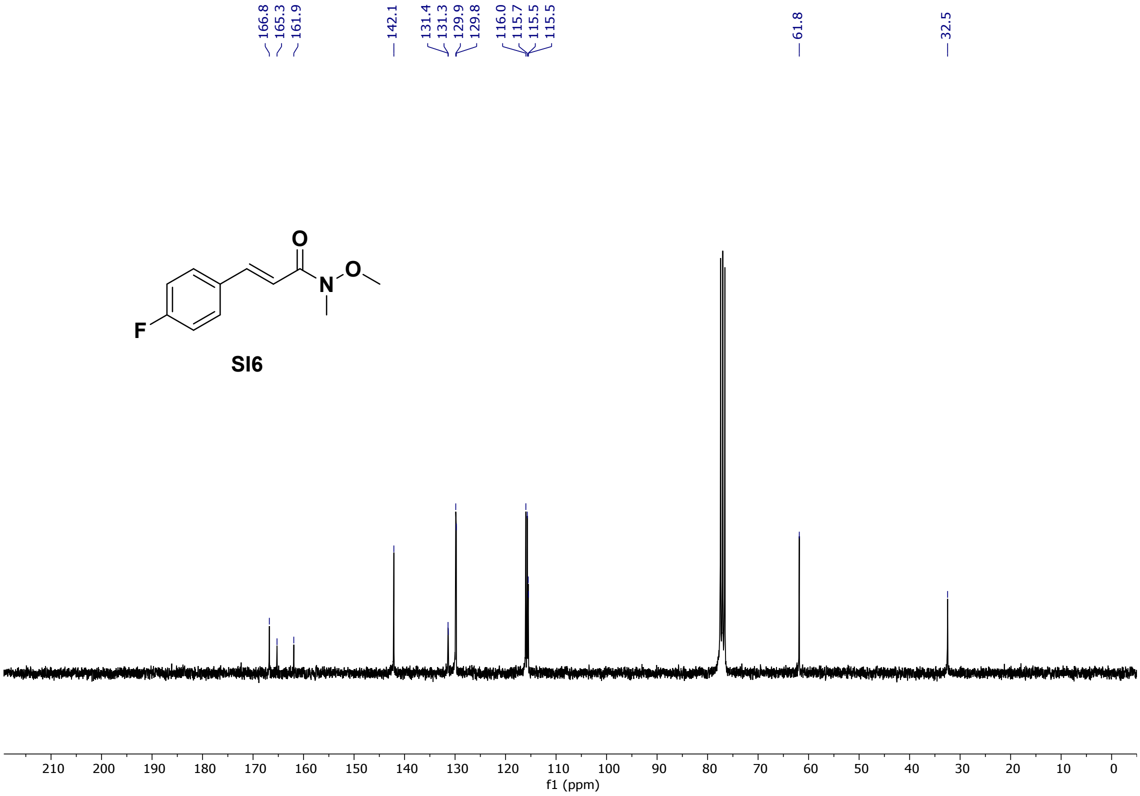

^{19}F NMR of **SI6** (471 MHz, $CDCl_3$)

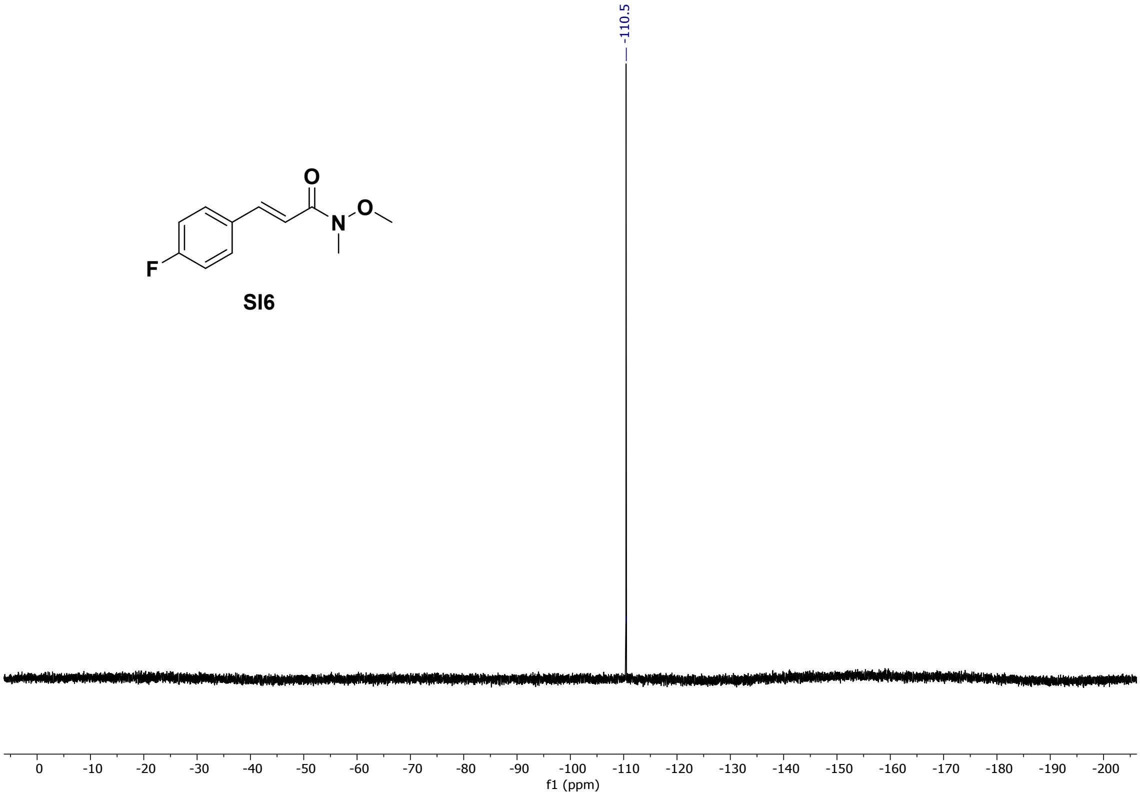

^{1}H NMR of **SI7** (300 MHz, $CDCl_3$)

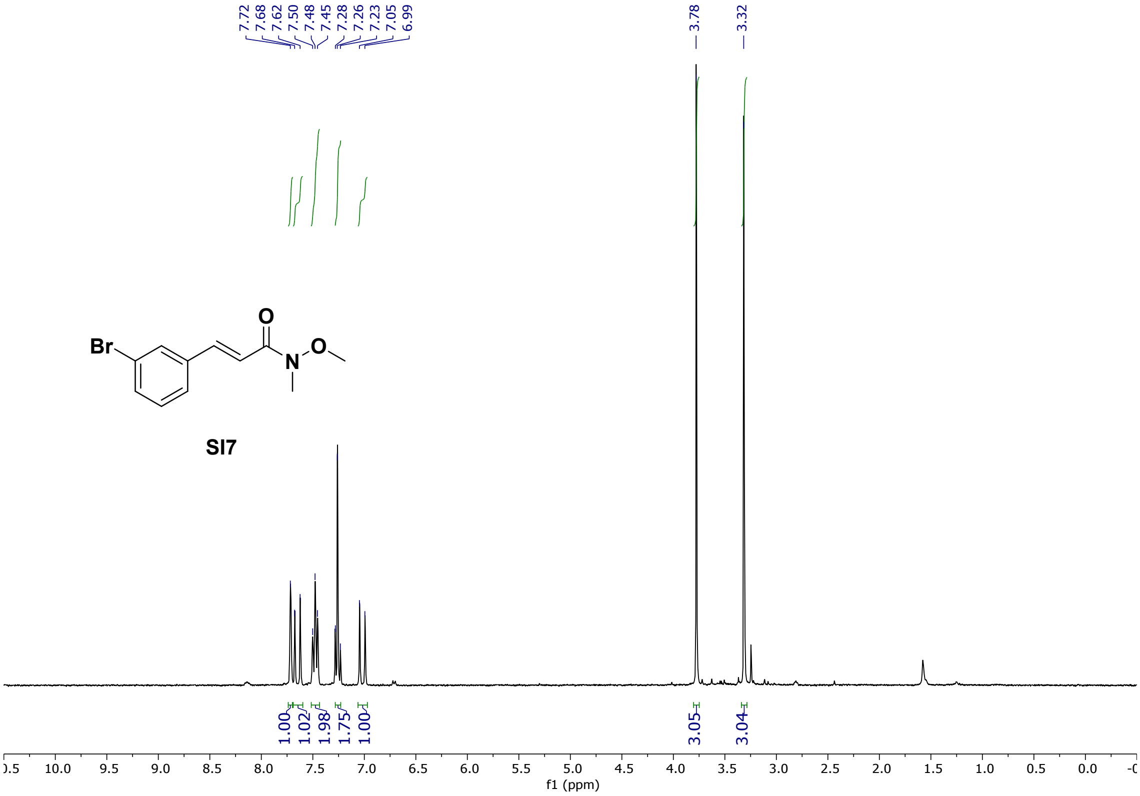

^{13}C NMR of **SI7** (75 MHz, $CDCl_3$)

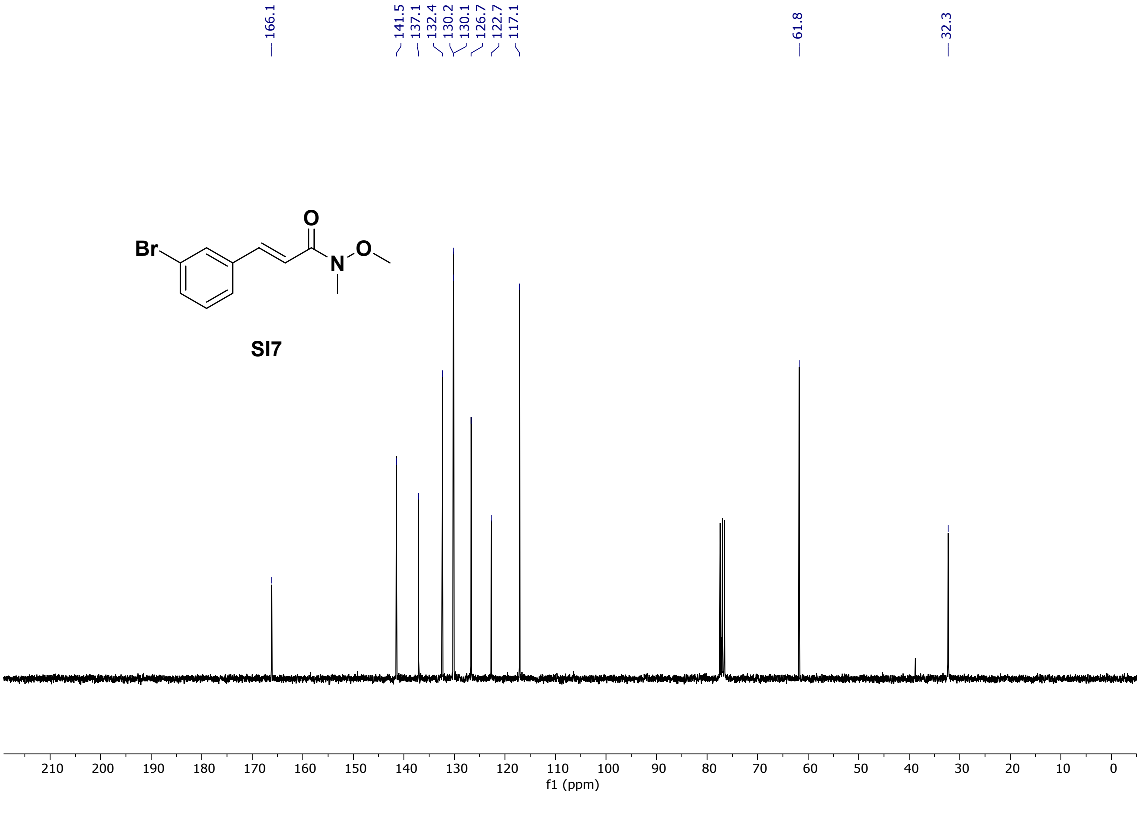

^{1}H NMR of **SI8** (300 MHz, $CDCl_3$)

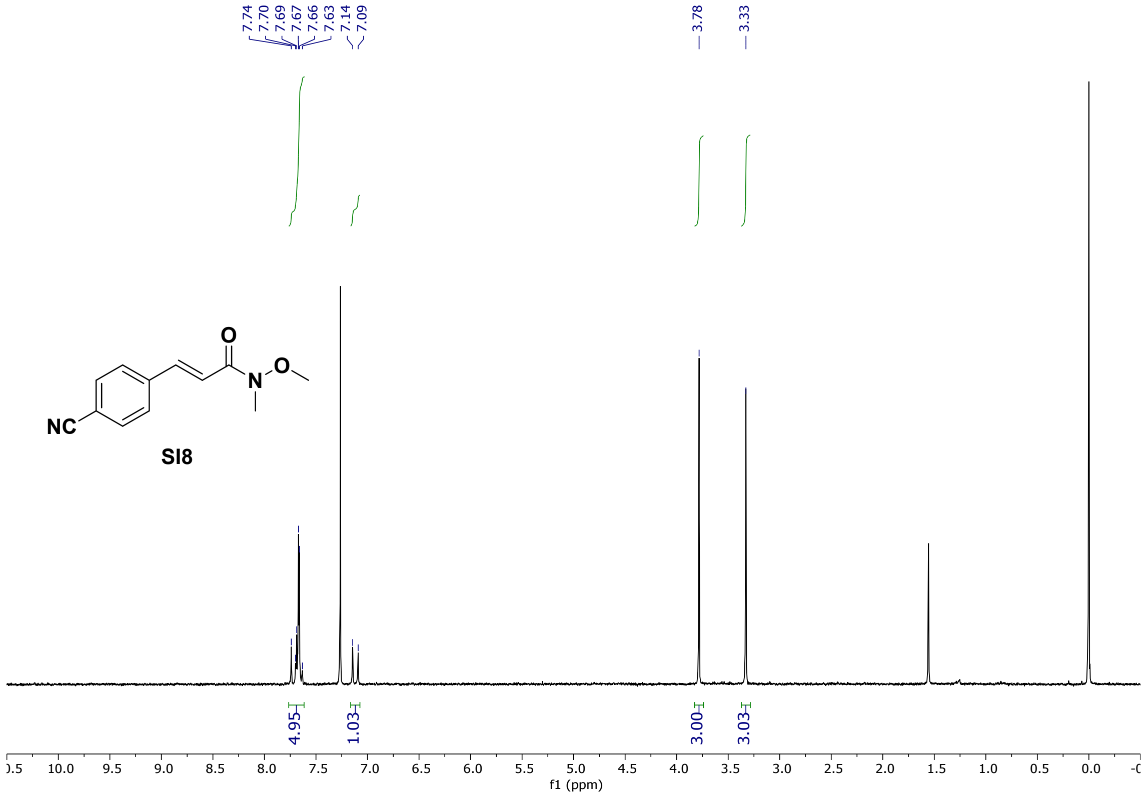

^{13}C NMR of **SI8** (75 MHz, $CDCl_3$)

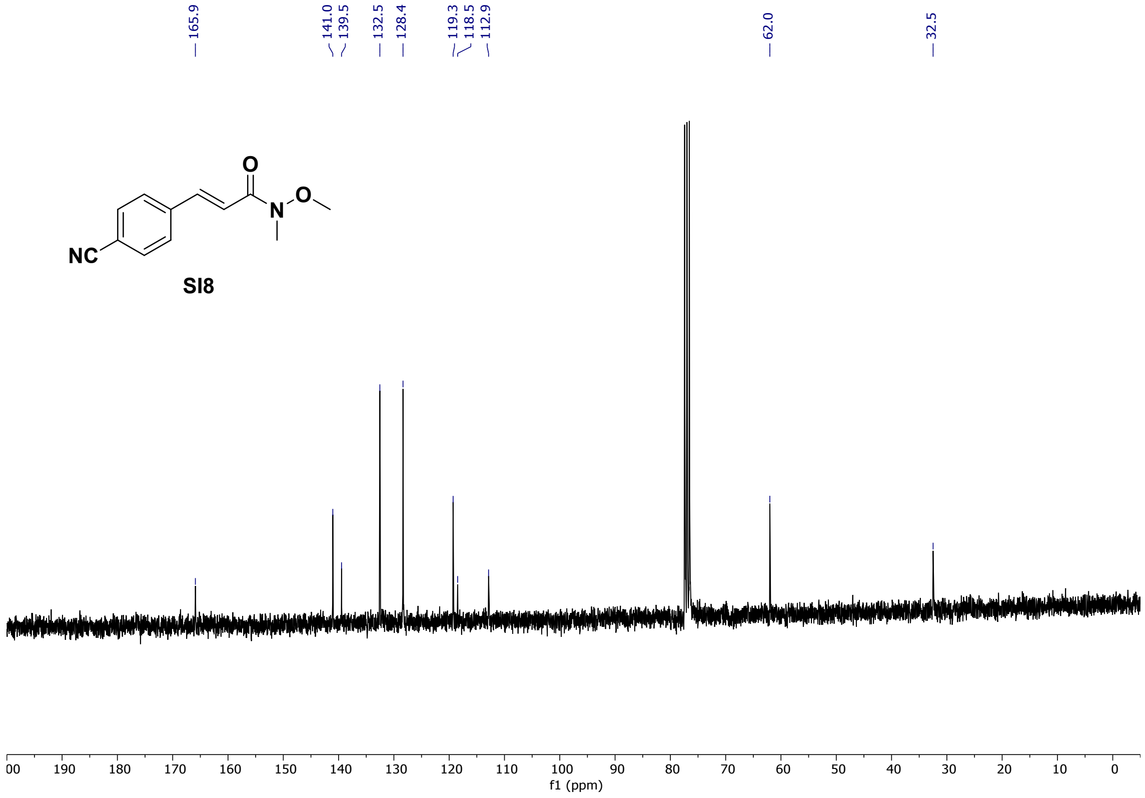

^{1}H NMR of **SI9** (300 MHz, $CDCl_3$)

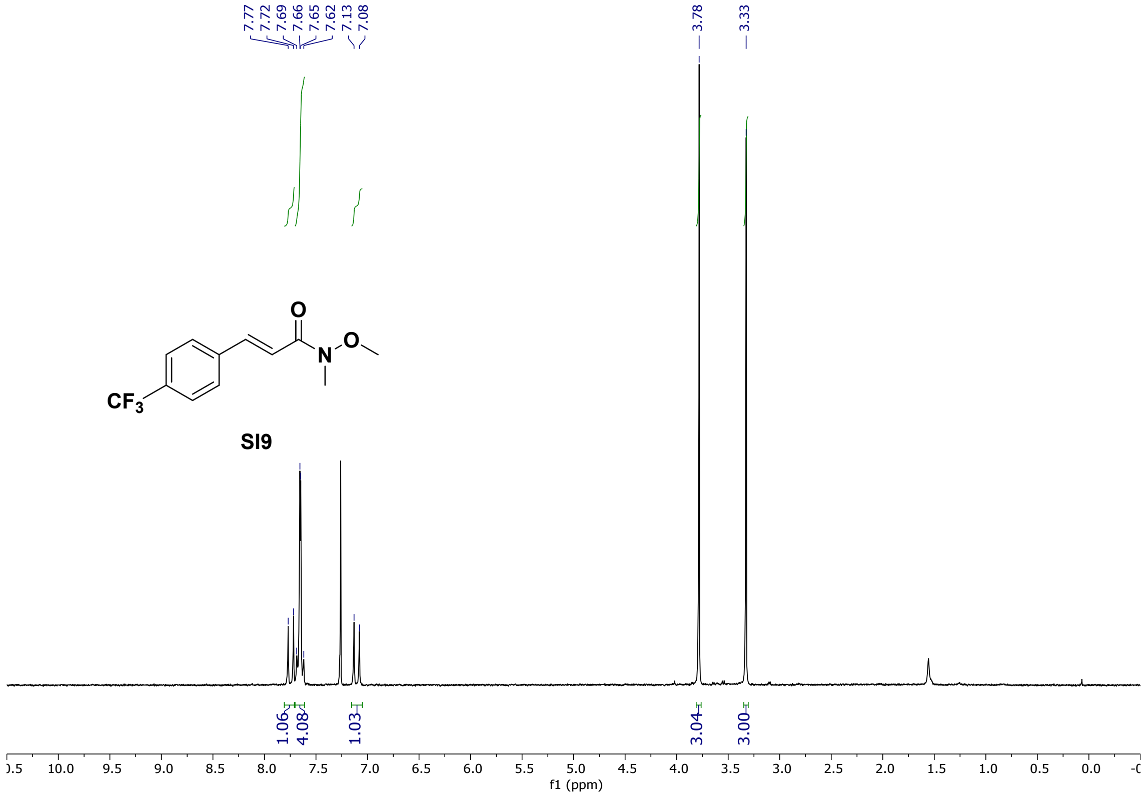

^{13}C NMR of **SI9** (75 MHz, $CDCl_3$)

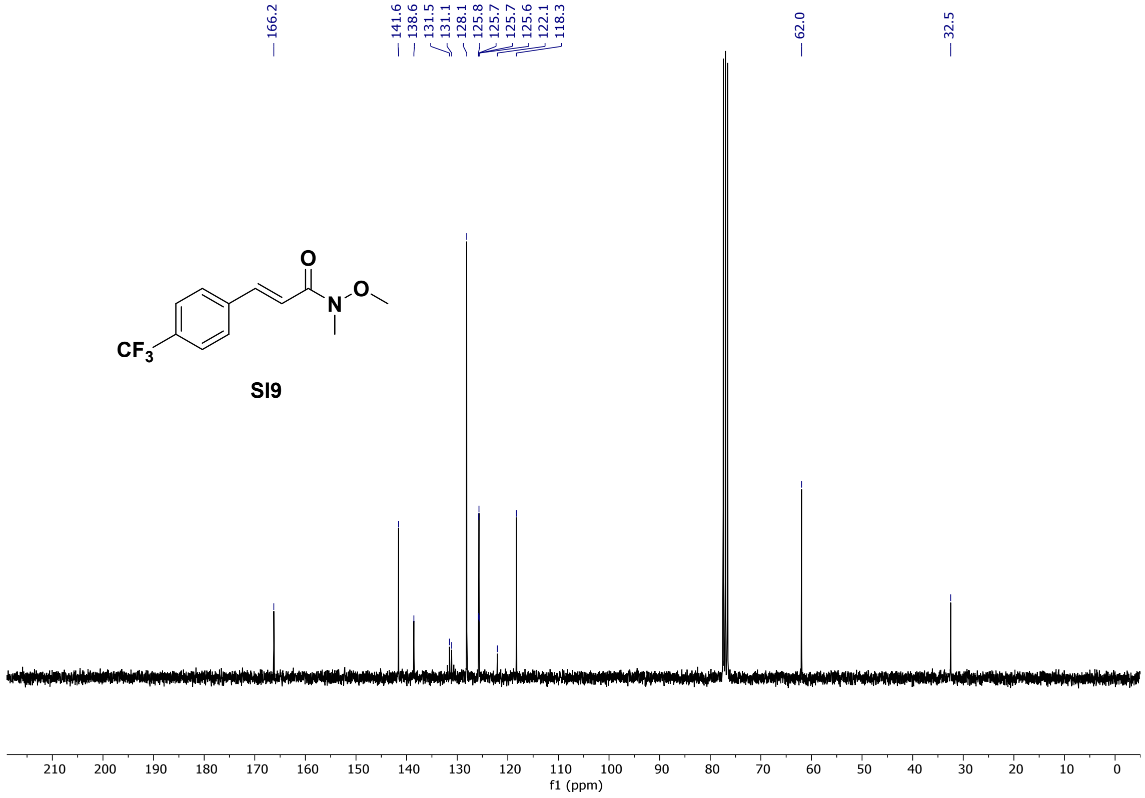

^{19}F NMR of **SI9** (471 MHz, $CDCl_3$)

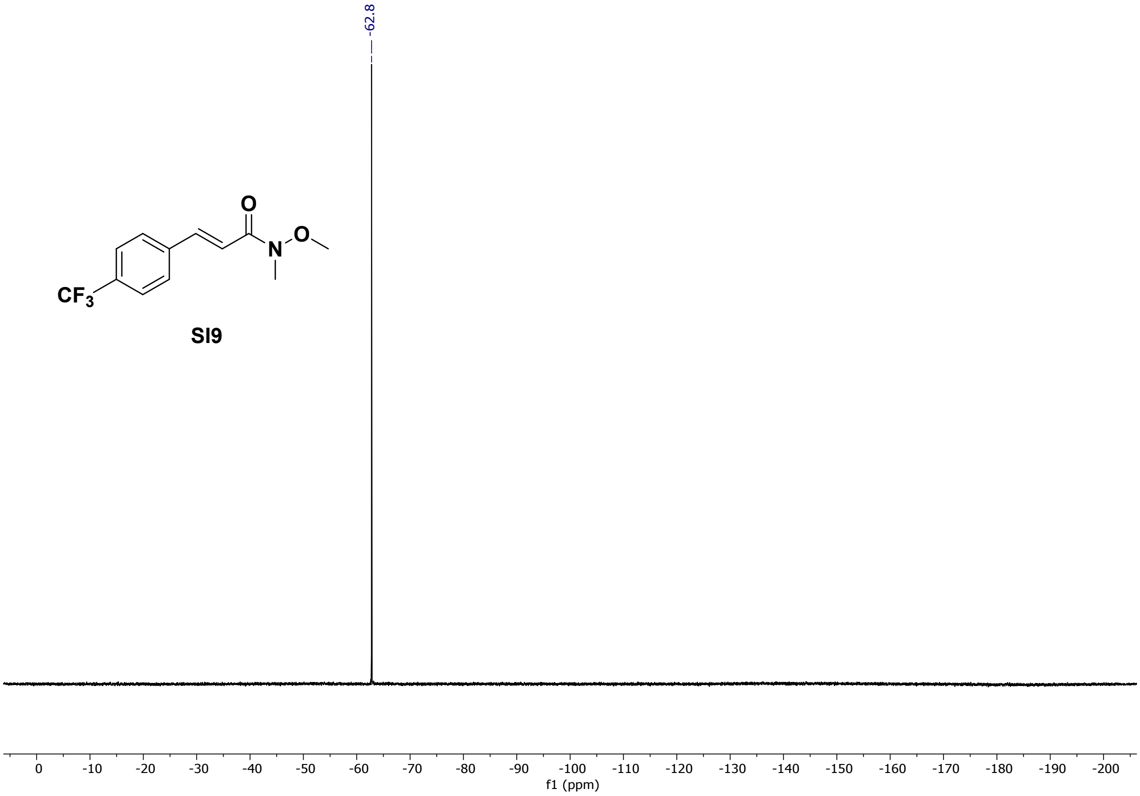

^{1}H NMR of **2a** (300 MHz, $CDCl_3$)

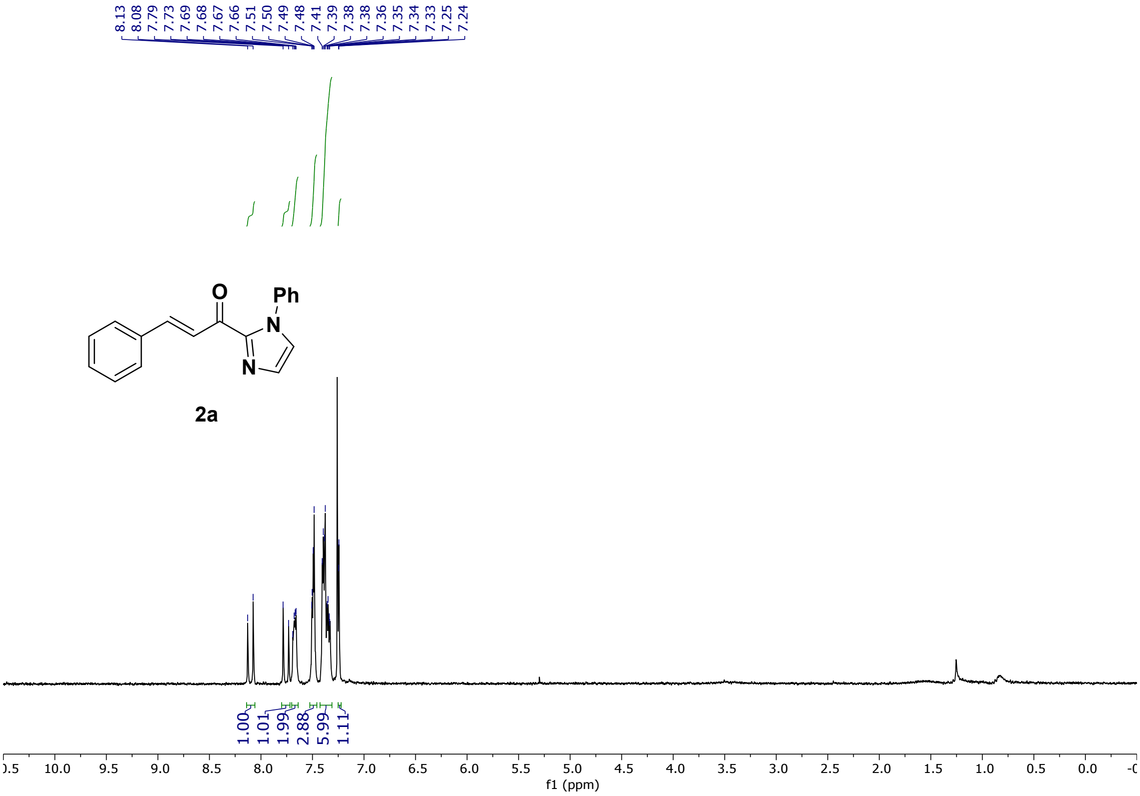

^{1}H NMR of **2o** (300 MHz, $CDCl_3$)

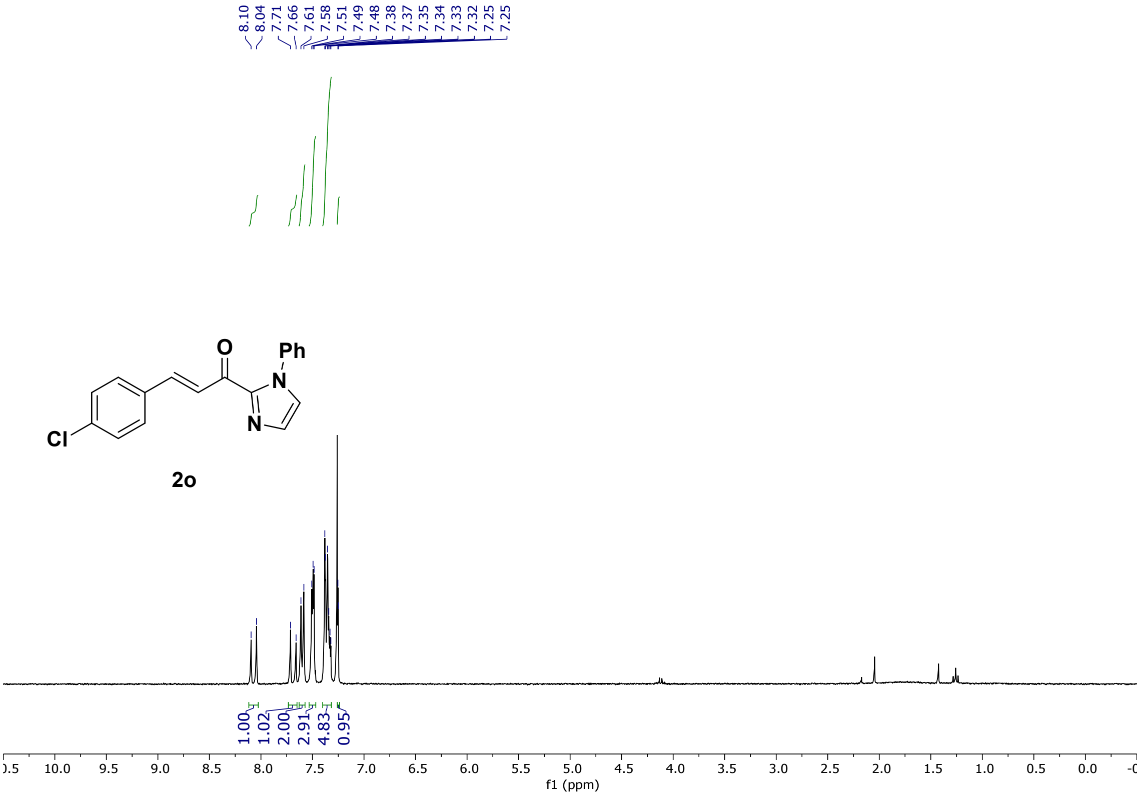

^{1}H NMR of **2p** (300 MHz, $CDCl_3$)

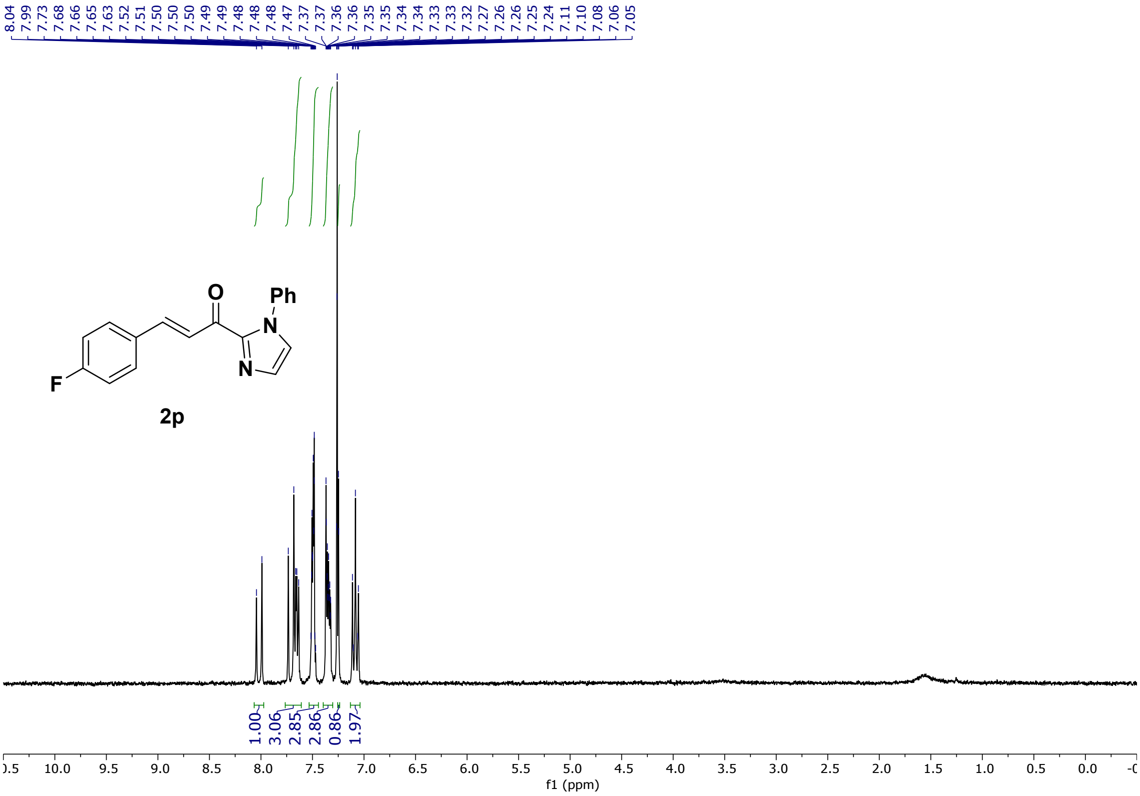

^{1}H NMR of **2q** (300 MHz, $CDCl_3$)

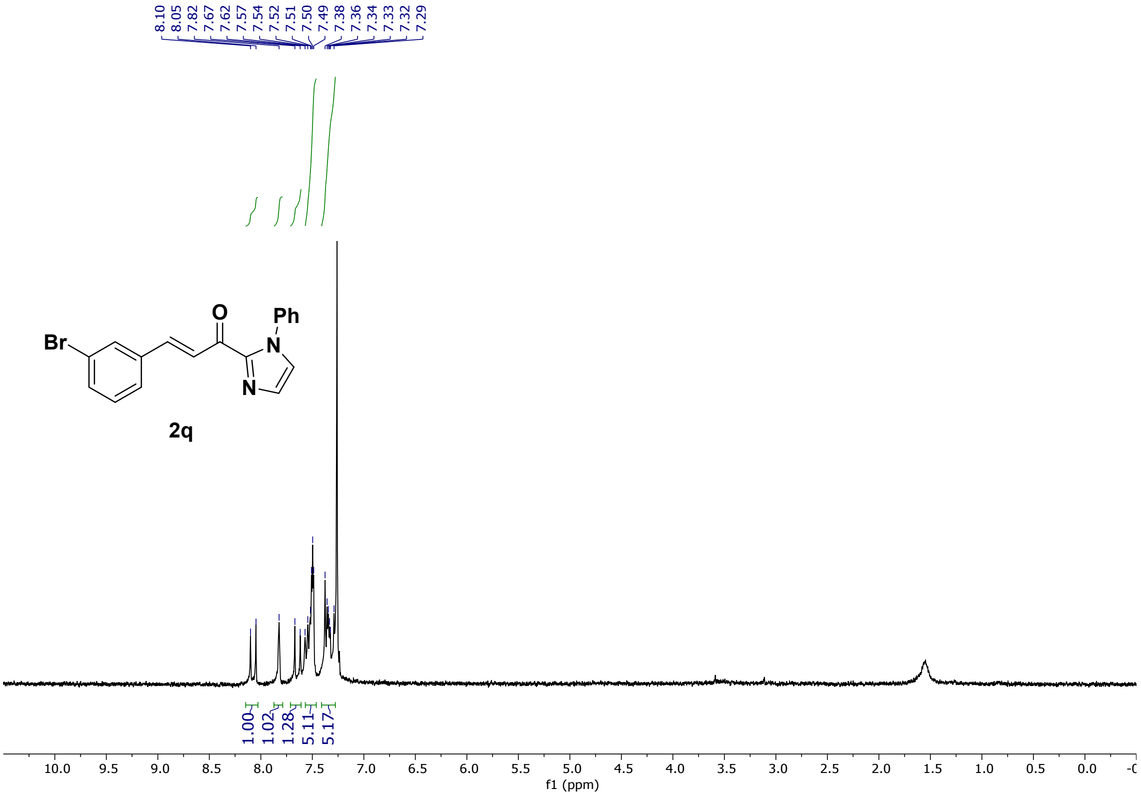

^{1}H NMR of **2r** (300 MHz, $CDCl_3$)

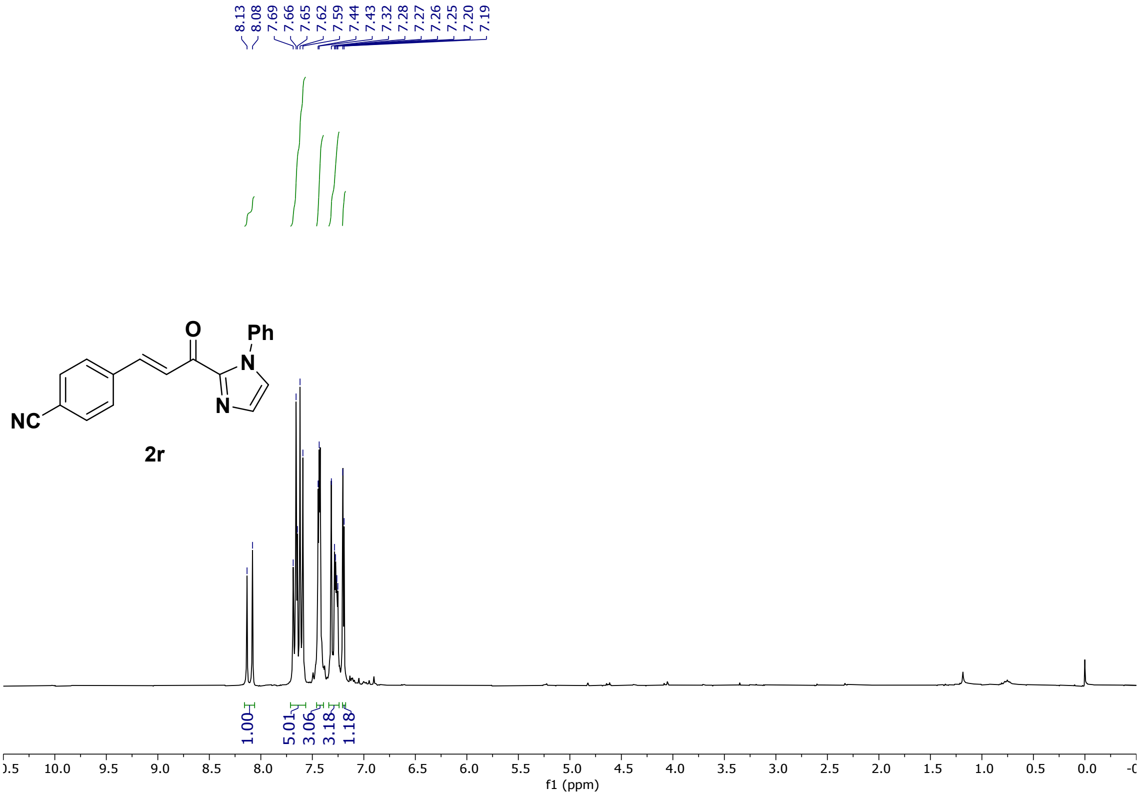

^{13}C NMR of **2r** (75 MHz, $CDCl_3$)

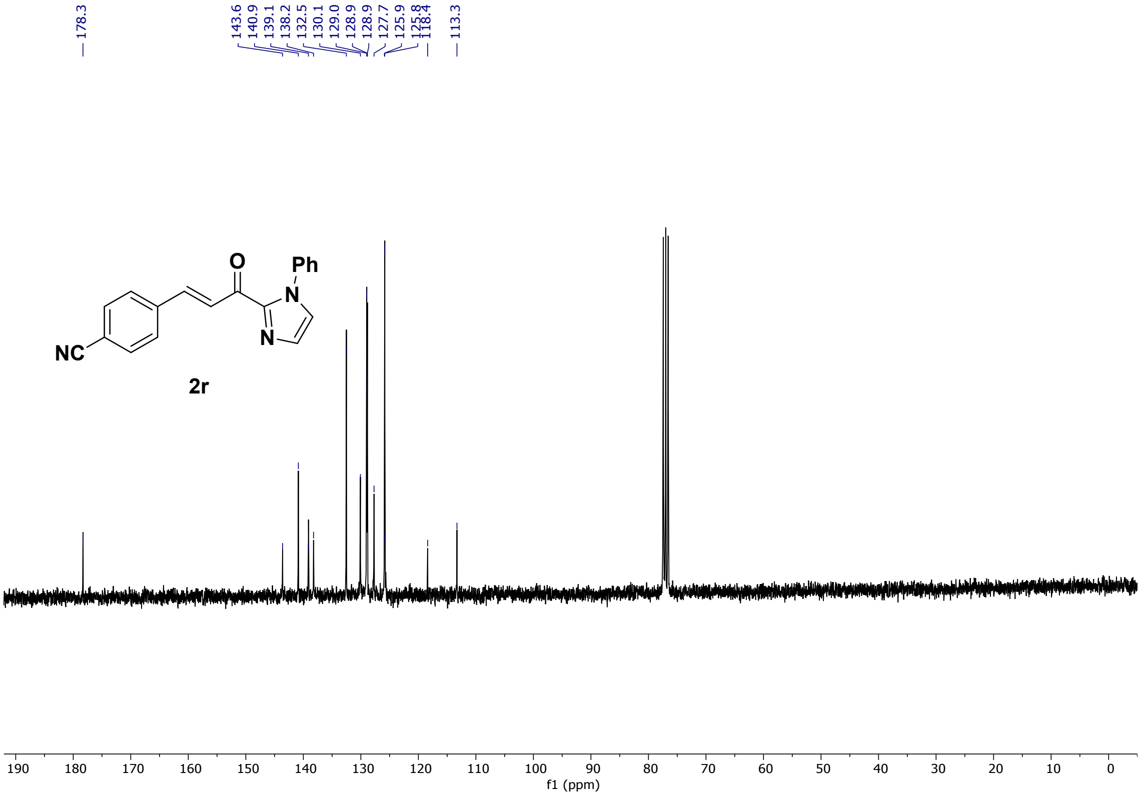

^{1}H NMR of **2s** (300 MHz, $CDCl_3$)

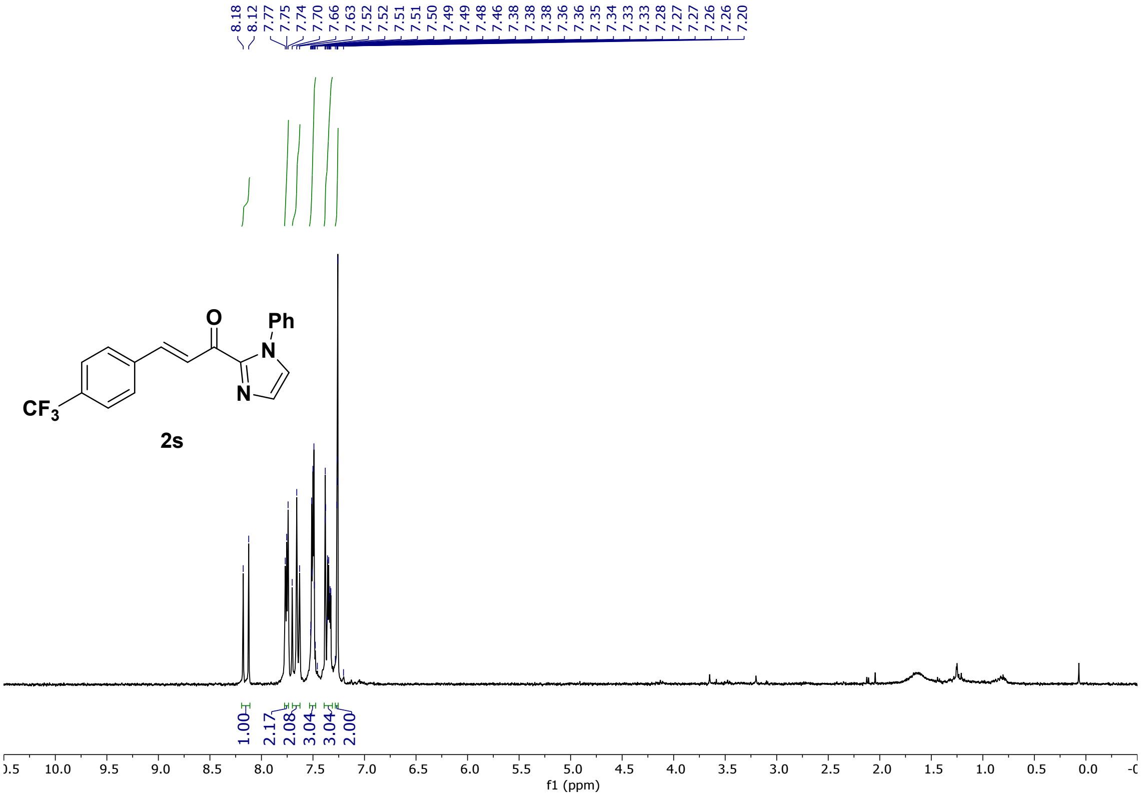

^{1}H NMR of **2u** (300 MHz, $CDCl_3$)

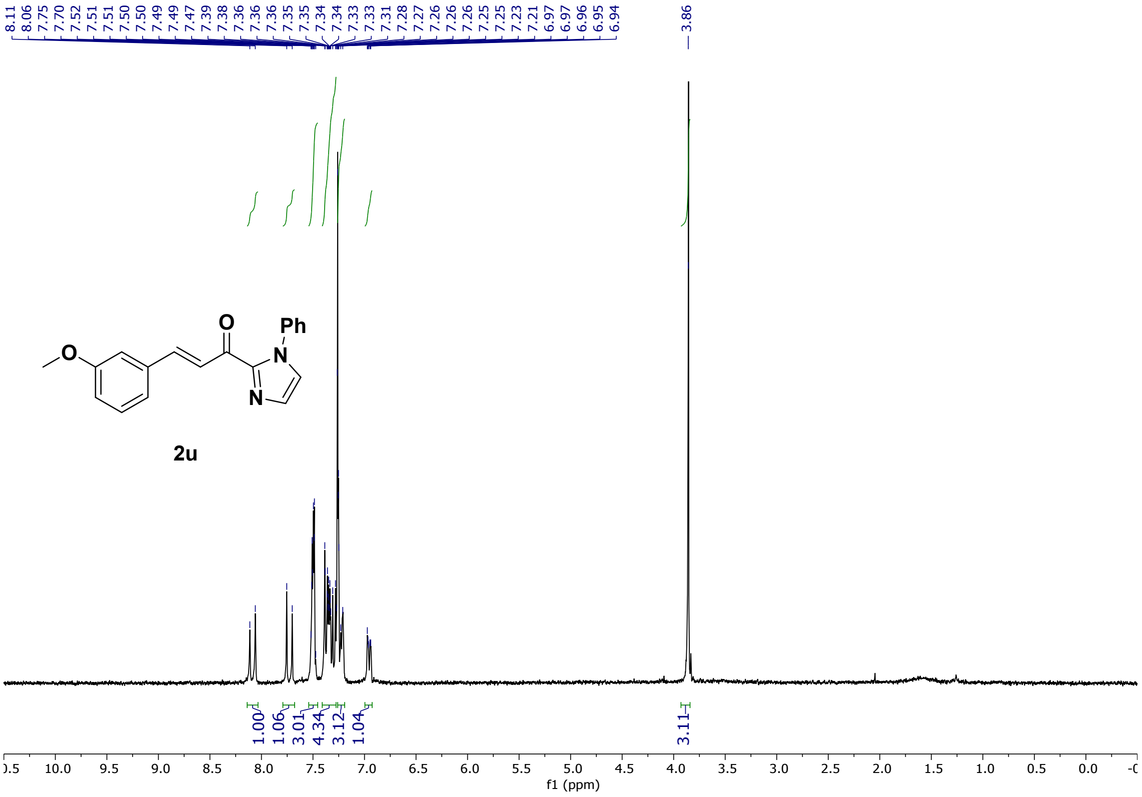

^{1}H NMR of **2v** (300 MHz, $CDCl_3$)

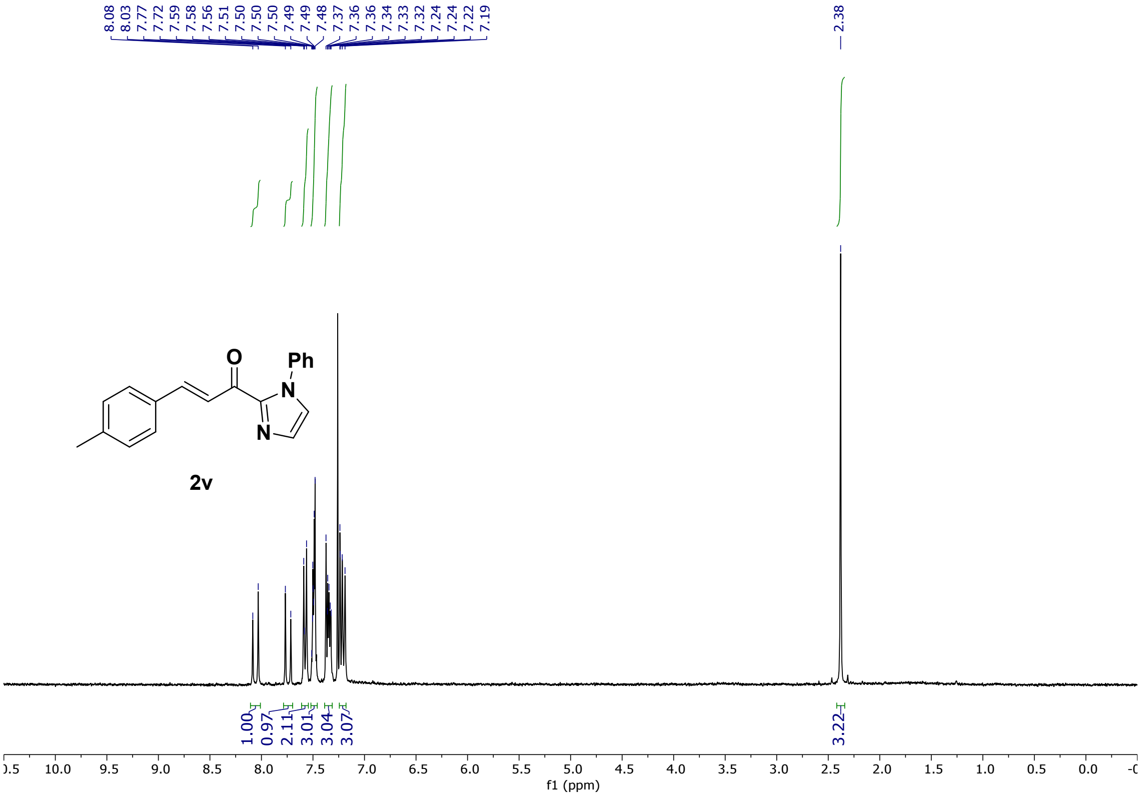

^{13}C NMR of **2v** (75 MHz, $CDCl_3$)

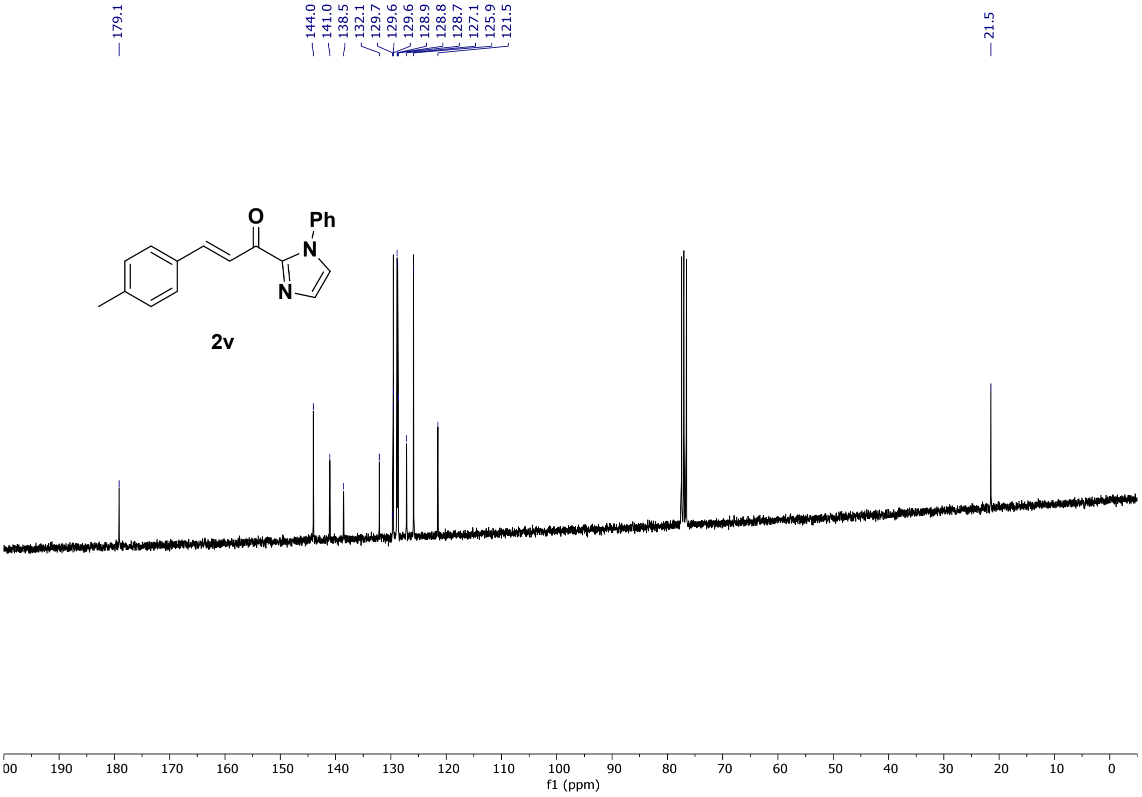

^{1}H NMR of **2w** (300 MHz, $CDCl_3$)

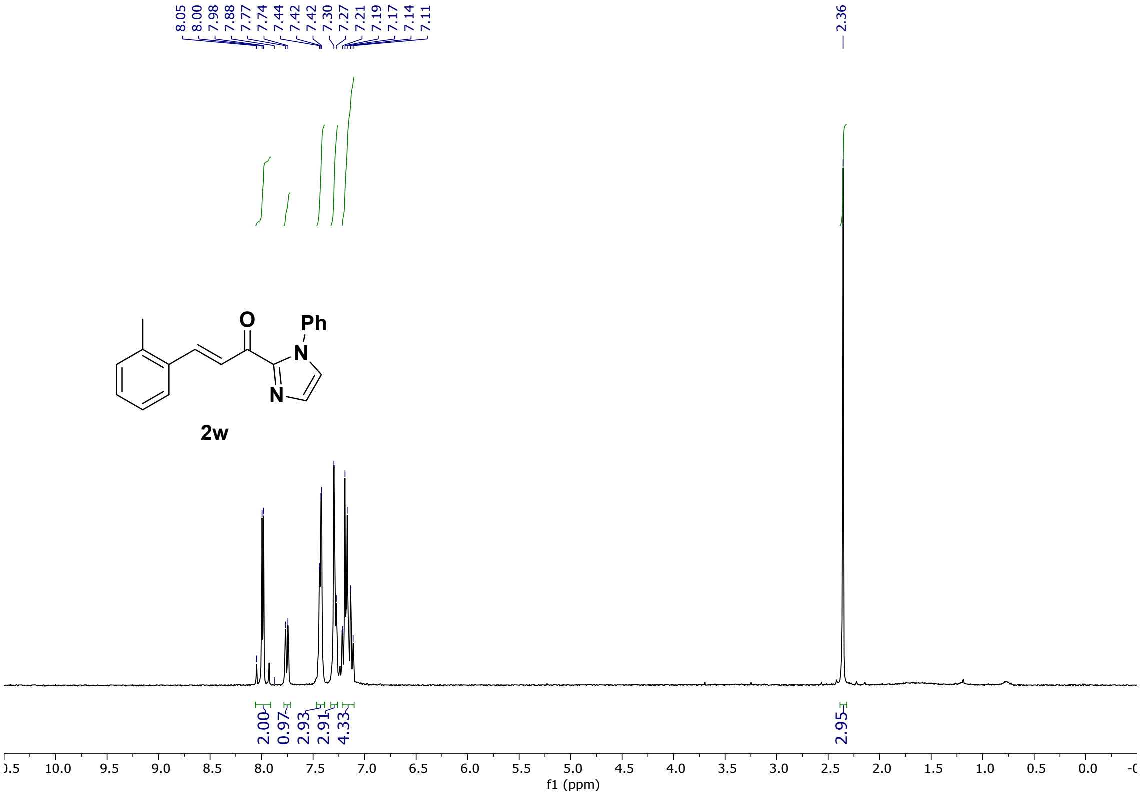

^{13}C NMR of **2w** (75 MHz, $CDCl_3$)

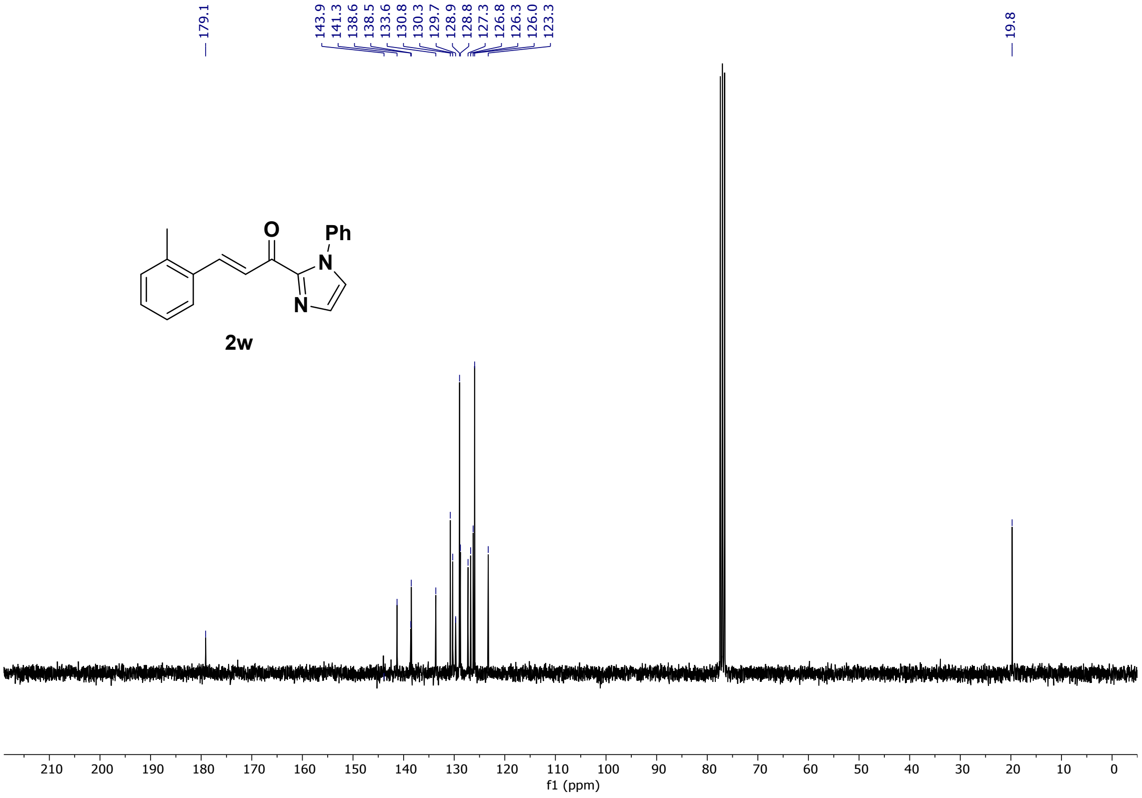

^{1}H NMR of **2x** (300 MHz, $CDCl_3$)

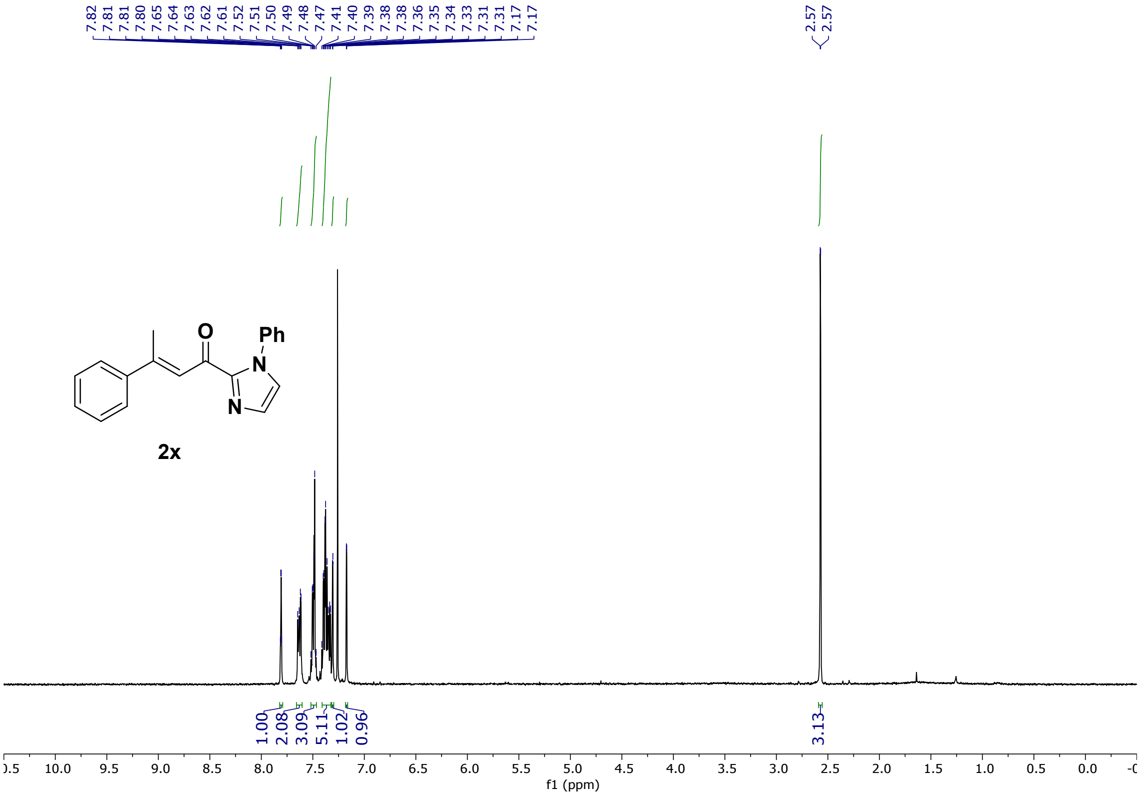

^{13}C NMR of **2x** (75 MHz, $CDCl_3$)

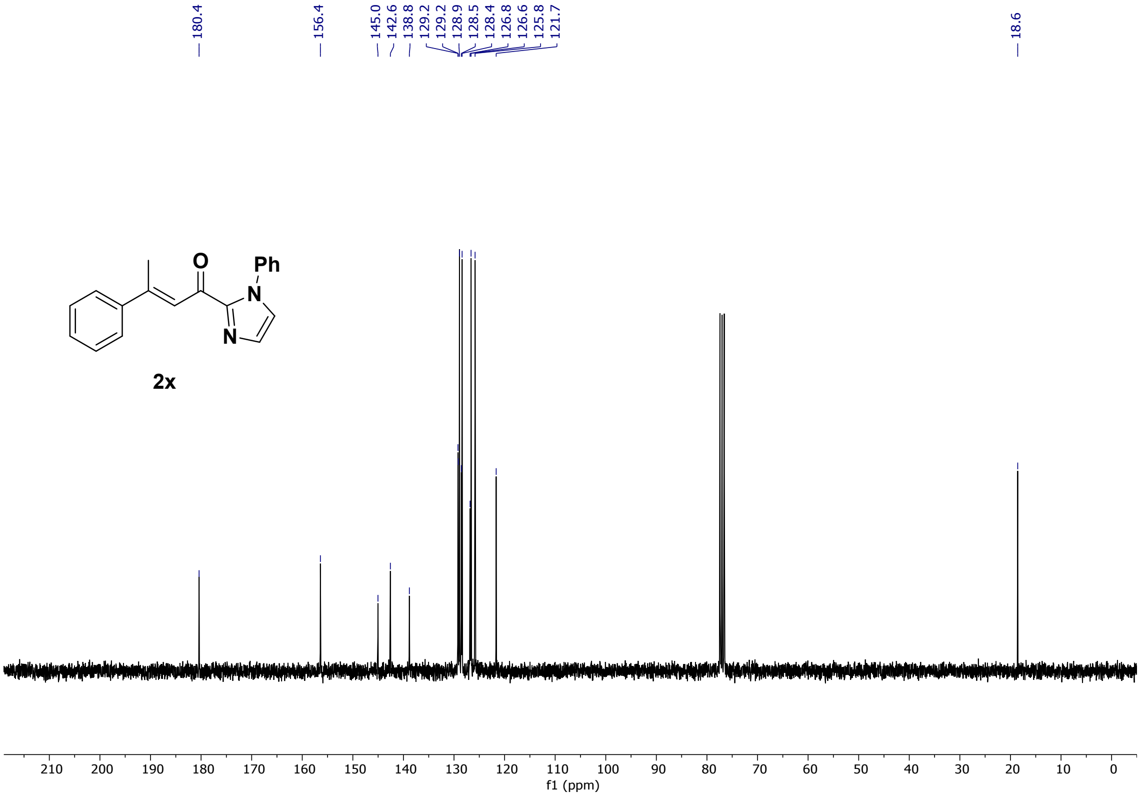

^1H NMR of **2y** (300 MHz, $CDCl_3$)

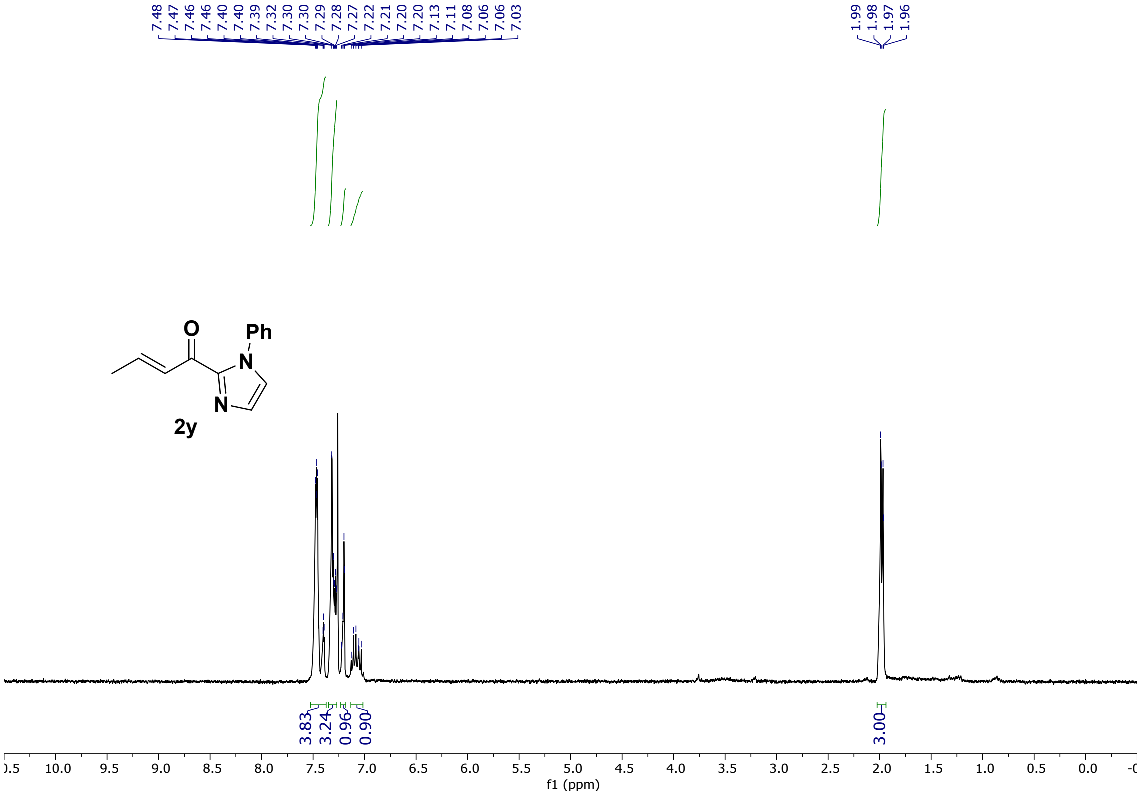

^1H NMR of **2z** (300 MHz, $CDCl_3$)

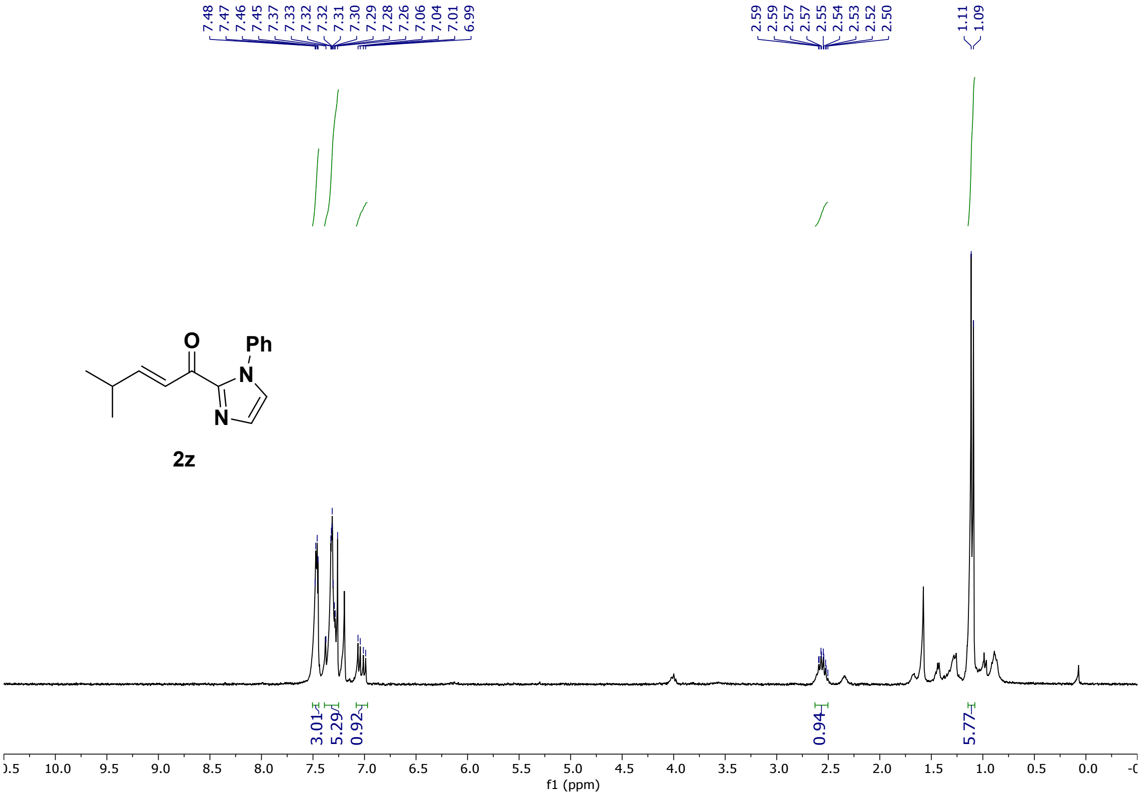

^{1}H NMR of **3a** (300 MHz, $CDCl_3$)

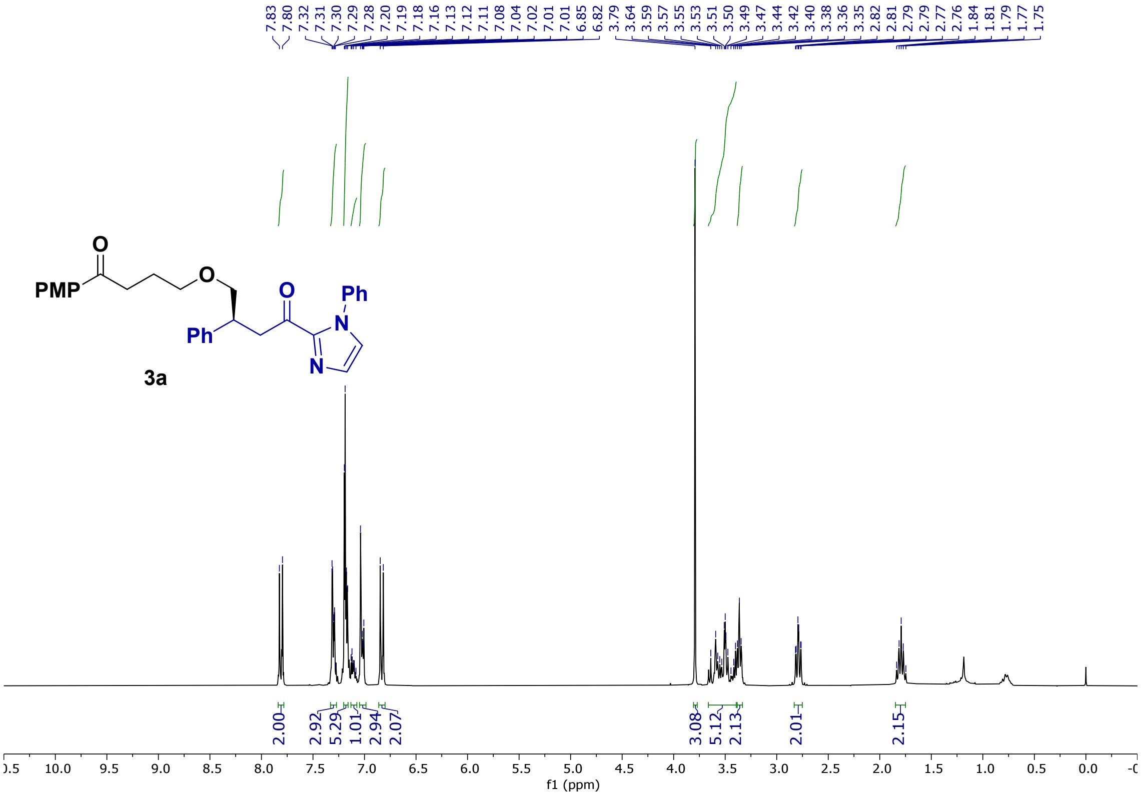

^{13}C NMR of **3a** (75 MHz, $CDCl_3$)

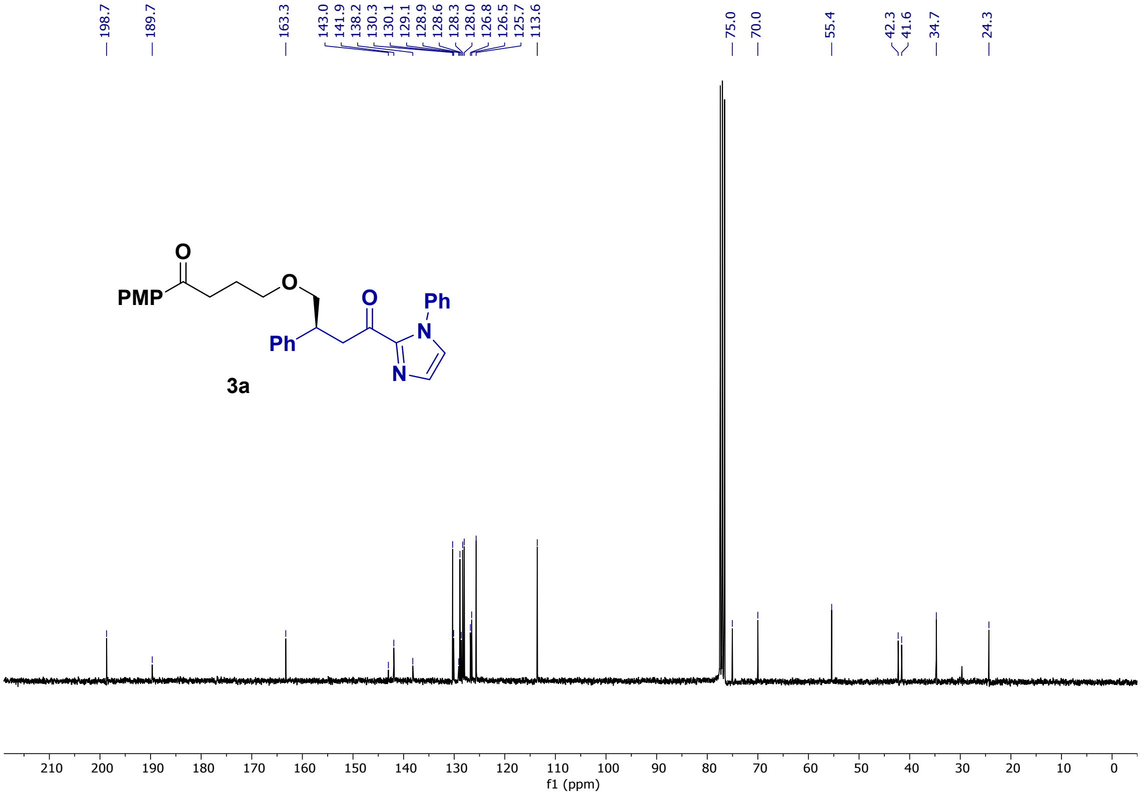

^{1}H NMR of **3b** (300 MHz, $CDCl_3$)

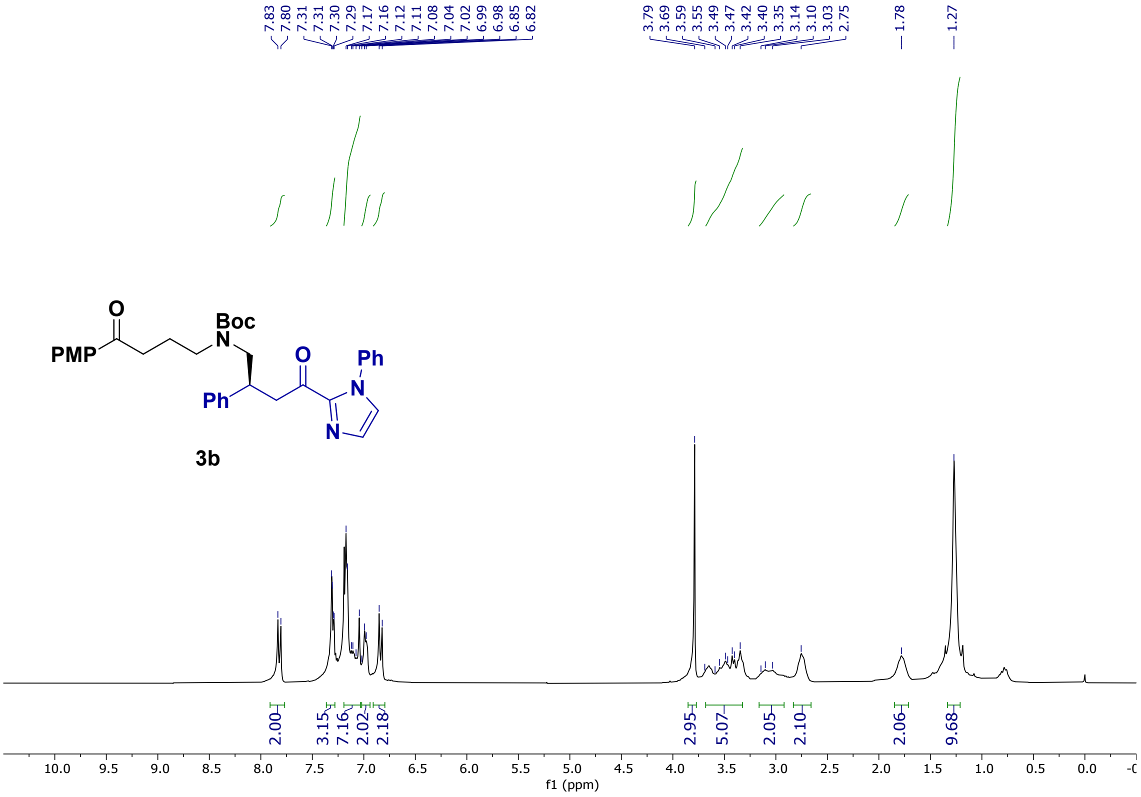

^{13}C NMR of **3b** (75 MHz, $CDCl_3$)

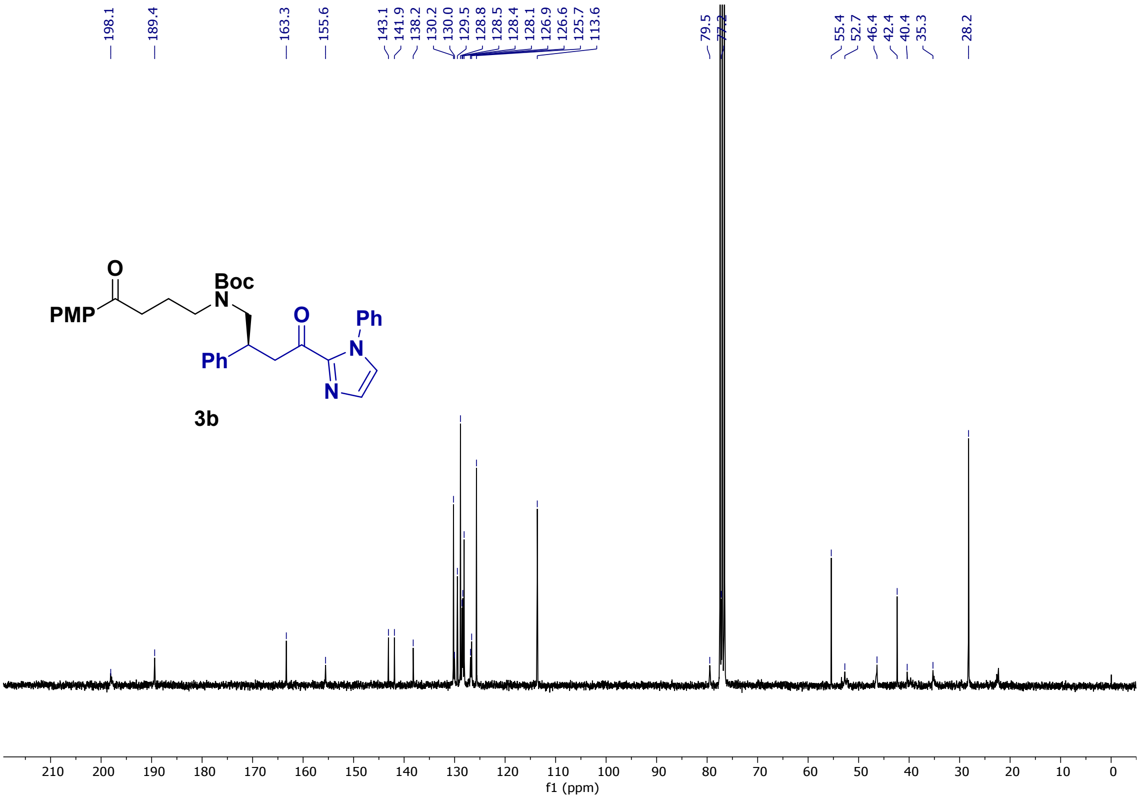

^{1}H NMR of **3c** (300 MHz, $CDCl_3$)

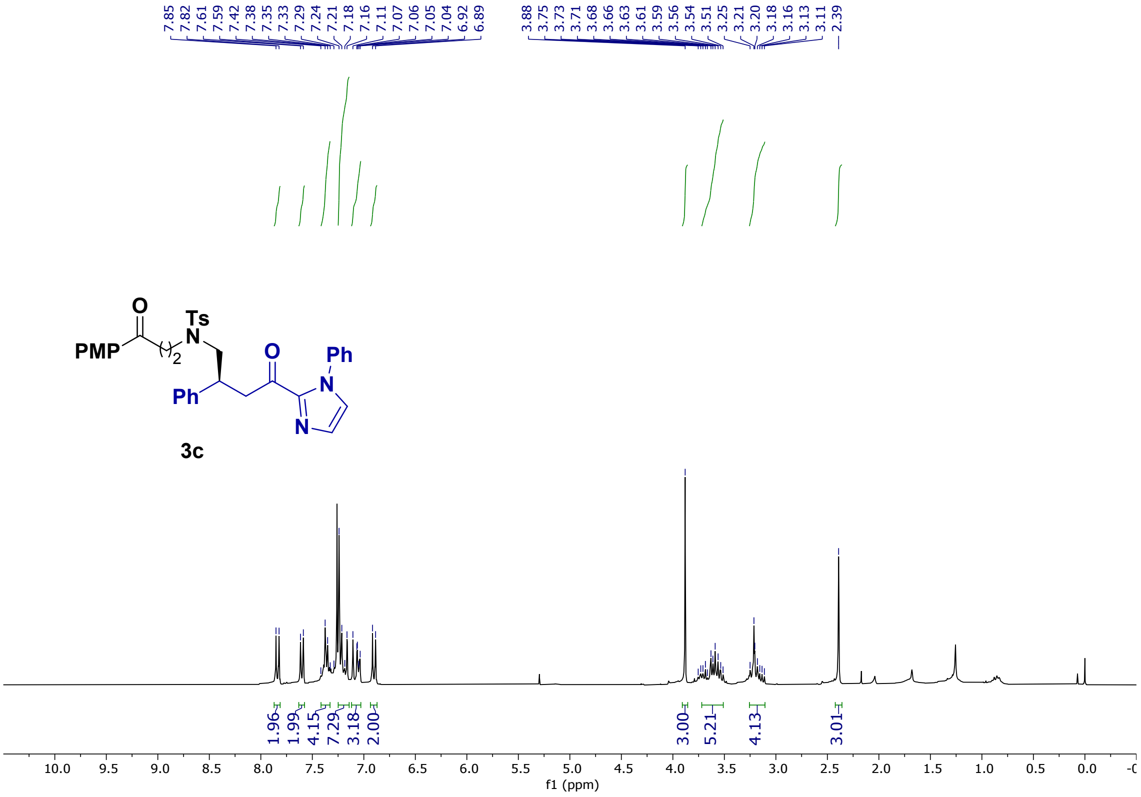

^{13}C NMR of **3c** (75 MHz, $CDCl_3$)

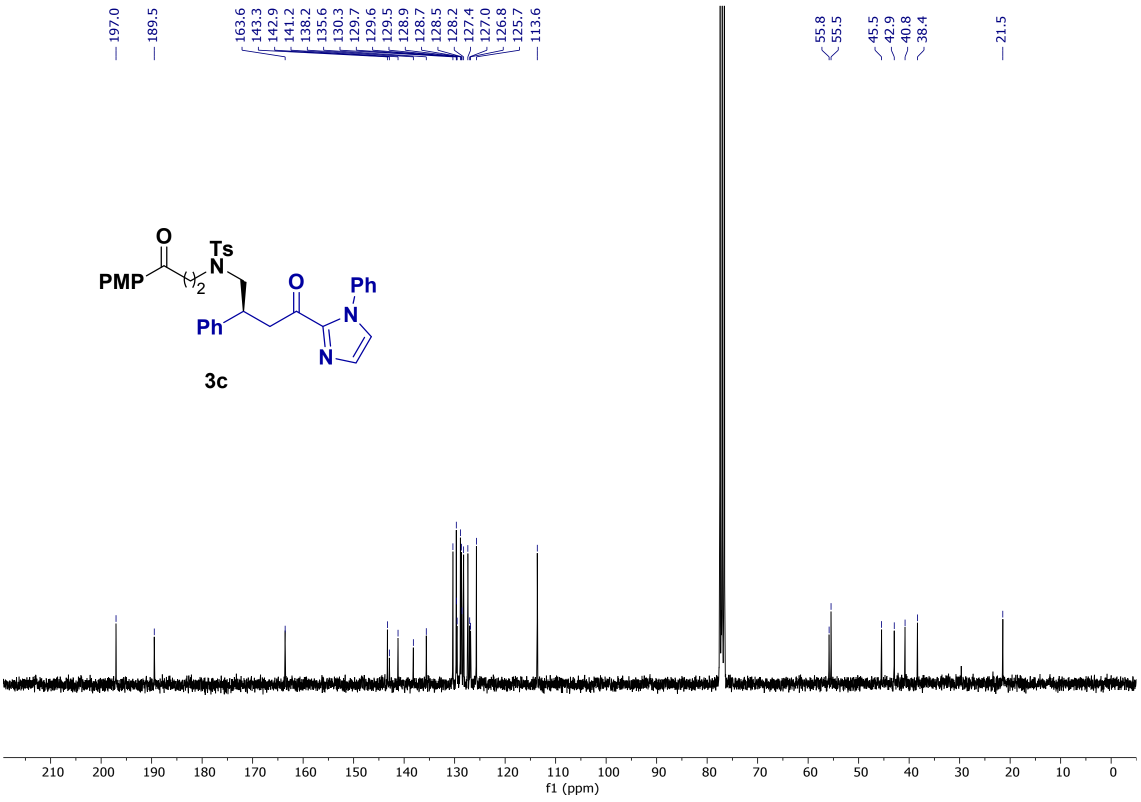

^{1}H NMR of **3d** (300 MHz, CDCl$_3$)

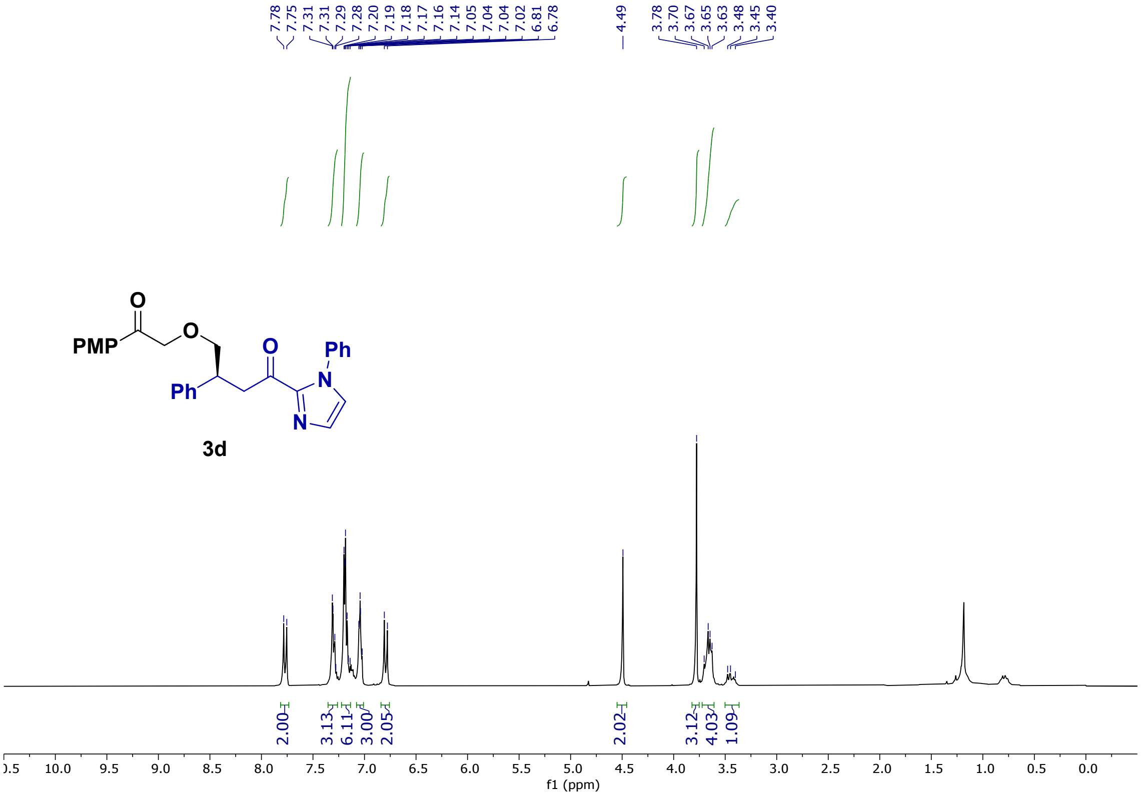

^{13}C NMR of **3d** (75 MHz, CDCl$_3$)

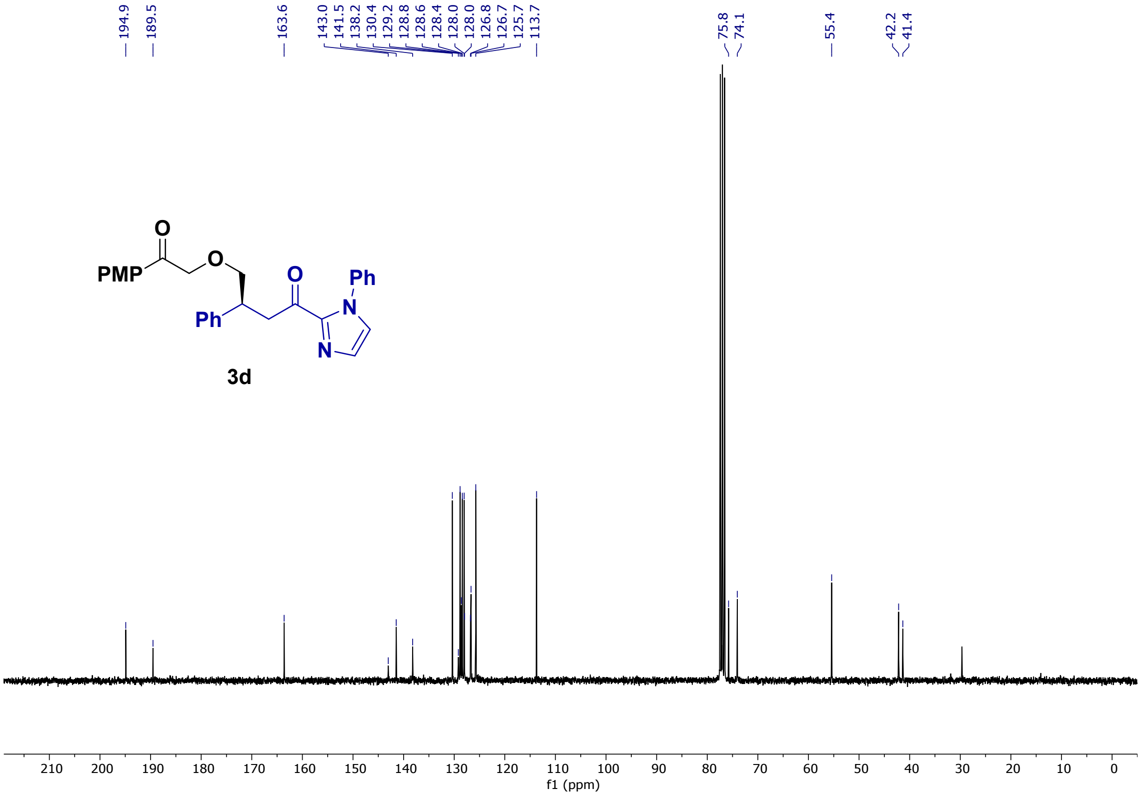

^{1}H NMR of **3e** (300 MHz, $CDCl_3$)

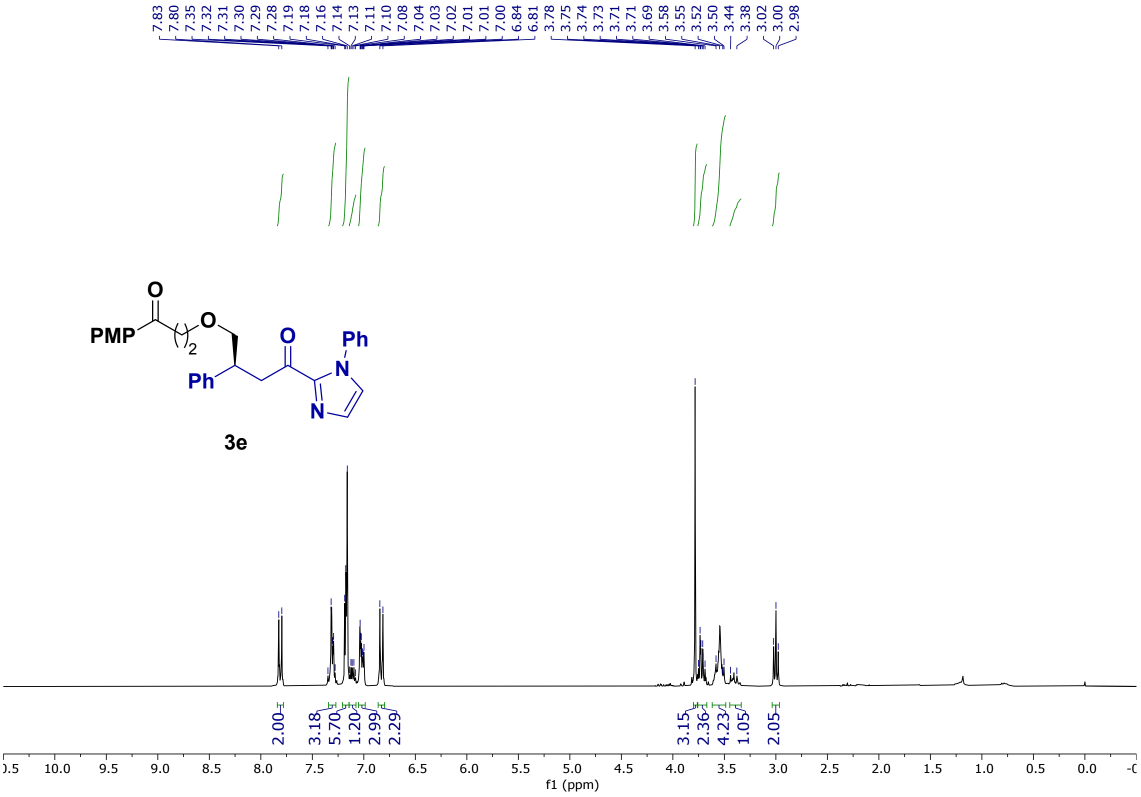

^{13}C NMR of **3e** (75 MHz, $CDCl_3$)

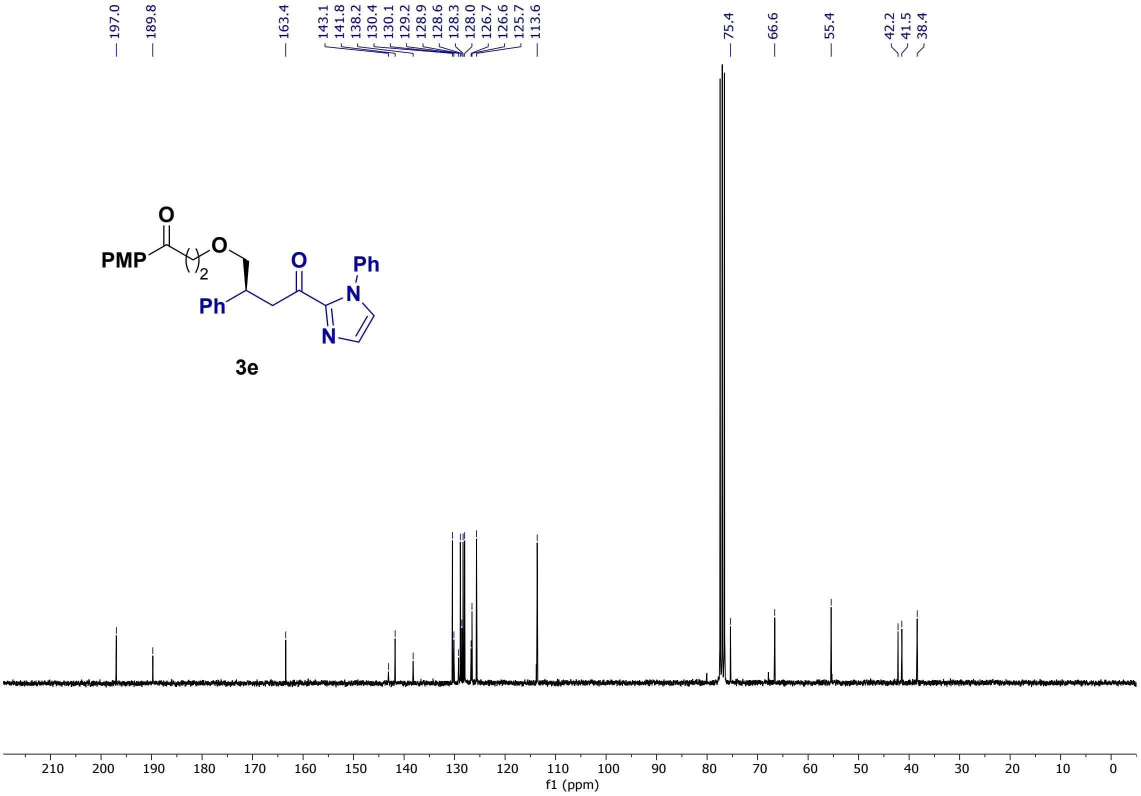

^{1}H NMR of **3f** (300 MHz, $CDCl_3$)

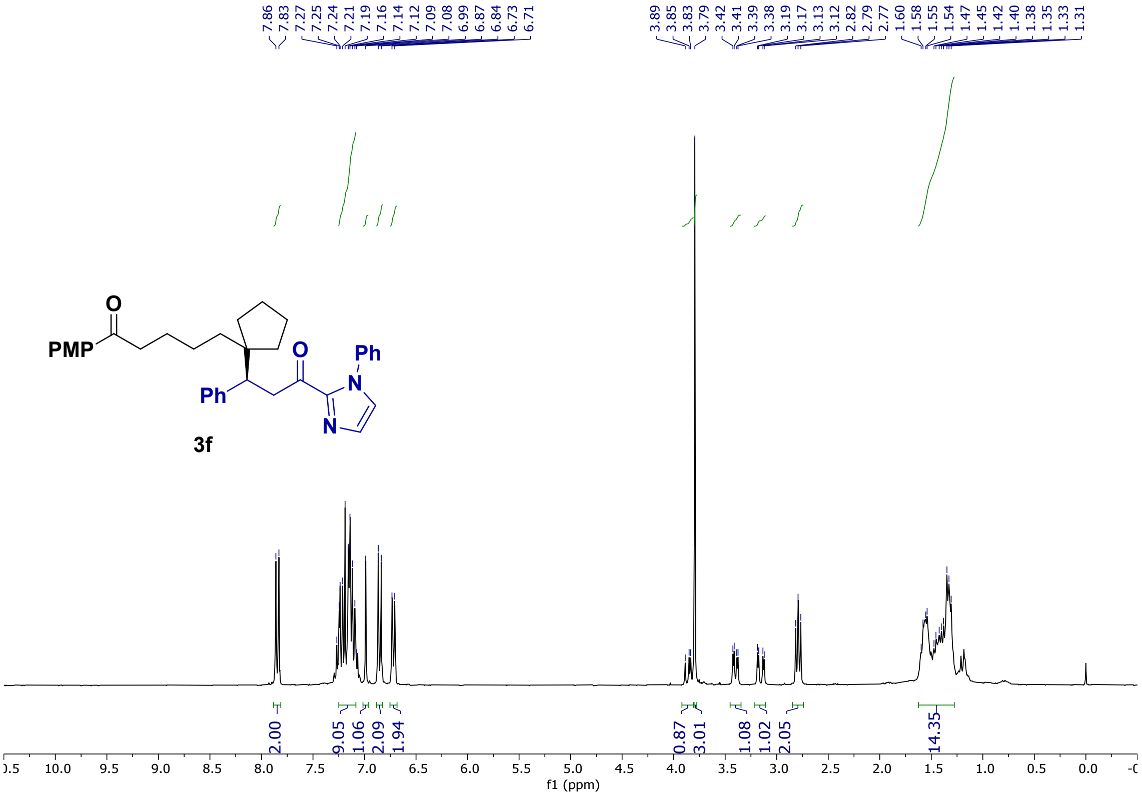

^{13}C NMR of **3f** (75 MHz, $CDCl_3$)

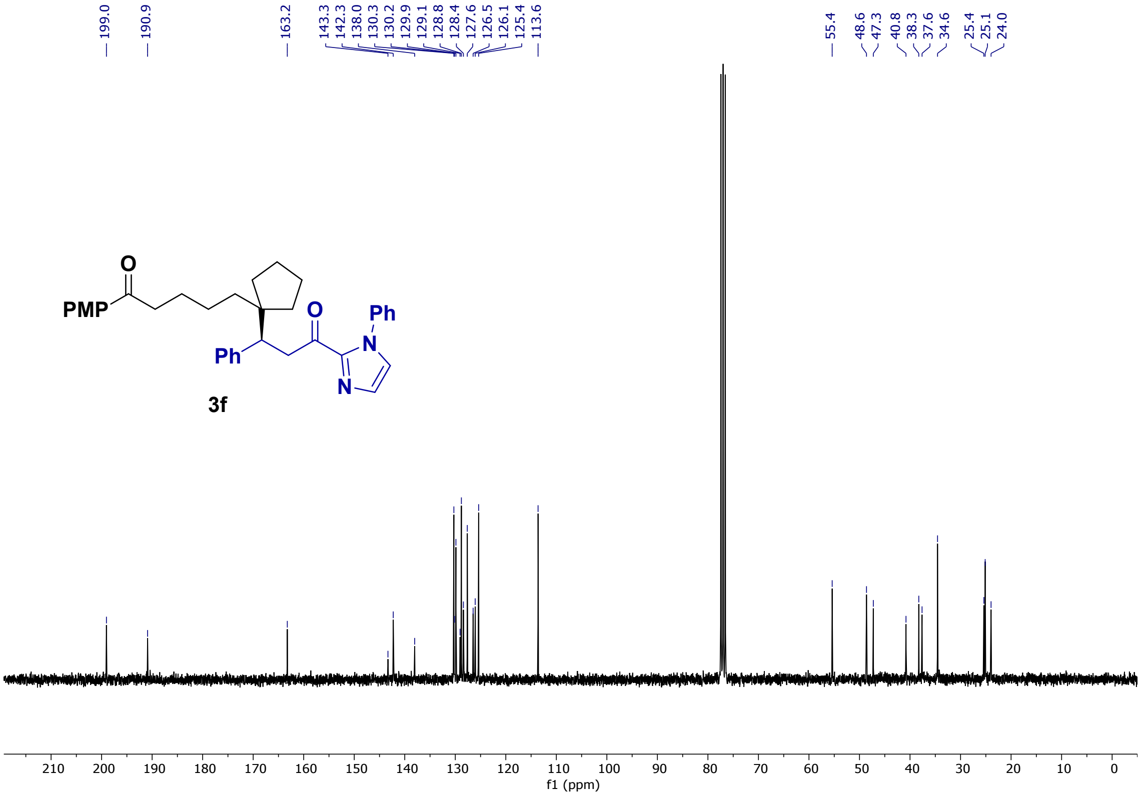

^1H NMR of **3g** (300 MHz, $CDCl_3$)

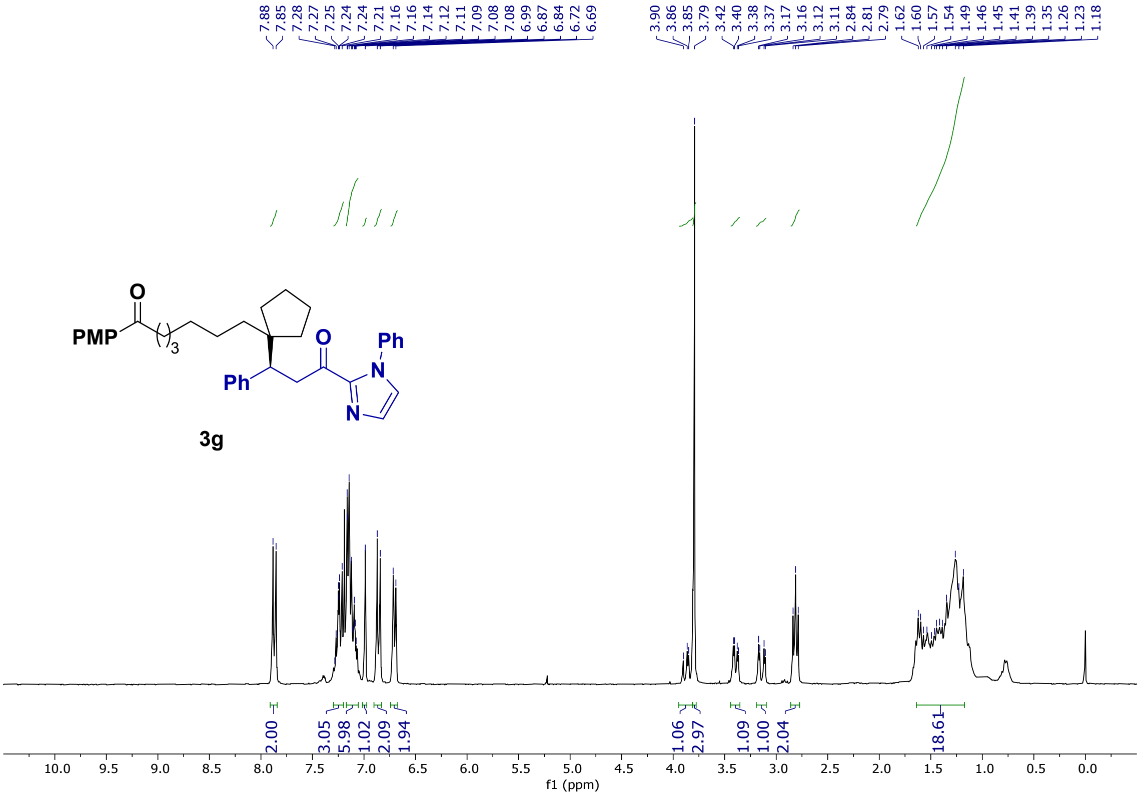

^{13}C NMR of **3g** (75 MHz, $CDCl_3$)

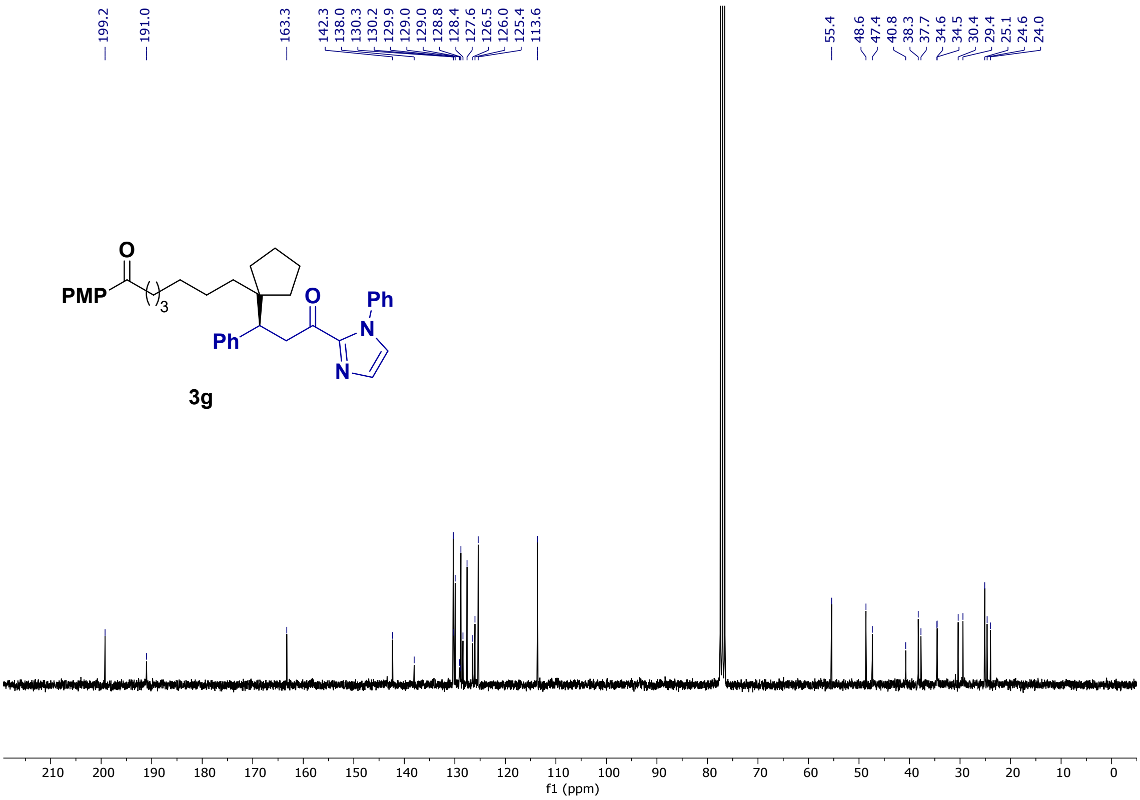

^{1}H NMR of **3h** (300 MHz, $CDCl_3$)

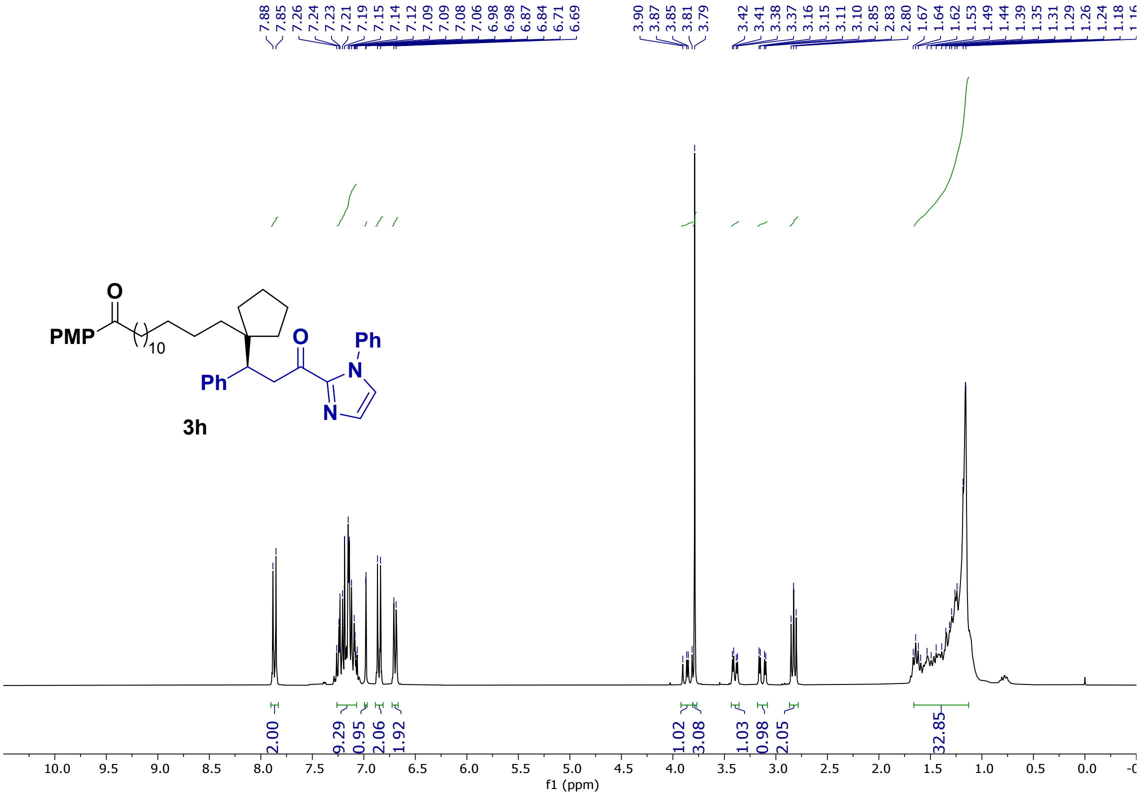

^{13}C NMR of **3h** (75 MHz, $CDCl_3$)

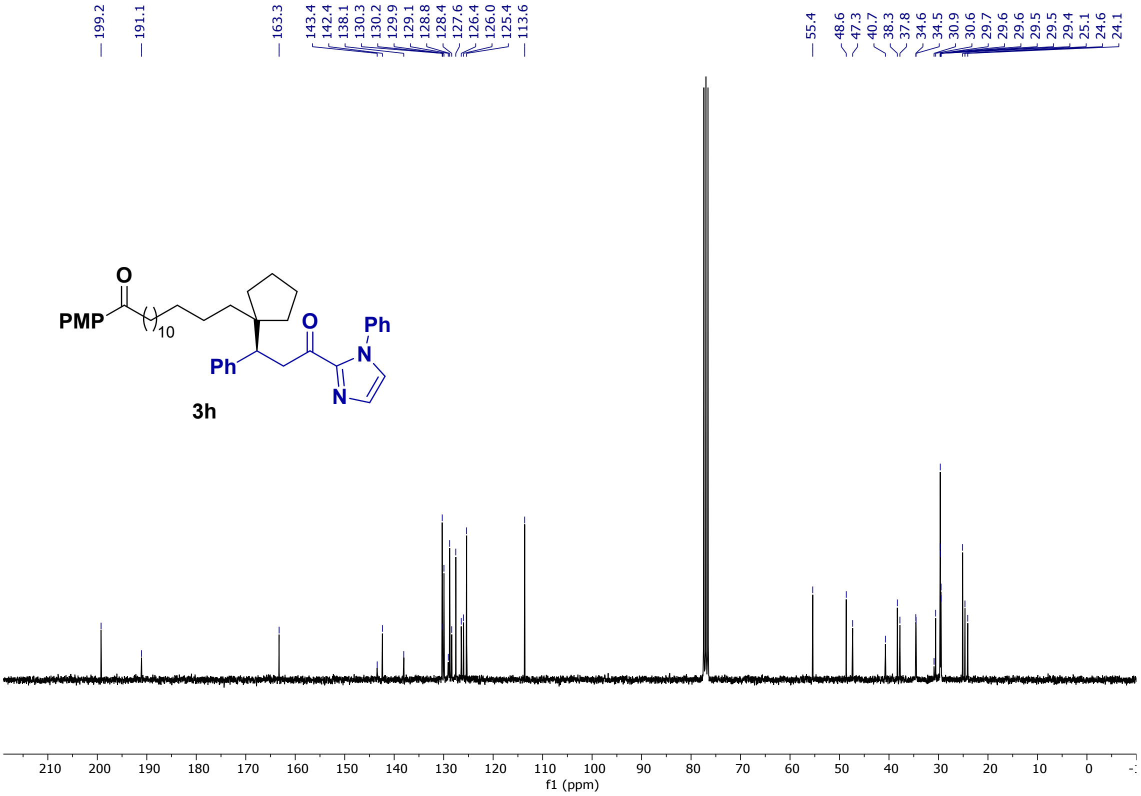

^{1}H NMR of **3i** (300 MHz, $CDCl_3$)

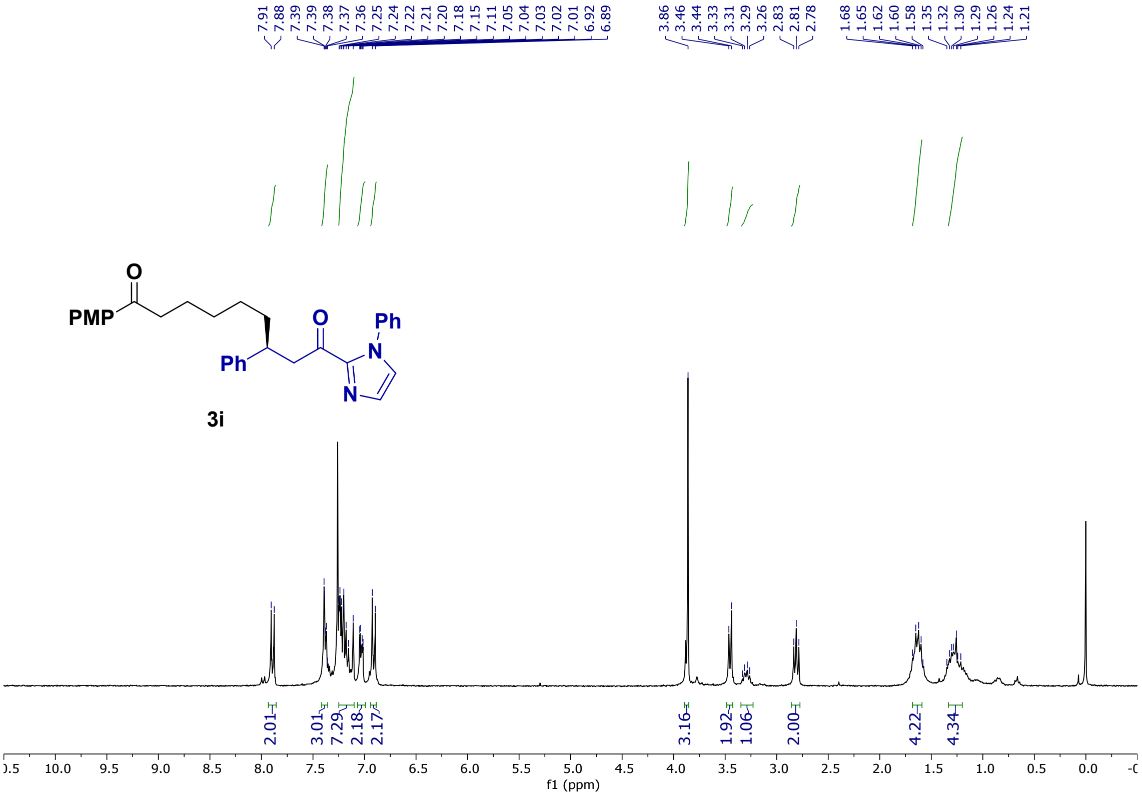

^{13}C NMR of **3i** (75 MHz, $CDCl_3$)

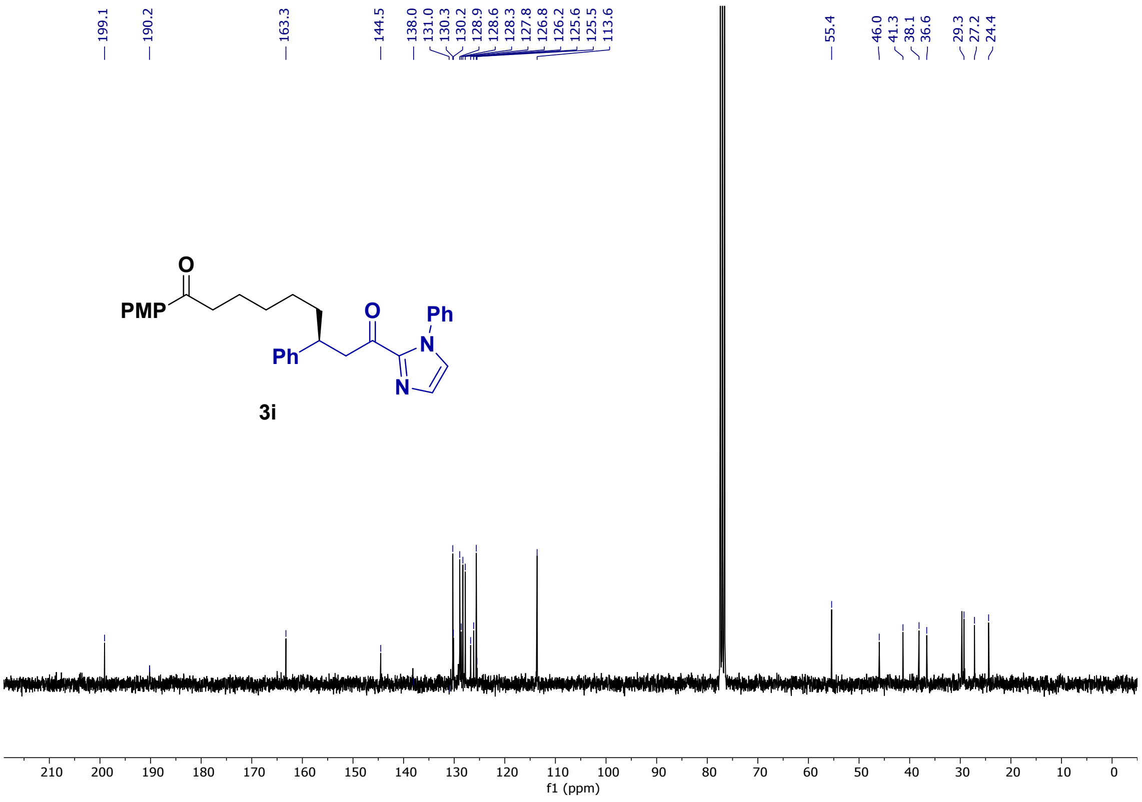

^{1}H NMR of **3j** (300 MHz, $CDCl_3$)

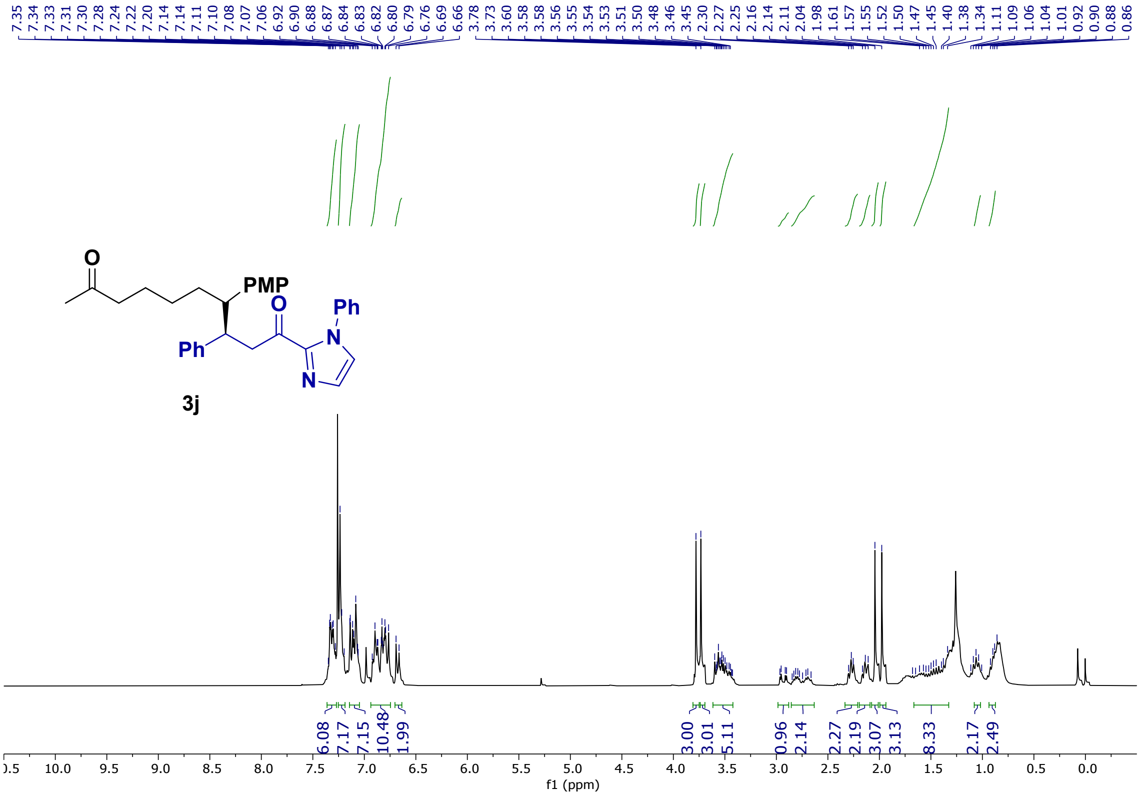

^{13}C NMR of **3i** (75 MHz, $CDCl_3$)

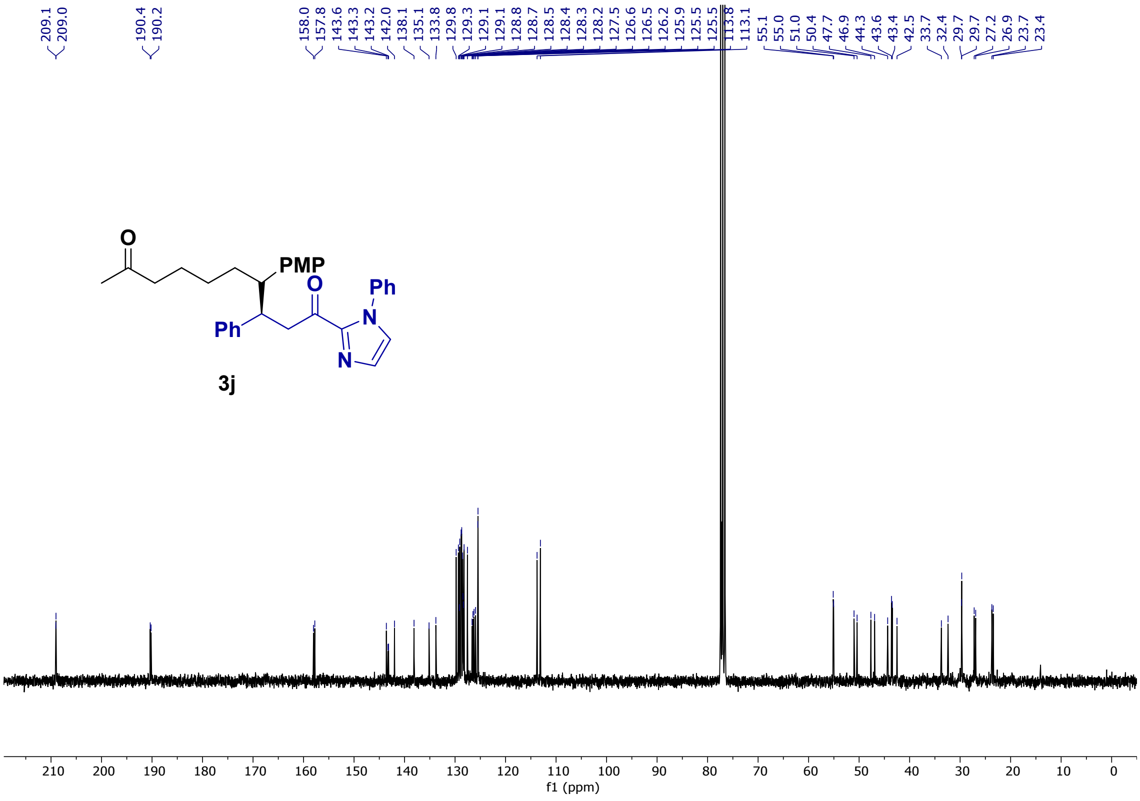

^{1}H NMR of **3k** (300 MHz, $CDCl_3$)

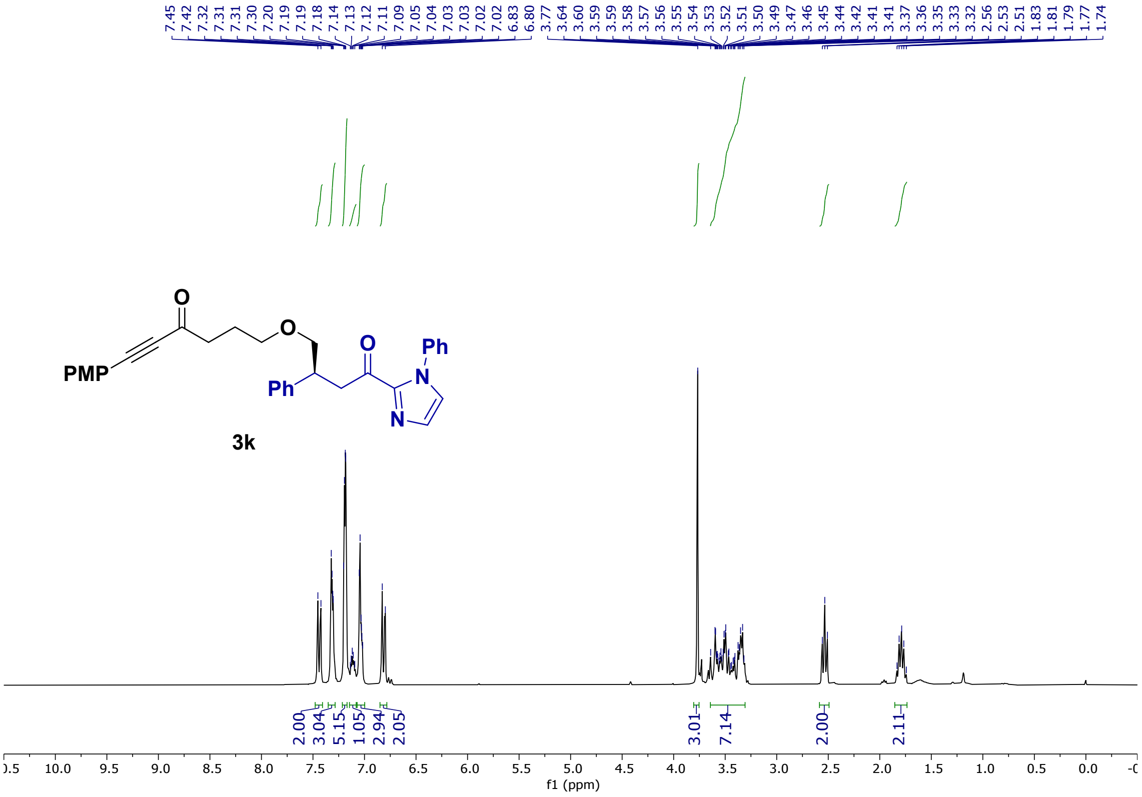

^{13}C NMR of **3k** (75 MHz, $CDCl_3$)

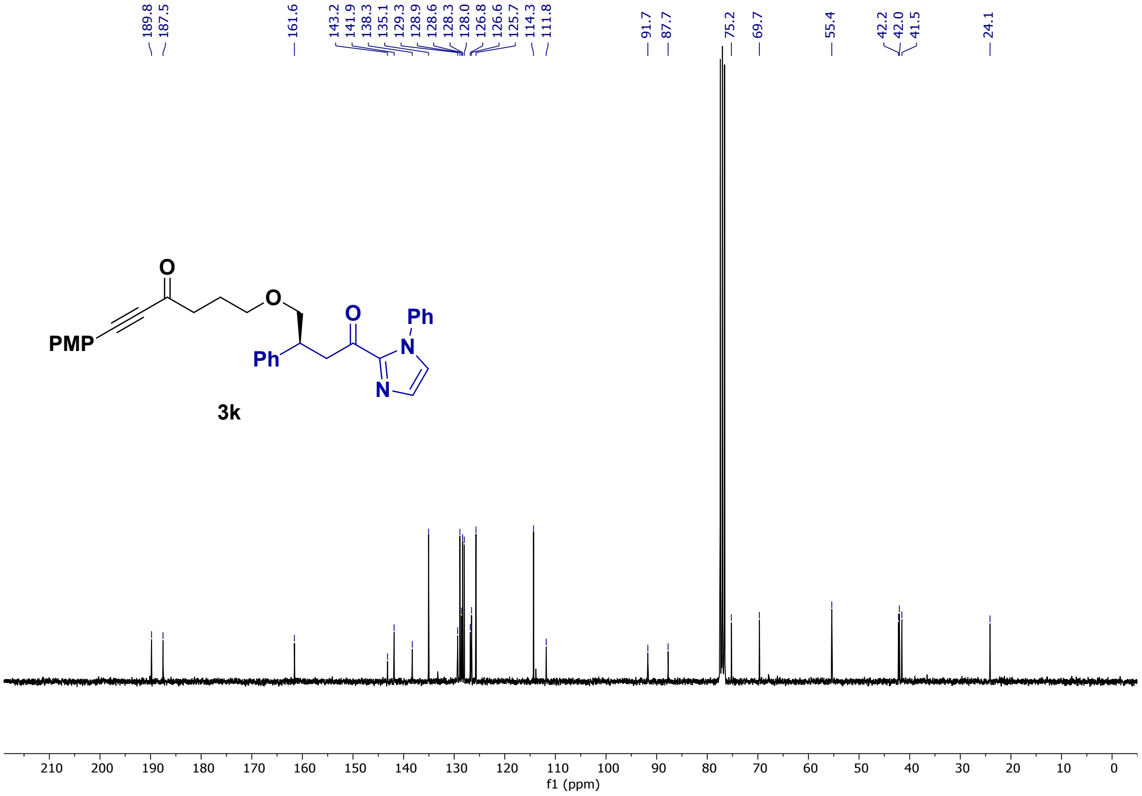

^{1}H NMR of **3l** (300 MHz, $CDCl_3$)

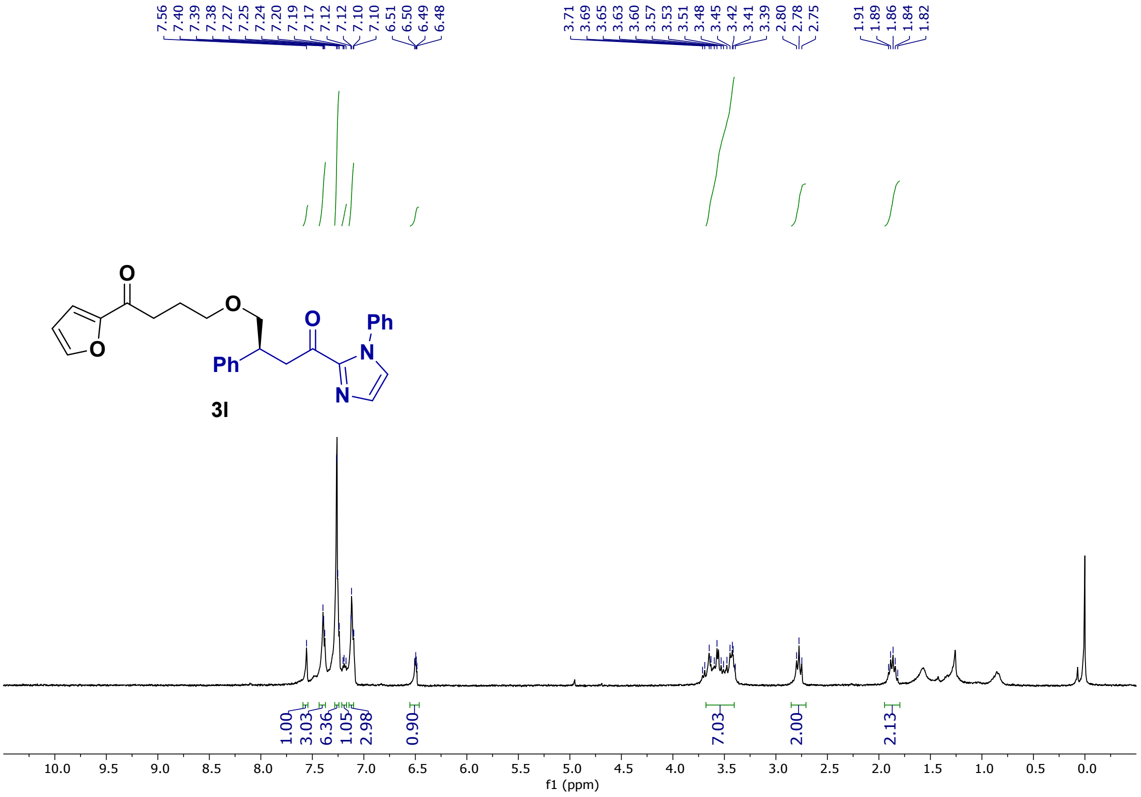

3l

^{13}C NMR of **3l** (75 MHz, $CDCl_3$)

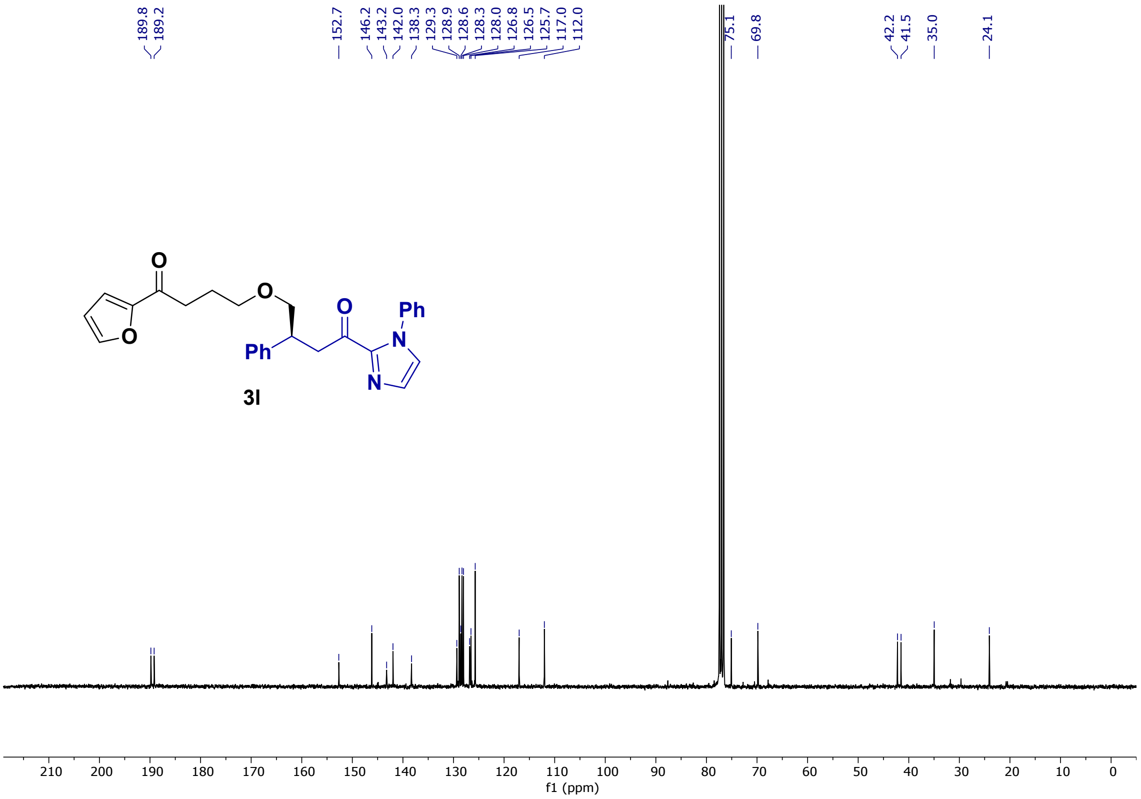

3l

^{1}H NMR of **3m** (300 MHz, $CDCl_3$)

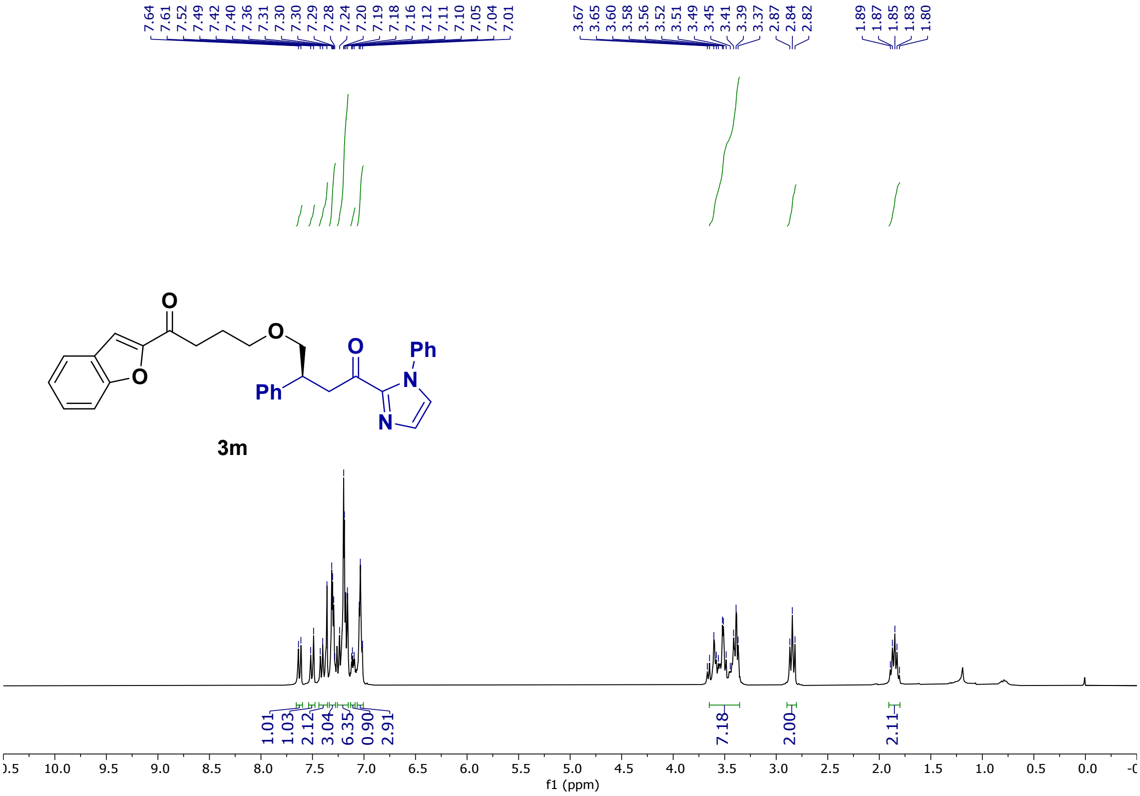

^{13}C NMR of **3m** (75 MHz, $CDCl_3$)

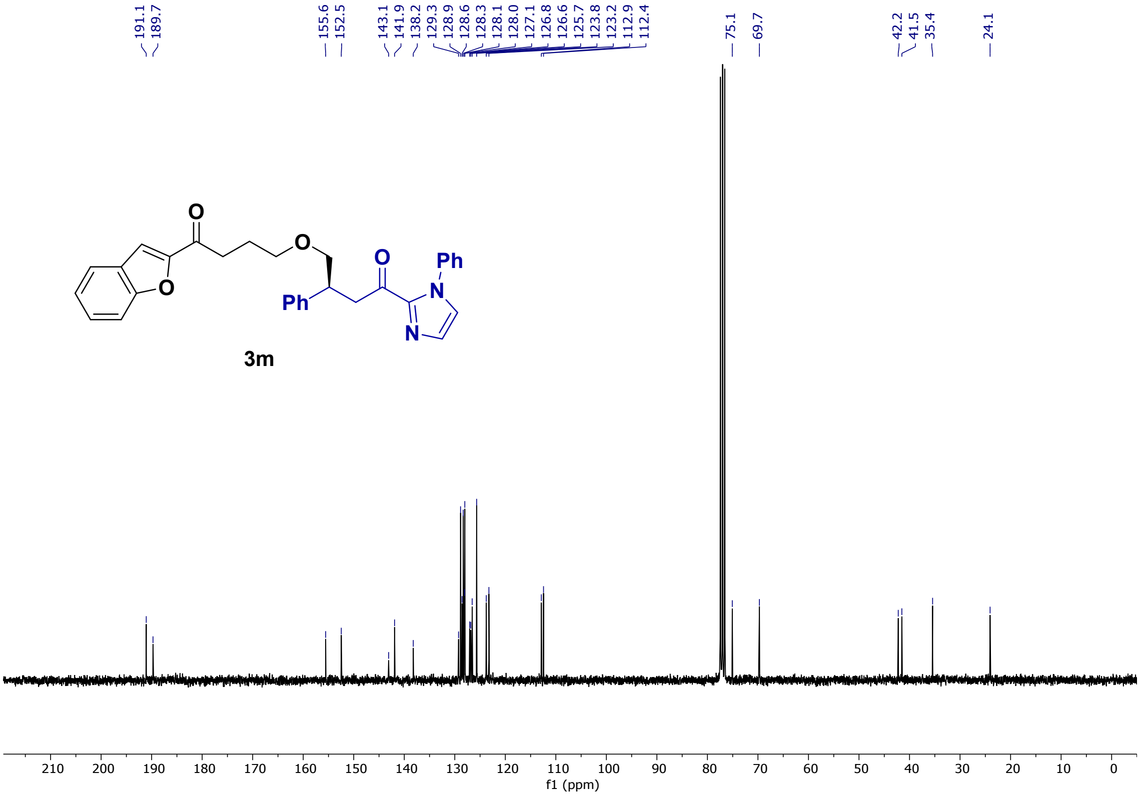

^{1}H NMR of **3n** (300 MHz, $CDCl_3$)

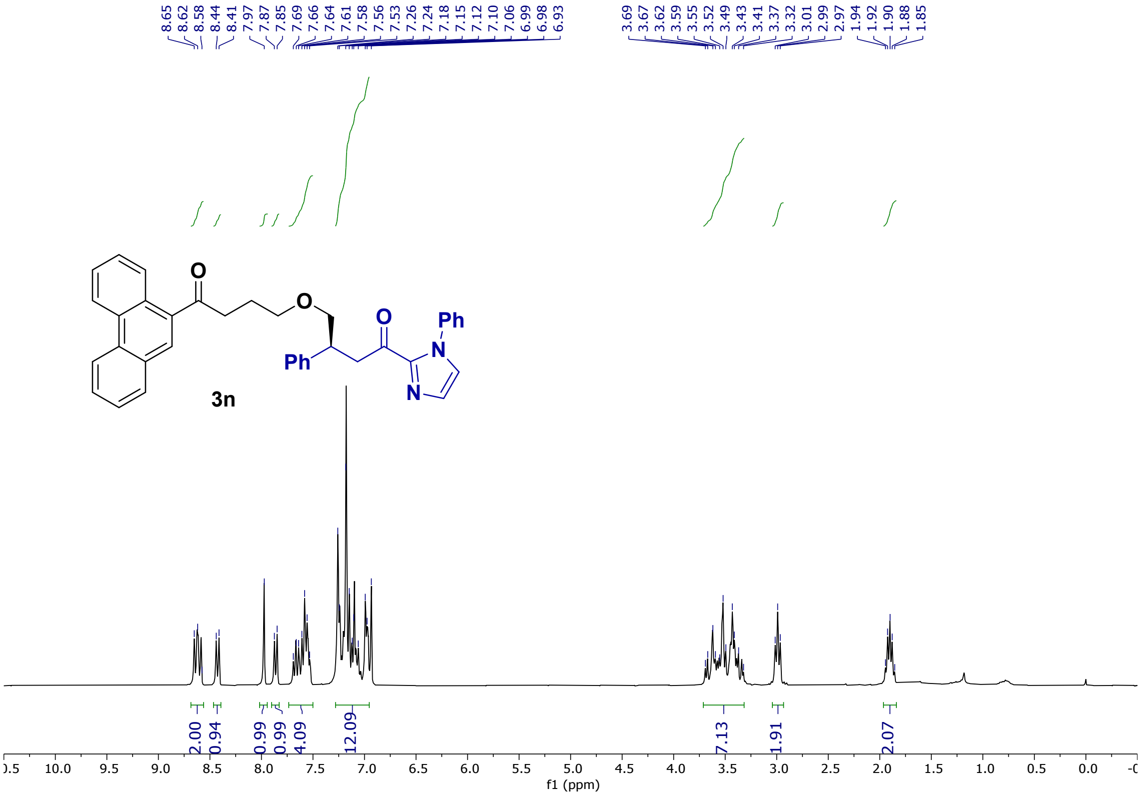

^{13}C NMR of **3n** (75 MHz, $CDCl_3$)

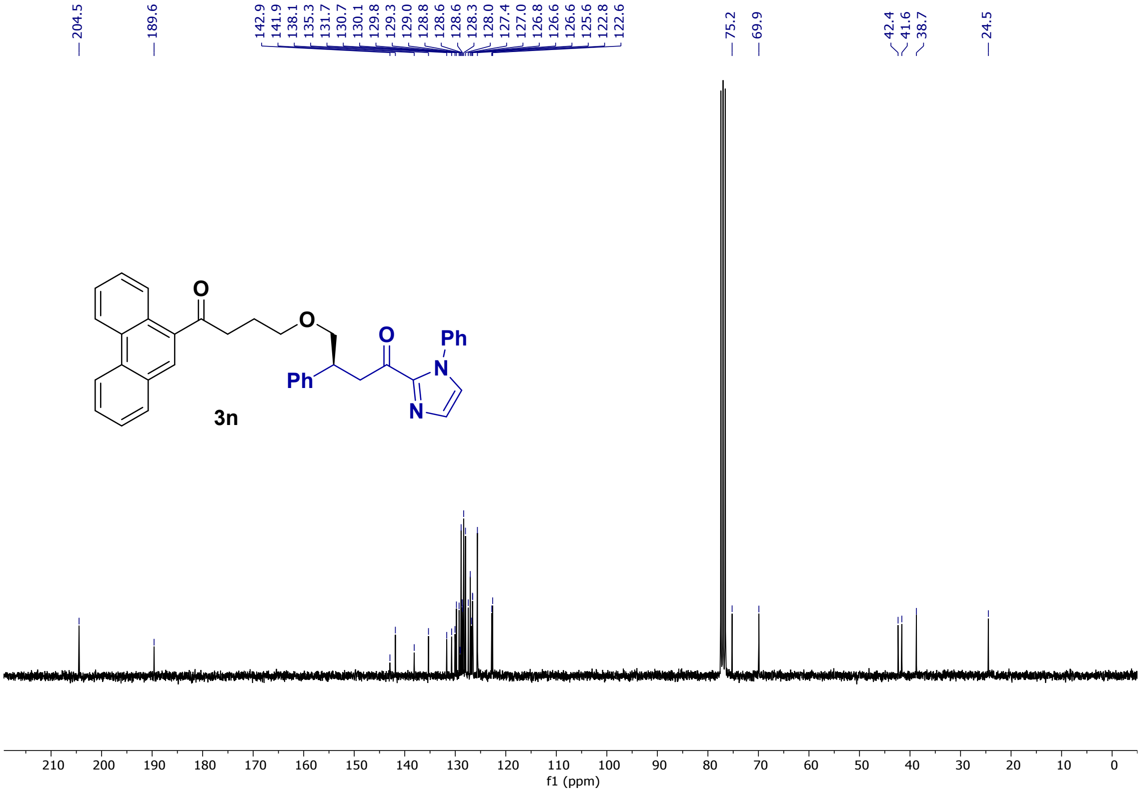

^{1}H NMR of **3o** (300 MHz, $CDCl_3$)

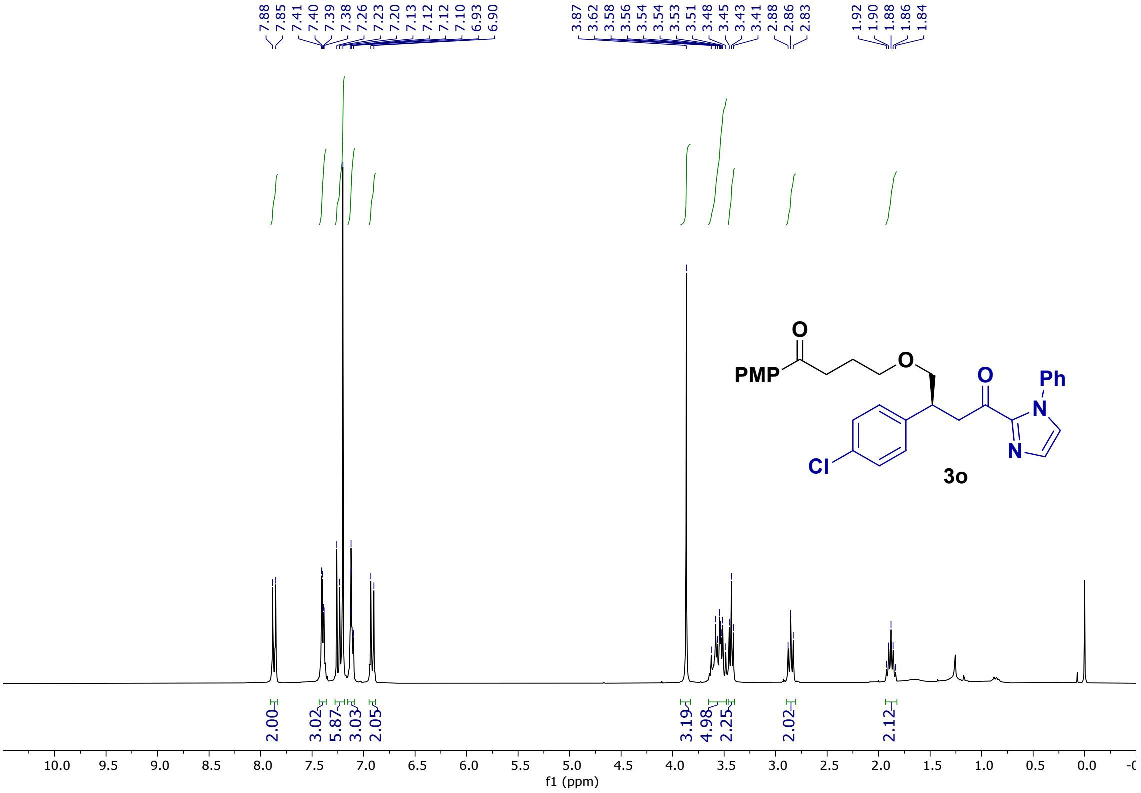

^{13}C NMR of **3o** (75 MHz, $CDCl_3$)

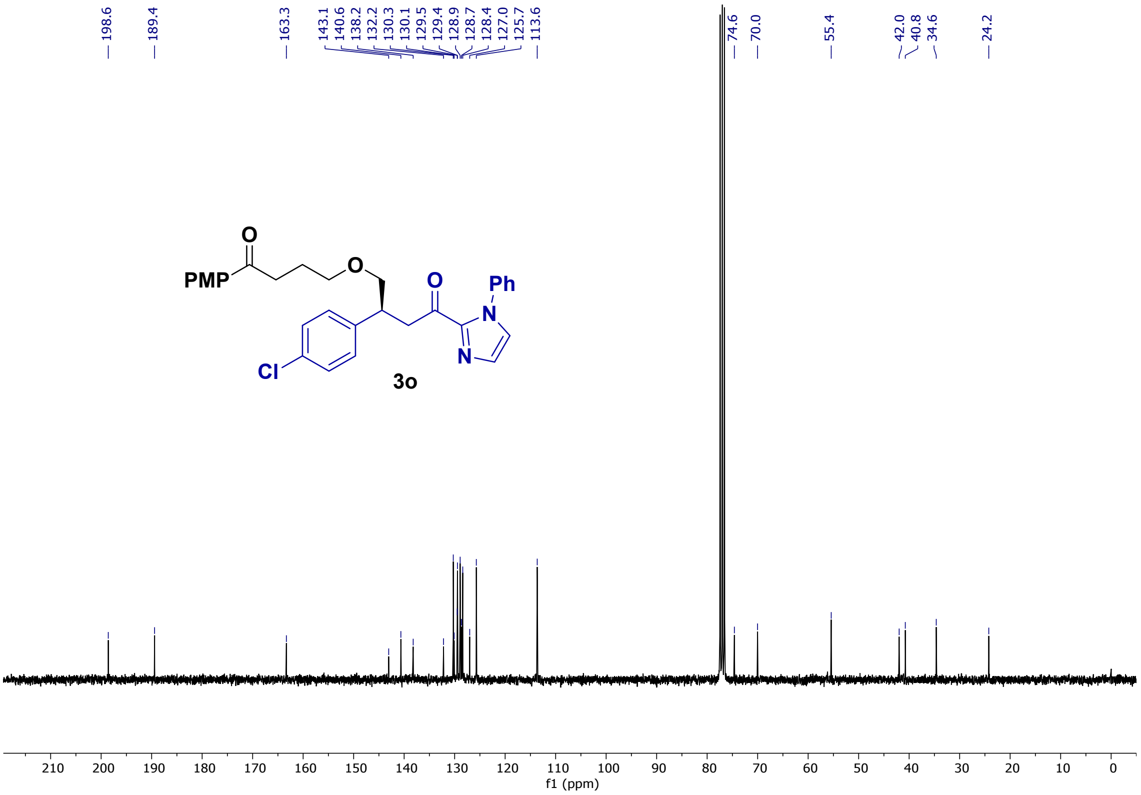

^{1}H NMR of **3p** (300 MHz, $CDCl_3$)

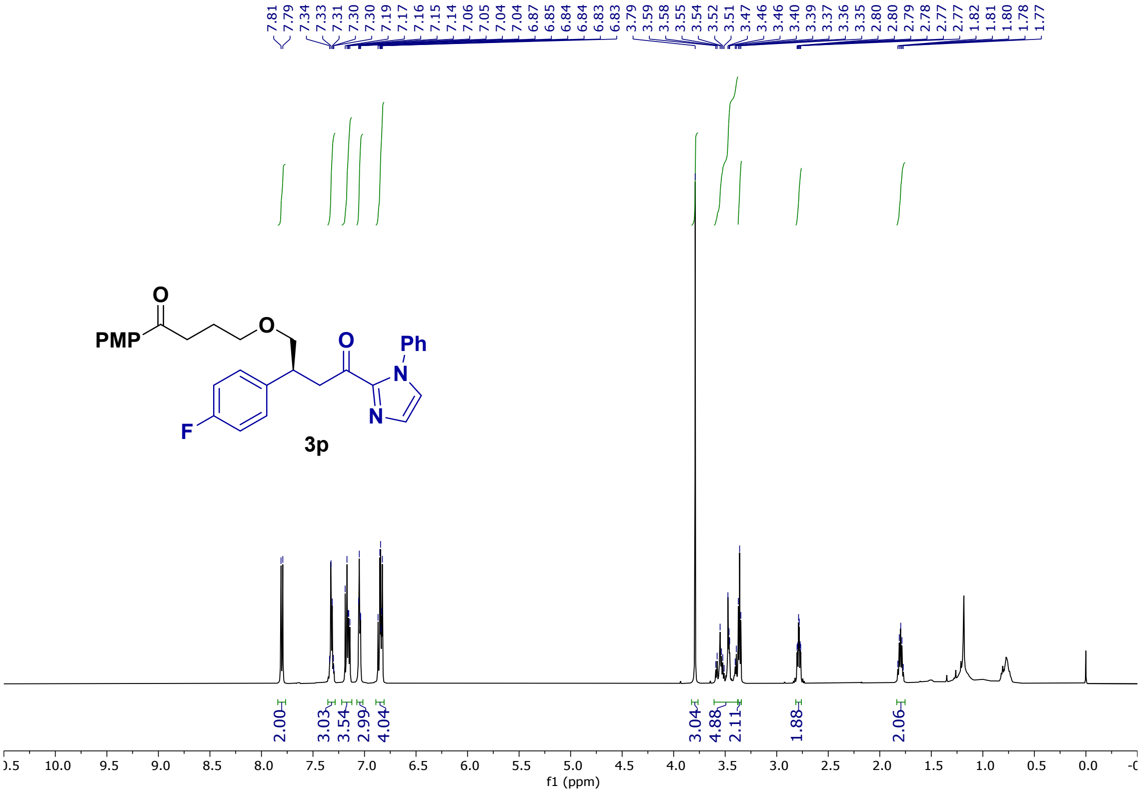

^{13}C NMR of **3p** (75 MHz, $CDCl_3$)

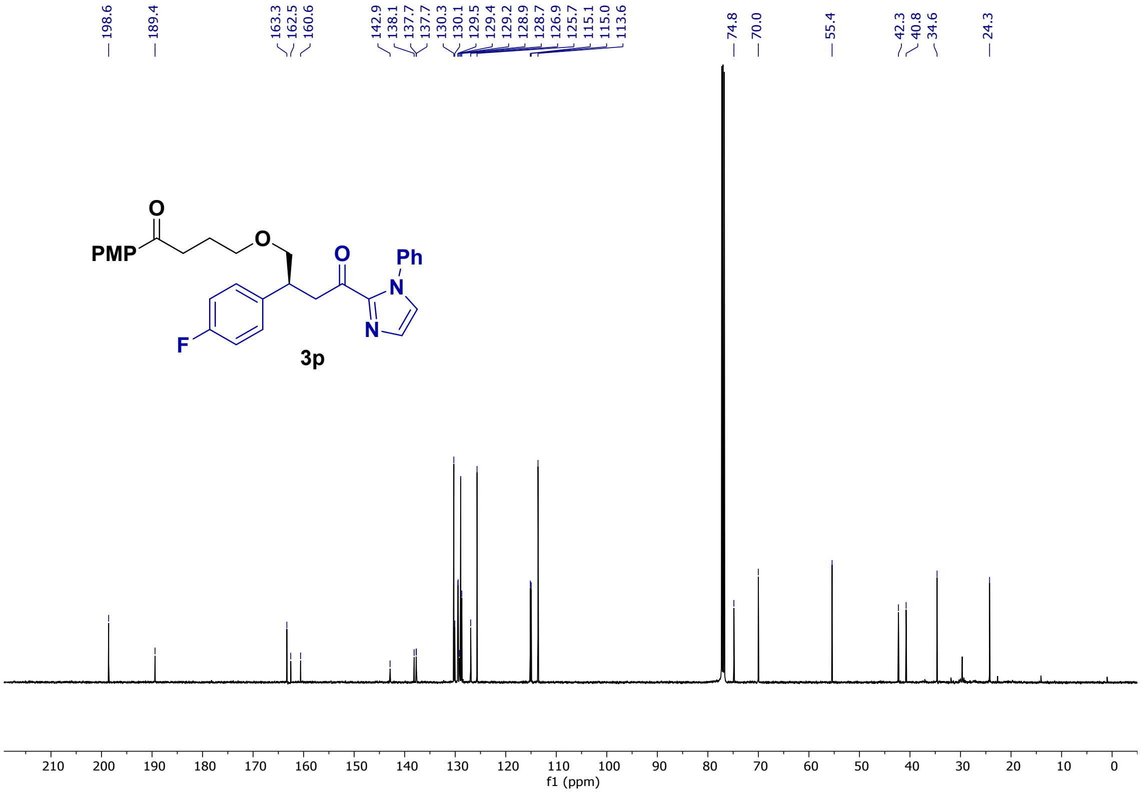

^{19}F NMR of **3p** (471 MHz, $CDCl_3$)

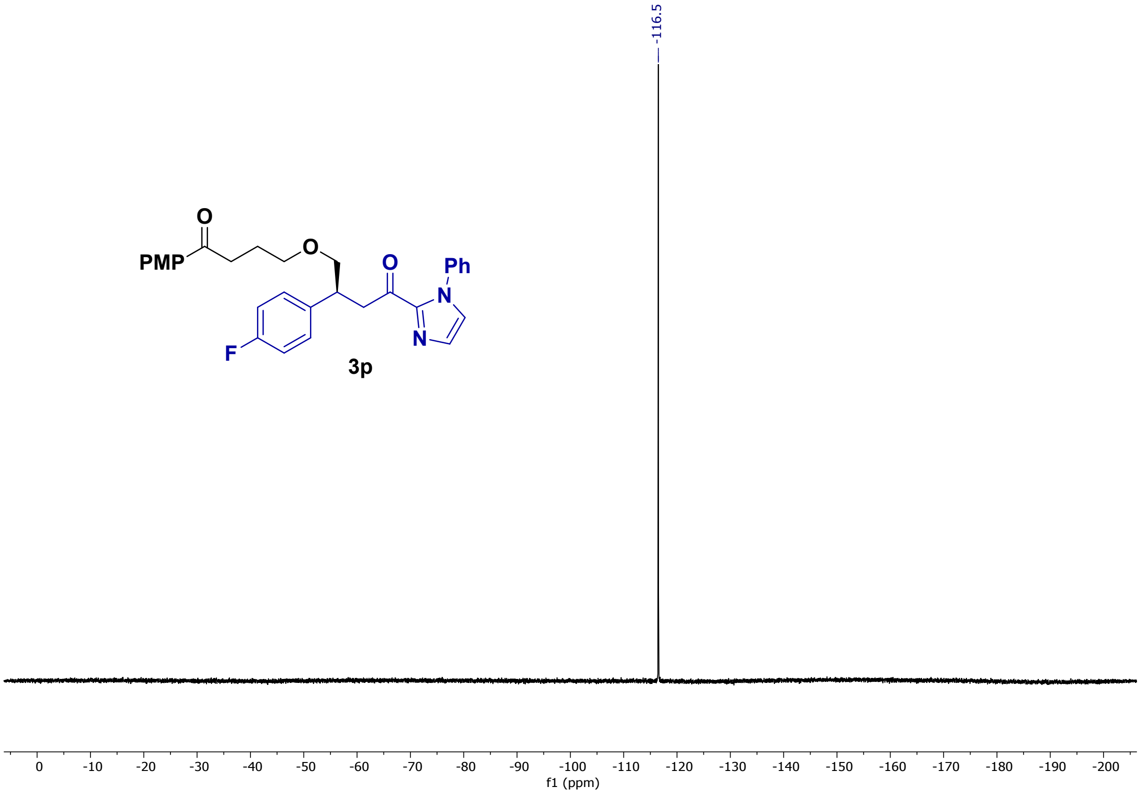

^{1}H NMR of **3q** (300 MHz, $CDCl_3$)

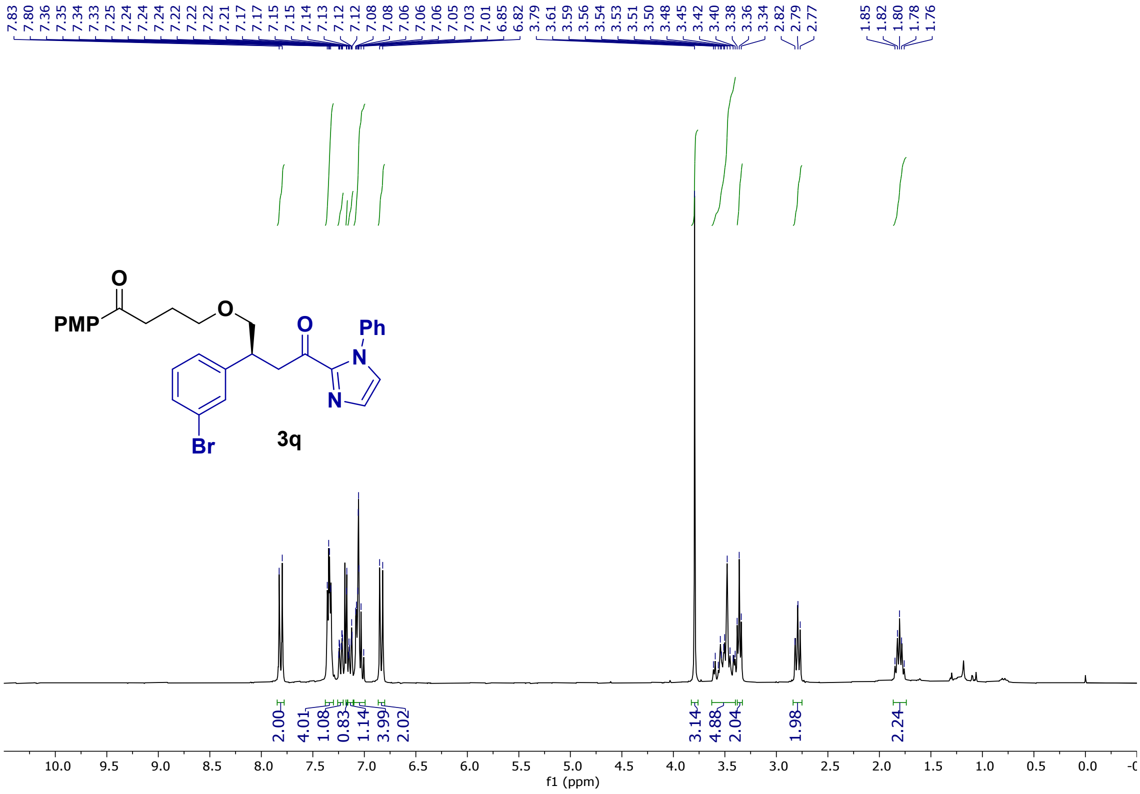

^{13}C NMR of **3q** (75 MHz, $CDCl_3$)

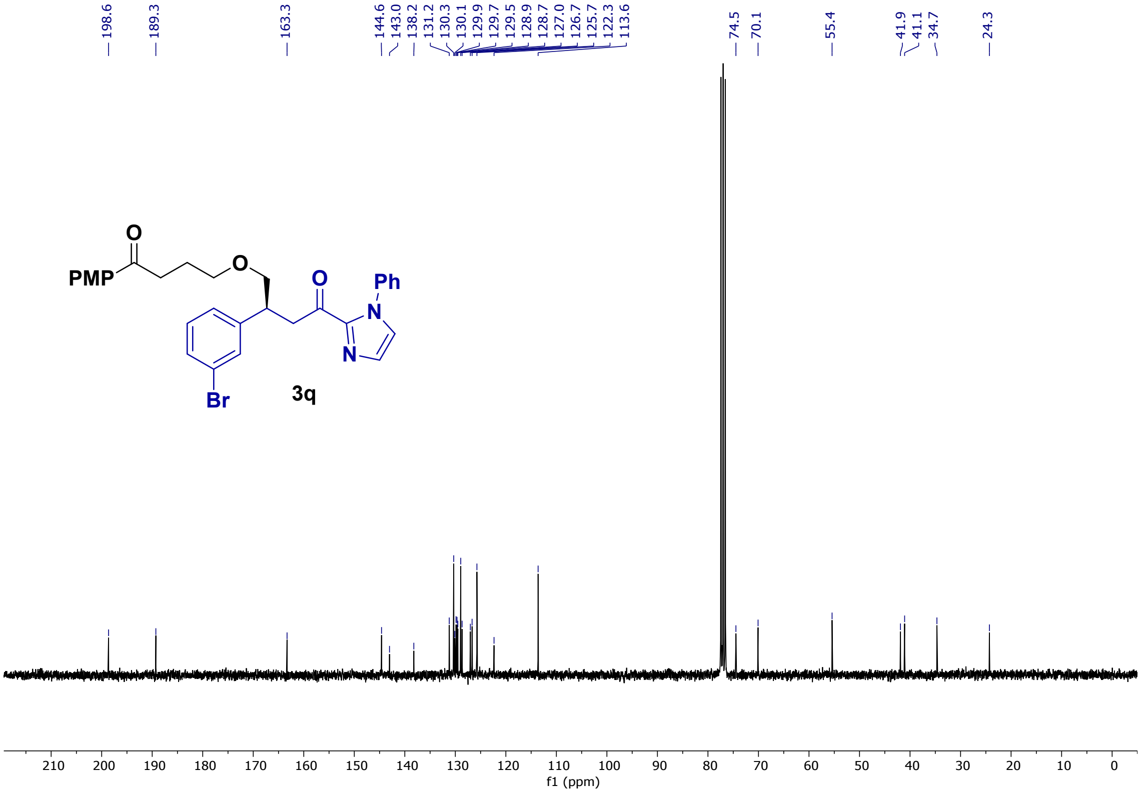

^{1}H NMR of **3r** (300 MHz, $CDCl_3$)

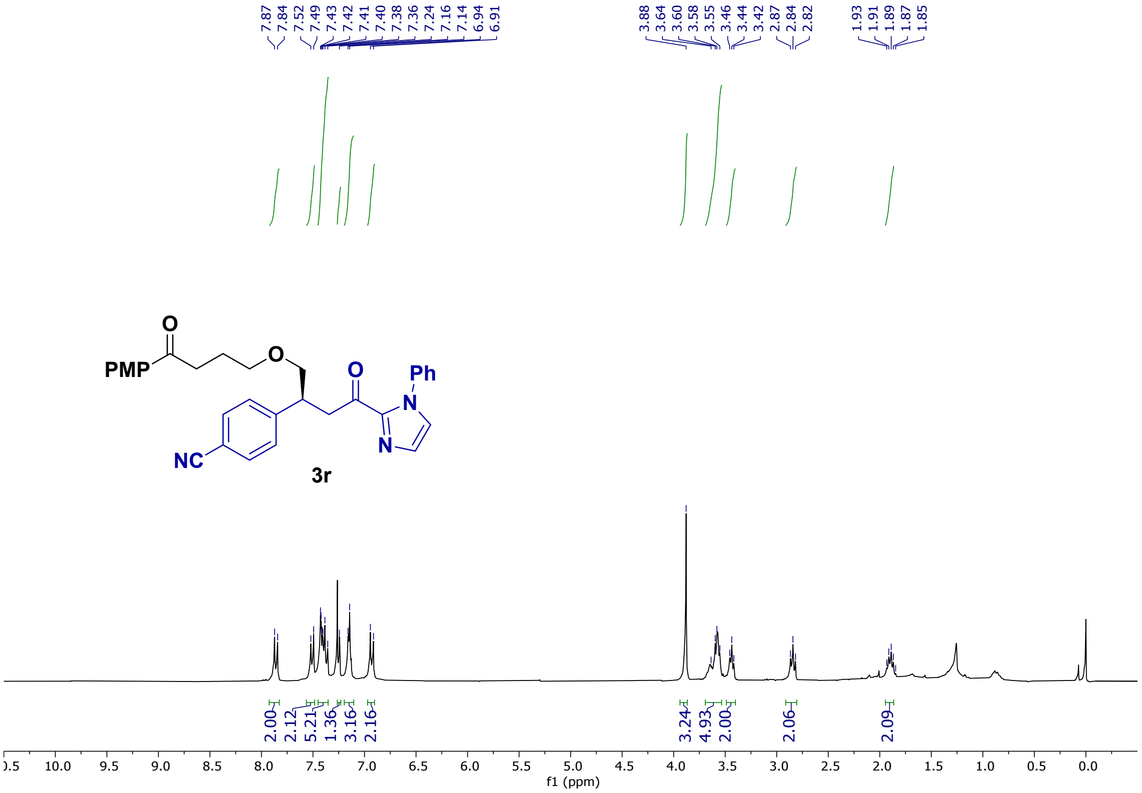

^{13}C NMR of **3r** (75 MHz, $CDCl_3$)

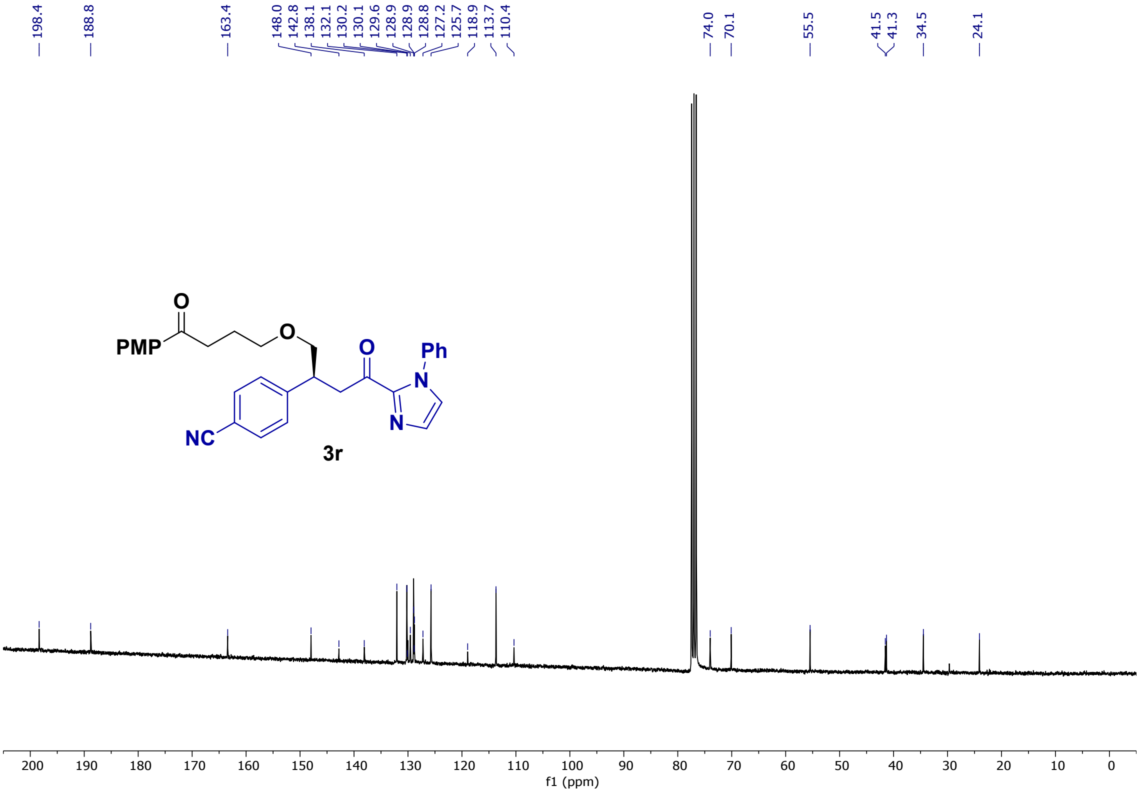

^{1}H NMR of **3s** (300 MHz, $CDCl_3$)

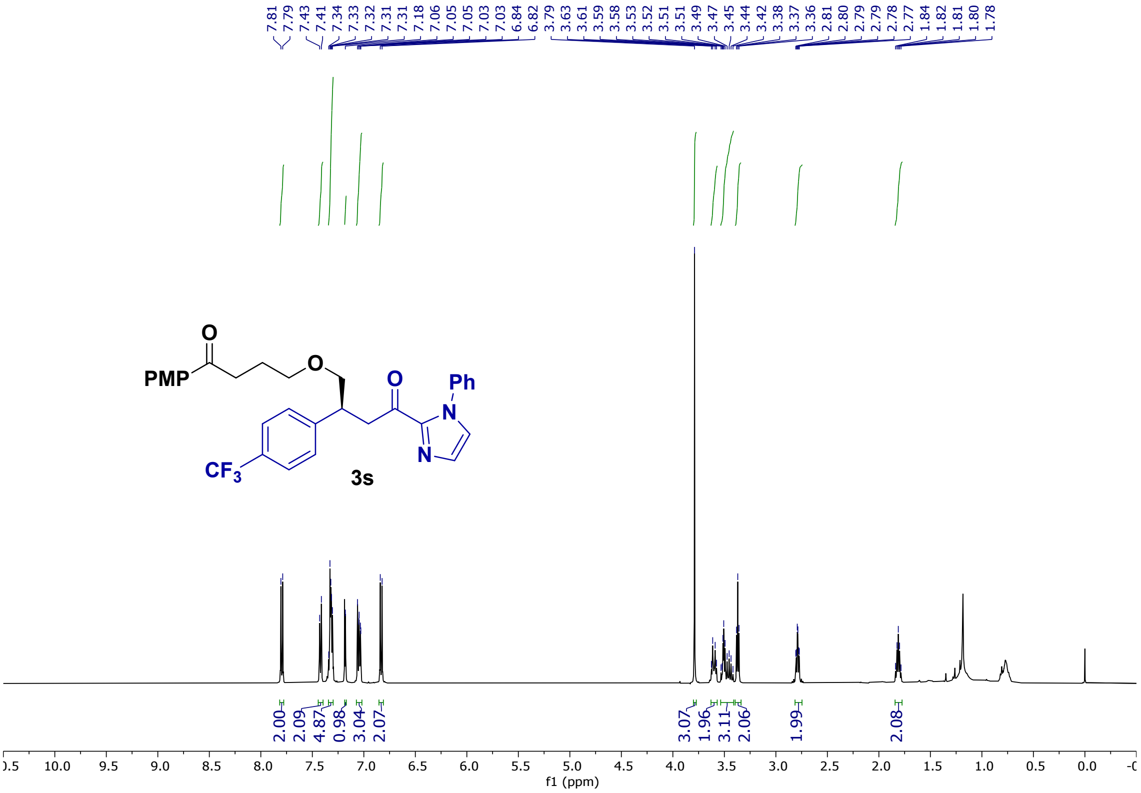

^{13}C NMR of **3s** (75 MHz, $CDCl_3$)

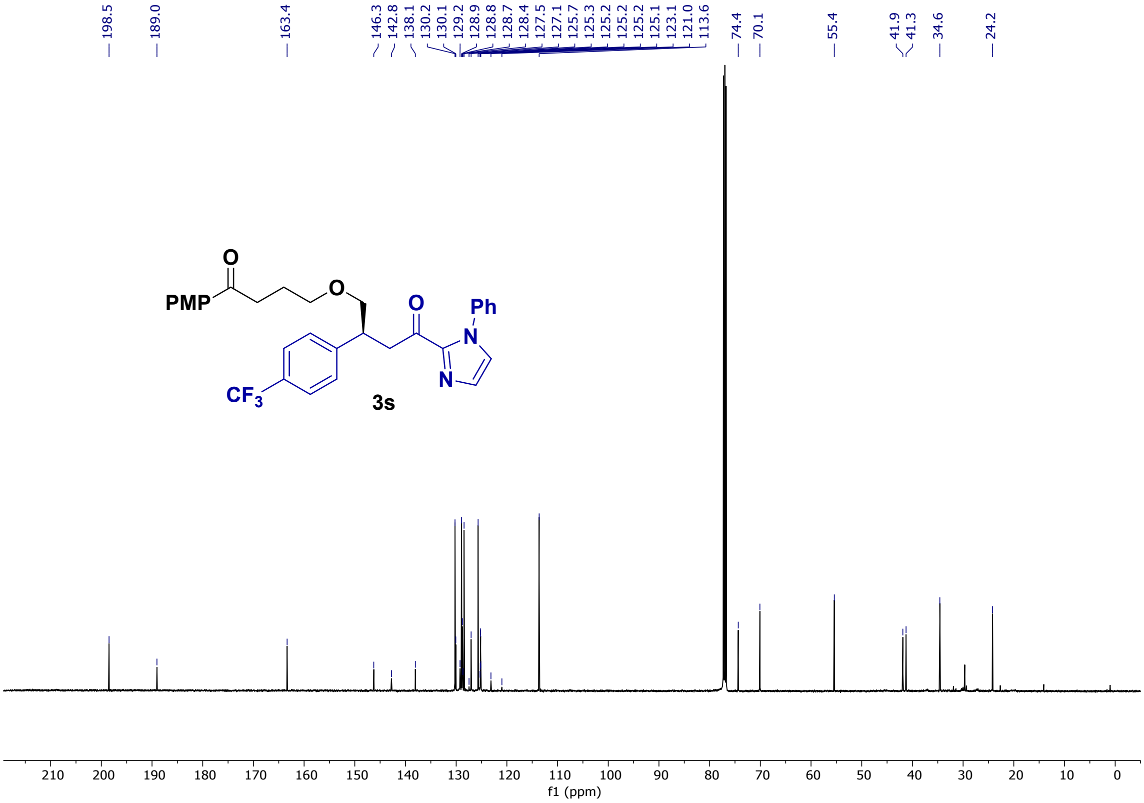

^{19}F NMR of **3s** (471 MHz, $CDCl_3$)

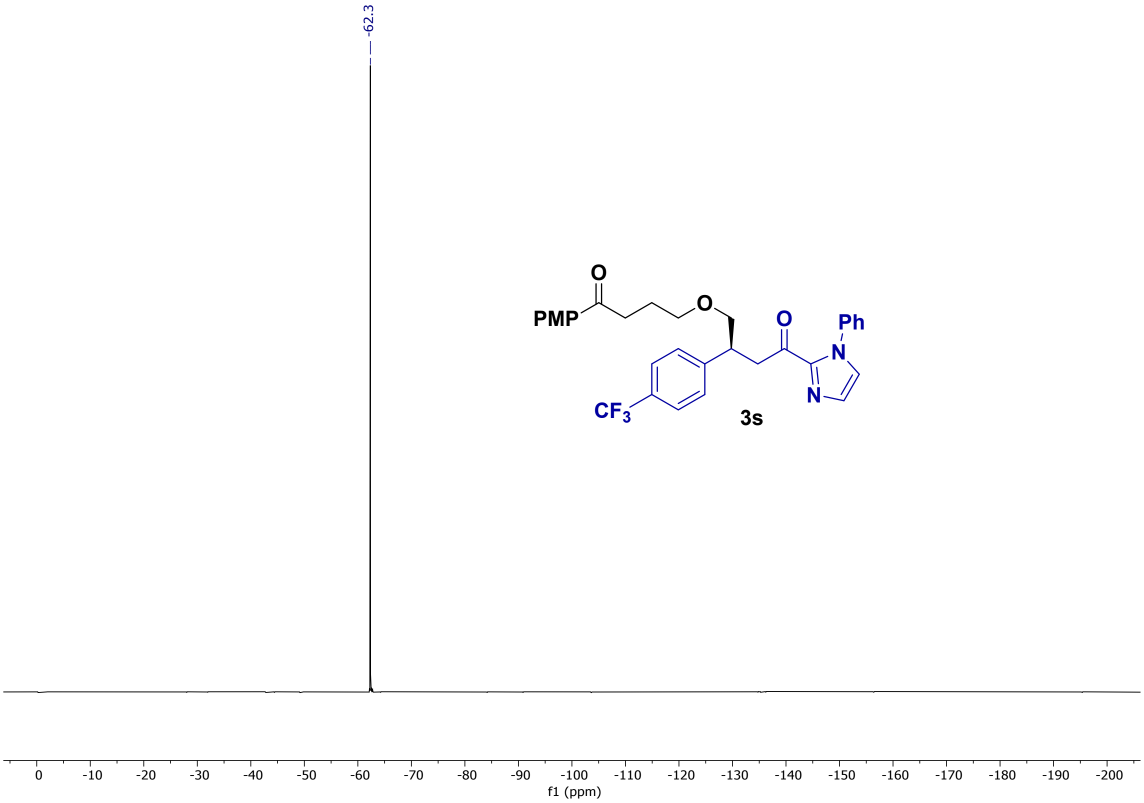

^{1}H NMR of **3u** (300 MHz, $CDCl_3$)

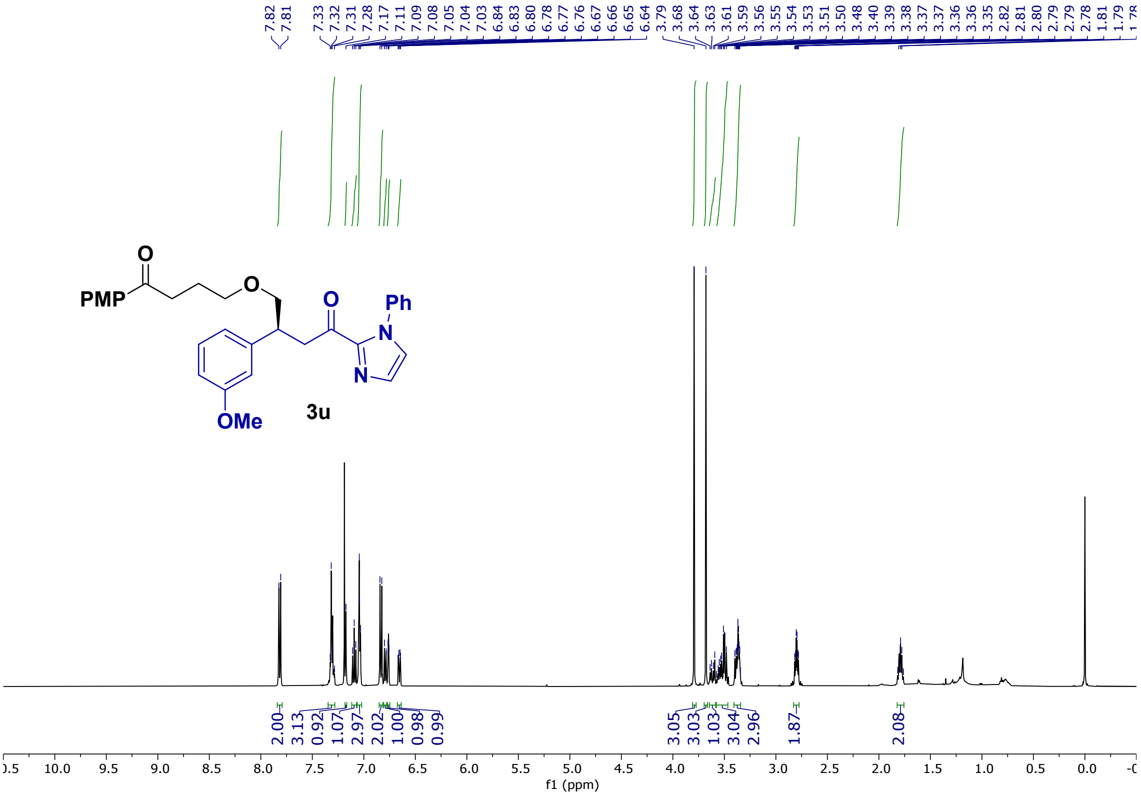

^{13}C NMR of **3u** (75 MHz, $CDCl_3$)

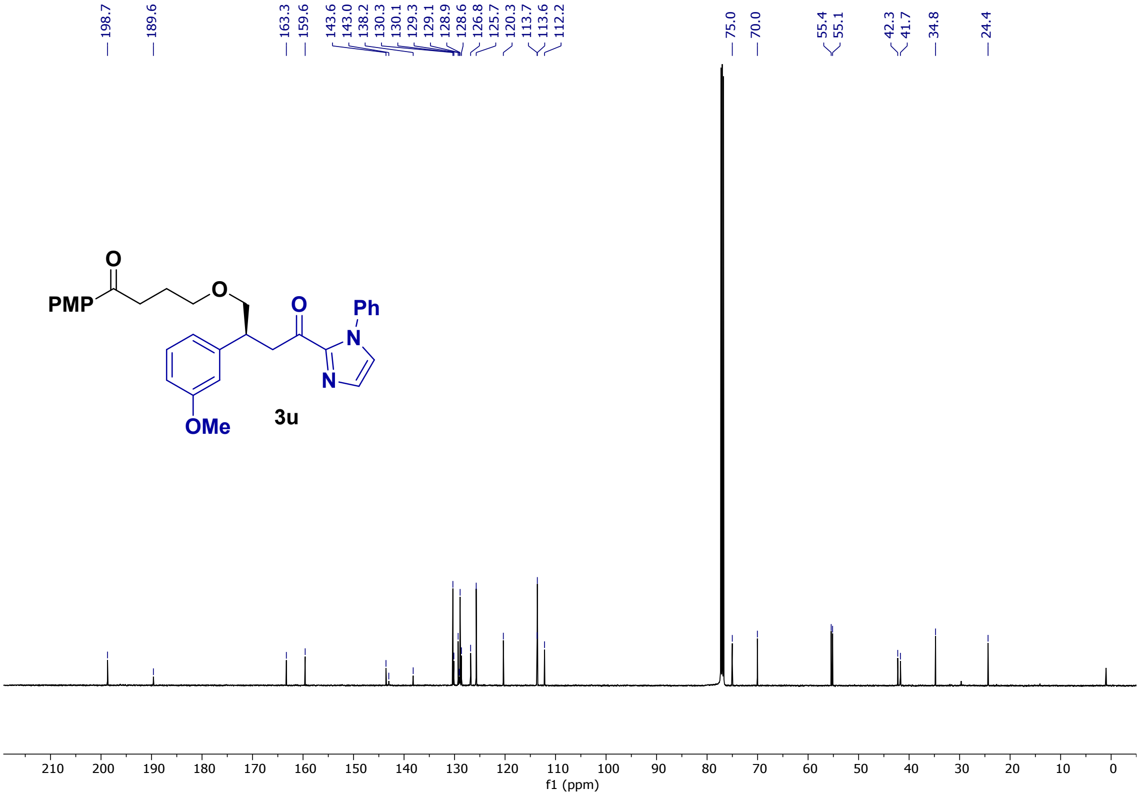

^{1}H NMR of **3v** (300 MHz, $CDCl_3$)

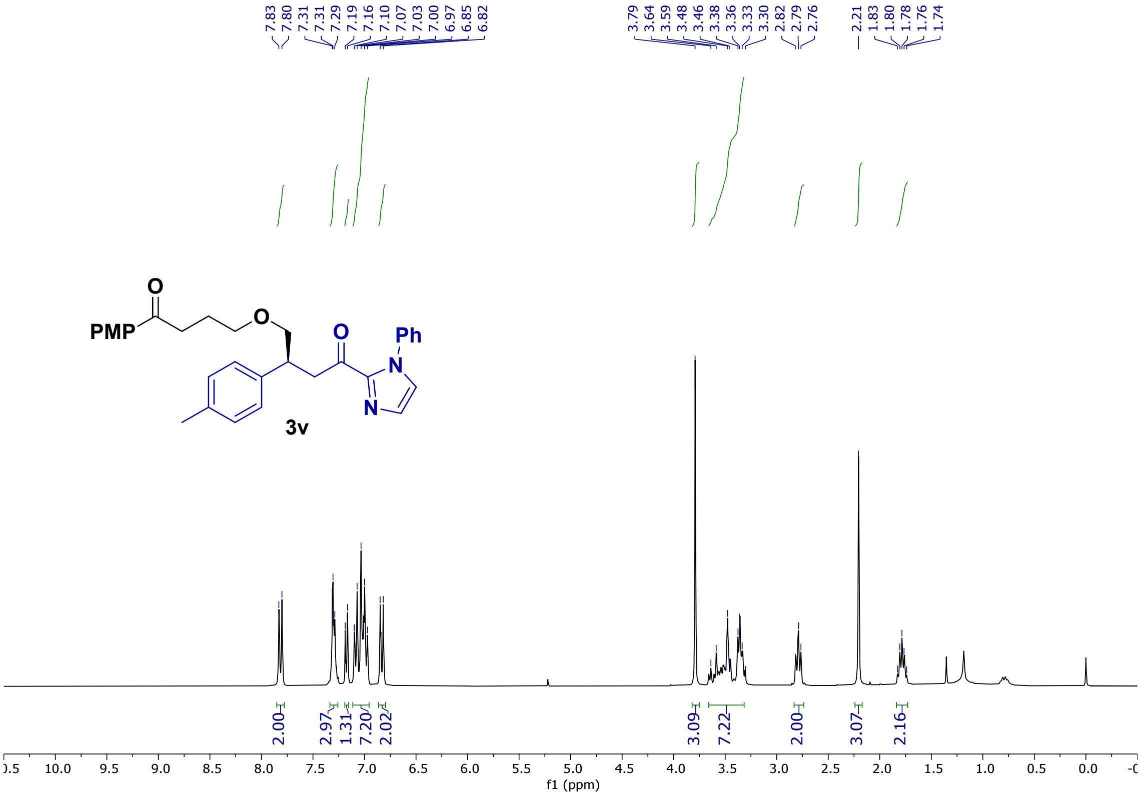

^{13}C NMR of **3v** (75 MHz, $CDCl_3$)

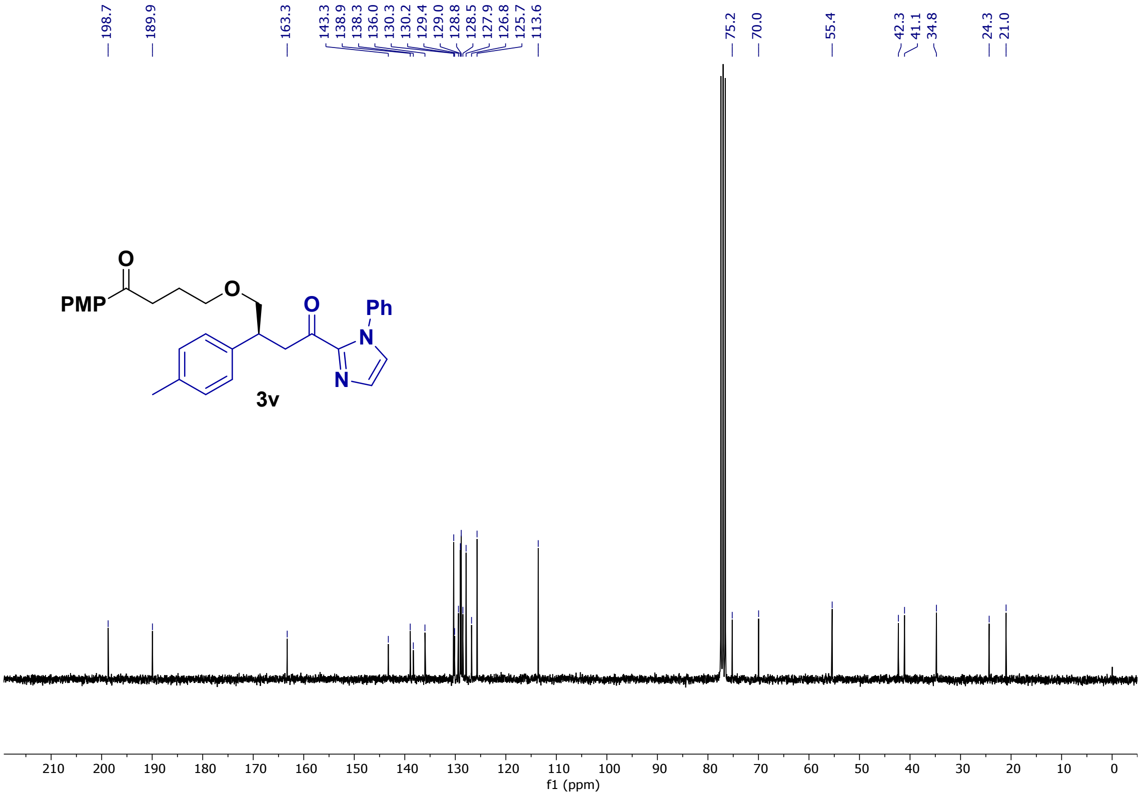

^{1}H NMR of **3w** (300 MHz, $CDCl_3$)

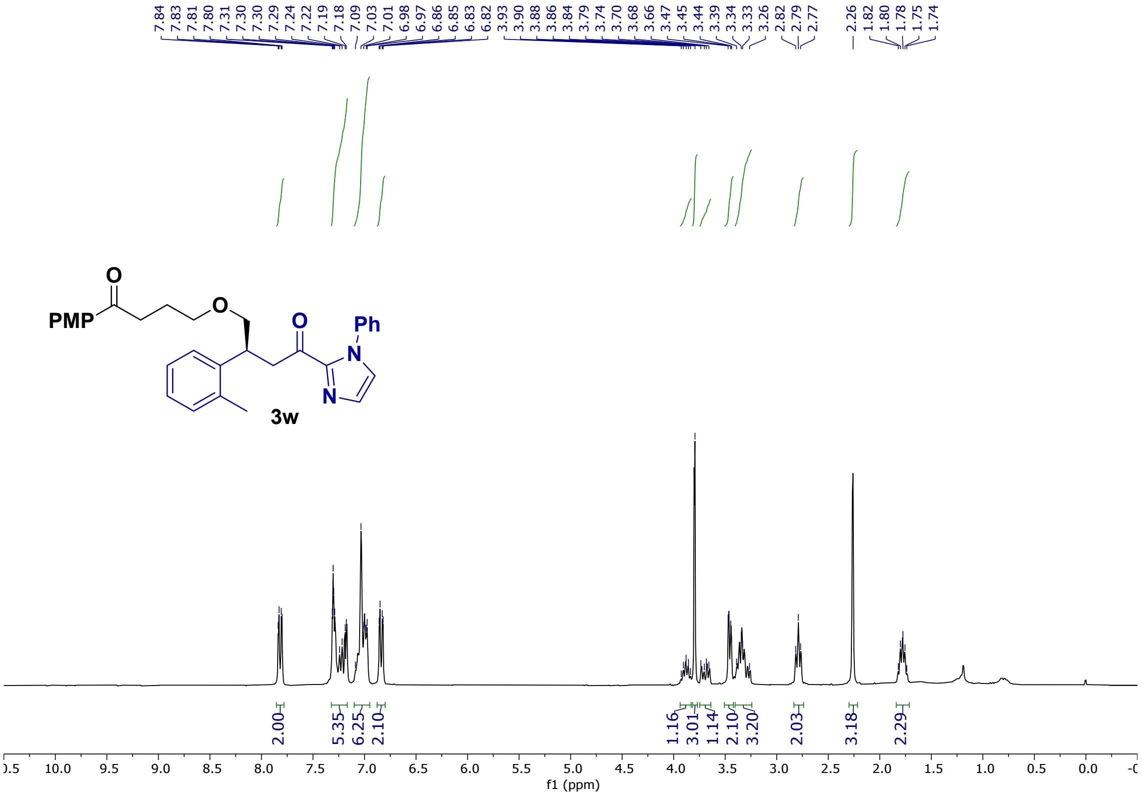

^{13}C NMR of **3w** (75 MHz, $CDCl_3$)

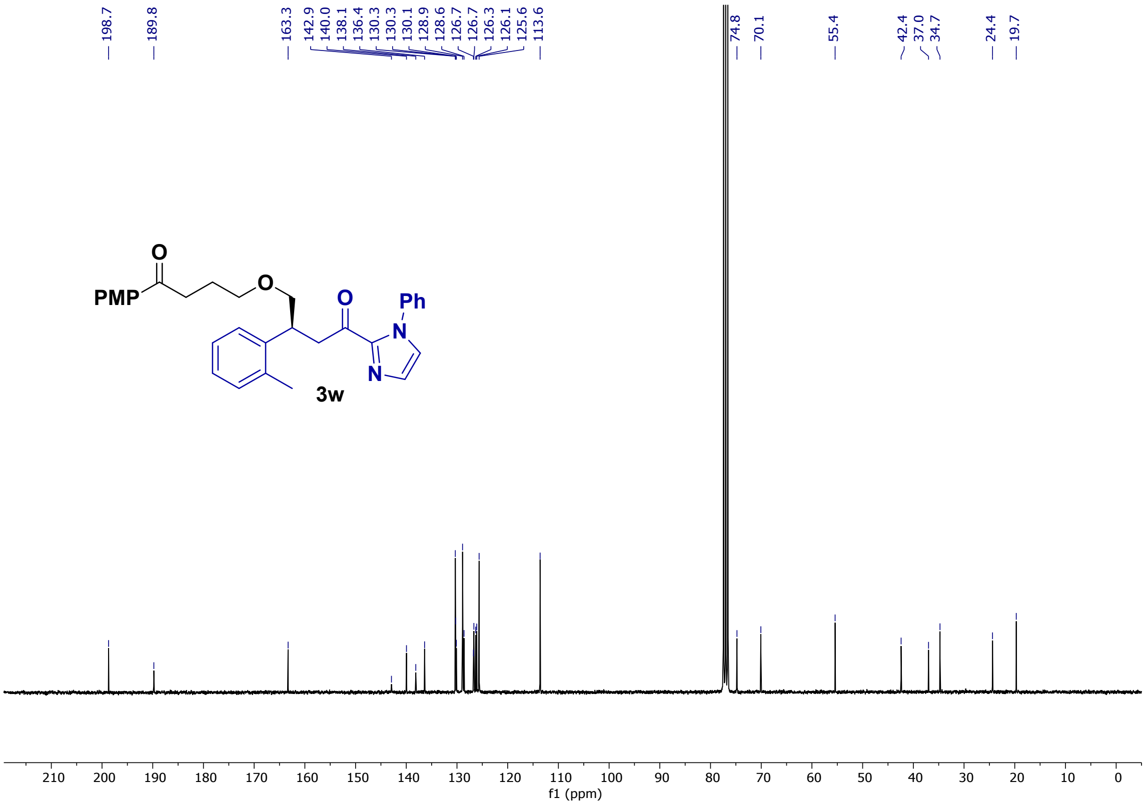

^{1}H NMR of **3x** (300 MHz, $CDCl_3$)

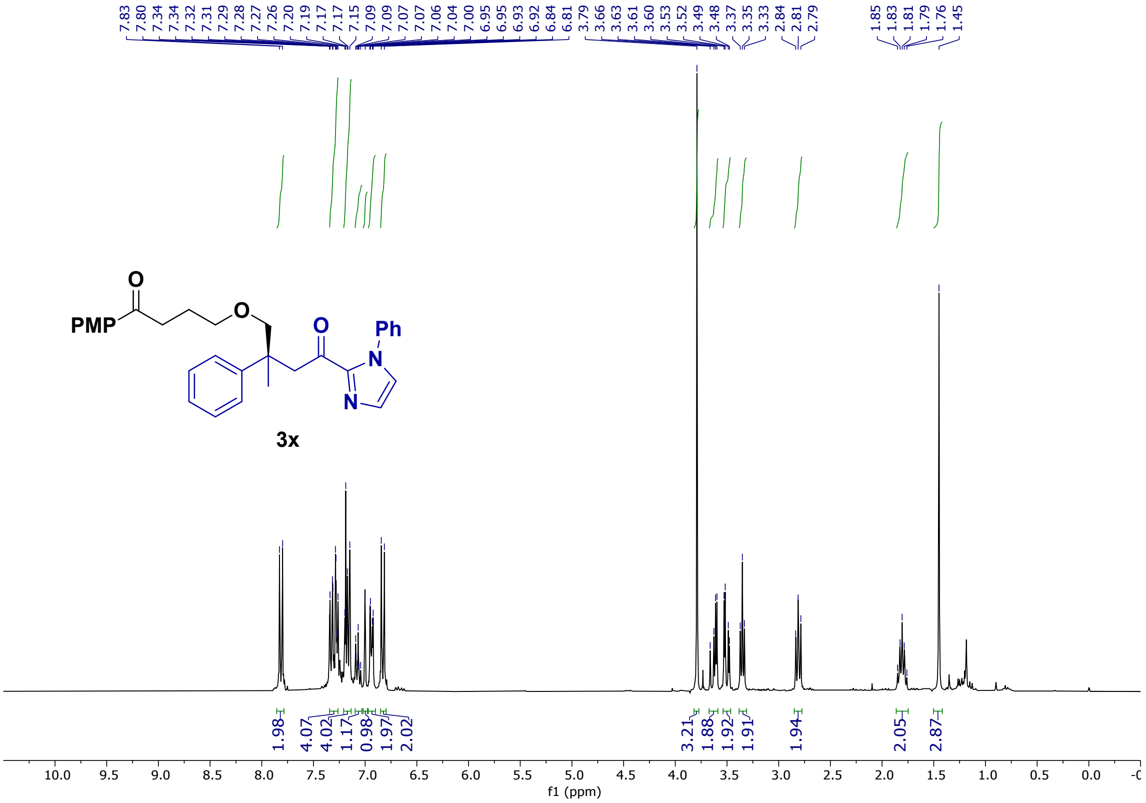

^{13}C NMR of **3x** (75 MHz, $CDCl_3$)

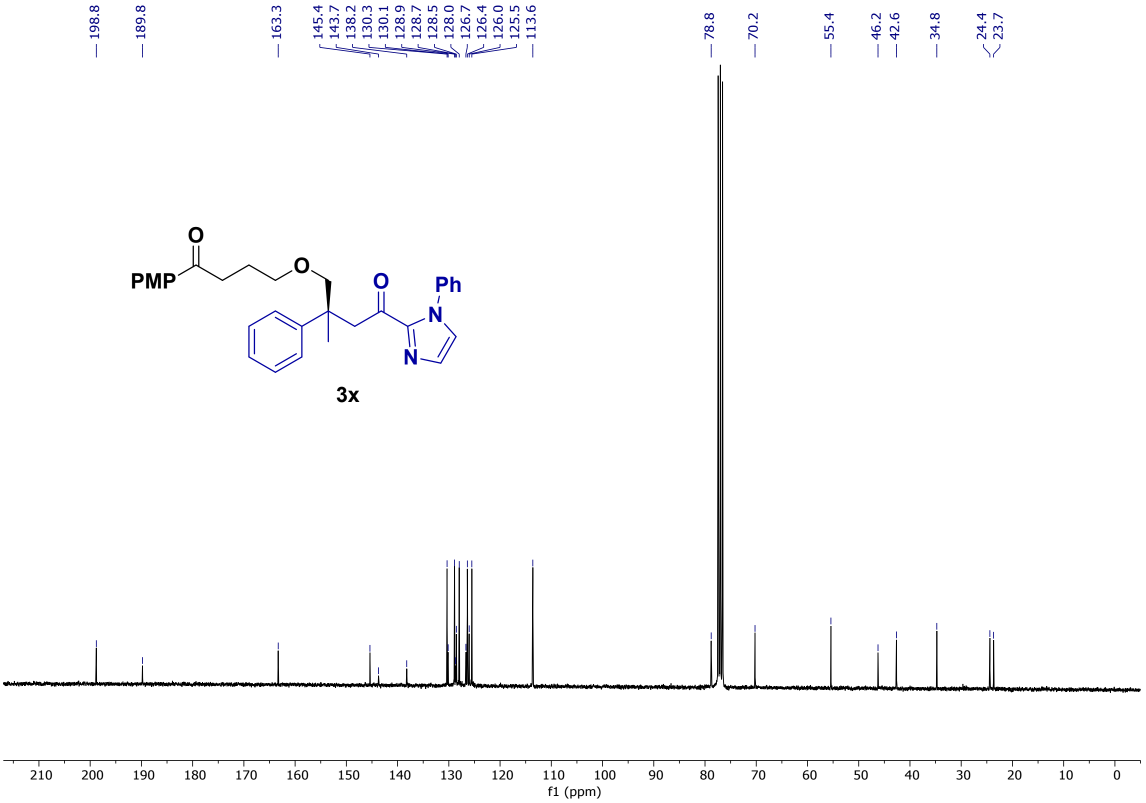

^{1}H NMR of **3y** (300 MHz, $CDCl_3$)

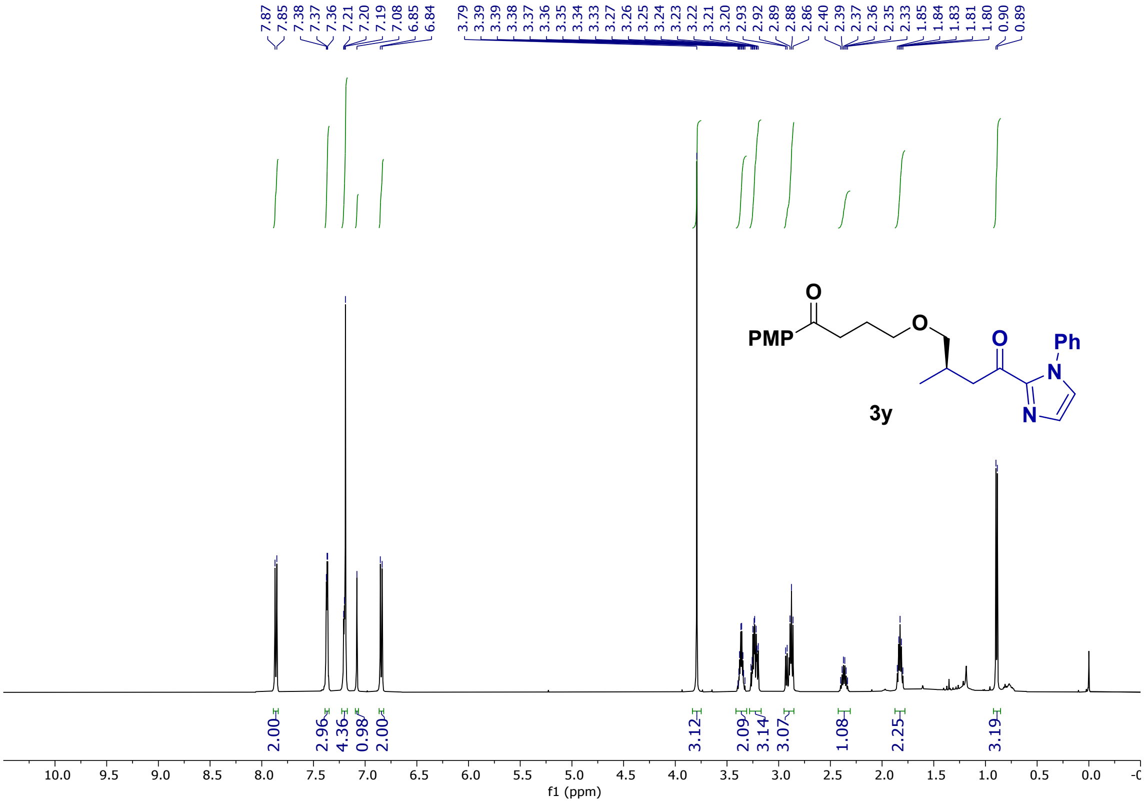

^{13}C NMR of **3y** (75 MHz, $CDCl_3$)

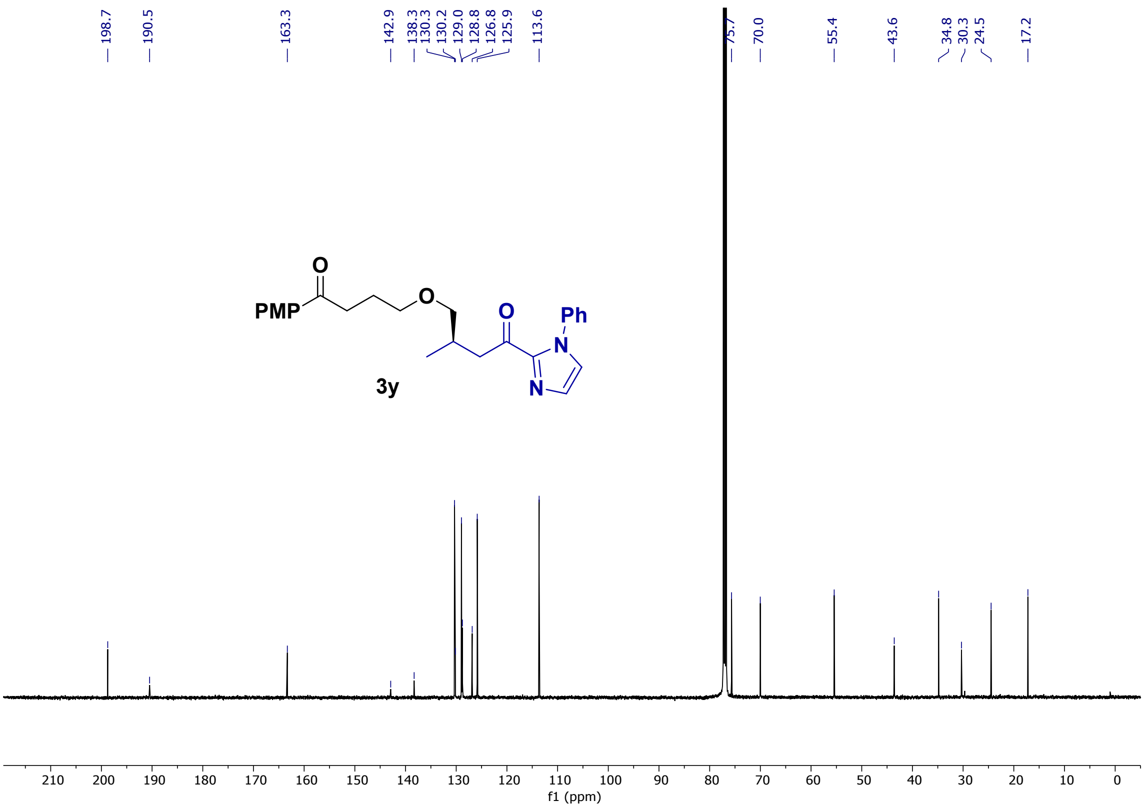

^{1}H NMR of **3z** (300 MHz, $CDCl_3$)

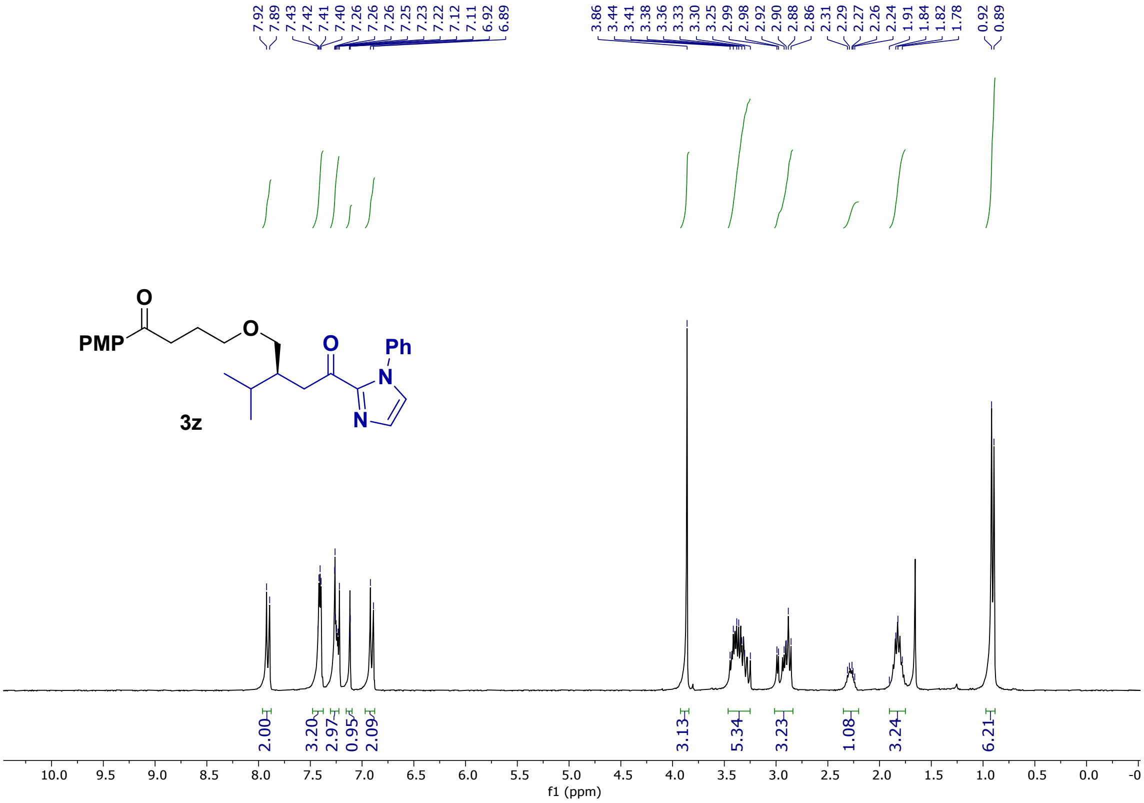

^{13}C NMR of **3z** (75 MHz, $CDCl_3$)

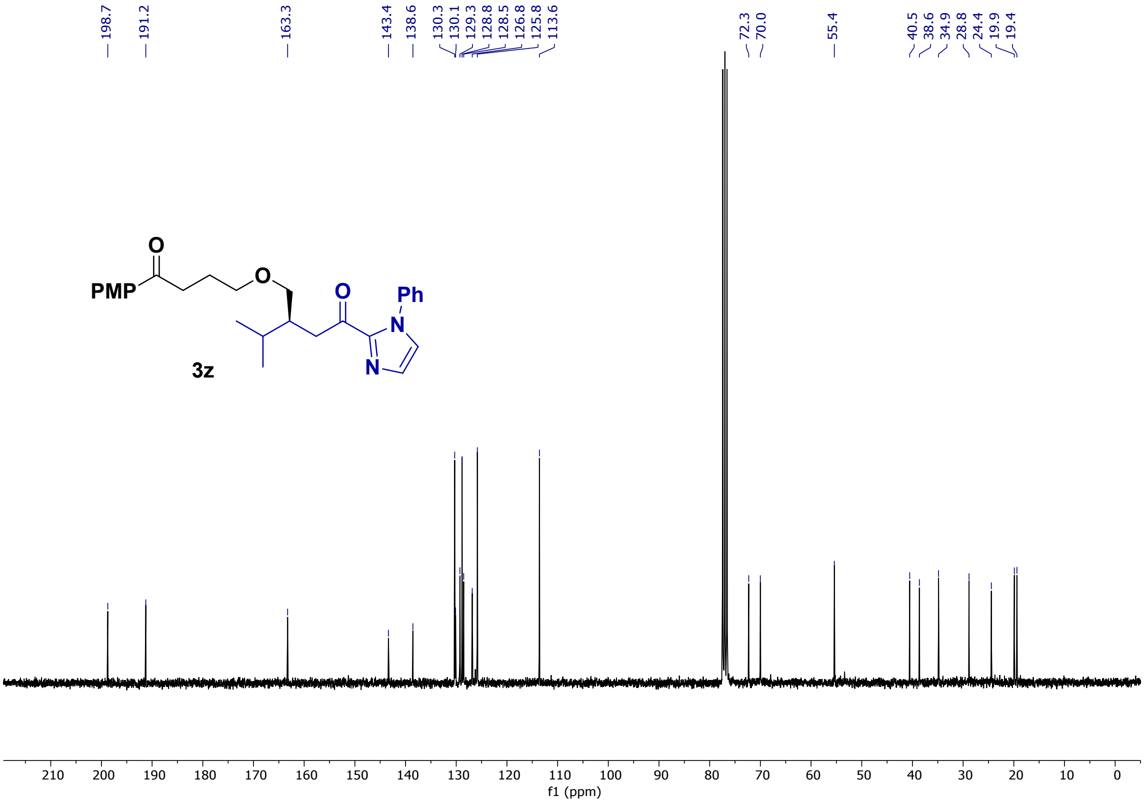

^{1}H NMR of **4** (300 MHz, $CDCl_3$)

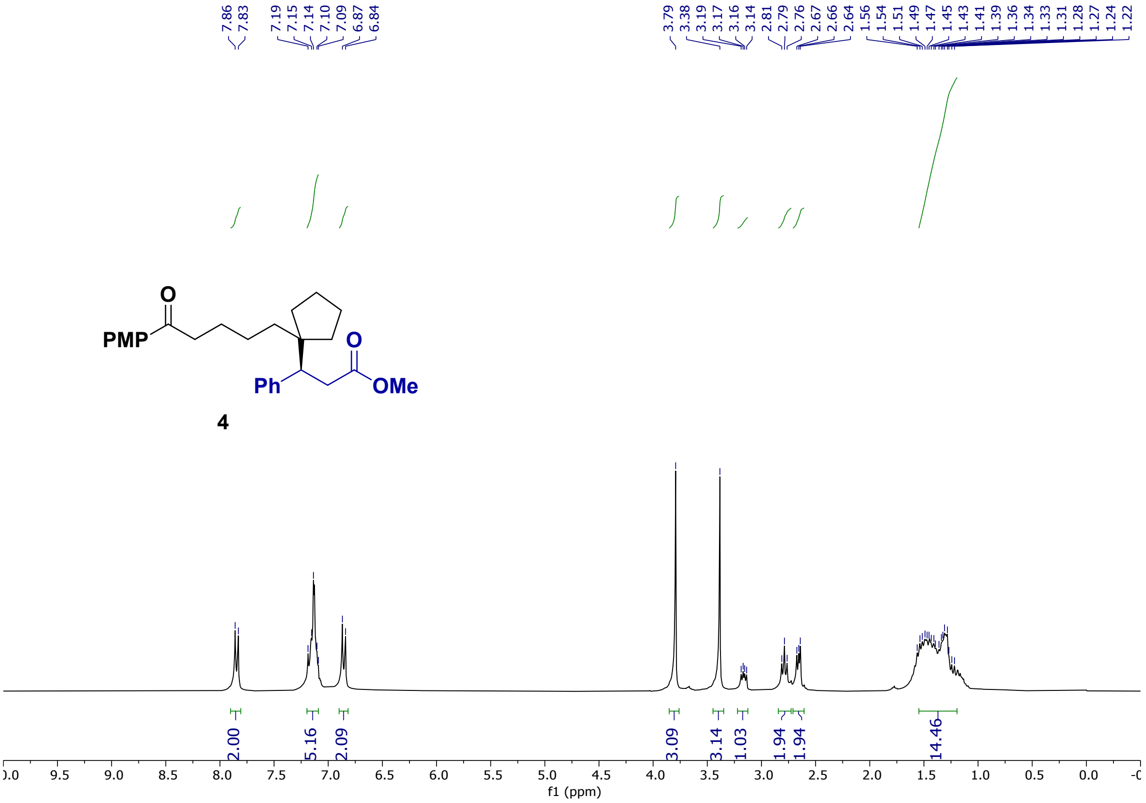

^{13}C NMR of **4** (75 MHz, $CDCl_3$)

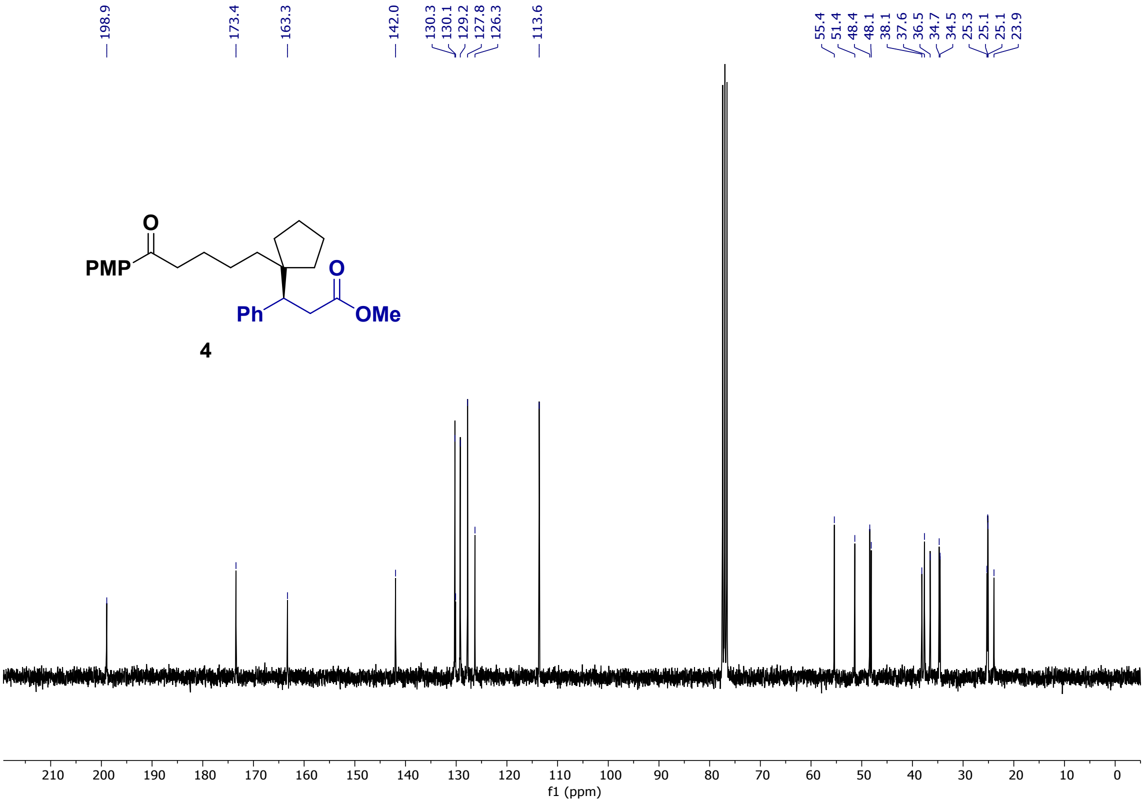

^{1}H NMR of **1ab** (300 MHz, $CDCl_3$)

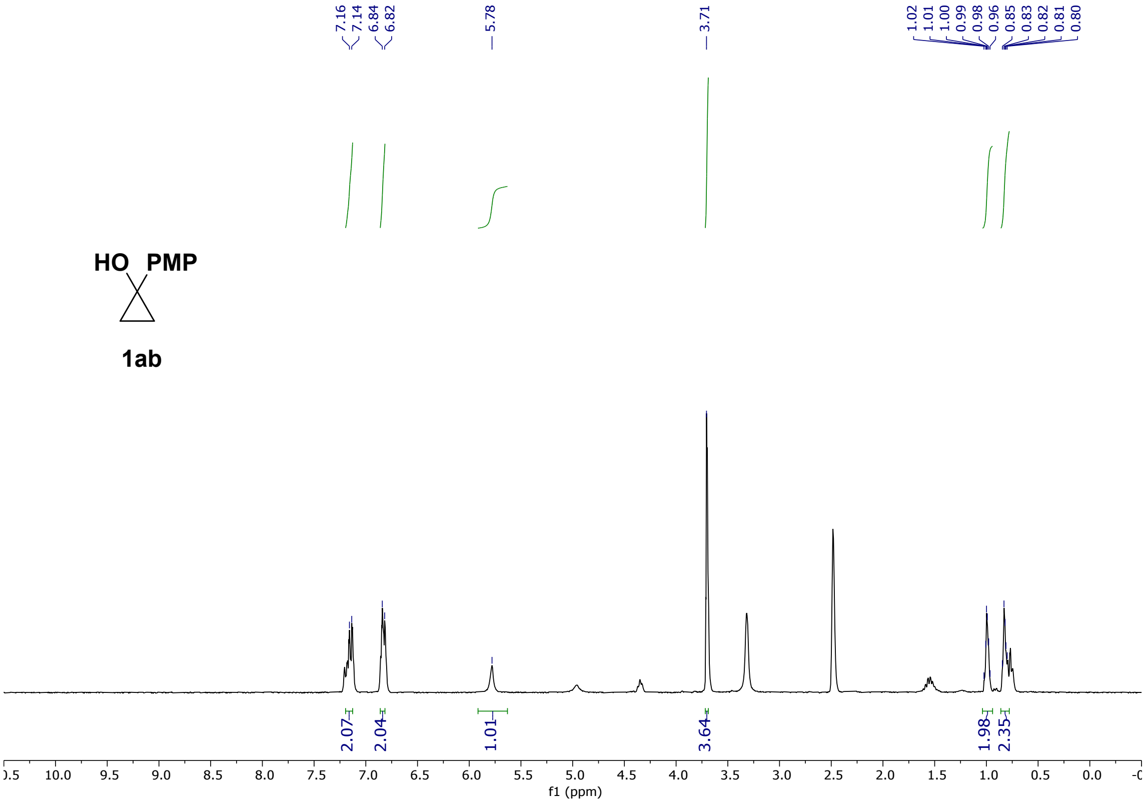

^{1}H NMR of **SI14** (300 MHz, $CDCl_3$)

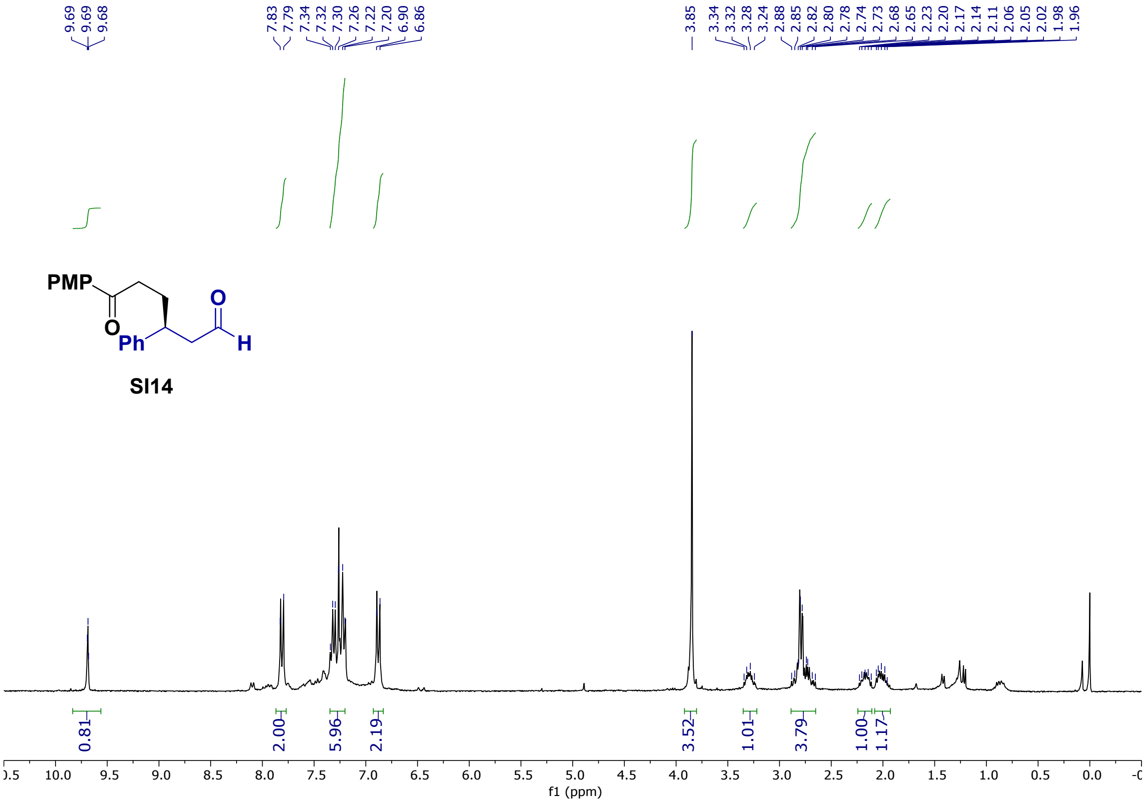

^1H NMR of **4ab** (300 MHz, $CDCl_3$)

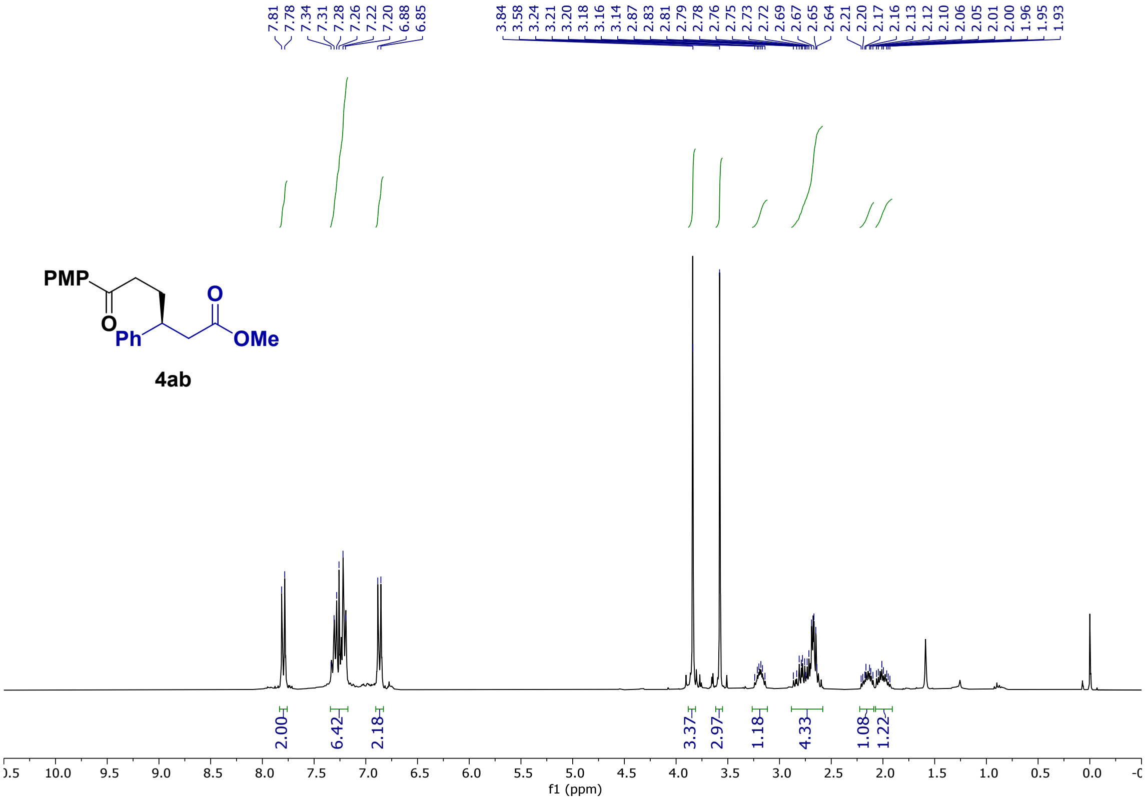

^{13}C NMR of **4ab** (75 MHz, $CDCl_3$)

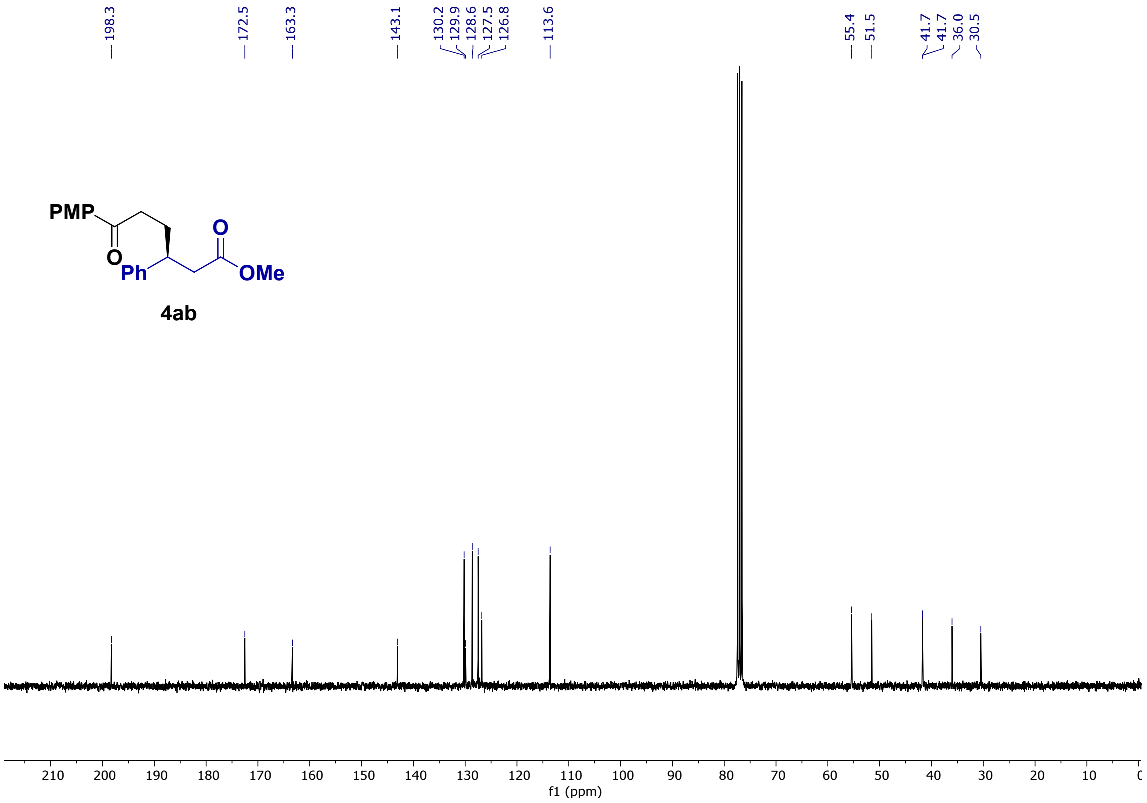

^{1}H NMR of **3ab** (300 MHz, $CDCl_3$)

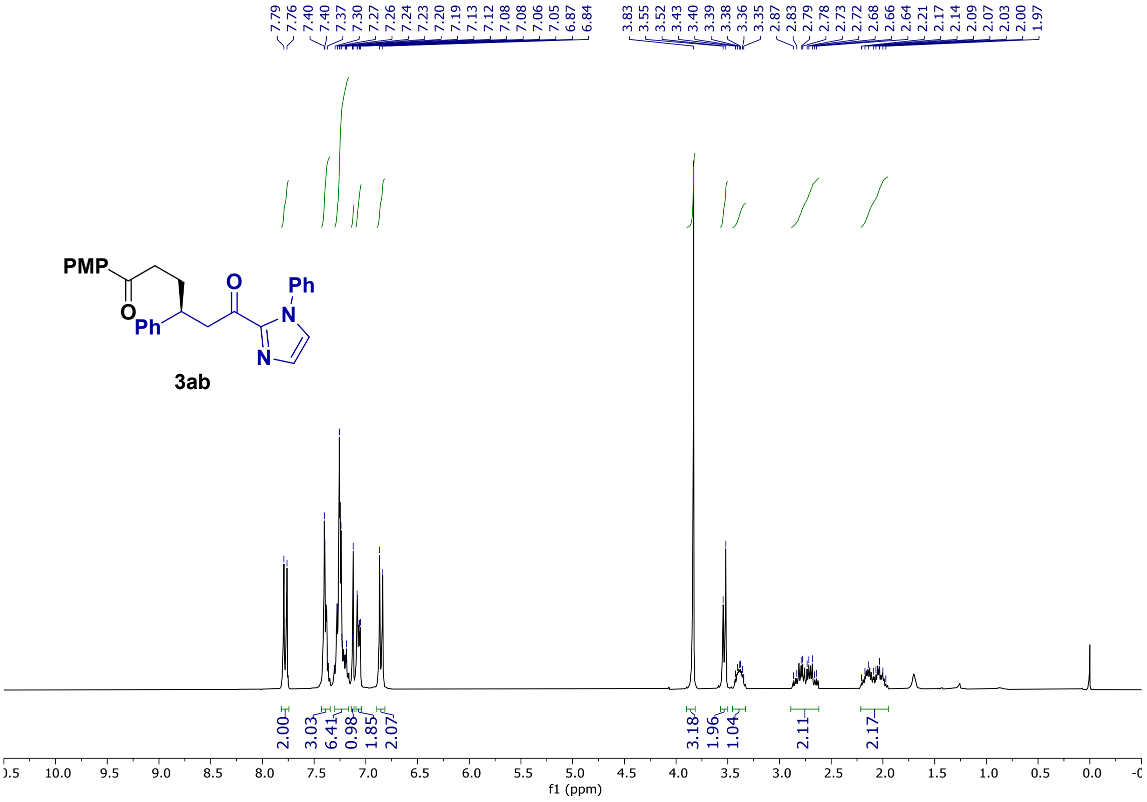

^{13}C NMR of **3ab** (75 MHz, $CDCl_3$)

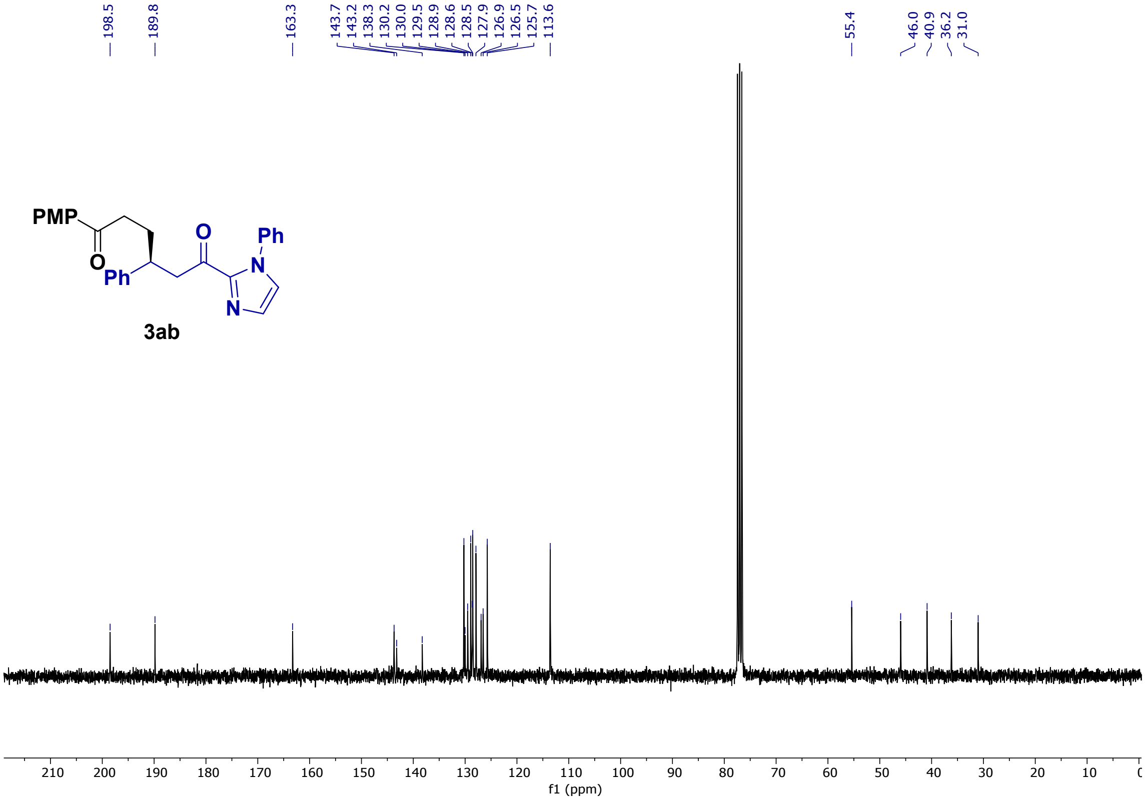

10. References

1. R. Soni, J.-M. Collinson, G. C. Clarkson, and M. Wills, *Org. Lett.* **2011**, 13, 4304 – 4307.

2. H. G. Yayla, H. Wang, K. T. Tarantino, H. S. Orbe, and R. R. Knowles, *J. Am. Chem. Soc*. **2016**, 138, 10794 – 10797.

3. R. A. Croft, J. J. Mousseau, C. Choi, J. A. Bull, *Chem. -A. Eur. J.* **2018**, 24, 818 – 821.

4. R. A. Croft, M. A. J. Dubois, A. J. Boddy, C. Denis, A. Lazaridou, A. S. Voisin-Chiret, R. Bureau, C. Choi, J. J. Mousseau, J. A. Bull, *Eur. J. Org. Chem*. **2019**, 31-32, 5385 – 5395.

5. L. De Luca, G. Giacomelli, and M. Taddei, *J. Org. Chem*. **2001**, 66, 2534 – 2537.

6. A. C. Silvanus, S. J. Heffernan, D. J. Liptrot, G. Kociok-Köhn, B. I. Andrews, and D. R. Carbery, *Org. Lett*. **2009**, 11, 1175 – 1178.

7. R. Shintani, T. Kimura and T. Hayashi, *Chem. Commun*, **2005**, 25, 3213 – 3214.

8. X. Huang, T. R. Quinn, K. Harms, R. D. Webster, L. Zhang, O. Wiest, and E. Meggers, *J. Am. Chem. Soc.* **2017**, 139, 9120 – 9123.

9. B. M. Trost and T. M. Lam, *J. Am. Chem. Soc*. **2012**, 134, 11319 – 11321.

10. D. A. Evans and K. R. Fandrick, *Org. Lett*. **2006**, 8, 2249 – 2252.

11. H. Huo, K. Harms, and E. Meggers, *J. Am. Chem. Soc*. **2016**, 138, 6936 – 6939.

12. H. J. Kuhn, S. E. Braslavsky, R. Schmidt, Name and symbol of the element with atomic number 111. *Pure Appl. Chem.* **2004**, *76*, 2105 – 2146.

13. S. Hamai, F. Hirayama, *J. Phys. Chem.* **1983**, *87*, 83 – 89.

14. E. Le Saux, M. Dengke, P. Bonilla, C. M. Holden, D. Lustosa, P. Melchiorre, *Angew. Chem. Int. Ed.* **2021**, 60, 5357 – 5362.

15. X.-P. He, Y.-J. Shu, J.-J. Dai, W.-M. Zhang, Y.-S. Feng, H.-J. Xu, *Org. Biomol. Chem*., **2015**, 13, 7159 – 7163.

16. R. I. Rodríguez, M. Sicignano, J. Alemán, *Angew. Chem. Int. Ed*, **2022**, e202112632.

17. C. F. Weise, V. H. Lauridsen, R. S. Rambo, E. H. Iversen, M.-L. Olsen, and K. A. Jørgensen, *J. Org. Chem*. **2014**, *79*, 3537 – 3546.
